# High‐Affinity Peptide‐Drug Conjugate Ligands for the TRIM24 PHD and Bromodomain

**DOI:** 10.1002/chem.202503011

**Published:** 2025-11-17

**Authors:** Michael A. Platt, Ekaterina Kot, Louise A. W. Martin, Antoine L. D. Wallabrègue, Liwen Song, Alistair M. Boyd, Lizbé Koekemoer, Ester M. Hammond, Stuart J. Conway

**Affiliations:** ^1^ Department of Chemistry, University of Oxford Chemistry Research Laboratory Mansfield Road Oxford OX1 3TA U.K; ^2^ Centre for Medicines Discovery, University of Oxford Nuffield Department of Medicine Research Building Roosevelt Drive Oxford OX3 7FZ U.K; ^3^ Department of Oncology, University of Oxford Nuffield Department of Medicine Research Building Roosevelt Drive Oxford OX3 7FZ U.K; ^4^ Department of Chemistry & Biochemistry University of California, Los Angeles 607 Charles E. Young Drive East Los Angeles California 90095 U.S.A

**Keywords:** bromodomains, peptide‐drug conjugates, peptidomimetics, plant homeodomains, protein‐protein interactions

## Abstract

TRIM24 is an epigenetic transcriptional coregulator that “reads” KMe_3_ and KAc histone modifications via its tandem plant homeodomain (PHD) and bromodomain (BRD), respectively. The PHD and BRD are potential therapeutic targets due to the roles of TRIM24 in breast cancer progression. However, there are currently no small‐molecule ligands for the PHD, and existing TRIM24 BRD inhibitors lack selectivity over the main off‐target, BRPF1. Here, we report the development of the first bivalent tool molecules capable of simultaneously engaging both the TRIM24 PHD and BRD. Key to this strategy was the identification of effective KMe_3_ bioisosteres that enhance H3 peptide binding to the TRIM24 PHD. The most promising of these was incorporated into a nine amino acid H3‐mimicking peptide, and linked to a TRIM24 BRD ligand. The resulting peptide‐drug conjugates (PDCs) bind to TRIM24 with picomolar affinity and a slow dissociation rate (*k*_off_), which is driven by an *in cis* bivalent binding mode. Although the PDCs showed limited effects on breast cancer cell proliferation in vitro, this work underscores their potential as tools for studying previously unliganded reader domains and consequently advancing our understanding of multivalent epigenetic regulation in disease.

## Graphical abstract

We report a series of bivalent peptide‐drug conjugate (PDC) ligands for tripartite motif‐containing protein 24 (TRIM24). These ligands simultaneously engage the PHD and BRD of TRIM24 through an intramolecular (*in cis*) binding mode, resulting in high affinity binding. By incorporating a trimethyllysine (KMe_3_) mimic and a BRD ligand, these PDCs can disrupt the multivalent chromatin interactions of TRIM24, resulting in complete inhibition of its epigenetic reader function.

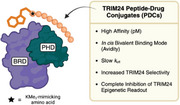

**Abbreviations**
KAc: acetylated lysine
AR: androgen receptor
BRD: bromodomain
BRPF1: bromodomain and PHD finger‐containing protein 1
DTT: dithiothreitol
ER: oestrogen receptor
GST: glutathione‐S‐transferase
HFIP: hexafluoroisopropanol
H3: histone 3
PDC: peptide‐drug conjugate
PHD: plant homeodomain
PDB: protein data bank
PROTAC: proteolysis targeting chimera
SPPS: solid phase peptide synthesis
SPR: surface plasmon resonance
TFA: trifluoroacetic acid
KMe_3_
: trimethylated lysine
TRIM: tripartite motif‐containing protein

## 1 Introduction

**Figure 1:**
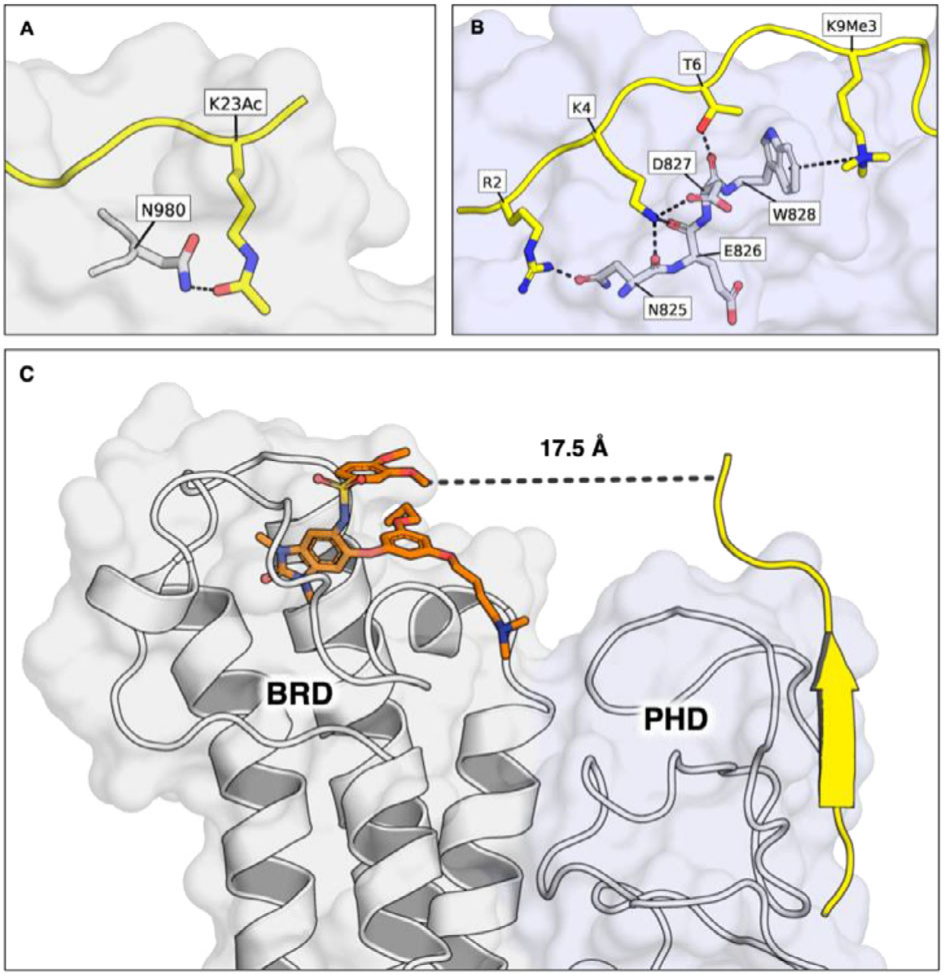
**A)**. An *apo* crystal structure of TRIM24 PHD‐BRD (1.7 Å, PDB ID: 9GD5, carbon = white) overlaid with the peptide (carbon = yellow) from the crystal structure of TRIM24 in complex with H3_(13–32)_K23Ac (3O34^[^ ^4^ ^]^). **B)**. An *apo* crystal structure of TRIM24 PHD‐BRD (1.7 Å, PDB ID: 9GD5, carbon = light blue) overlaid with the peptide (carbon = yellow) from the crystal structure of TRIM33α (which has an identical PHD domain) in complex with H3_(1–22)_K9Me_3_K14AcK18Ac (3U5O^[^ ^12^ ^]^). **C)**. The X‐ray crystal structure of the TRIM24 PHD‐BRD in complex with IACS‐9571 (PDB ID: 4YC9,^[^ ^13^ ^]^ carbon = orange) overlaid with the peptide (carbon = yellow) from the crystal structure of TRIM24 in complex with H3_(1–10)_ (PDB ID: 3O37^[^ ^4^ ^]^). The distance shown is between K9 ($C_{\alpha }$) on the H3 peptide and a solvent‐exposed methoxy group of IACS9571. The BRD and PHD domains are shaded in white and light blue, respectively.

Tripartite motif‐containing protein 24 (TRIM24) is an epigenetic reader protein involved in the coregulation of transcription factors, including the androgen (AR) and oestrogen (ER) receptors.^[^ ^1^, ^2^, ^3^ ^]^ Its epigenetic reader function is mediated by a C‐terminal tandem plant homeodomain (PHD) and bromodomain (BRD), which was previously reported to recognize unmethylated histone 3 lysine 4 (H3K4) and acetylated histone 3 lysines 18 or 23 (H3K18/23Ac), respectively (Figure 1A, 1B).^[^ ^4^ ^]^ Previous studies from our group, and that of Mann,^[^ ^5^ ^]^ have shown that TRIM24 can also bind to trimethylated histone 3 lysine 9 (H3K9Me_3_) via a cation‐π interaction with a solvent‐exposed tryptophan (W828) in the PHD (Figure 1B), further enhancing its affinity for chromatin.^[^ ^6^ ^]^ Interestingly, studies have shown that the PHD and BRD can bind H3K4K23Ac or H3K9Me_3_K18Ac with high affinity, suggesting that TRIM24 can simultaneously recognize multiple epigenetic marks on a single histone tail.^[^ ^4^, ^6^ ^]^

TRIM24 overexpression has been implicated in the development and progression of breast and prostate cancer.^[^ ^4^, ^7^ ^]^ It is hypothesized that TRIM24 promotes proliferation in ER^+^ breast cancer by recruiting the oestrogen receptor to chromatin, leading to the upregulation of oestrogen‐dependent oncogenes.^[^ ^4^ ^]^ This process is mediated through combinatorial recognition of H3K4K23Ac by the PHD‐BRD cassette.^[^ ^4^ ^]^ A separate study identified a unique role of the PHD and BRD in promoting breast cancer cell adhesion, through SUMOylation of TRIM24.^[^ ^8^ ^]^ This finding suggests an alternative role for TRIM24 in certain types of breast cancer. Given the involvement of TRIM24 in cancer progression, its PHD and BRD present promising targets for therapeutic intervention.

Like the majority of PHD domains, the TRIM24 PHD has a low predicted ligandability and druggability due to its surface groove‐mediated histone recognition and lack of a well‐defined binding pocket (Figure 1B, 1C).^[^ ^9^ ^]^ However, in contrast to most KMe_3_‐interacting PHDs it also lacks an aromatic cage for KMe_3_ recognition, further limiting its tractability.^[^ ^6^, ^10^, ^11^ ^]^ As a result, it is unsurprising that no chemical tools have been reported for the TRIM24 PHD to date.

In contrast to the PHD, the TRIM24 BRD is more amenable to small molecule binding, due to its deep hydrophobic KAc‐binding pocket, which is typical of BRDs (Figure 1C).^[^ ^14^ ^]^ Two high‐affinity TRIM24 BRD inhibitors containing a benzimidazolone KAc mimic, have been developed (Supplementary Figure S1), with IACS‐9571 demonstrating the highest affinity for TRIM24 (*K*_d_ = 31 nM).^[^ ^13^, ^15^ ^]^ Despite their high affinity, neither ligand was selective over BRPF1 (Supplementary Figure S1), which shares ∼40% amino acid sequence identity (NCBI Blast) in its BRD with that of TRIM24, and displayed negligible effects on breast cancer cell proliferation.^[^ ^13^, ^15^, ^16^, ^17^ ^]^ In recent years, a number of alternative scaffolds and KAc mimics have been explored for TRIM24 BRD ligands (Supplementary Figure S1), however, these compounds have low affinity for TRIM24 and remained unselective over BRPF1.^[^ ^18^, ^19^ ^]^ A proteolysis targeting chimera (PROTAC; dTRIM24), derived from the benzimidazolone‐containing BRD ligands, which selectively degrades TRIM24 (Supplementary Figure S1), has also been developed.^[^ ^20^, ^21^ ^]^ Interestingly, while small molecule BRD inhibitors demonstrated no phenotypic effects on cancer cells, dTRIM24 did show antiproliferative effects. This suggests that inhibition of the BRD alone is insufficient to hinder the role of TRIM24 in cancer cells, but that other domains, including the PHD, could be involved in regulating cancer cell proliferation.^[^ ^20^, ^21^ ^]^ This has previously been observed in the BRD‐containing proteins SMARCA2 and SMARCA4, where inhibition of the BRD has limited cellular effects but degradation of the whole protein, including the ATPase domain, leads to cell death.^[^ ^22^ ^]^

Given the important role of the TRIM24 PHD in breast and prostate cancer, and the intriguing potential for interplay with the BRD, we investigated whether it was possible to develop chemical tools capable of inhibiting both the BRD and PHD. While the PHD is generally considered hard to ligand with small molecules, its proximity to the BRD presents an opportunity for bivalent inhibition (Figure 1C). In this study we report a novel strategy to simultaneously target the TRIM24 PHD and BRD using bivalent peptide‐drug conjugates (PDCs), leveraging avidity to enhance PHD binding affinity. We detail the design of these PDC tools, including a screening approach to identify KMe_3_‐mimicking moieties that improve H3 peptide affinity for the TRIM24 PHD. We then describe the synthesis of the PDCs and their subsequent biophysical characterization, assessing affinities, selectivities, and binding modes.

## 2 Results and Discussion

### 2.1 TRIM24 PHD Ligand Design–H3K9Me_3_‐Mimicking Peptides

**Figure 2:**
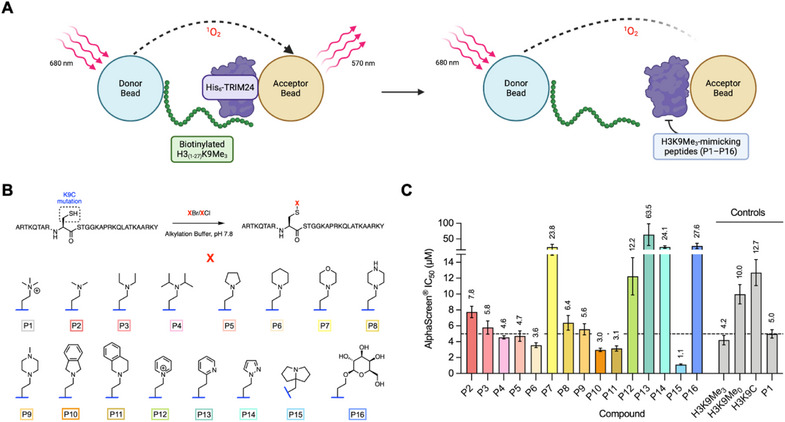
**A)**. Graphical representation of the AlphaScreen^®^ competition assay used to evaluate KMe_3_ bioisosteres. Biotinylated H3_(1–27)_K9Me_3_ and His_6_‐TRIM24 (PHD‐BRD) were attached to the donor and acceptor beads, respectively. Excitation of the donor bead produces singlet oxygen, which can diffuse to the acceptor bead when the beads are in close proximity (i.e., peptide bound to protein). Reaction of singlet oxygen at the acceptor bead surface produces a chemiluminescent emission signal. Singlet oxygen is readily quenched in solution, therefore, inhibition of biotinylated H3K9Me_3_‐TRIM24 binding causes a reduction in the emission signal.^[^ ^23^ ^]^ Figure created using BioRender.com. **B)**. Cysteine‐selective alkylation of a cysteine mutant (H3K9C) histone‐mimicking peptide and structures of the peptide modifications (X). *Reagents and conditions*: DTT, alkylation buffer, 37 °C, rt, 1 hour; then DTT, XBr/XCl, rt/50 °C, 5 hours; then 2‐mercaptoethanol, rt, 30 minutes. **C)**. AlphaScreen^®^ IC_50_ values of H3K9Me_3_‐mimicking peptides for inhibiting the interaction between biotinylated H3_(1–27)_K9Me_3_ and His_6_‐TRIM24 (PHD‐BRD). The dashed line represents the IC_50_ value of peptide **P1**–the sulfur containing analogue of H3K9Me_3_. Mean IC_50_ values (in µM) are shown above the bars. Error bars indicate the standard deviation of triplicate data.

To enhance the affinity of H3 peptides for the TRIM24 PHD, we decided to investigate KMe_3_‐bioisosteres that would bind effectively to W828 without necessarily being permanently charged. To screen for KMe_3_ mimics, we drew inspiration from previous studies that have used cysteine‐selective alkylation as a means to introduce KAc/KMe_3_ analogues into intact histones.^[^ ^24^, ^25^, ^26^ ^]^ Here, we used a similar approach to incorporate a small library of putative KMe_3_ mimics into H3‐mimicking peptides containing a cysteine mutation at position K9 (H3_(1–27)_K9C). The mimics (Figure 2B) were designed to probe a number of different interactions with W828 in the TRIM24 PHD, including cation‐π (**P1**–**P12** and **P15**), π‐π (**P10**, **P11**, **P13**, **P14**), and CH‐π (**P16**) interactions (Figure 2B).^[^ ^27^ ^]^ First, alkylating agents containing KMe_3_ mimics were synthesized (Supplementary Schemes S1–S7). Cysteine‐selective alkylation was then performed on the H3K9C peptide at pH 7.8 to deprotonate C9, while ensuring that potentially nucleophilic lysine residues remained protonated (Figure 2B). Reactions were monitored using LC‐MS (Supplementary Figure S4, Supplementary Table S1) and conditions such as reaction temperature and alkylating agent equivalents were optimized to minimize overalkylation. NanoLC‐MS/MS was used to confirm alkylation at residue C9 for two model peptides (**P1** and **P2**). However, we also observed alkylation on other residues, such as K23 and the N‐terminus, for peptide **P2** (Supplementary Table S2). After purification and MALDI‐TOF MS characterization (Supplementary Figure S5), the bioisostere‐containing peptides were evaluated in an AlphaScreen competition assay to determine IC_50_ values for displacement of biotinylated H3_(1–27)_K9Me_3_ from the TRIM24 PHD (Figure 2A, 2C and Supplementary Tables S3, S4).

The difference in IC_50_ values between unbiotinylated H3_(1–27)_K9Me_3_ (4.2 µM), H3_(1–27)_K9Me_0_ (10.0 µM), and H3_(1–27)_K9C (12.7 µM) (Figure 2C; Supplementary Table S4) demonstrated the contribution of the cation‐π interaction for binding to the PHD finger, which is in accordance with previous work.^[^ ^6^ ^]^ The solvent exposed nature of W828 is likely the reason that affinities of the methylated and unmethylated peptides are not more different. We observed a general trend of increasing affinity with increasing basicity of the amine nitrogen atom (Figure 2C and Supplementary Table S3). This trend was exemplified by piperidine‐containing peptide **P6** (3.56 µM), which exhibited stronger binding than morpholine‐containing peptide **P7** (23.8 µM), likely due to the electron withdrawing effects from the oxygen atom (Figure 2C and Supplementary Table S3). Peptides containing KMe_3_ mimics that are uncharged at physiological pH (**P13**, **P14**, and **P16**) showed low TRIM24 affinities (Figure 2C), indicating that π‐π and CH‐π interactions alone are insufficient to compensate for loss of the cation‐π interaction with W828. Amine basicity and cation–π interactions are not the only factors influencing affinity for the TRIM24 PHD. For example, peptides **P10** and **P11** demonstrated increased affinity compared to peptides **P2**–**P6**, despite having lower basicities (Figure 2C and Supplementary Table S3). This was likely due to additional π‐π contacts and lipophilic interactions with W828. **P15** showed the most significant increase in affinity, with an approximate fourfold boost relative to **P1** (Figure 2C). The pyrrolizidine moiety in **P15** contains a lipophilic amine with the highest basicity among the mimics and contains a highly conformationally‐constrained nitrogen atom, all of which likely contribute to the high affinity. Although pyrrolizidine (**P15**) appeared the optimal KMe_3_ mimic to use in our PDCs, it proved difficult to incorporate into an unnatural amino acid, which is required to be included in the H3‐mimicking peptide. Therefore, instead we chose to advance the isoindoline, seen in **P10**, as a more synthetically tractable alternative, which still exhibited higher binding affinity than KMe_3_.

### 2.2 Synthesis of PDCs 4‐–6

**Figure 3:**
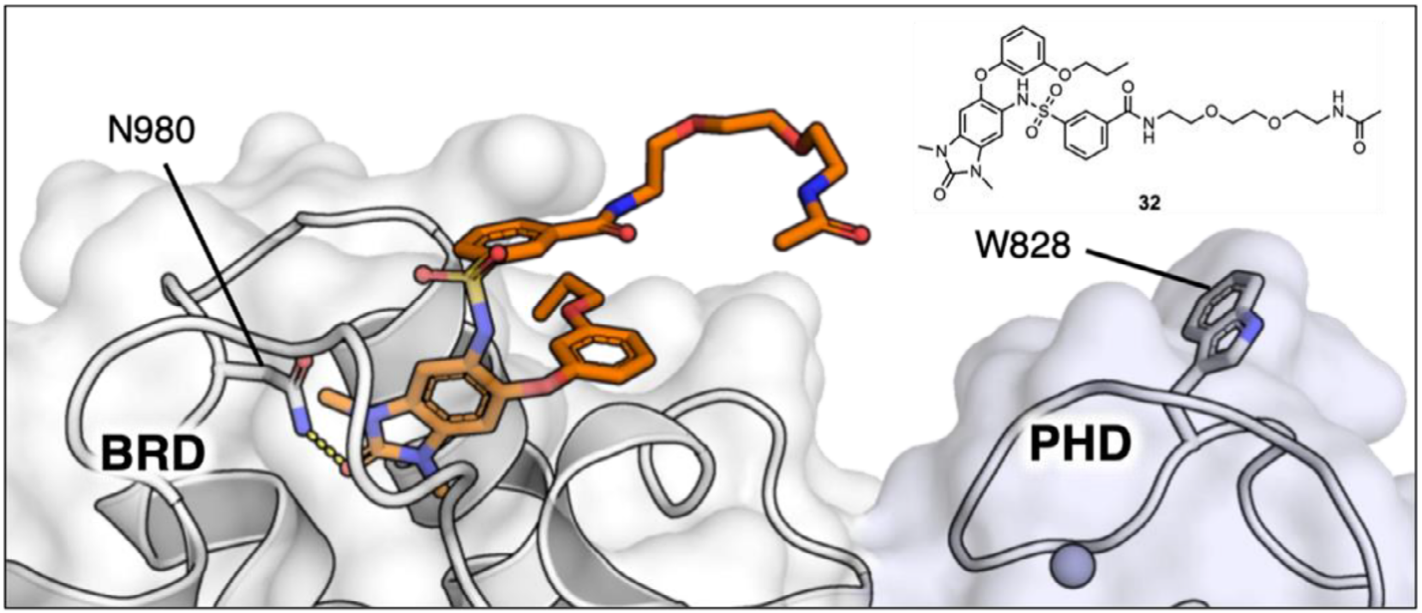
A) crystal structure (1.5 Å, PDB ID: 9GDG) of TRIM24 (PHD‐BRD) in complex with TRIM24 BRD ligand **32**, derived from the dTRIM24 PROTAC.^[^ ^21^ ^]^ The structure shows an exit vector from the benzenesulfonamide ring, that points toward W828 in the TRIM24 PHD. The TRIM24 PHD and BRD are shaded in light blue and white, respectively. Grey spheres represent Zn^2+^ ions.

Having identified a suitable KMe_3_ mimic for enhancing the affinity of H3‐mimicking peptides for the TRIM24 PHD, we next began the synthesis of PDCs 4–6 (Scheme 2). The BRD‐binding component of the PDCs is based on compound **28**, a component of the TRIM24 PROTAC (Supplementary Figure S1) developed by Bradner et al., as it contains a suitable handle for linker attachment.^[^ ^21^ ^]^ BRD ligand **28** was synthesized using a 6‐step synthesis, adapted from a patent procedure, in 30% overall yield (Supplementary Scheme S8).^[^ ^28^ ^]^ Crystallization of compound **32** in complex with the TRIM24 PHD‐BRD confirmed that the PEG linker is solvent‐exposed and a suitable vector for the attachment of H3‐mimicking peptides (Figure 3 and Supplementary Figure S6). Main text paragraph.

**Scheme 1:**
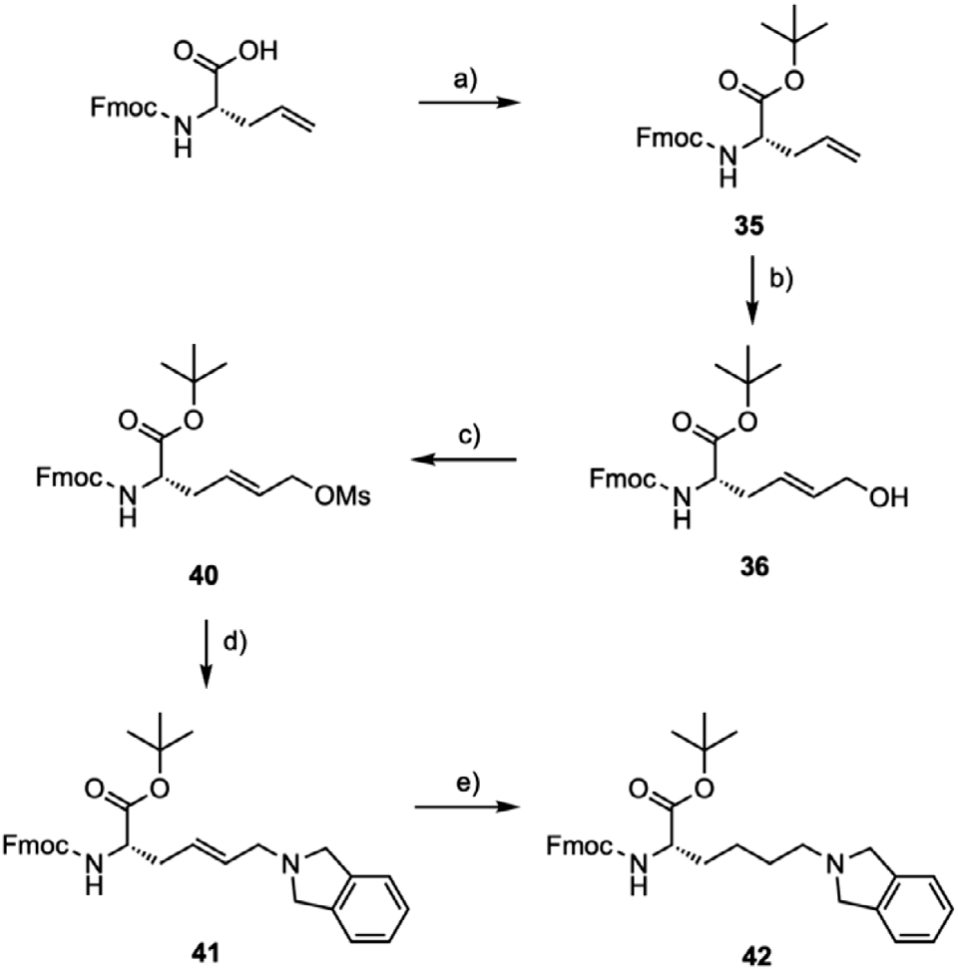
Synthesis of unnatural amino acid **42**. *Reagents and conditions*: a) *tert*‐Butyl‐2,2,2‐trichloroacetimidate, BF_3_•OEt_2_, CH_2_Cl_2_, cyclohexane, rt, 6 hours (69%); b) *cis*‐Butene‐1,4‐diol, Grubbs’ II (cat., 5 mol%), CH_2_Cl_2_, reflux, 40 °C, 6 hours (70%); c) MsCl, NEt_3_, CH_2_Cl_2_, 0 °C–rt, 1 hour (83%); d) Isoindoline•HCl, K_2_CO_3_, KI, DMF, 0 °C–rt, 1 hour (71%); e) PtO_2_ (10 mol%), H_2(g)_ 1 atm, EtOAc, rt, 6 hours (83%).

For the PHD‐binding moiety of the PDCs, the isoindoline KMe_3_ mimic (from **P10**, Figure 2B) was incorporated into an unnatural amino acid (**42**) (Scheme 1). Although side chain sulfur oxidation was not observed for our histone‐mimicking peptides, we envisaged it could still pose stability issues for the PDCs in the future; therefore, a cysteine alkylation strategy was avoided. Instead, Fmoc l‐allyl glycine, protected as the *^t^* Bu ester, was reacted with *cis*‐butene‐1,4‐diol in a Grubbs’ cross metathesis reaction to afford allylic alcohol **36** as the *E*‐isomer in 70% yield (Scheme 1). We next sought to activate the alcohol to nucleophilic substitution with isoindoline. However, attempts to convert alcohol **36** to an alkyl bromide using Appel conditions unexpectedly caused cyclisation to form compound **39** (Supplementary Scheme S9). We postulated this likely occurred via successive S_N_2’ mechanisms, involving cyclisation of the amide nitrogen onto the double bond to form the six‐membered ring (Supplementary Figure S7). To prevent cyclisation, it was imperative to avoid isomerization of the double bond through the first S_N_2’ step, and so we decided to convert **36** to mesylate **40** instead, which was achieved in 83% yield (Scheme 1). Mesylate **40** was subsequently reacted with isoindoline to afford compound **41** in 71% yield (Scheme 1). Finally, reduction of the double bond using a PtO_2_ catalyst in the presence of H_2_ gas gave the desired amino acid **42** in 24% total yield over 5 steps (Scheme 1).

**Scheme 2:**
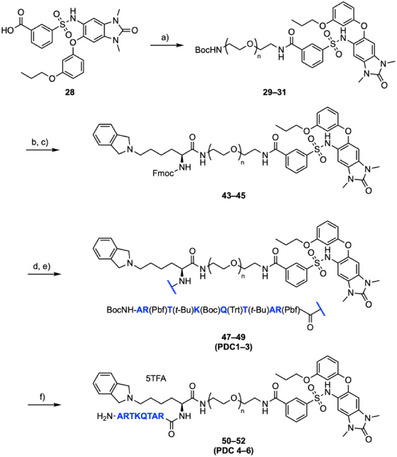
Synthesis of PDC4–6 (**50**–**52**). *Reagents and conditions*: a) BocNH(CH_2_CH_2_O)_n_CH_2_CH_2_NH_2_, HATU, DIPEA, DMF, rt, 24 hours (**29**: n = 2, 66%; **30**: n = 3, 80%; **31**: n = 4, 88%); b) Amino acid **42**, 4 M HCl in dioxane, rt, 24 hours; c) HATU, DIPEA, DMF, rt, 24 hours (**43**: n = 2, 64%; **44**: n = 3, 49%; **45**: n = 4, 77%); d) 20% *v/v* Piperidine, DMF, rt, 1 hour; e) Peptide **46**, HATU, DIPEA, CH_2_Cl_2_, rt, 24 hours (**47**: n = 2, 52%; **48**: n = 3, 57%; **49**: n = 4, 42%); f) TFA/TIPS/H_2_O (38:1:1), rt, 4 hours (**50**: n = 2, 32%; **51**: n = 3, 51%; **52**: n = 4, 50%).

To assemble the PDCs, Boc‐protected amino functionalized PEG linkers were attached to BRD ligand **28**, to afford compounds **29**–**31** in 66–88% yield (Scheme 2). Removal of the Boc group enabled the attachment of amino acid **42** to the PEG amines, using HATU‐mediated coupling conditions, to yield compounds **43**–**45** in 49–77% yield (Scheme 2). The protected H3_(1‑8)_ peptide **46** was synthesized using Fmoc solid phase peptide synthesis (SPPS) on 2‐chlorotrityl (2‐ClTrt) resin with a coupling mixture of DIC and Oxyma Pure (Supplementary Scheme S3). Resin cleavage was achieved, without removal of the sidechain protecting groups, using hexafluoroisopropanol (HFIP) to afford protected peptide **46** (Supplementary Scheme S10). Peptide **46** was subsequently coupled onto the N‐terminus of compounds **43**–**45** (Scheme 2), and a global TFA deprotection strategy (Scheme 2) was used to remove the sidechain protecting groups affording PDCs 4–6 (**50**–**52**) in 32–51% yield. Monovalent control compounds, designed to bind to either the PHD or BRD (but not both), were also synthesized for use in investigating the binding mode of the PDCs (Supplementary Schemes S11, S12).

### 2.3 Evaluation of Binding Affinities and Kinetics

**Figure 4:**
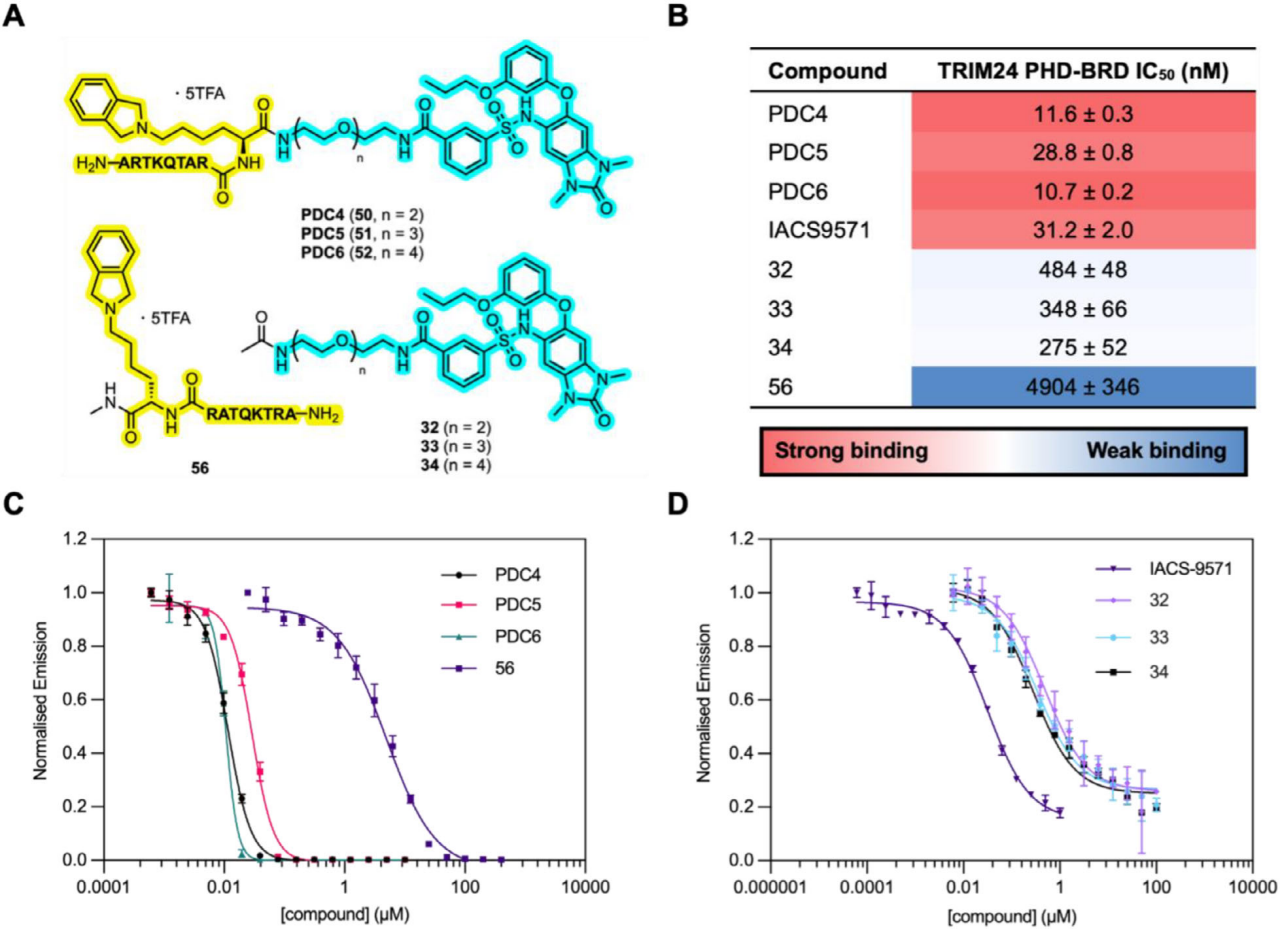
**A)**. Structures of PDCs 4–6 (**50**–**52**), TRIM24 BRD ligands (**32**–**34**), and the TRIM24 PHD‐binding peptide (**56**). The PHD‐ and BRD‐binding sections of the PDCs are shown in yellow and cyan, respectively. **B)**. AlphaScreen^®^ competition assay IC_50_ values, in nM, for the inhibition of the interaction between His_6_‐TRIM24 and biotinylated H3_(1–27)_K9Me_3_K18Ac (i.e., binding to both the PHD and BRD). IC_50_ values are quoted as the mean of triplicate data ± standard error of the mean. Hot colors indicate high affinity, and cool colors indicate lower affinity, for the TRIM24 PHD‐BRD cassette. **C). and D)**. AlphaScreen^®^ competition assay dose‐response curves. For normalization, emission was divided by the lowest compound concentration emission signal in each data set. Error bars indicate the standard deviation of triplicate data.

Next, we investigated whether PDCs 4–6 (Figure 4A) could simultaneously bind to the TRIM24 PHD and BRD. For this we used another AlphaScreen^®^ competition assay to determine if the PDCs could displace a selection of histone‐mimicking competitor peptides (H3K9Me_3_, H3K18Ac, and H3K9Me_3_K18Ac) from the TRIM24 PHD‐BRD cassette. Competition with the H3K9Me_3_ peptide reports on the ability of a compound to bind to the TRIM24 PHD, competition with the H3K18Ac peptide reports on the ability of a compound to bind to the TRIM24 BRD, and competition with the H3K9Me_3_K18Ac peptide reports on the ability of the compound to bind to both the TRIM24 PHD and BRD. Unbiotinylated analogues of the competitor peptides were included as positive controls, and H3_(1–15)_K4Me_3_ was used as a technical negative control, as previous work has shown that K4 methylation prevents binding to TRIM24 (Supplementary Tables S5–S7).^[^ ^4^, ^29^ ^]^ A TruHits assay was also performed to preclude assay interference (Supplementary Figure S8).

Pleasingly, PDCs 4–6 exhibited low nanomolar IC_50_ values when displacing PHD‐ and BRD‐binding histone‐mimicking peptides (Figure 4B, 4C, and 4D; Supplementary Tables S5–S7), indicating their ability to engage both domains. The data suggest the PDCs have higher affinity for TRIM24 than the current best‐in‐class BRD inhibitor, IACS9571 (Figure 4B, Supplementary Figures S5–S7). Furthermore, the PDCs inhibited the binding of the competitor peptides more effectively than the parent PHD‐binding (compound **56**) or BRD‐binding (compounds **32**–**34**) ligands (Figure 4B, 4C, and 4D). For instance, PDC4 (compound **50**) demonstrated 422‐fold and 42‐fold lower IC_50_ values (Figure 4B) for displacing H3K9Me_3_K18Ac, relative to its corresponding parent PHD (compound **56**) and BRD ligands (compound **32**), respectively. Intriguingly, in the assay involving H3K9Me_3_K18Ac, the AlphaScreen^®^ signal plateaued significantly above baseline for IACS9571 and parent BRD ligands **32**–**34** (Figure 4D).

The dual‐modified H3_(1–27)_K9Me_3_K18Ac peptide has been identified as one of the highest affinity histone‐mimicking peptides for the TRIM24 PHD and BRD, with a *K*_d_ = 1.94 µM, measured using ITC.^[^ ^6^ ^]^ It was suggested that this strong binding arises from avidity effects due to simultaneous interactions with both the PHD and BRD.^[^ ^29^ ^]^ Consequently, only compounds capable of inhibiting *both* the PHD and BRD can fully displace the H3_(1–27)_K9Me_3_K18Ac competitor peptide. While IACS9571 and BRD ligands **32**–**34** can block K18Ac binding to the BRD, the competitor peptide can still engage the PHD, albeit with lower affinity, via its H3_(1–9)_K9Me_3_ motif. However, the previously described data indicate the PDCs bind to both domains, thereby completely inhibiting binding of H3_(1–27)_K9Me_3_K18Ac. This could explain why the PDCs reduce the AlphaScreen signal to baseline, in contrast to the residual signal observed for IACS9571 and parent BRD ligands **32**–**34** (Figure 4D). Interestingly, the parent PHD ligand **56** also reduces the signal to baseline, perhaps indicating that it can weakly bind to the BRD and fully displace the competitor peptide by two molecules of **56** engaging with the PHD and BRD, respectively.

**Figure 5:**
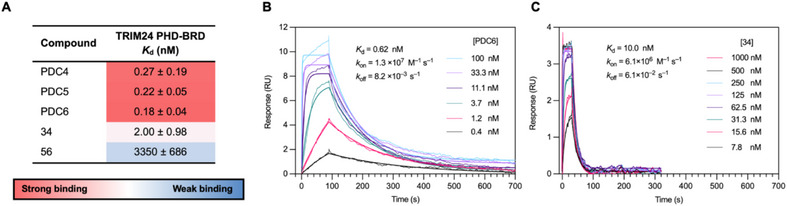
**A)**. BROMO*scan K*_d_ values for binding to TRIM24 (PHD‐BRD). *K*_d_ values are quoted as the mean of duplicate data ± 95% confidence interval. **B)**. SPR sensograms overlaid with the fitted association and dissociation curves for the binding of PDC6 (**52**) to TRIM24. *K*_d_ values and rate constants are quoted as the mean of means for triplicate data. **C)**. SPR sensograms overlaid with the fitted association and dissociation curves for the binding of BRD ligand **34** to TRIM24. *K*_d_ values and rate constants are quoted as the mean of means for triplicate data. Raw SPR data with error values is included in the Supporting Information (Supplementary Table S9).

Having shown that the PDCs can simultaneously inhibit both the PHD and BRD, we next wanted to probe the binding affinities and kinetics in more detail. The BROMO*scan* assay was used to obtain *K*_d_ values for binding to the TRIM24 PHD‑BRD. The PDCs exhibited remarkably high, picomolar, affinities for TRIM24 in this assay, which indicate substantially stronger binding than parent ligands **34** and **56** ‐ 11‐fold and > 18000‐fold, respectively (Figure 5A). We then employed surface plasmon resonance (SPR) to probe the binding kinetics of the PDCs, with H3_(1‑27)_K9Me_3_K18Ac serving as the positive control (Supplementary Figure S10). In accordance with the BROMO*scan* data, PDC6 again demonstrated picomolar binding (*K*_d_ = 0.6 ± 0.2 µM), a 17‐fold increase in affinity compared to its parent BRD ligand **34** (Figure 5B, 5C; Supplementary Table S9). It became evident from the sensograms (Figure 5B, 5C) that the high affinity of the PDCs results primarily from a slow *k*_off_. This was supported by the calculated values from the fitted curves which revealed a sevenfold slower *k*_off_ for PDC6 compared to the parent BRD ligand **34** (Figure 5B, 5C and Supplementary Table S9). These data are consistent with the PDCs binding simultaneously to the PHD and BRD.

### 2.4 Evaluation of Binding Mode

**Figure 6:**
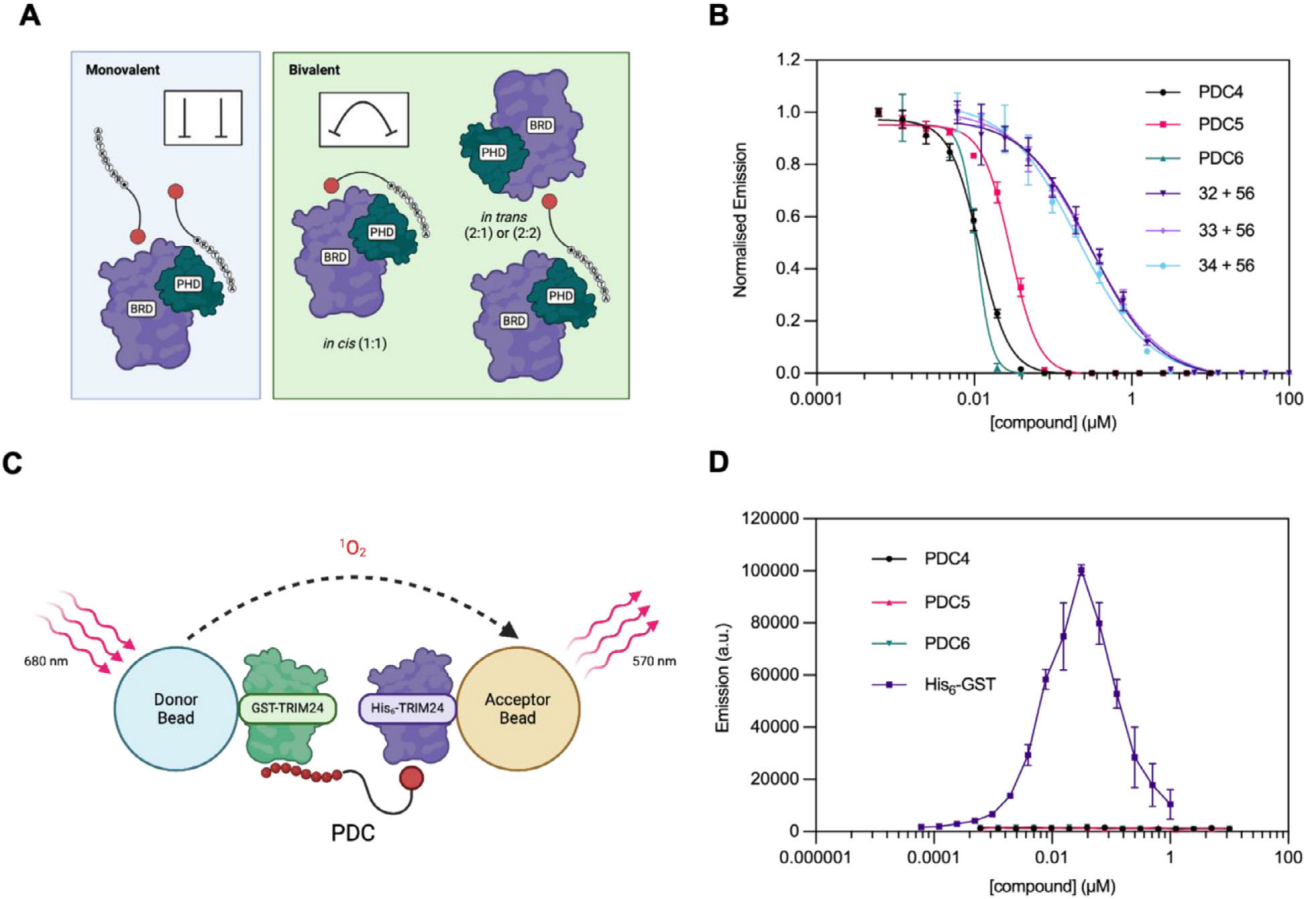
**A)**. Schematic representation of the possible binding modes of the PDCs to the TRIM24 PHD and BRD. The red circle represents the TRIM24 BRD ligand and white circles with single letter amino acid codes represent the PHD‐binding peptide. A “star” indicates the unnatural KMe_3_‐mimicking amino acid. Figure created using BioRender.com. **B)**. AlphaScreen^®^ competition assay dose‐response curves for the inhibition of the interaction between His_6_‐TRIM24 (PHD‐BRD) and biotinylated H3_(1–27)_K9Me_3_K18Ac. For compound mixtures, both compounds were used at the same concentration. For normalization, emission was divided by the lowest compound concentration emission signal in each data set. Mean values of triplicate data are plotted and error bars indicate the standard deviation of triplicate data. **C)**. Graphical representation of the AlphaScreen^®^ ternary complex detection assay. GST‐TRIM24 and His_6_‐TRIM24 were attached to glutathione donor and Ni^2+^ chelate acceptor beads, respectively. A ternary complex is formed if the PDCs bind *in trans* to the PHD of one TRIM24 protein and the BRD of another. This brings the donor and acceptor beads into proximity resulting in an AlphaScreen^®^ emission signal. In the absence of a ternary complex no signal will be produced. Figure created using BioRender.com. **D)**. AlphaScreen^®^ ternary complex detection assay dose‐response curves. His_6_‐GST has been included as a technical positive control without TRIM24 to illustrate the expected result if a ternary complex is formed. Mean values of triplicate data are plotted and error bars indicate the standard deviation of triplicate data.

Due to their bifunctional nature, there are a number of possible modes that could be adopted by the PDCs when binding to TRIM24 (Figure 6A). To determine which of these was being observed, we first employed an AlphaScreen^®^ assay to differentiate between monovalent and bivalent binding. In this assay, we compared the PDCs to mixtures of their unlinked monovalent parent PHD and BRD ligands (e.g., PDC4 versus compounds **32** + **56**) by measuring their ability to displace biotinylated H3_(1–27)_K9Me_3_K18Ac from the TRIM24 PHD and BRD. The PDCs 4–6 demonstrated substantially lower IC_50_ values than mixtures of the corresponding unlinked parent ligands (Figure 6B and Supplementary Table S7), consistent with a bivalent mode of binding to the TRIM24 BRD‐PHD. PDC4, for example, displayed a 26‐fold lower IC_50_ than the mixture containing compounds **32** and **56**. These results strongly suggest that the PDCs bind simultaneously to the PHD and BRD in a bivalent manner. Additionally, the data indicate that shorter PEG linkers enhance bivalent binding, as PDC4 – the shortest PEG chain tested–showed the greatest reduction in IC_50_ compared to its unlinked parent ligands (**32** + **56**).

Given the growing evidence for bivalent binding, we sought to determine whether this was occurring through an *in cis* (to the same copy of the protein) or *in trans* (to two copies of the protein) binding mode. To investigate this, we used an AlphaScreen proximity induction assay, developed in the Conway group,^[^ ^30^ ^]^ to assess the ability of the PDCs to form a ternary complex (Figure 6C). In this assay an emission signal is only generated If the PDCs bind *in trans*, meaning they interact with the PHD of one protein and the BRD of another. As a technical positive control, His_6_‐GST was tested without any TRIM24 protein added to confirm the characteristic bell‐shaped curve of ternary complex formation, illustrating a hook effect (Figure 6D).^[^ ^31^ ^]^ In contrast, PDCs 4–6 failed to produce a similar emission signal, indicating that no ternary complex was formed (Figure 6D). These results suggest that the PDCs are binding to the TRIM24 PHD and BRD via an *in cis* binding mode.

### 2.5 Evaluation of TRIM24 Selectivity

**Figure 7:**
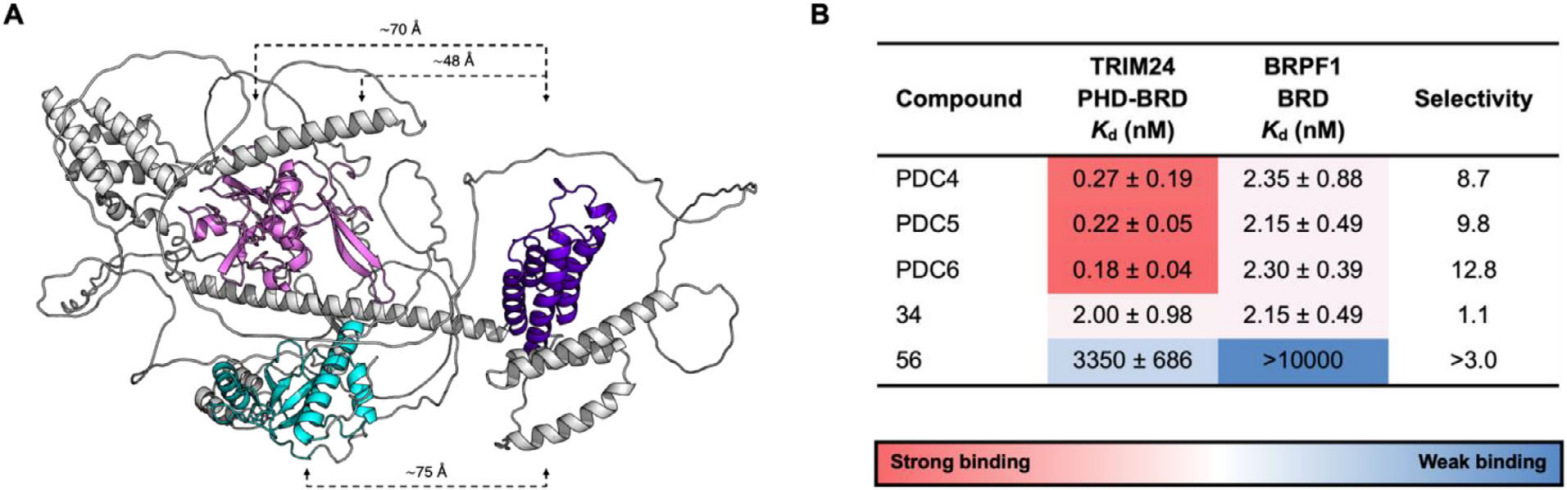
**A)**. AlphaFold structure (AF‐P55201‐F1‐v4) of full length BRPF1 showing the predicted positions of the PZP domain (Pink), the PWWP domain (cyan), and the BRD (purple). Approximate distances between the BRD and PWWP domain, and each PHD of the PZP domain were estimated using the AlphaFold model in PyMOL, and are shown (not to scale). **B)**. BROMO*scan K*_d_ values and selectivities for binding to TRIM24 (PHD‐BRD) and BRPF1 (BRD) [Selectivity =  *K*_d, BRPF1_/*K*_d, TRIM24_]. *K*_d_ values are quoted as the mean of duplicate data ± 95% confidence interval.

Despite the discovery of highly selective BRPF1 BRD inhibitors by Bamborough et al., achieving selectivity over BRPF1 still remains a significant challenge for TRIM24 BRD inhibitors.^[^ ^32^ ^]^ For example, the TRIM24 inhibitor IACS9571, exhibits higher affinity for BRPF1 than TRIM24 (14 nM and 31 nM, respectively).^[^ ^13^ ^]^ In addition to its BRD, BRPF1 contains a PWWP domain that recognizes H3K36Me_3_, and a PZP domain (containing two PHDs separated by a zinc knuckle) that bivalently binds DNA and unmodified H3.^[^ ^33^, ^34^ ^]^ An AlphaFold model of BRPF1 predicts that the PWWP and PZP domains are positioned further from the BRD than the PHD is in TRIM24 (Figure 7A and Supplementary Figure S9). This spatial arrangement suggests that simultaneous *in cis* binding of the PDCs developed here to the BRD and PZP/PWWP domains is unlikely to be possible. Therefore, this potentially provides a route to gaining selectivity over BRPF1 through avidity using bivalent PDCs.

We evaluated the affinity of PDCs 4–6 for the BRD of BRPF1 using a BROMO*scan* assay (an assay for the BRD and PHDs of BRPF1 does not exist). The parent PHD ligand (compound **56**) showed no detectable binding to the BRPF1 BRD and, as expected, the BRD ligand **34** showed similar affinity for the BRDs of both TRIM24 and BRPF1 (Figure 7B and Supplementary Table S8). In contrast, the PDCs demonstrated around 10‐fold selectivity for TRIM24 (Figure 7B). These findings suggest that *in cis* binding of PDCs to the PHD and BRD in TRIM24 could enhance selectivity over BRPF1. A full‐length BRPF1 construct was not available for evaluation in the BROMOscan assay; however, future studies could explore whether the PDCs maintain selectivity when binding to the full‐length protein. While we have not assessed the selectivity of the PDCs over the other BRD‐ and PHD‐ containing members of the TRIM proteins, TRIM28, TRIM33, and TRIM66, we would expect the PDCs to bind preferentially to TRIM24. It has previously been shown that the parent TRIM24 ligand, IACS9571, does not bind to TRIM33α or β.^[^ ^6^, ^13^ ^]^ TRIM28 possesses a “noncanonical” BRD that has a Thr rather than the conserved KAc‐binding Asn residue in the ligand binding pocket, and so will likely not bind to IACS9571 either. Both TRIM28 and TRIM66 have different residues at the equivalent position of W828, Thr in TRIM28 and Phe in TRIM66, and so neither are likely to form an effective cation‐π interaction with the PDCs, conferring selectivity for TRIM24.^[^ ^6^ ^]^

### 2.6 Evaluation of Phenotypic Effects

Since TRIM24 overexpression is known to upregulate oestrogen‐dependent genes involved in cell proliferation and breast cancer development, we sought to evaluate the phenotypic effects of the PDCs in the ER^+^ MCF‐7 cell line.^[^ ^4^ ^]^ To provide a comparison in the absence of ER expression, we also included the triple‐negative CAL‐51 cell line. Before assessing phenotypic effects, we first examined the cell permeability of the PDCs in MCF‐7 cells. Interestingly despite, its peptidic nature, PDC5 demonstrated a degree of cell permeability in a previously reported LC‐MS assay (Supplementary Figures S11–S19).^[^ ^35^, ^36^ ^]^ In contrast, PDCs 4 and 6, as well as PHD peptide **56**, exhibited no detectable cell permeability. We suggest that the cationic residues in the peptide might contribute to the permeability of PDC5, and the lower permeability of PDC4 and 6 might result from the difference in PEG linker lengths, although further studies would be needed to confirm this. Given these data, PDC4 and 6 were excluded from further evaluation. Consistent with other TRIM24 BRD inhibitors (e.g., IACS9571), PDC5, and its parent BRD ligand **33** did not show cytotoxic effects on ER^+^ or triple‐negative breast cancer cell lines at concentrations up to 10 μ M (Supplementary Figure S20).^[^ ^13^, ^15^ ^]^ Similarly, in proliferation assays, neither compound significantly affected breast cancer cell growth (Supplementary Figures S21–S25). Further optimization of these high‐affinity PDC tools will enable a more detailed investigation of the function of TRIM24 and the role of its PHD and BRD in breast cancer.

We have demonstrated that PDCs can enhance PHD domain affinity by leveraging bivalent binding with a neighboring BRD. Despite their high prevalence in the human proteome, PHDs remain an underexplored class of epigenetic readers due to their predicted low ligandability.^[^ ^9^ ^]^ Many PHD fingers are, however, part of larger tandem reader cassettes or are closely associated with more ligandable reader domains such as BRDs, Tudor domains, and chromodomains.^[^ ^9^, ^37^, ^38^ ^]^ For instance in 12 PHD‐containing proteins, the PHD is located within 30 amino acids of an adjacent BRD (e.g., TRIM28, TRIM33, and TRIM66).^[^ ^12^, ^37^, ^39^, ^40^ ^]^ Beyond the TRIM protein family, other closely linked histone‐interacting PHD‐BRD pairs exist, including those in BPTF, BAZ2A/B, and CREBBP/P300.^[^ ^41^, ^42^, ^43^ ^]^ Given the challenges of liganding PHDs in isolation, a bivalent PDC strategy, similar to the one used here, could be explored to improve affinity for other PHDs through avidity. This approach could help with deconvoluting the biological consequences of PHD‐mediated epigenetic regulation and provide deeper insights into the roles of this domain in disease.

## 3 Conclusion

In this study, we have developed high affinity PDC tools targeting the TRIM24 PHD and BRD. To achieve this, we screened a series of H3‐mimicking peptides containing KMe_3_ isosteres, to identify modifications that enhance TRIM24 PHD binding while inhibiting the cation‐ π interaction between H3K9Me_3_ and W828. Among these, isoindoline emerged as one of the most promising KMe_3_ mimics and was incorporated into a series of PDCs, each consisting of a histone‐mimicking peptide, linked to a small molecule TRIM24 BRD ligand. We comprehensively evaluated the affinity, selectivity, and binding mode of these PDCs using AlphaScreen^®^, BROMO*scan*, and SPR. These assays demonstrated that the PDCs simultaneously inhibit the TRIM24 PHD and BRD with exceptionally high affinity, which we showed is driven by a slow *k*_off_ and an *in cis* bivalent binding mode. Although the PDCs elicited modest effects on breast cancer cell proliferation in vitro, our findings highlight their potential for inhibiting tandem epigenetic reader domains to enhance affinity and target selectivity. Beyond their application to TRIM proteins, PDCs could serve as valuable tools for studying PHD domains and advancing our understanding of the biological consequences of epigenetic regulation.

## Supporting Information

The authors have cited additional references separately within the SI.^[^ ^45^, ^46^, ^47^, ^48^, ^49^, ^50^, ^51^, ^52^, ^53^, ^54^, ^55^, ^56^, ^57^, ^58^, ^59^, ^60^, ^61^, ^62^, ^63^, ^64^, ^65^, ^66^, ^67^, ^68^, ^69^, ^70^, ^71^, ^72^, ^73^, ^74^, ^75^, ^76^, ^77^, ^78^, ^79^, ^80^, ^81^, ^82^, ^83^, ^84^, ^85^, ^86^, ^87^ ^]^

## Conflict of Interest

There are no conflicts to declare.

## Supporting information



Supporting Information

## Data Availability

The data that support the findings of this study are available in the supplementary material of this article.
