# Supplementary material for: High‐Affinity Peptide‐Drug Conjugate Ligands for the TRIM24 PHD and Bromodomain
Source: Chemistry. 2025 Nov 17;31(72):e03011. doi: 10.1002/chem.202503011 (PMC12731528; doi:10.1002/chem.202503011)
Supplement: Supplementary file 1 — Supporting Information [file CHEM-31-e03011-s001.pdf]

Supporting Information (SI)

High-Affinity Peptide-Drug Conjugate Ligands for the TRIM24 PHD and Bromodomain

Michael A. Platt,[1] Ekaterina Kot,[2] Louise A. W. Martin,[3] Antoine L. D. Wallabrègue,[1] Liwen Song,[1] Alistair M. Boyd,[1] Lizbé Koekemoer,[2] Ester M. Hammond,[3] Stuart J. Conway[1,4]*

Institutions:

[1]Department of Chemistry, Chemistry Research Laboratory, University of Oxford, Mansfield Road, Oxford, OX1 3TA, U.K.

[2]Centre for Medicines Discovery, Nuffield Department of Medicine Research Building, University of Oxford, Roosevelt Drive, Oxford, OX3 7FZ

[3]Department of Oncology, Old Road Campus Research Building, University of Oxford, Roosevelt Drive, Oxford, OX3 7DQ, U.K.

[4]Department of Chemistry and Biochemistry, University of California Los Angeles, 607 Charles E. Young Drive East, Los Angeles, California, 90095, U.S.A.

*To whom correspondence should be addressed.

Table of Contents

1 Supplementary Figures 4
2 Supplementary Tables 56
3 Supplementary Schemes 67
4 Biological and Biochemical Methods 74
5 Chemistry Experimental Section 90
6 NMR Spectra of Novel Compounds 129
7 LC-MS Chromatograms for **50**–**52** and **56** 167
8 HPLC Chromatograms for **P1**–**P16**, **28**, **32**–**34**, **50**–**52** and **56** 171
SI References 183

1 Supplementary Figures

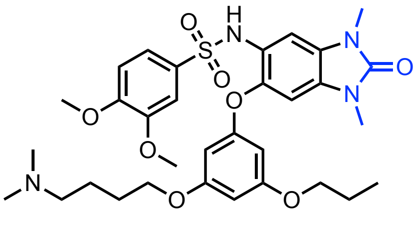

IACS9571

TRIM24 K_d 31 nM

BRPF1 K_d 14 nM

(Palmer *et al.* 2015)

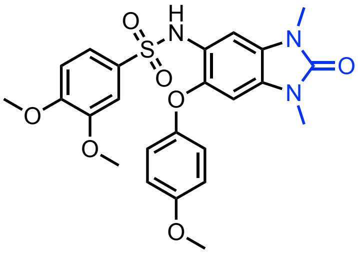

TRIM24 K_d 0.22 µM

BRPF1 K_d 0.14 µM

(Bennett *et al.* 2015)

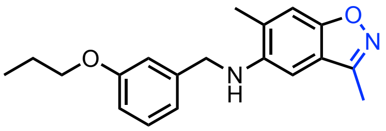

TRIM24 IC_{50} 1.88 µM

BRPF1 IC_{50} –

(Hu *et al.* 2020)

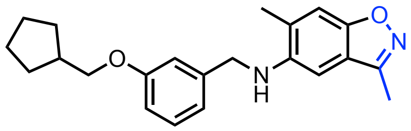

TRIM24 IC_{50} 2.53 µM

BRPF1 IC_{50} –

(Hu *et al.* 2020)

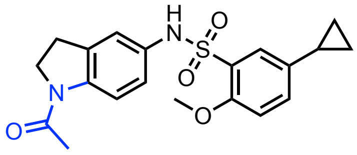

TRIM24 IC_{50} 0.98 µM

BRPF1 IC_{50} 1.16 µM

(Xiang *et al.* 2022)

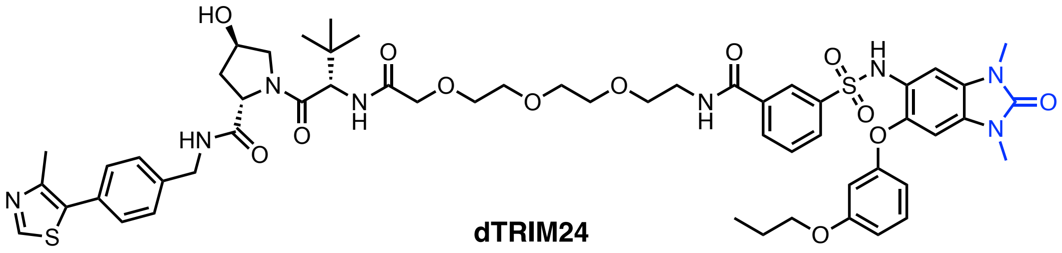

dTRIM24

TRIM24 K_d 42 nM

BRPF1 K_d 52 nM

(Gechijian *et al.* 2018)

Figure S1: Structures of the reported TRIM24 BRD ligands and the dTRIM24 PROTAC.[1–5] The presumed KAc-mimicking moieties are shown in blue. A dash (–) indicates that the affinity was not reported.

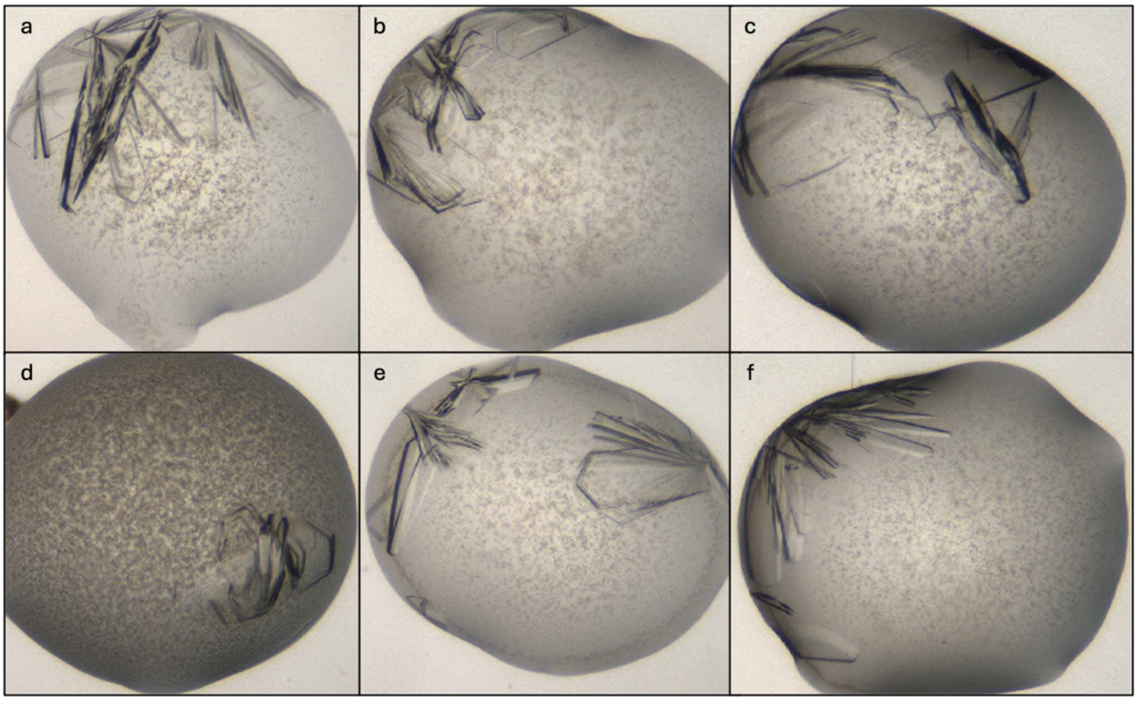

Figure S2: A selection of *apo* untagged TRIM24 (PHD-BRD) crystals obtained in the fine screen. *Conditions:* a) 1.8 M ammonium sulfate, 0.1 M HEPES (pH 7.4), PEG-400 5 % *v*/*v*, 2:1; b) 1.8 M ammonium sulfate, 0.1 M HEPES (pH 7.4), PEG-400 5 % *v*/*v*, 1:1; c) 2.0 M ammonium sulfate, 0.1 M HEPES (pH 7.7), PEG-400 2% *v*/*v*, glycerol 10% *v*/*v*, 1:1; d) 2.0 M ammonium sulfate, 0.1 M HEPES (pH 7.9), PEG-400 2% *v*/*v*, glycerol 8% *v*/*v*, 1:2; e) 1.8 M ammonium sulfate, 0.1 M HEPES (pH 7.7), PEG-400 2% *v*/*v*, 1:1; f) 1.8 M ammonium sulfate, 0.1 M HEPES (pH 7.1), PEG-400 5% *v*/*v*, 1:2. Ratios are given as protein : reservoir. Reservoir solutions are aqueous.

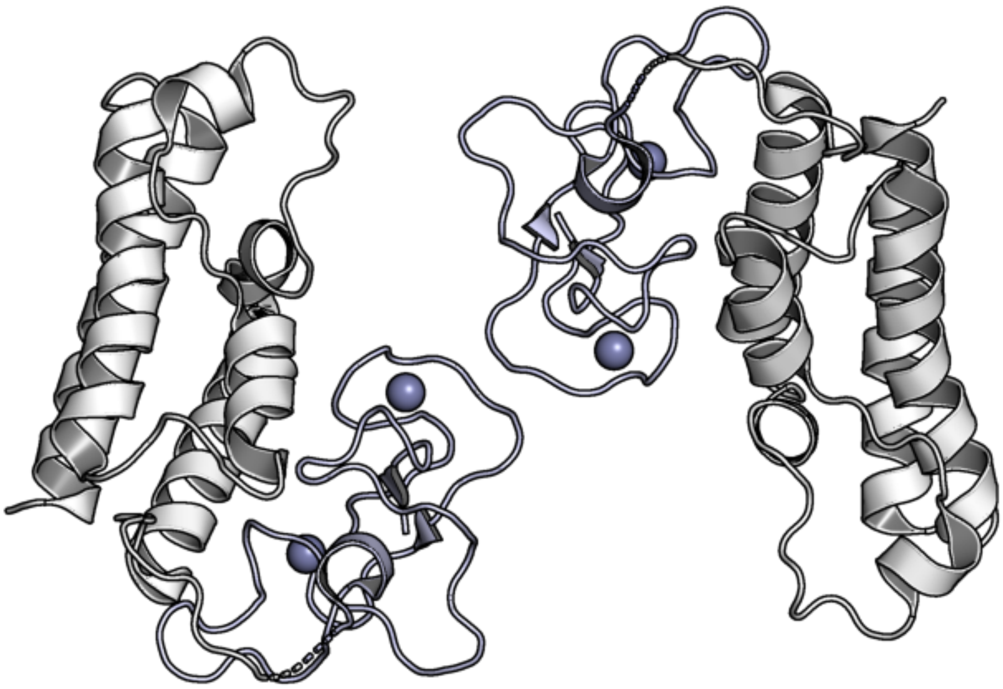

Figure S3: Unit cell for the new *apo* crystal structure of TRIM24 in the C121 space group (1.7 Å, PDB ID: 9GD5). The PHD and BRD are coloured in light blue and white, respectively. Grey spheres represent Zn^{2+} ions. Dashed lines represent unresolved residues in disordered regions.

Figure S4: ESI$^+$ and deconvoluted mass spectra for histone-mimicking peptides **P1** and **P2** before purification. Structures of the peptides are shown with single letter amino acid codes. The modification is shown in blue. Theoretical molecular weights were calculated in ChemDraw.

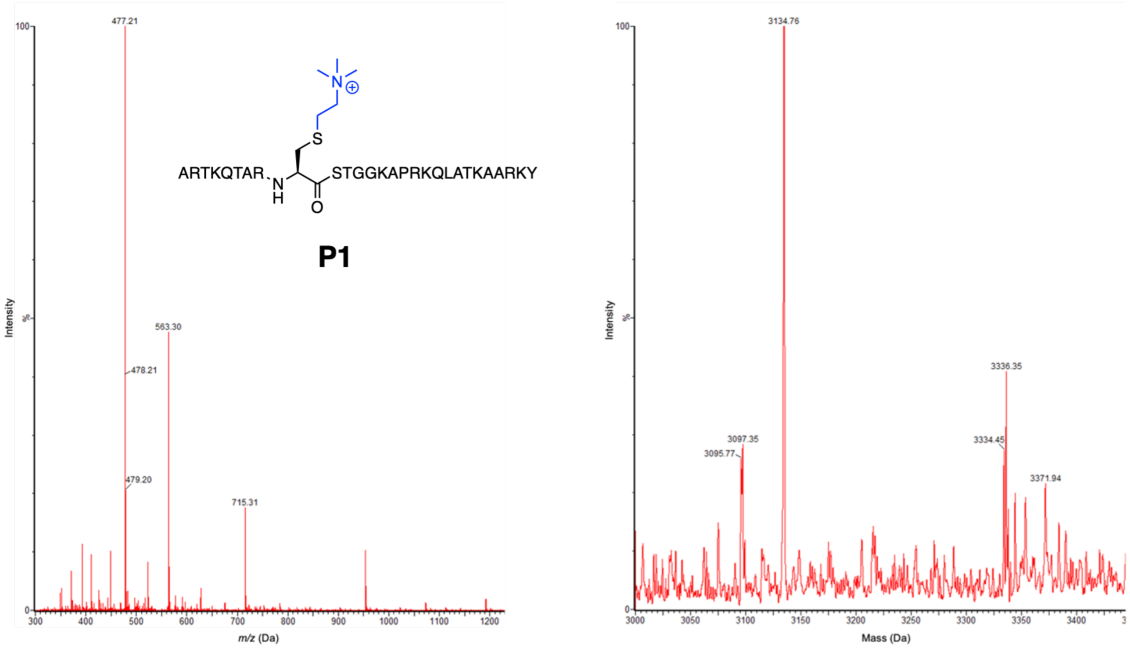

Observed MW: 3134.76 Da; Theoretical MW: 3134.73 Da

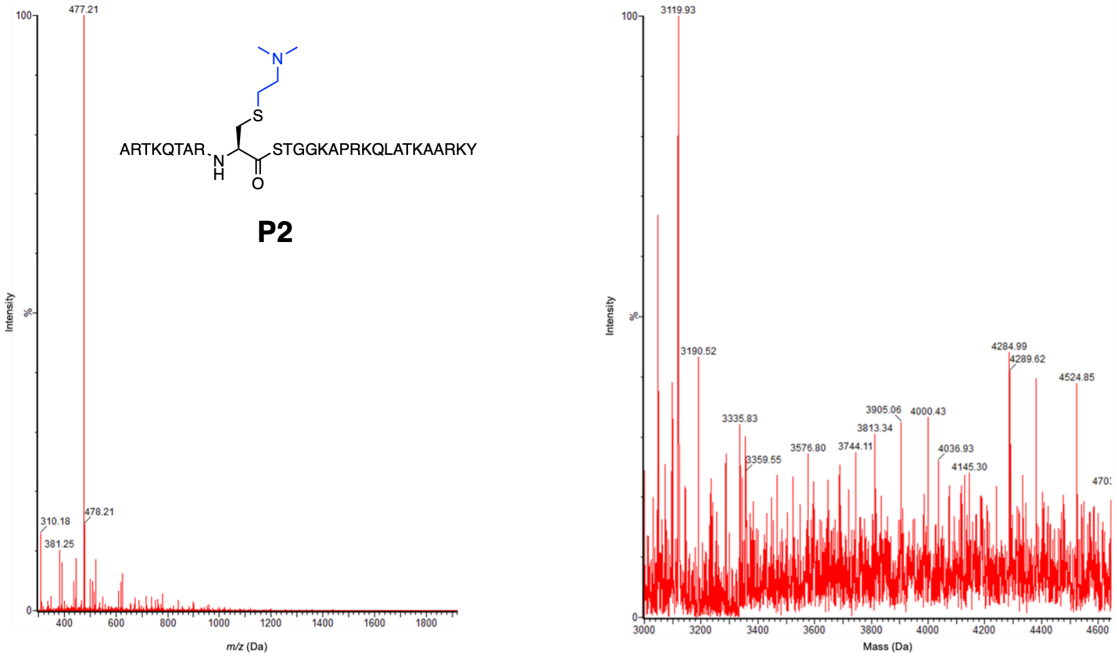

Observed MW: 3119.93 Da; Theoretical MW: 3119.70 Da

Figure S4 (cont.): ESI$^+$ and deconvoluted mass spectra for histone-mimicking peptides **P3** and **P4** before purification. Structures of the peptides are shown with single letter amino acid codes. The modification is shown in blue. Theoretical molecular weights were calculated in ChemDraw.

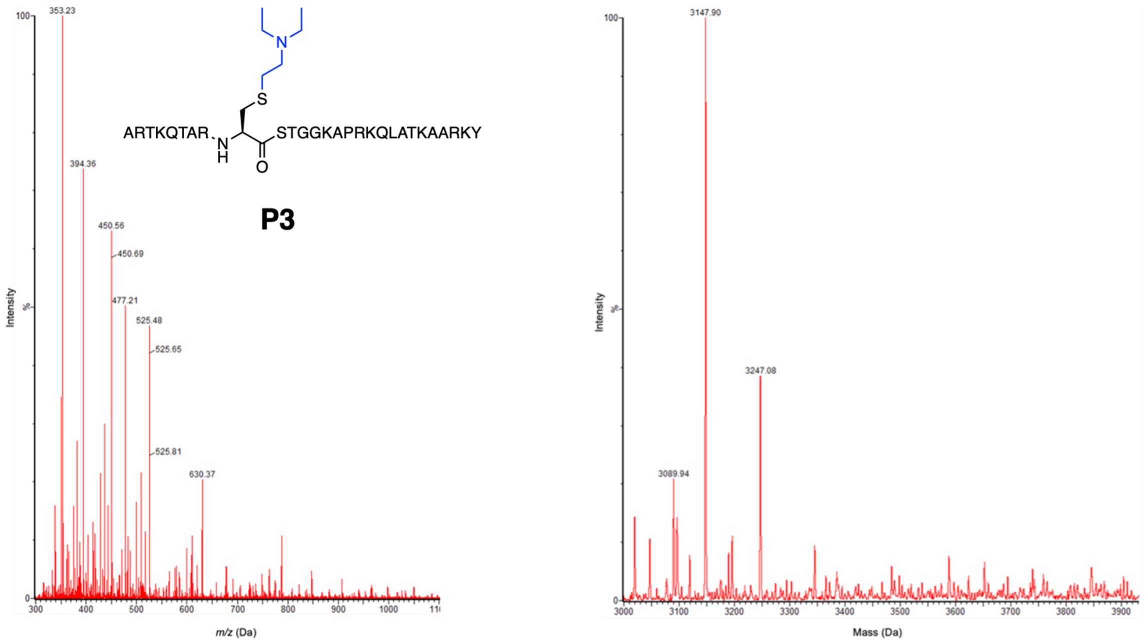

Observed MW: 3147.90 Da; Theoretical MW: 3147.75 Da

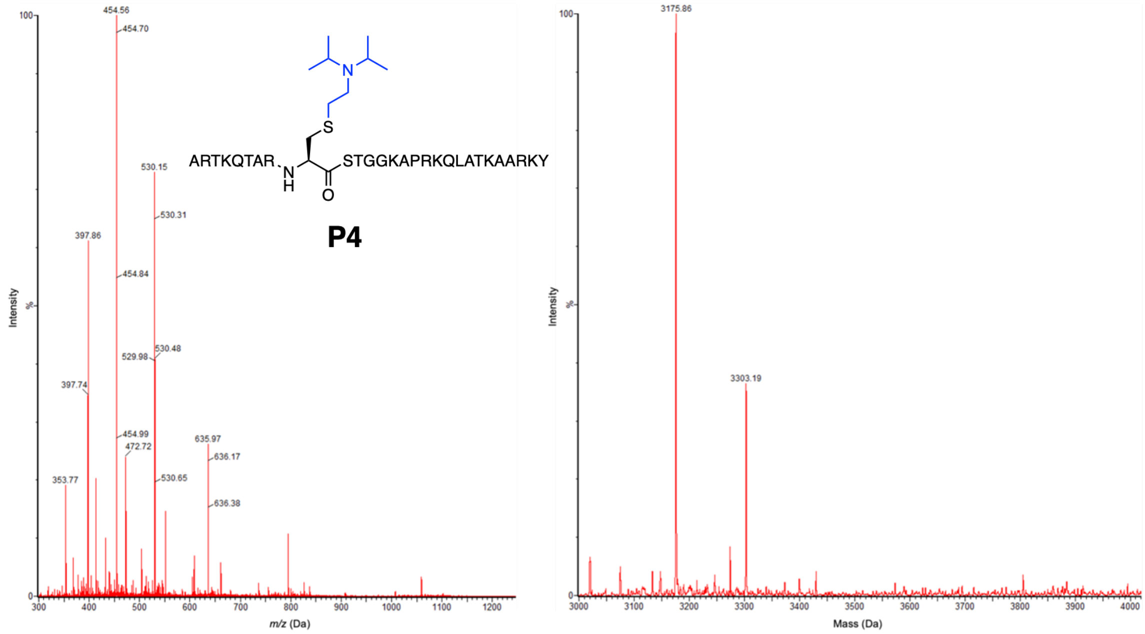

Observed MW: 3175.86 Da; Theoretical MW: 3175.81 Da

Figure S4 (cont.): ESI^{+} and deconvoluted mass spectra for histone-mimicking peptides **P5** and **P6** before purification. Structures of the peptides are shown with single letter amino acid codes. The modification is shown in blue. Theoretical molecular weights were calculated in ChemDraw.

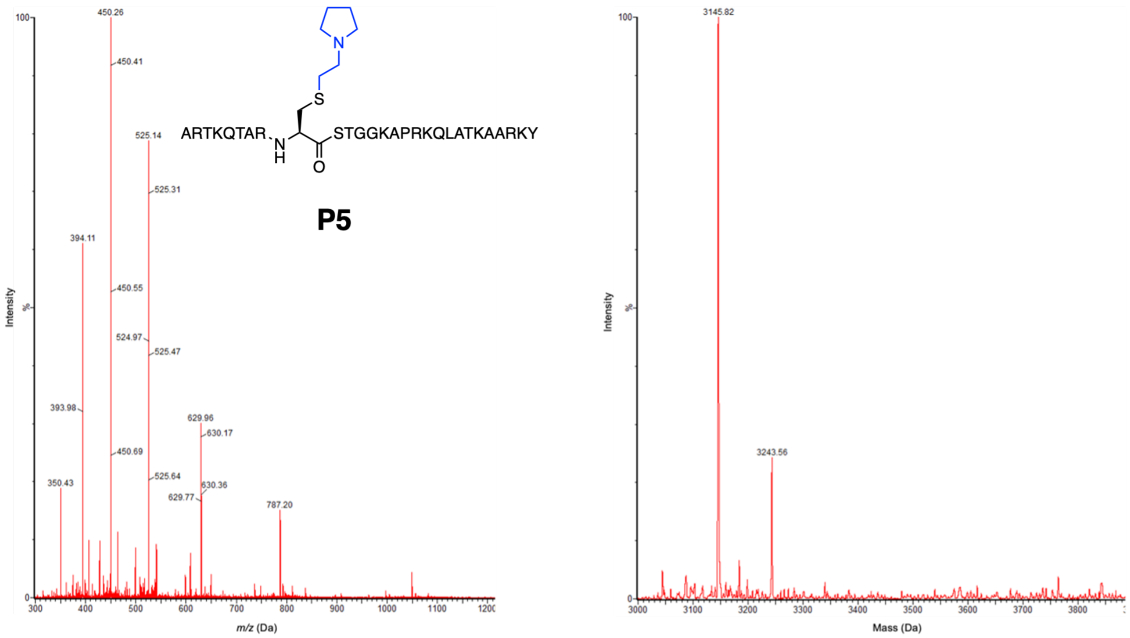

Observed MW: 3145.82 Da; Theoretical MW: 3145.74 Da

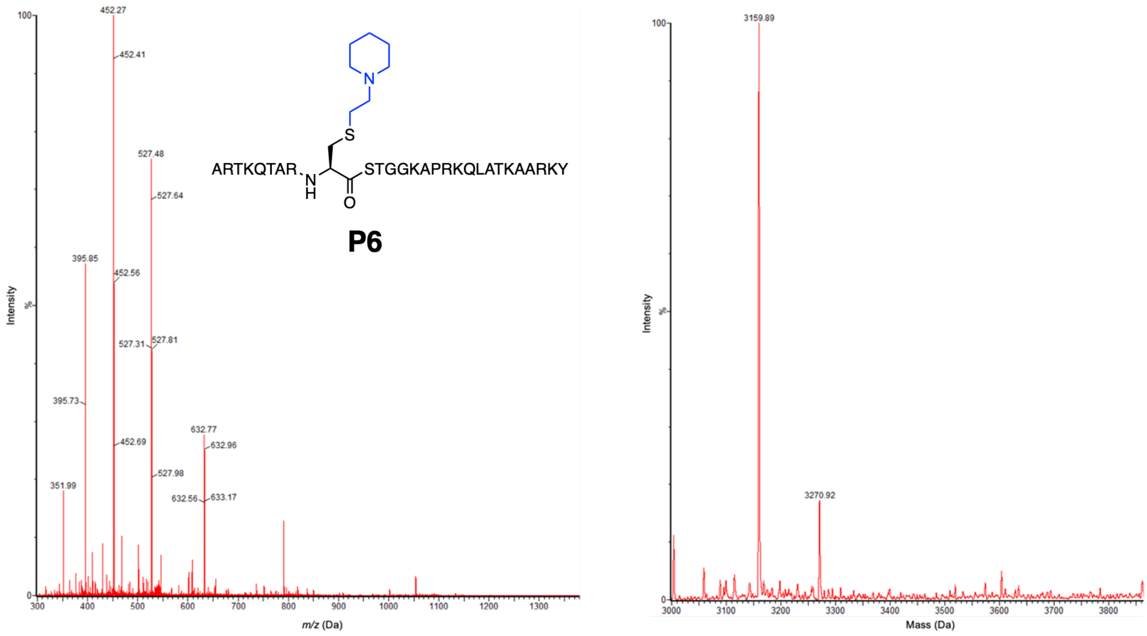

Observed MW: 3159.89 Da; Theoretical MW: 3159.76 Da

Figure S4 (cont.): ESI⁺ and deconvoluted mass spectra for histone-mimicking peptides **P7** and **P8** before purification. Structures of the peptides are shown with single letter amino acid codes. The modification is shown in blue. Theoretical molecular weights were calculated in ChemDraw.

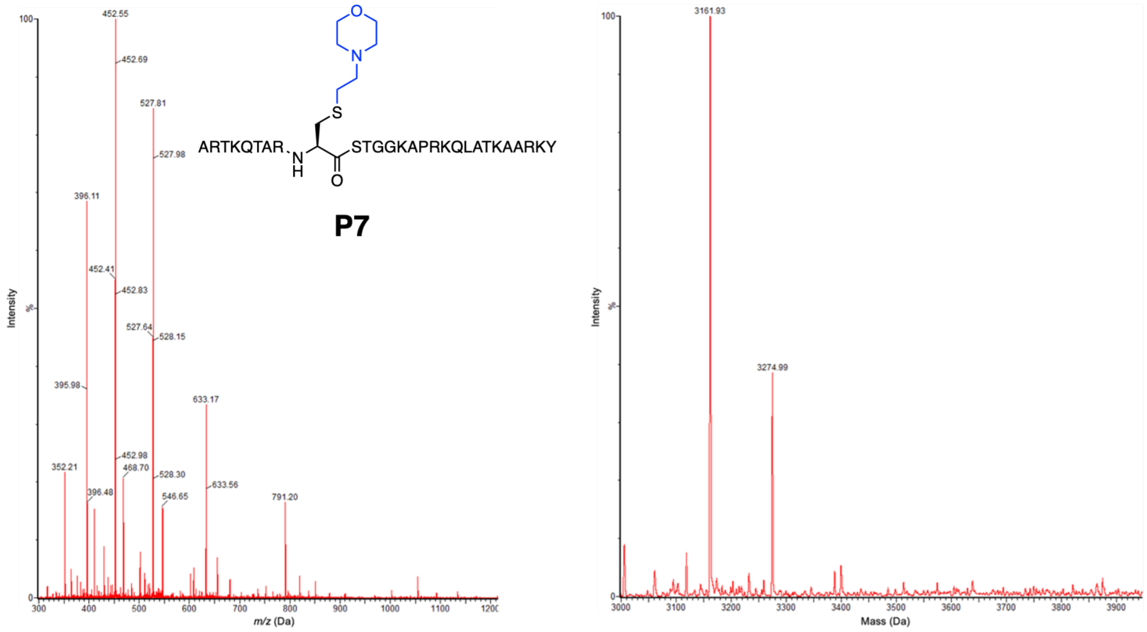

Observed MW: 3161.93 Da; Theoretical MW: 3161.74 Da

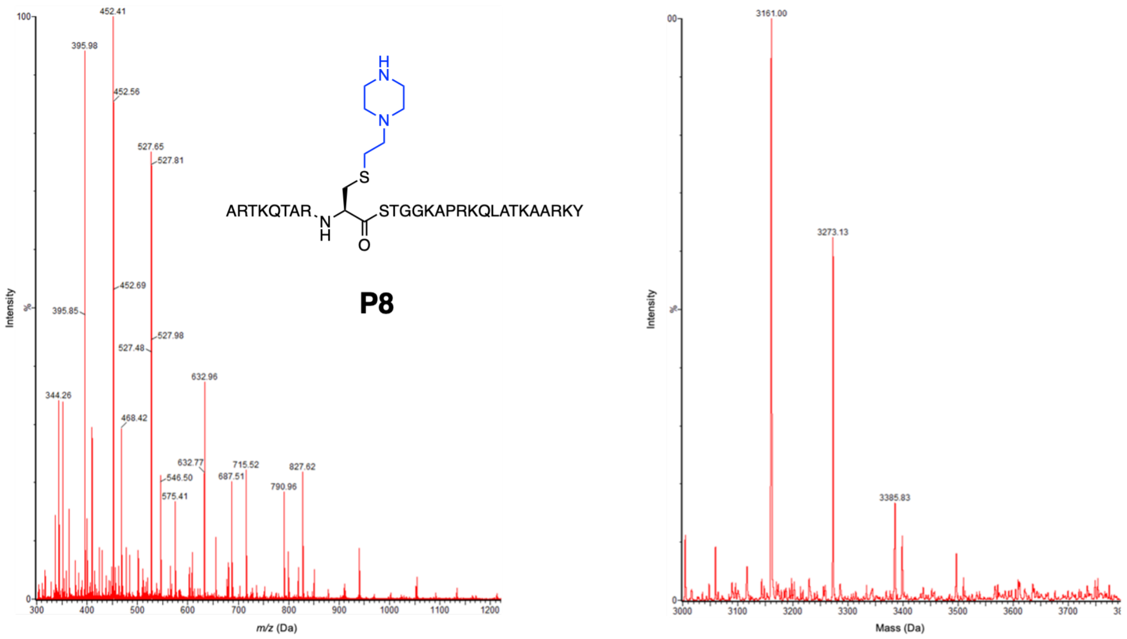

Observed MW: 3161.00 Da; Theoretical MW: 3160.75 Da

Figure S4 (cont.): ESI+ and deconvoluted mass spectra for histone-mimicking peptides **P9** and **P10** before purification. Structures of the peptides are shown with single letter amino acid codes. The modification is shown in blue. Theoretical molecular weights were calculated in ChemDraw.

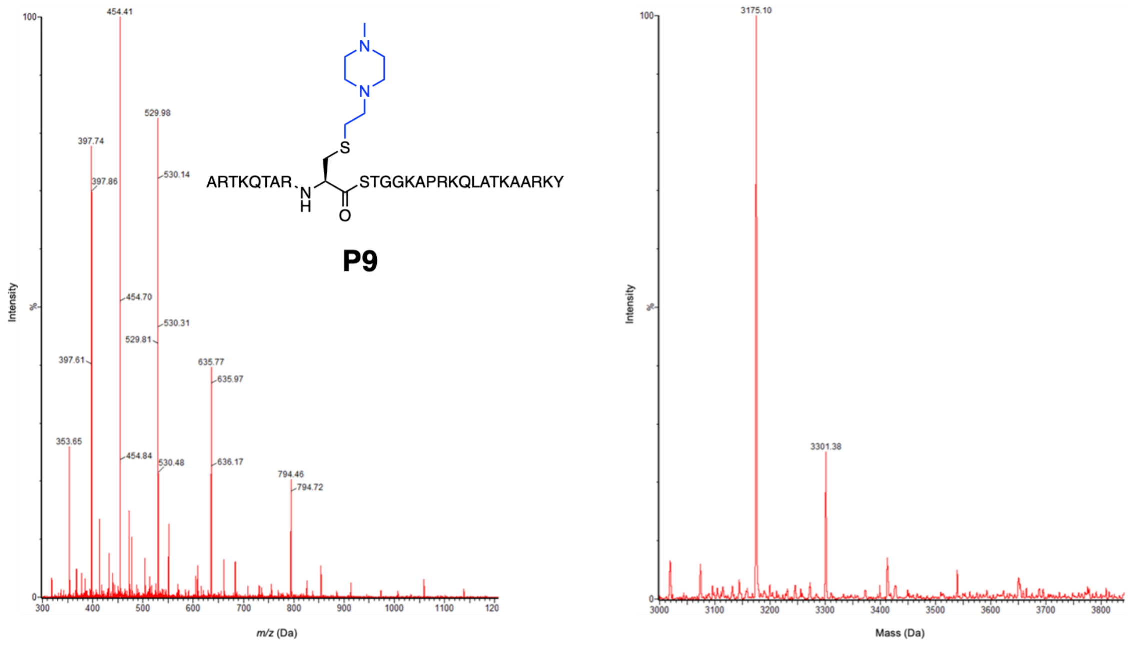

Observed MW: 3175.10 Da; Theoretical MW: 3174.78 Da

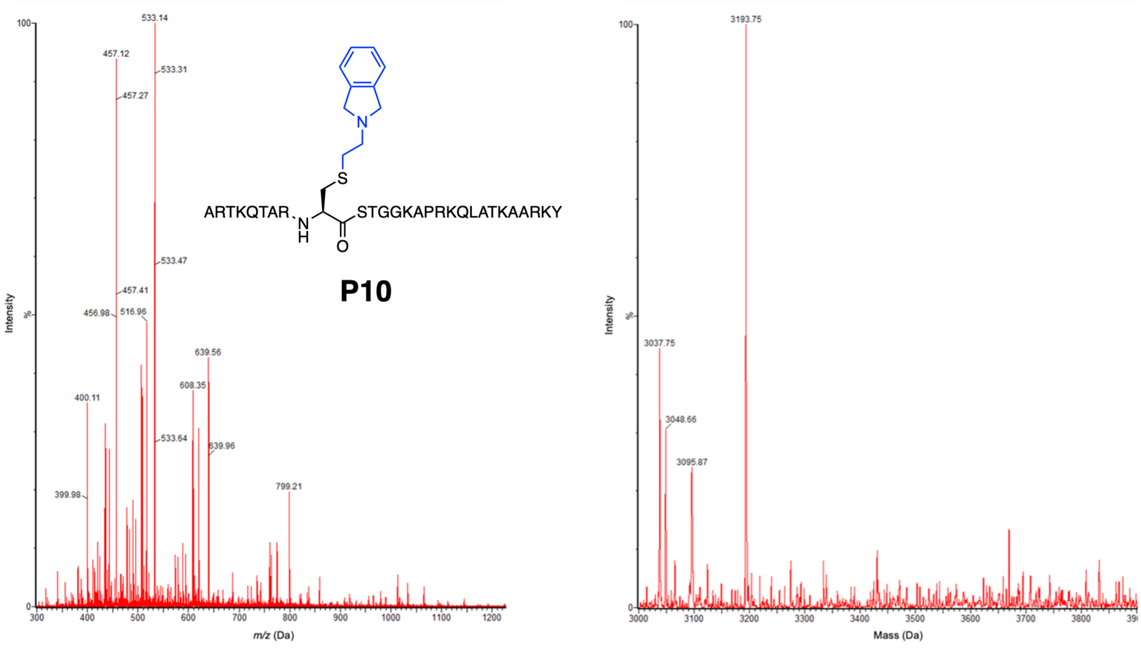

Observed MW: 3193.75 Da; Theoretical MW: 3193.78 Da

Figure S4 (cont.): ESI$^+$ and deconvoluted mass spectra for histone-mimicking peptides **P11** and **P12** before purification. Structures of the peptides are shown with single letter amino acid codes. The modification is shown in blue. Theoretical molecular weights were calculated in ChemDraw.

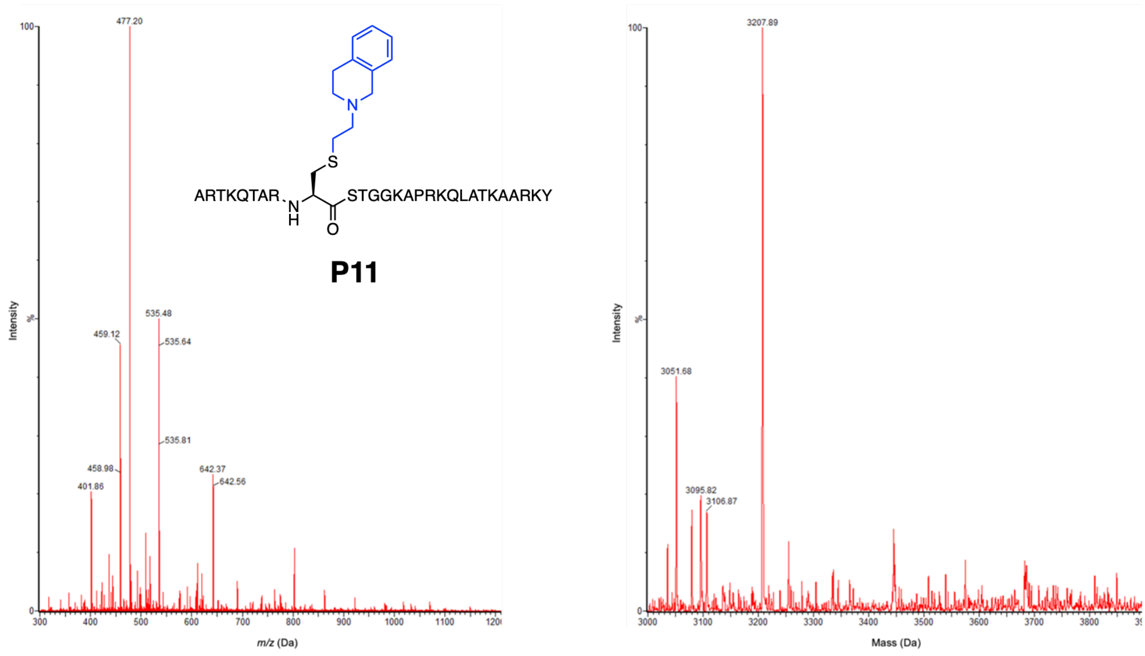

Observed MW: 3207.89 Da; Theoretical MW: 3207.81 Da

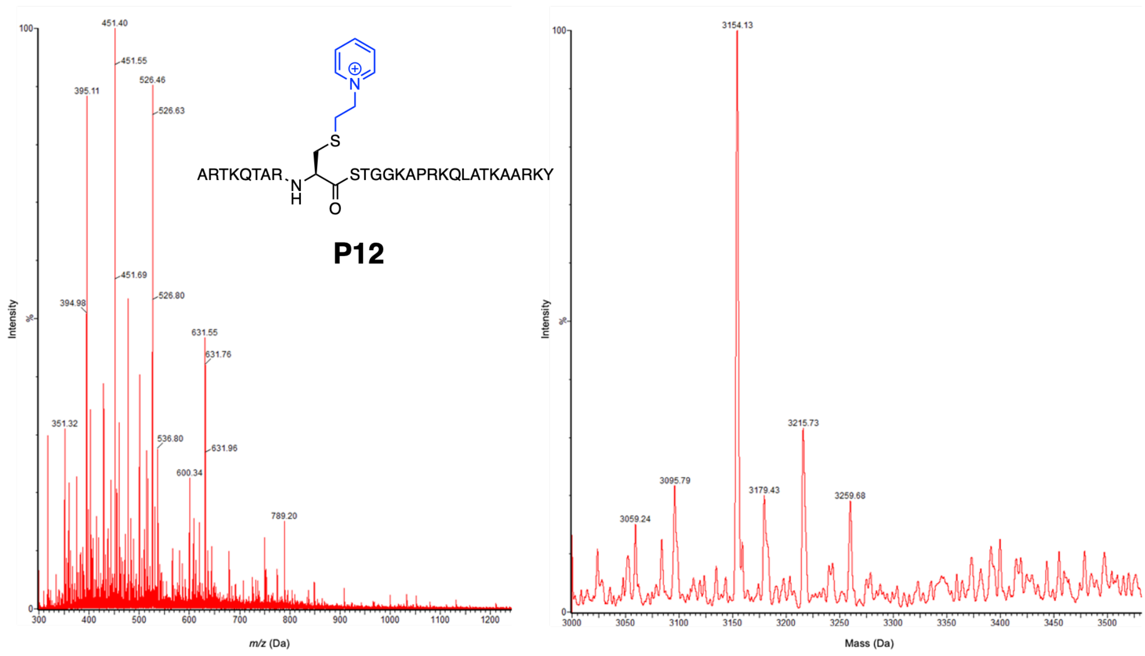

Observed MW: 3154.13 Da; Theoretical MW: 3154.72 Da

Figure S4 (cont.): ESI^{+} and deconvoluted mass spectra for histone-mimicking peptides **P13** and **P14** before purification. Structures of the peptides are shown with single letter amino acid codes. The modification is shown in blue. Theoretical molecular weights were calculated in ChemDraw.

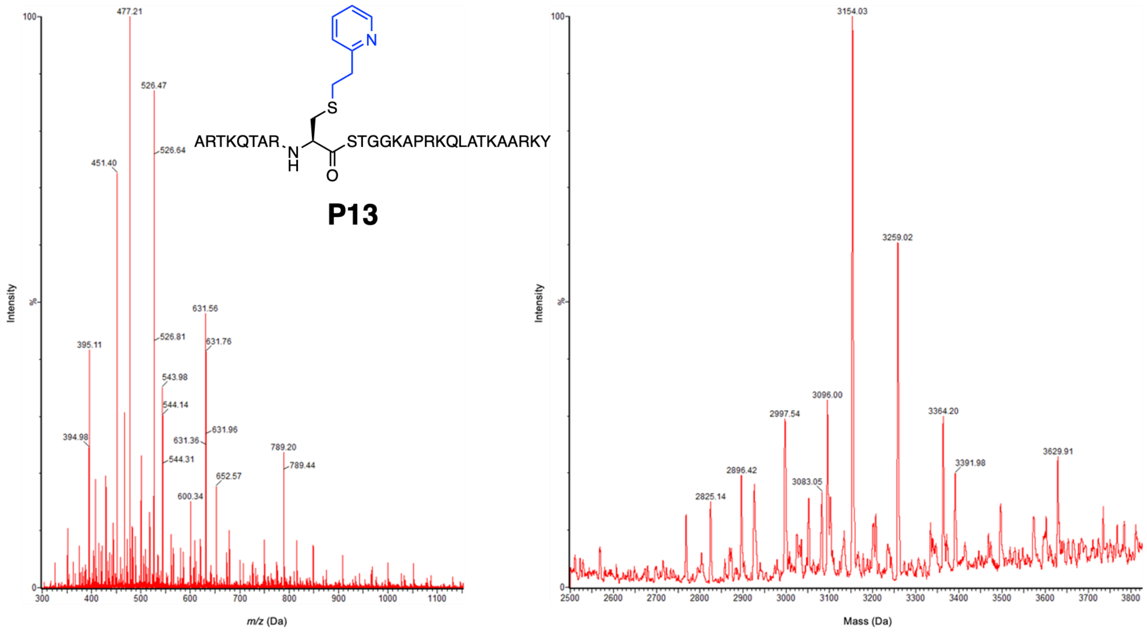

Observed MW: 3154.03 Da; Theoretical MW: 3153.72 Da

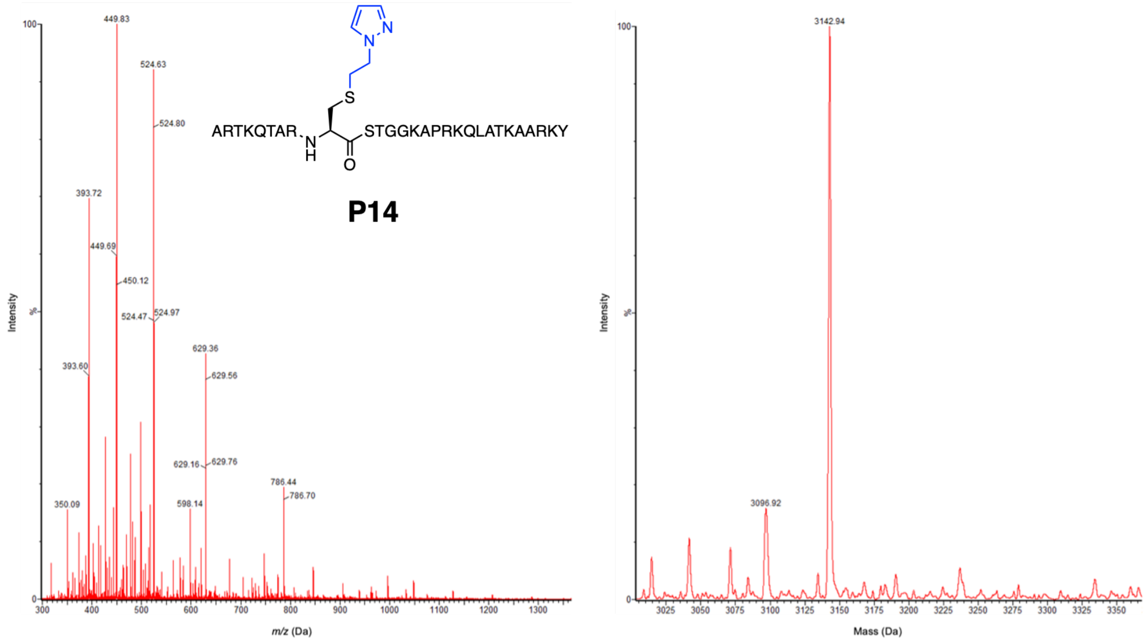

Observed MW: 3142.94 Da; Theoretical MW: 3142.69 Da

Figure S4 (cont.): ESI$^+$ and deconvoluted mass spectra for histone-mimicking peptides **P15** and **P16** before purification. Structures of the peptides are shown with single letter amino acid codes. The modification is shown in blue. Theoretical molecular weights were calculated in ChemDraw.

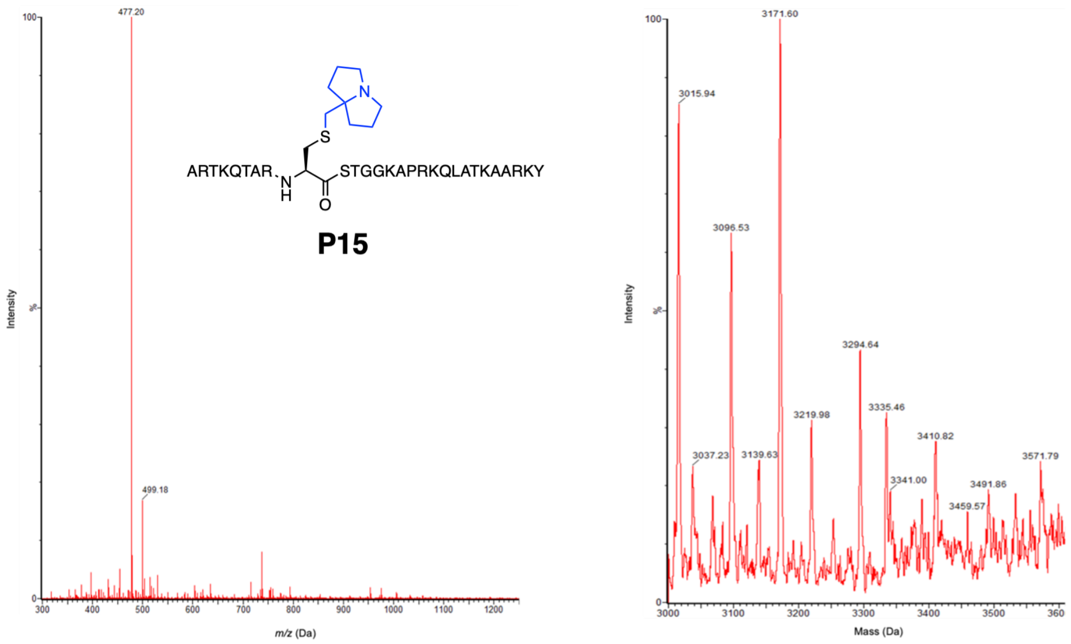

Observed MW: 3171.60 Da; Theoretical MW: 3171.78 Da

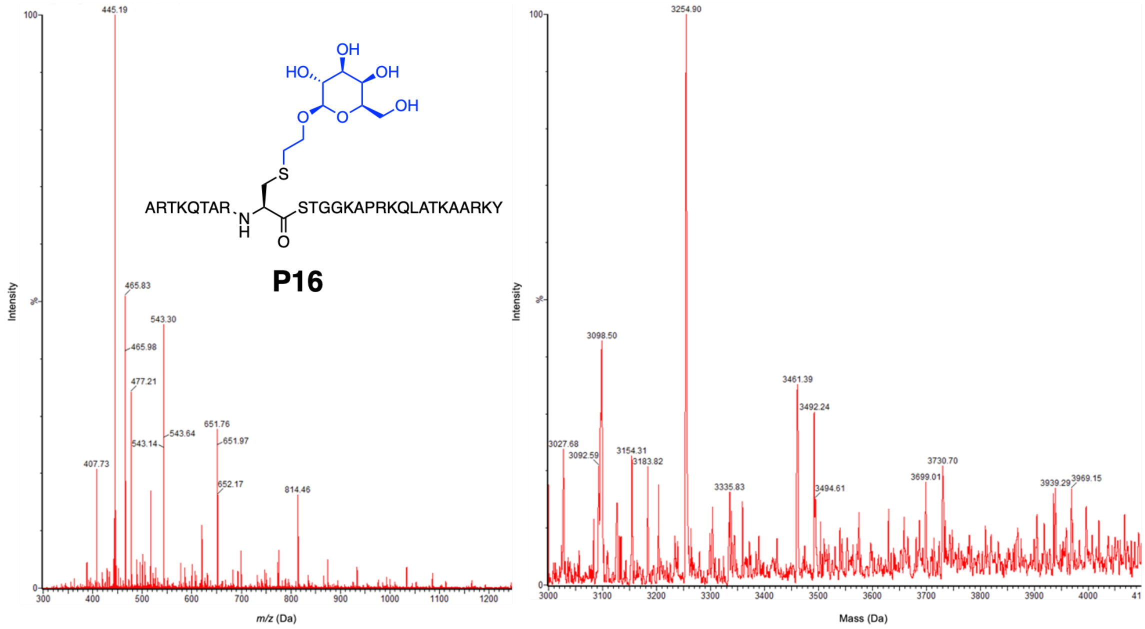

Observed MW: 3254.90 Da; Theoretical MW: 3254.77 Da

Figure S5: MALDI-TOF MS (blue) and simulated spectra (red, [M+H]$^+$) for **P2** and **P3**. Spectra were simulated in Bruker flexAnalaysis software. **P1** could not be observed by MALDI-TOF MS. Structures of the peptides are shown with single letter amino acid codes. The modification is shown in blue.

P2

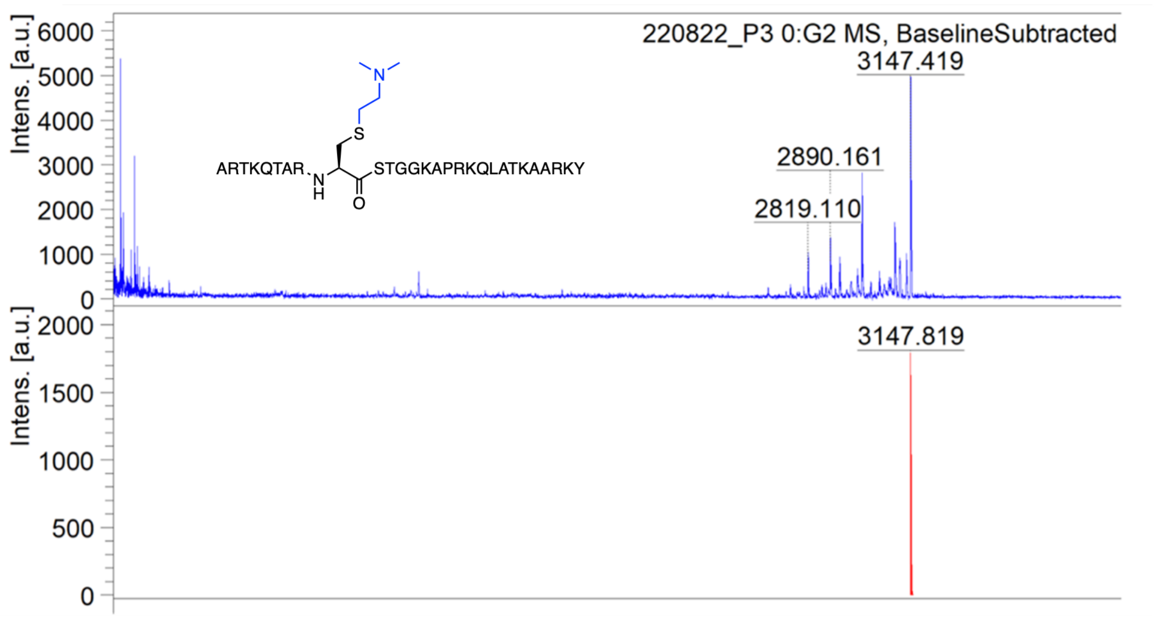

P3

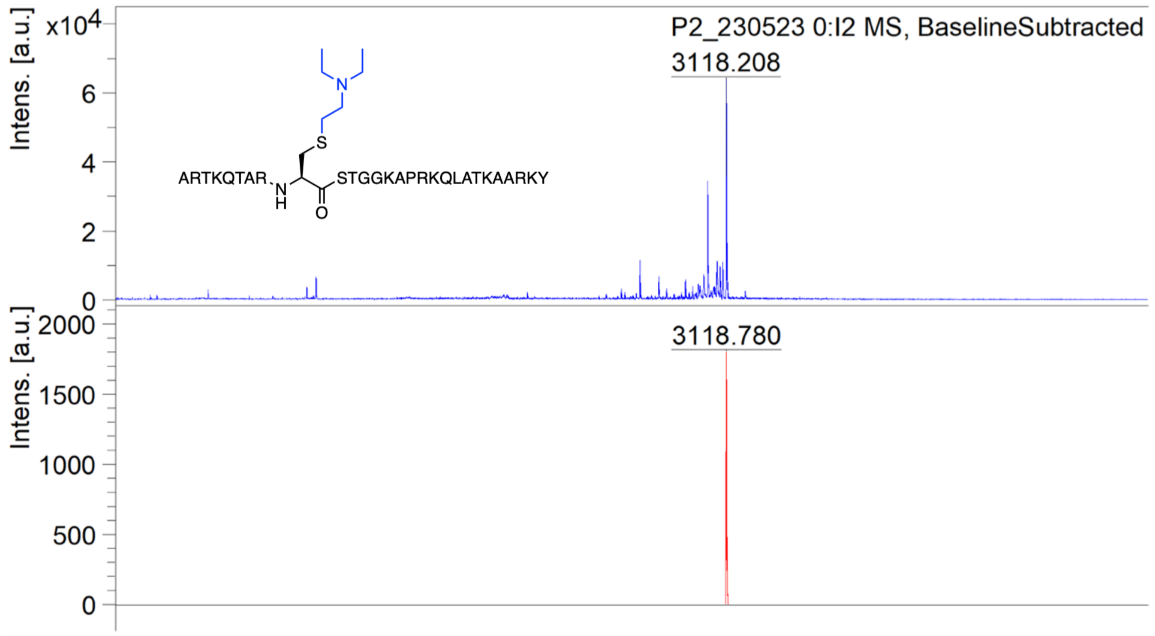

Figure S5 (cont.): MALDI-TOF MS (blue) and simulated spectra (red, $[M+H]^+$) for **P4** and **P5**. Spectra were simulated in Bruker flexAnalaysis software. Structures of the peptides are shown with single letter amino acid codes. The modification is shown in blue.

P4

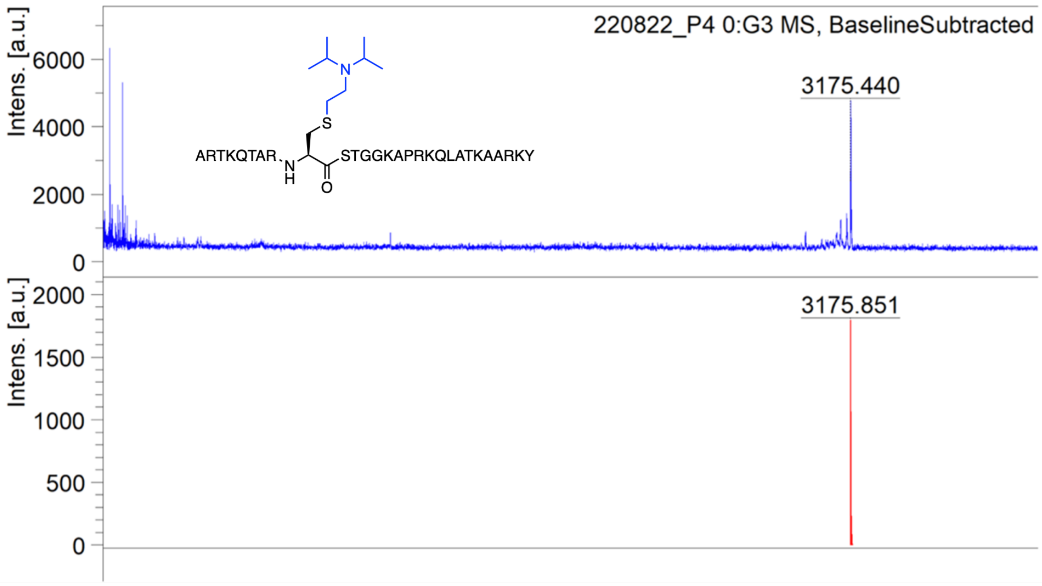

P5

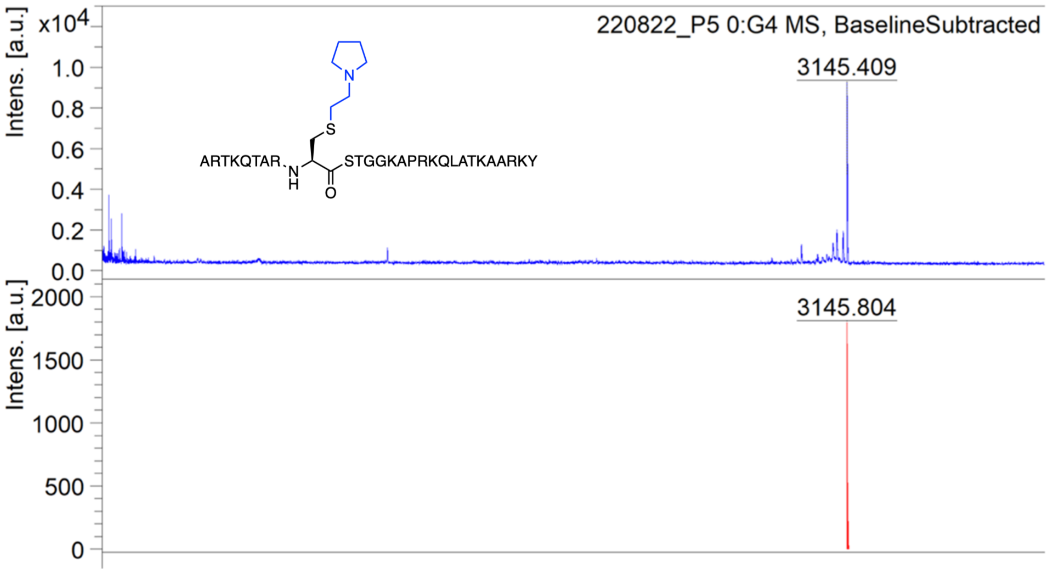

Figure S5 (cont.): MALDI-TOF MS (blue) and simulated spectra (red, $[M+H]^{+}$) for **P6** and **P7**. Spectra were simulated in Bruker flexAnalaysis software. Structures of the peptides are shown with single letter amino acid codes. The modification is shown in blue.

P6

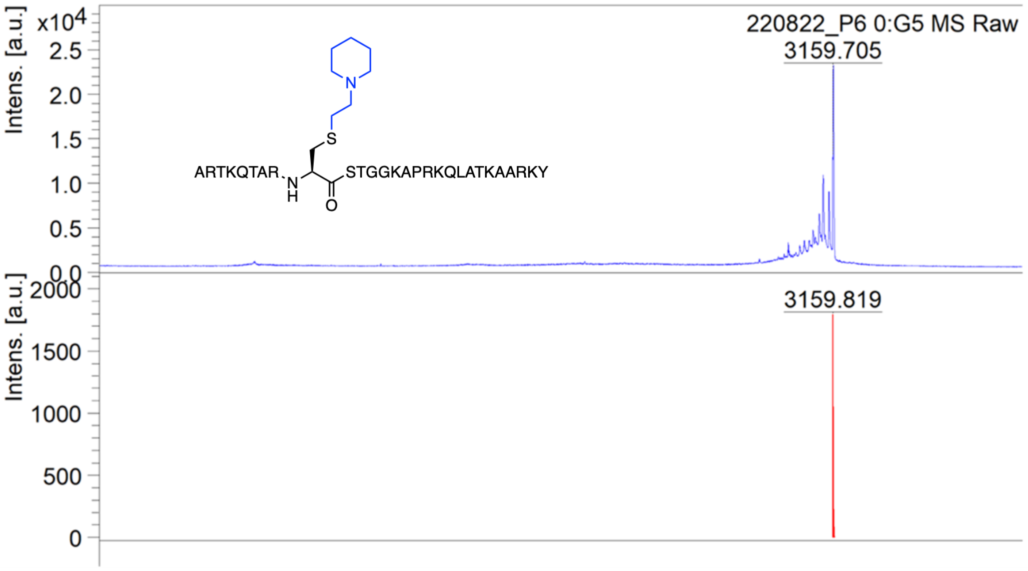

P7

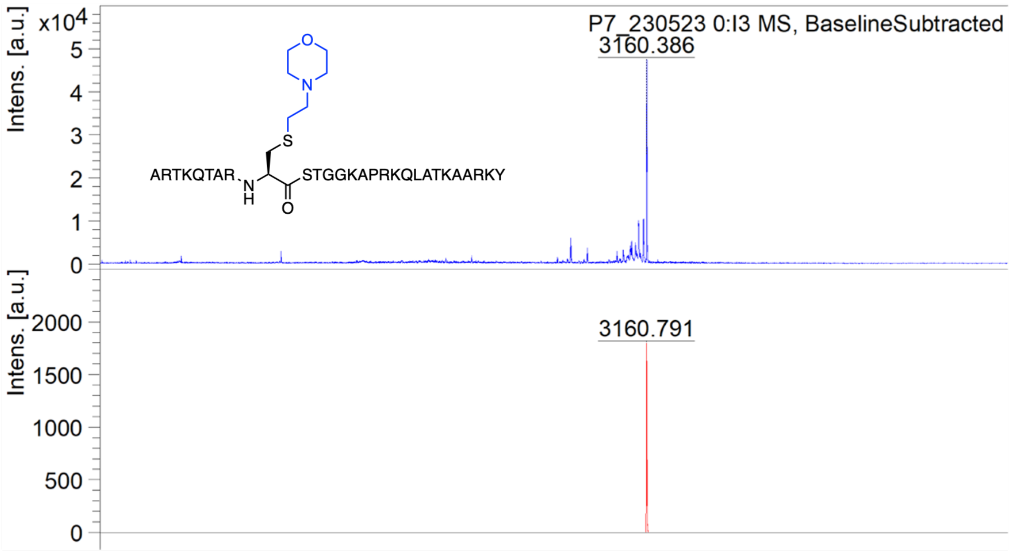

Figure S5 (cont.): MALDI-TOF MS (blue) and simulated spectra (red, $[M+H]^+$) for **P8** and **P9**. Spectra were simulated in Bruker flexAnalaysis software. Structures of the peptides are shown with single letter amino acid codes. The modification is shown in blue.

P8

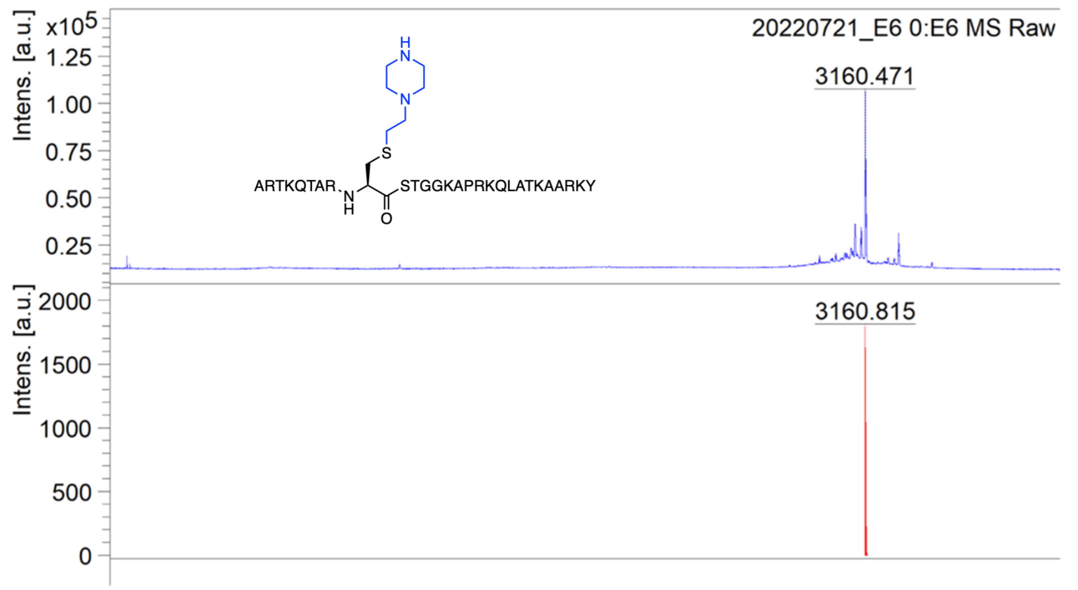

P9

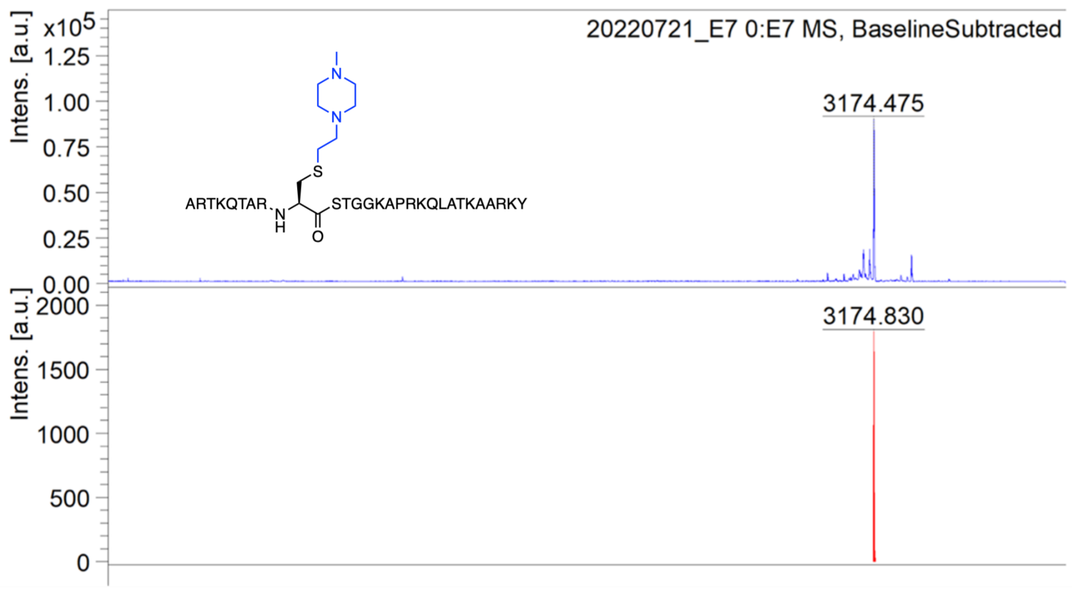

Figure S5 (cont.): MALDI-TOF MS (blue) and simulated spectra (red, $[M+H]^+$) for **P10** and **P11**. Spectra were simulated in Bruker flexAnalaysis software. Structures of the peptides are shown with single letter amino acid codes. The modification is shown in blue.

P10

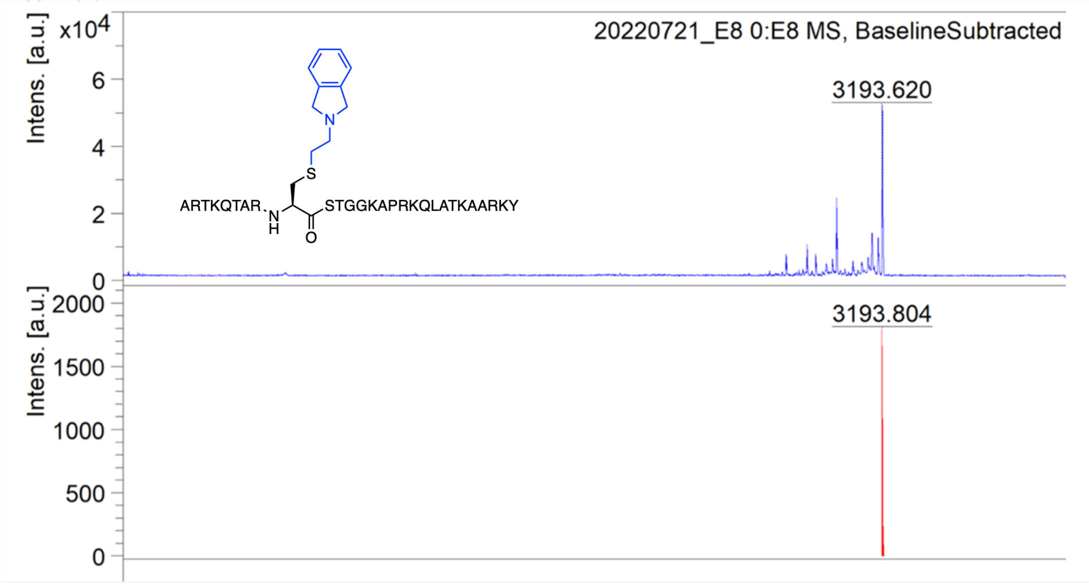

P11

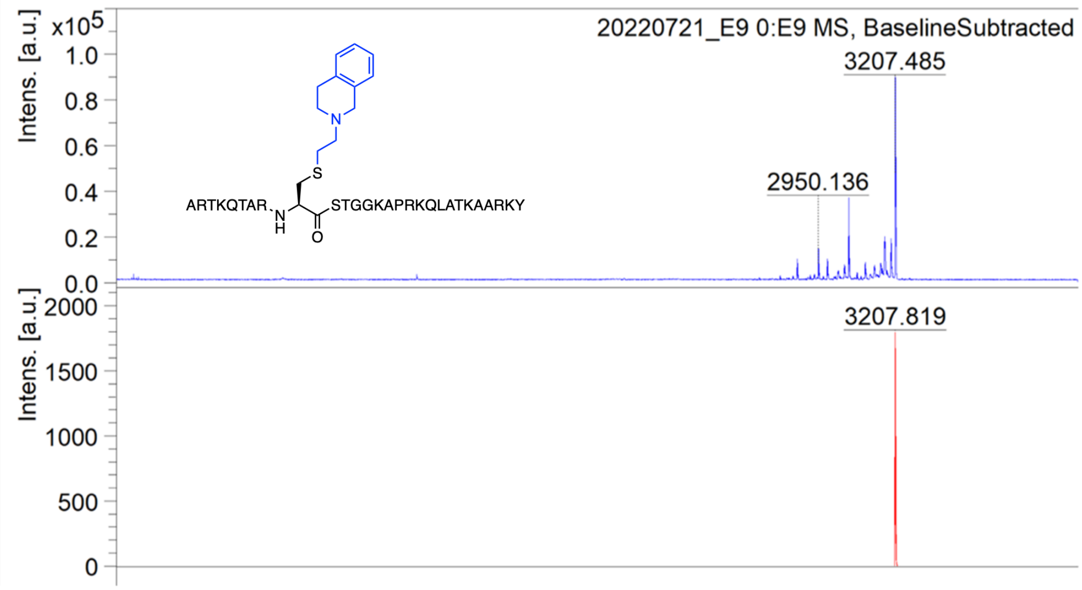

Figure S5 (cont.): MALDI-TOF MS (blue) and simulated spectra (red, $[M+H]^+$) for **P13** and **P14**. Spectra were simulated in Bruker flexAnalaysis software. **P12** could not be observed by MALDI-TOF MS. Structures of the peptides are shown with single letter amino acid codes. The modification is shown in blue.

P13

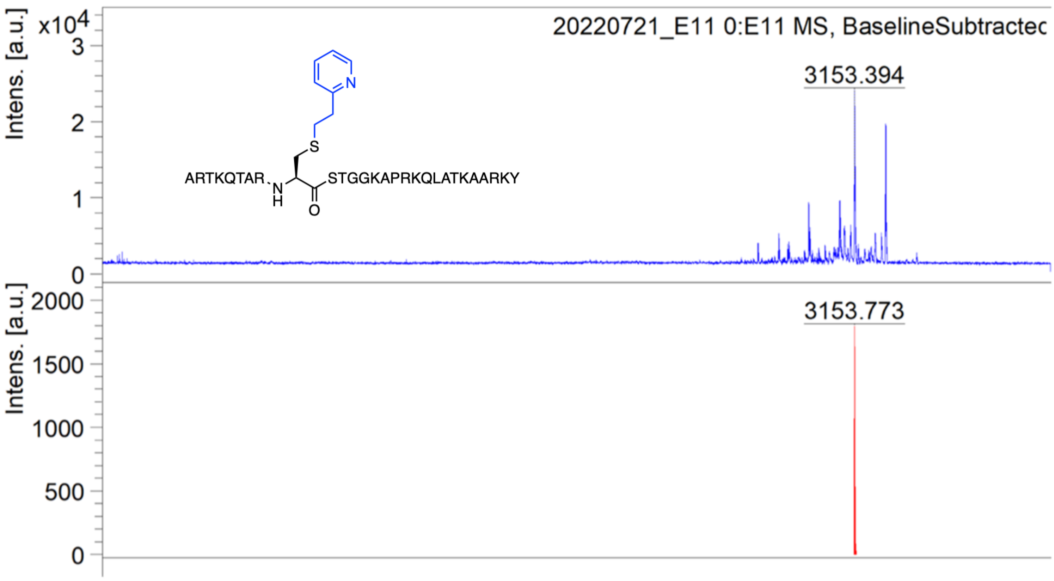

P14

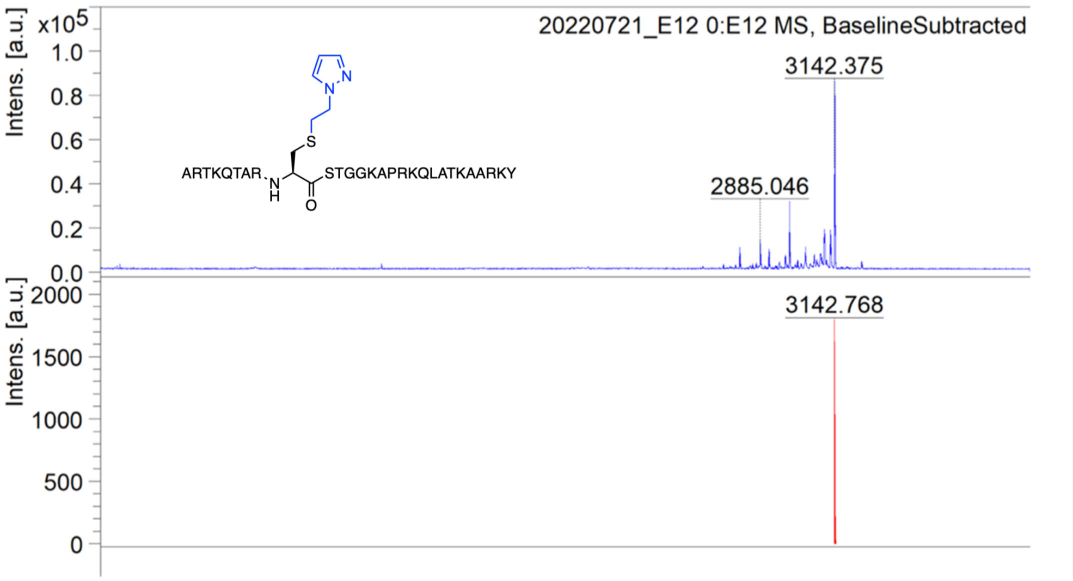

Figure S5 (cont.): MALDI-TOF MS (blue) and simulated spectra (red, [M+H]$^+$) for **P15** and **P16**. Spectra were simulated in Bruker flexAnalaysis software. Structures of the peptides are shown with single letter amino acid codes. The modification is shown in blue.

P15

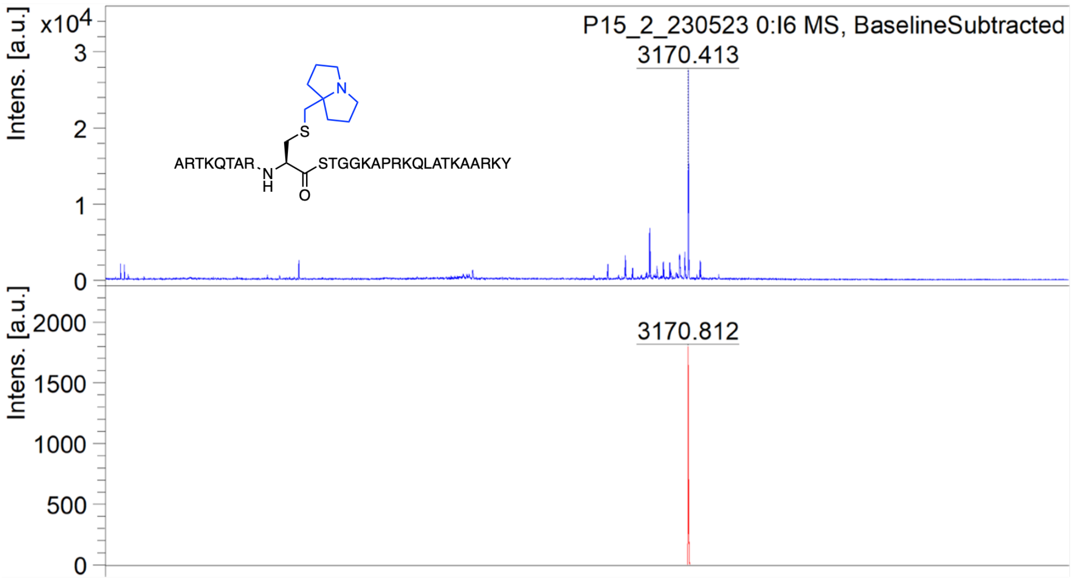

P16

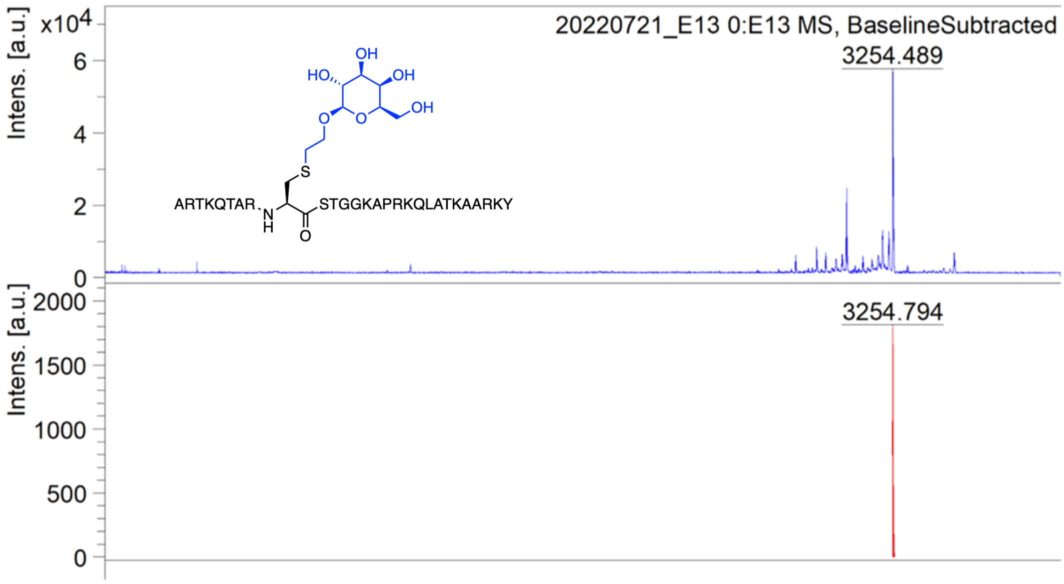

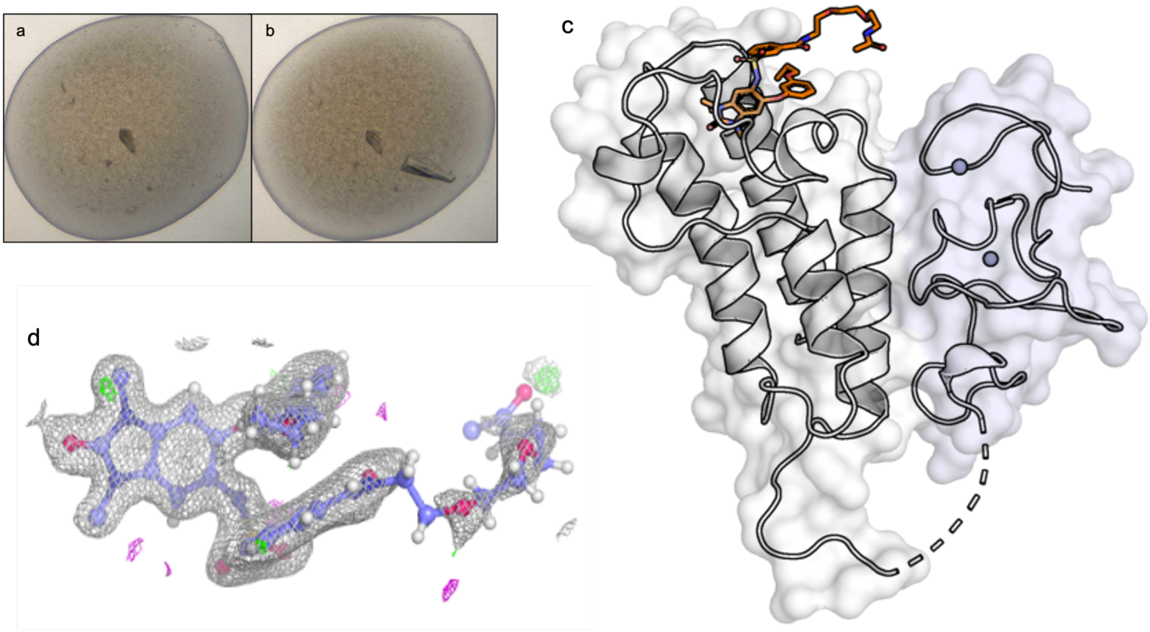

Figure S6: a,b) Images of crystalisation wells for the co-crystallisation of BRD ligand **32** with TRIM24 (PHD-BRD) after a) 28 days and b) 56 days. *Conditions*: 1.8 M ammonium sulfate, 0.1 M HEPES (pH 7.3), PEG-400 5 % *v/v*, 2:1 (protein : reservoir). Reservoir solutions are aqueous; c) Unit cell for the crystal structure of TRIM24 (PHD-BRD) in complex with BRD ligand **32** (1.5 Å, PDB ID: 9GDG). The PHD and BRD are coloured in light blue and white, respectively. Grey spheres represent Zn^{2+} ions. Dashed lines represent unresolved residues in disordered regions; d) Experimental electron density around BRD ligand **32** from the PDB validation report. Limited electron density was observed for the PEG chain and so it was placed in an arbitrary position in the crystal structure.

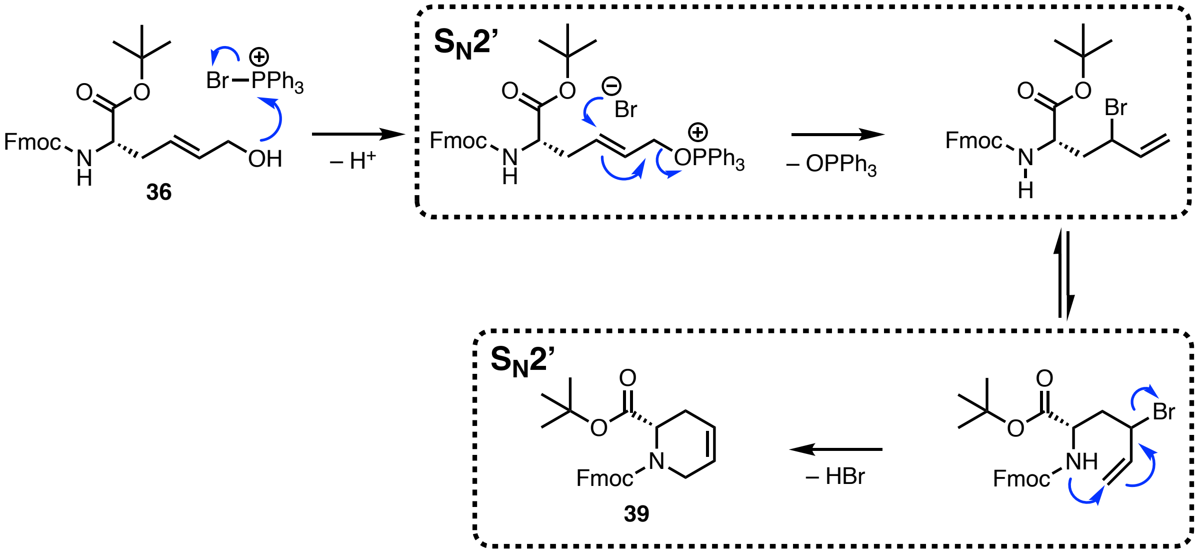

Figure S7: Proposed mechanism for the formation of compound **39** from allylic alcohol **36** involving successive S_N2' mechanisms.

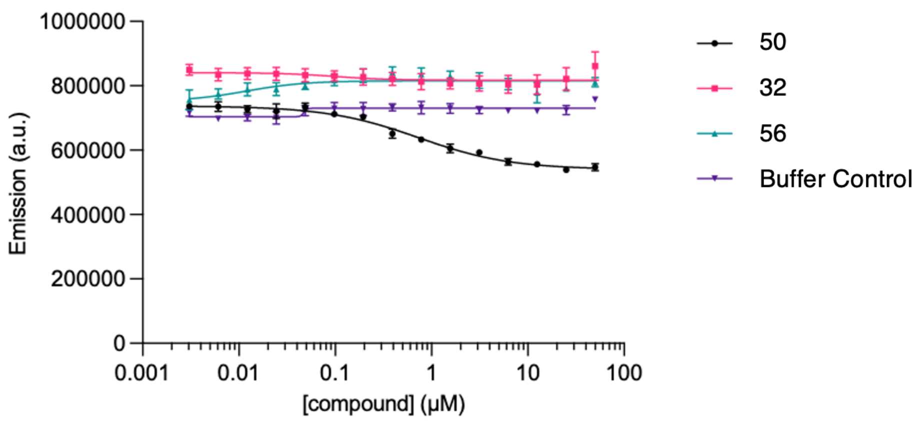

Figure S8: AlphaScreen® TruHits assay dose-response curves. Dose-response curves were obtained in triplicate with error bars indicating the standard deviation.

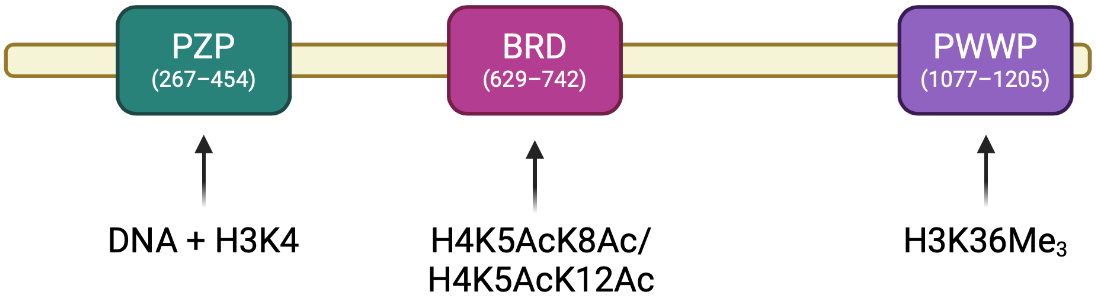

Figure S9: Domain composition of BRPF1. The PZP domain (residues 267–454) binds bivalently to DNA and unmodified H3K4, while the BRD (residues 629–742) and PWWP (residues 1077–1205) domains bind to diacetylated H4 and $H3K36Me_3$, respectively. Figure created with BioRender.com.[6–8]

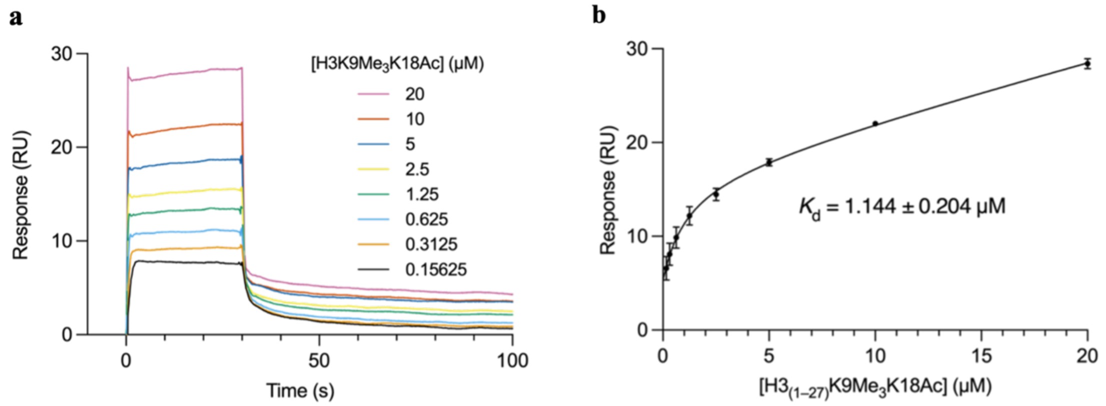

Figure S10: a) Representative reference-subtracted SPR sensograms for the binding of $H3_{(1\text{-}27)}K9Me_3K18Ac$ to His_6-TRIM24 (PHD-BRD); b) Binding saturation curve for the binding of $H3_{(1\text{-}27)}K9Me_3K18Ac$ to His_6-TRIM24 (PHD-BRD). K_d values (equilibrium analysis) are quoted as the mean of triplicate data ± standard error of the mean. Error bars indicate the standard deviation. $H3_{(1\text{-}27)}K9Me_3K18Ac$ ITC K_d = 1.94 ± 0.11 μM.[9]

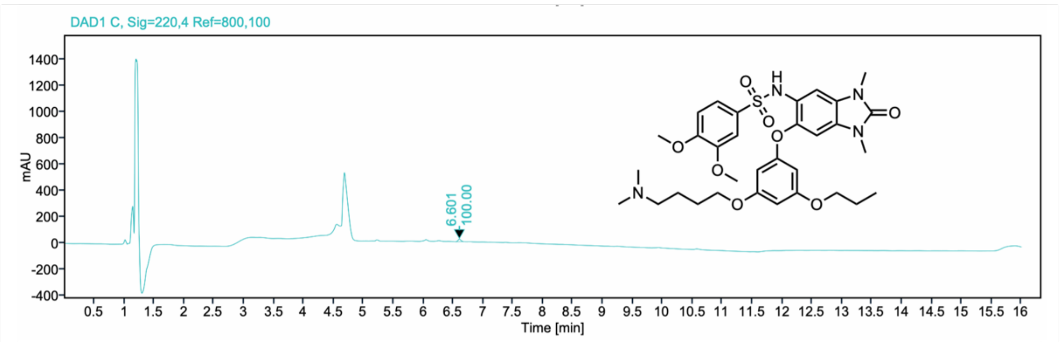

Figure S11: LC-MS chromatogram of IACS9571 (t_R = 6.60 min) in HBS buffer supplemented with protease inhibitors.

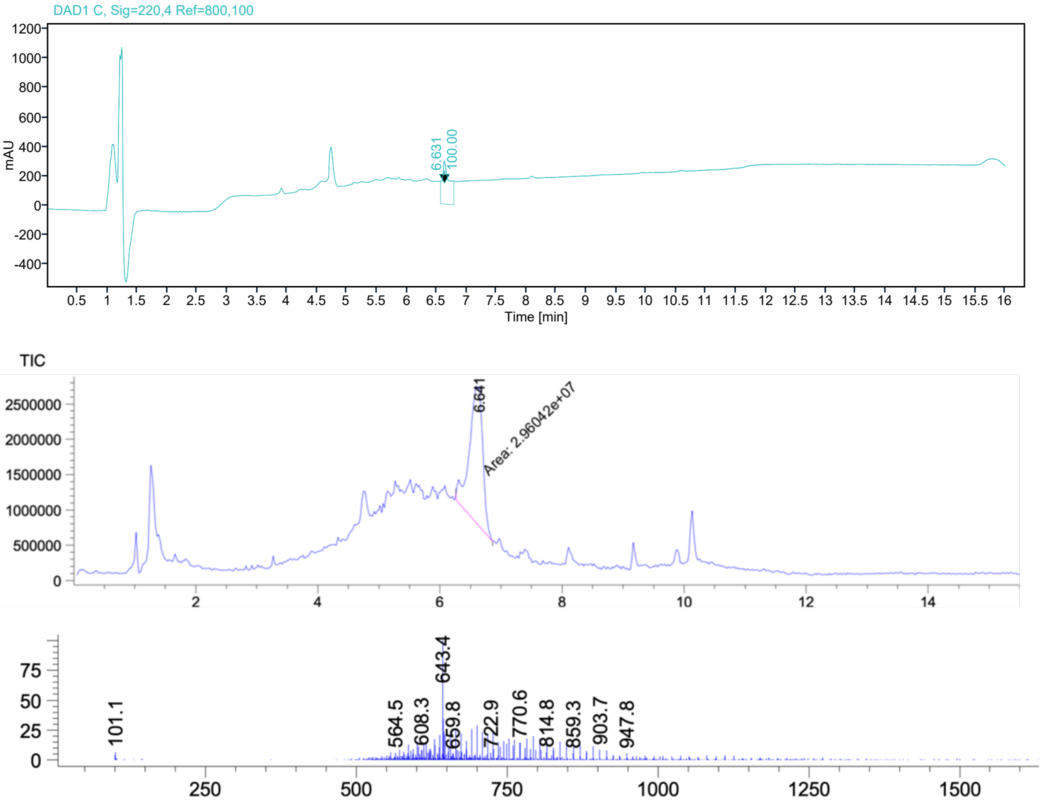

Figure S12: LC-MS chromatograms and ESI$^+$ mass spectrum (t_R = 6.641 min) from the cell permeability assay for IACS9571 (*n* = 1). IACS9571 exact mass = 642.27 (observed mass = 643.4, $[M+H]^+$).

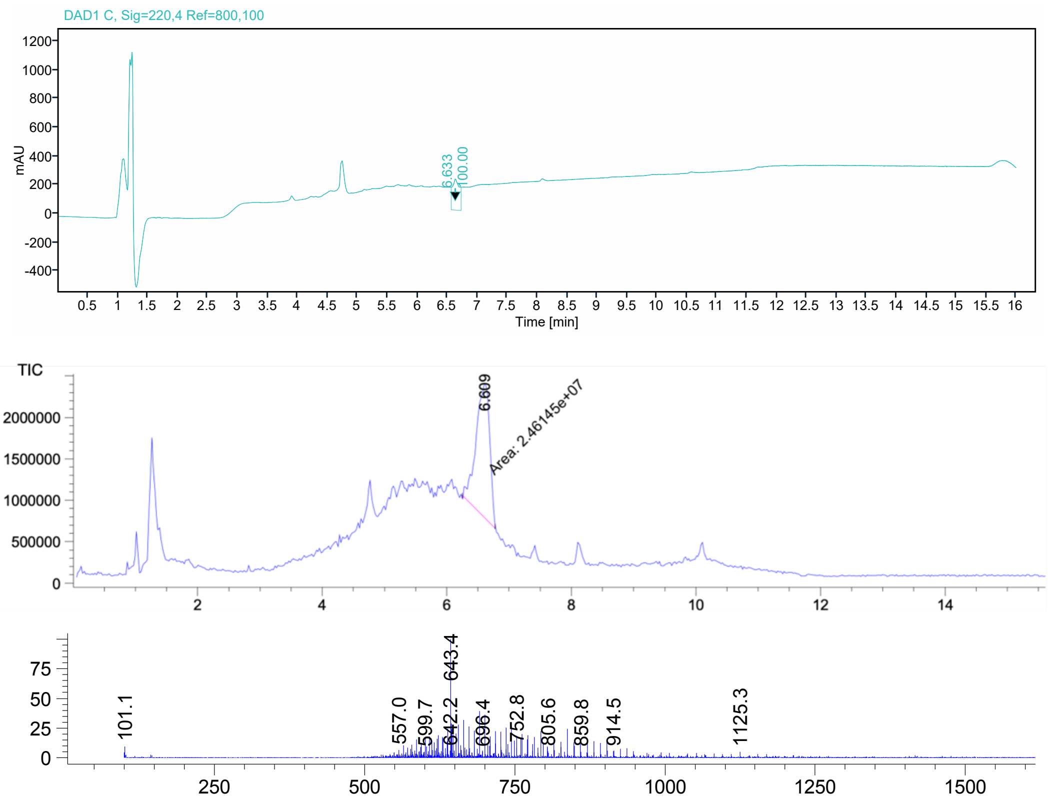

Figure S13: LC-MS chromatograms and ESI$^+$ mass spectrum (t_R = 6.609 min) from the cell permeability assay for IACS9571 (*n* = 2). IACS9571 exact mass = 642.27 (observed mass = 643.4, $[M+H]^+$).

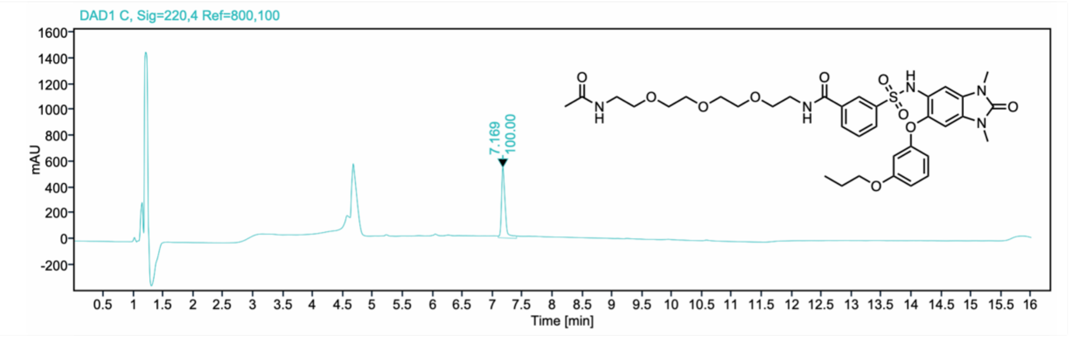

Figure S14: LC-MS chromatogram of compound **33** (t_R = 7.17 min) in HBS buffer supplemented with protease inhibitors.

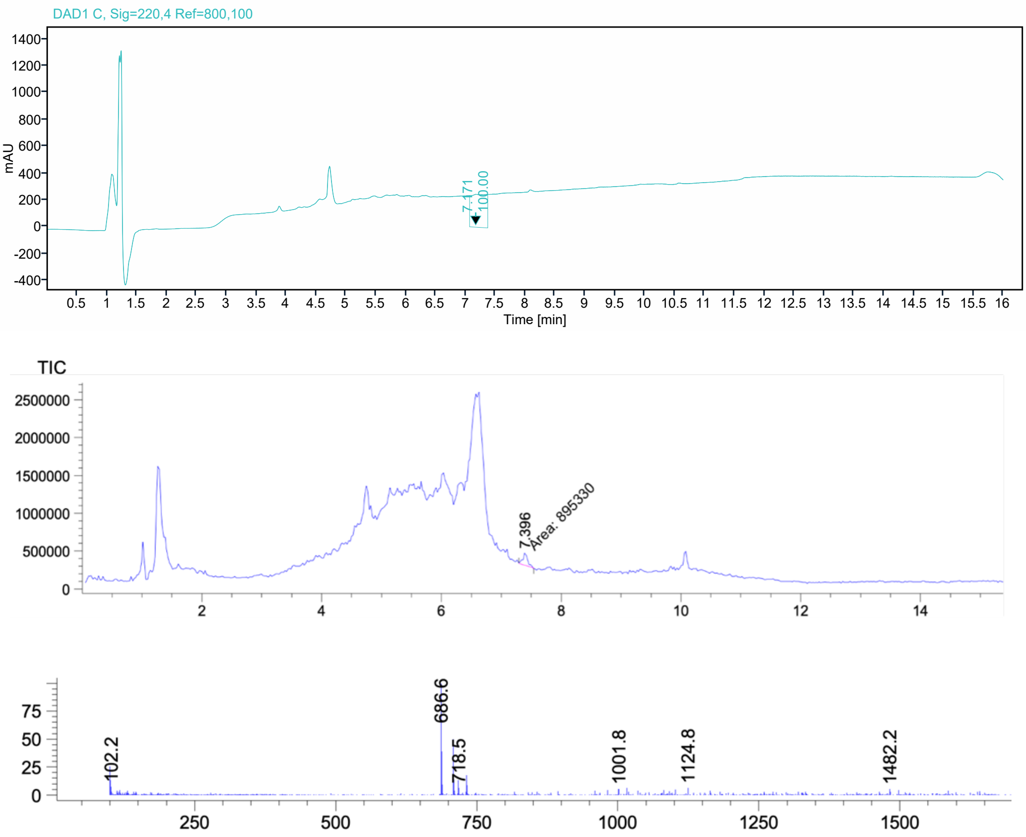

Figure S15: LC-MS chromatograms and ESI$^+$ mass spectrum (t_R = 7.396 min) from the cell permeability assay for compound **33** (n = 1). Compound **33** exact mass = 727.29 (observed mass = 686.6, [M−Ac+2H]$^+$).

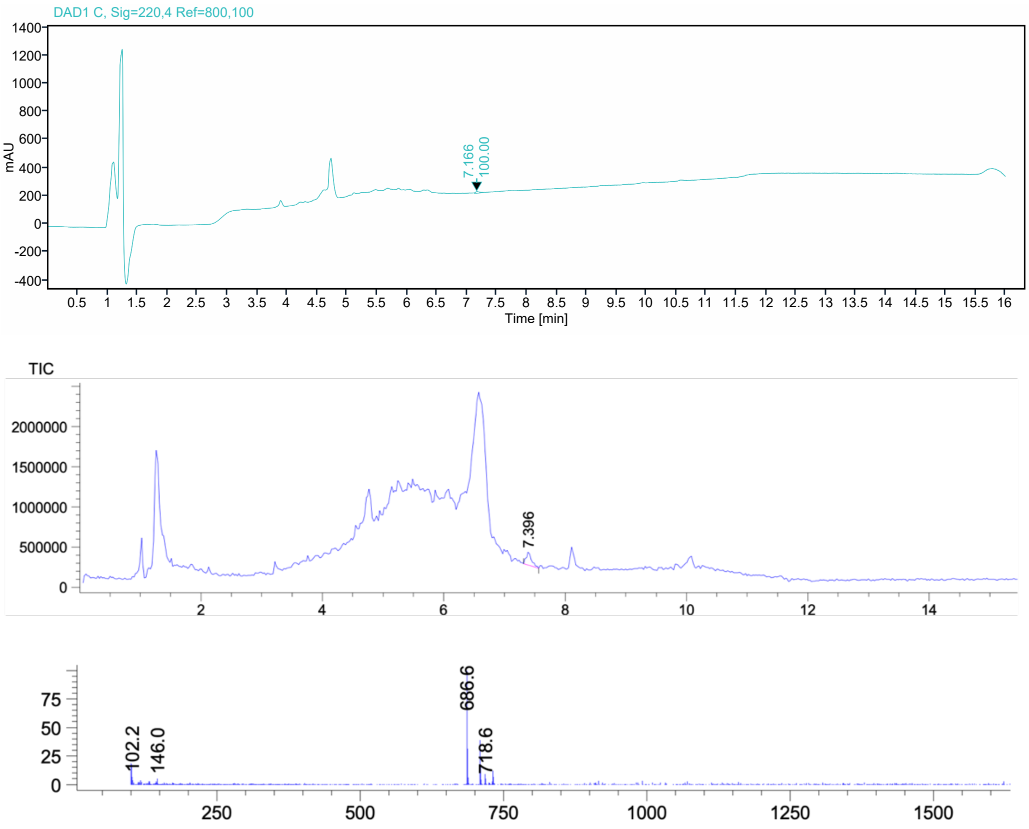

Figure S16: LC-MS chromatograms and ESI$^+$ mass spectrum (t_R = 7.396 min) from the cell permeability assay for compound **33** (n = 2). Compound **33** exact mass = 727.29 (observed mass = 686.6, [M−Ac+2H]$^+$).

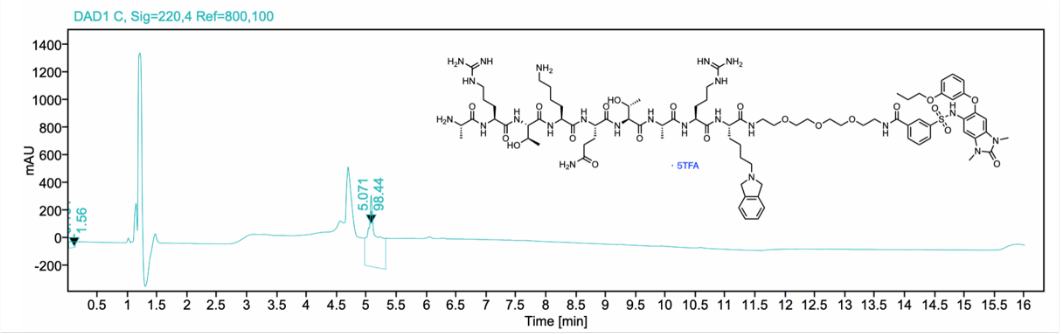

Figure S17: LC-MS chromatogram of PDC5 (compound **51**) (t_R = 5.07 min) in HBS buffer supplemented with protease inhibitors.

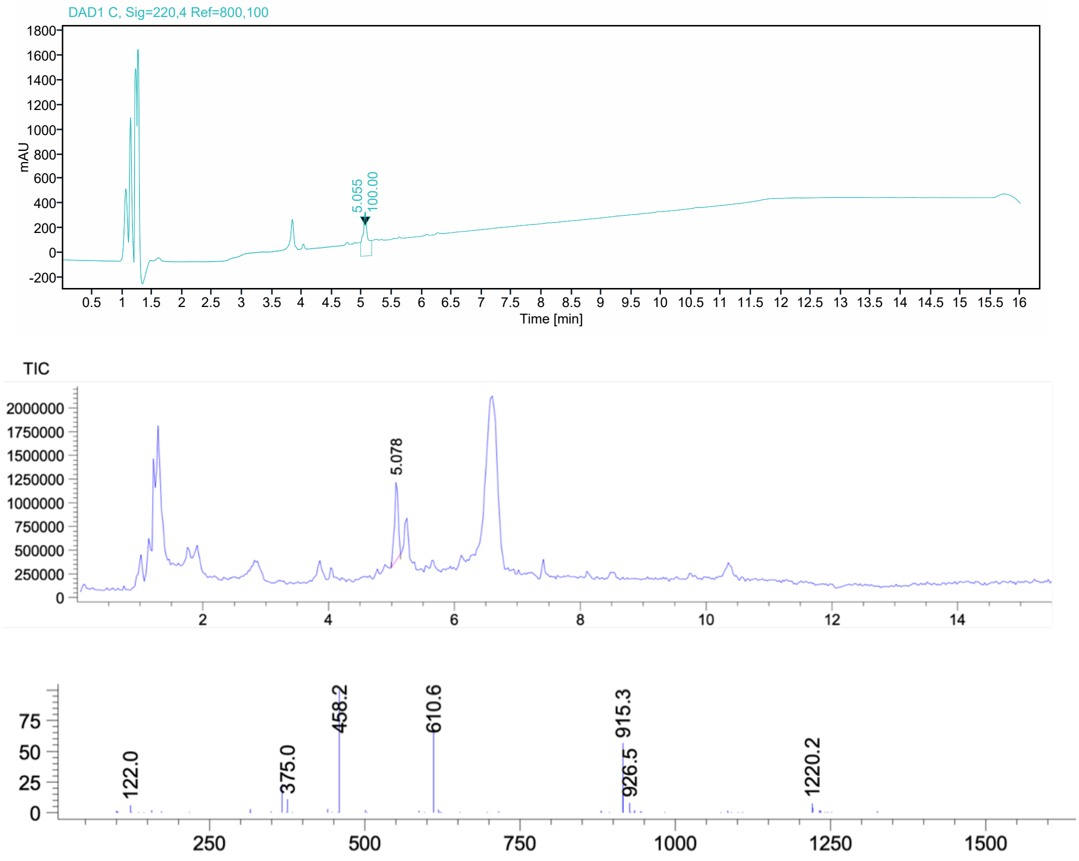

Figure S18: LC-MS chromatograms and ESI$^+$ mass spectrum (t_R = 5.078 min) from the cell permeability assay for PDC5 (compound **51**) (n = 1). PDC5 exact mass = 1827.95 (observed masses = 915.3, $[M-5TFA+2H]^{2+}$; 610.6, $[M-5TFA+3H]^{3+}$; 458.2, $[M-5TFA+4H]^{4+}$).

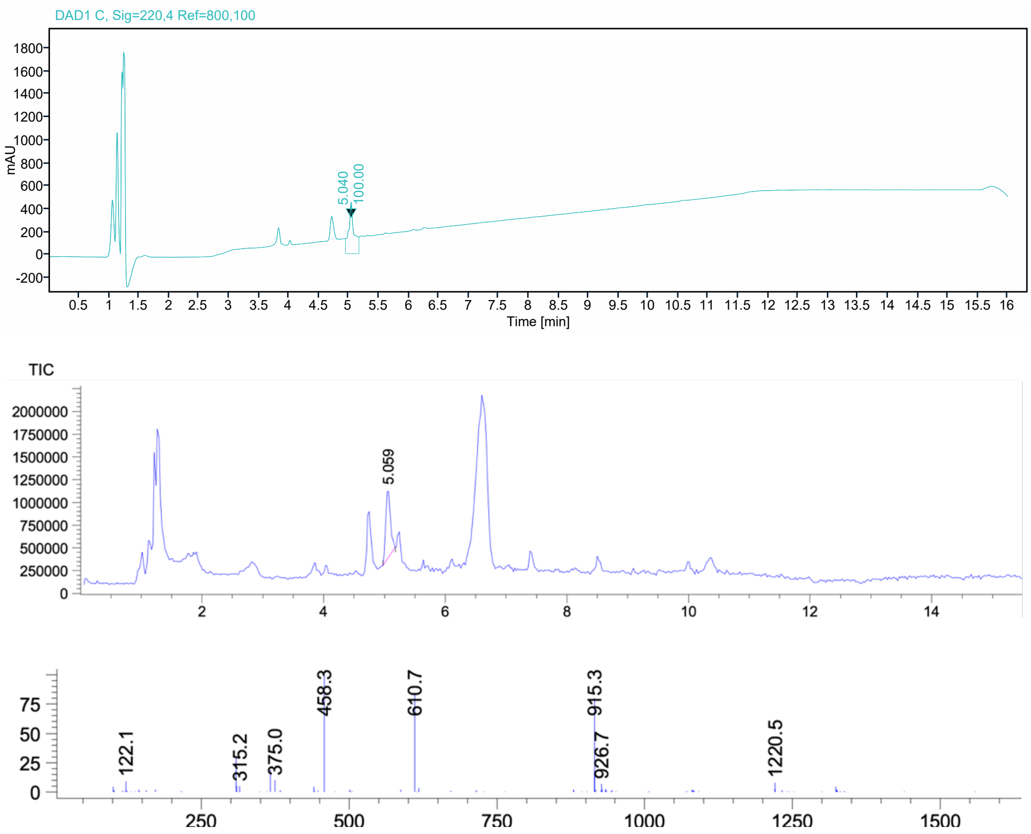

Figure S19: LC-MS chromatograms and ESI$^+$ mass spectrum (t_R = 5.059 min) from the cell permeability assay for PDC5 (compound **51**) (*n* = 2). PDC5 exact mass = 1827.95 (observed masses = 915.3, $[M-5TFA+2H]^{2+}$; 610.7, $[M-5TFA+3H]^{3+}$; 458.3, $[M-5TFA+4H]^{4+}$).

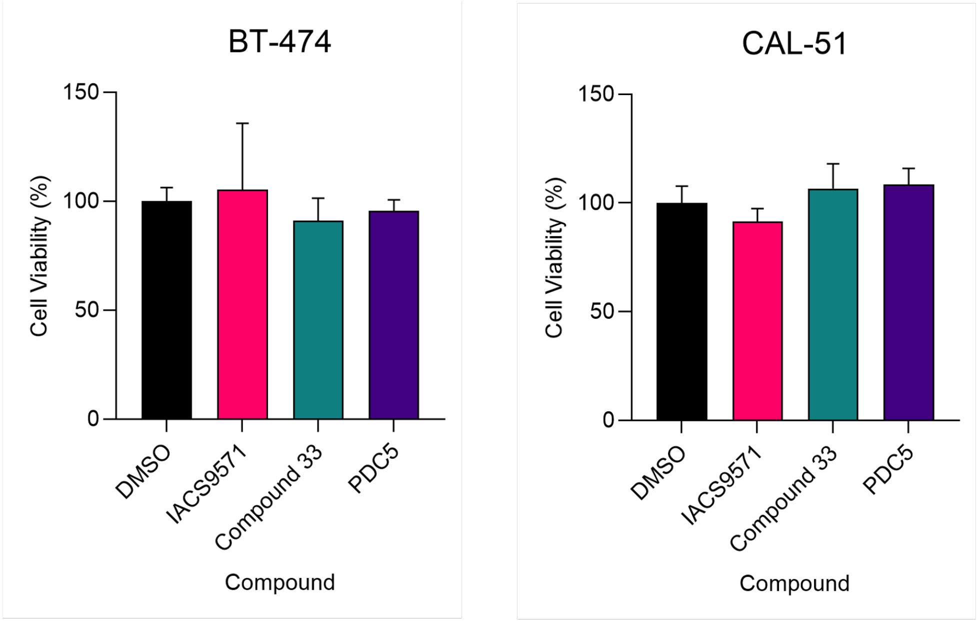

Figure S20: Bar graphs showing the results of the MTT assay for two breast cancer cell lines, BT-474 (ER^+) and CAL-51 (triple negative), treated with the indicated compound (10 μM). The data are represented as the mean of means for two biological repeats (*n* = 2), each consisting of three technical repeats. Error bars indicate the standard error of the mean. BT-474 was used in place of MCF-7 cells due to issues with procurement at the time of running the assay.

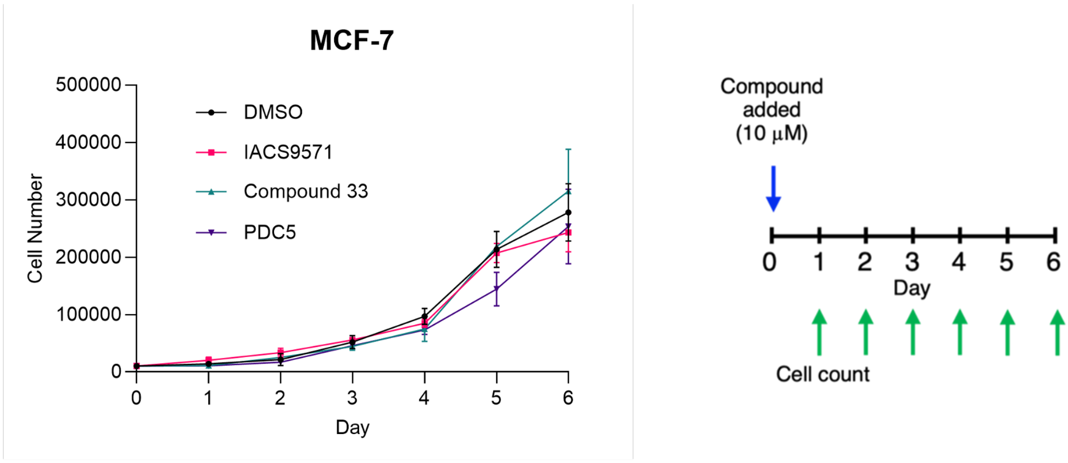

Figure S21: Graph showing the results of the proliferation assay for MCF-7 cells treated with the indicated compound (10 μM). Compounds were added after seeding on day 0 only and counting was carried out each day for 6 days. Data are represented as the mean of triplicate data from one biological repeat. Error bars indicate the standard deviation.

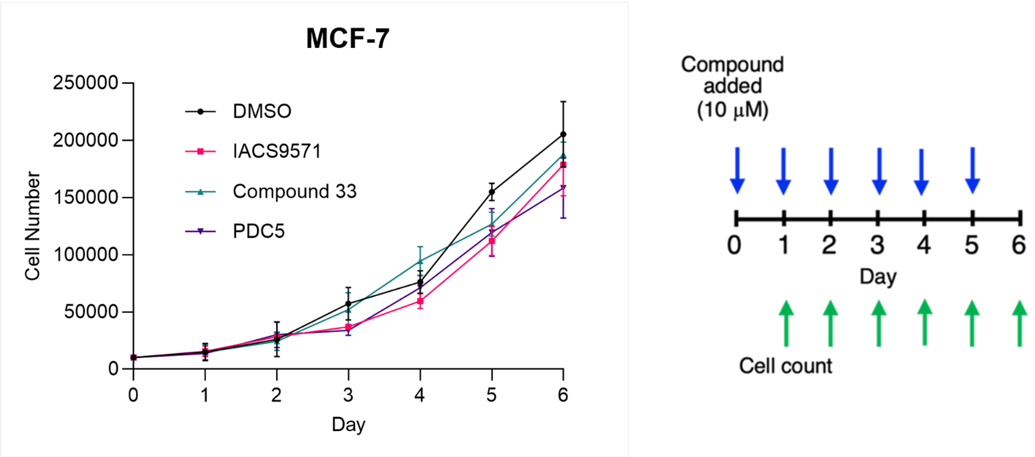

Figure S22: Graph showing the results of the proliferation assay for MCF-7 cells repeatedly treated with the indicated compound (10 μM) over 6 days. Compound was added each day for 6 days and counting was carried out prior to addition of compound on the following day. Data are represented as the mean of triplicate data from one biological repeat. Error bars indicate the standard deviation.

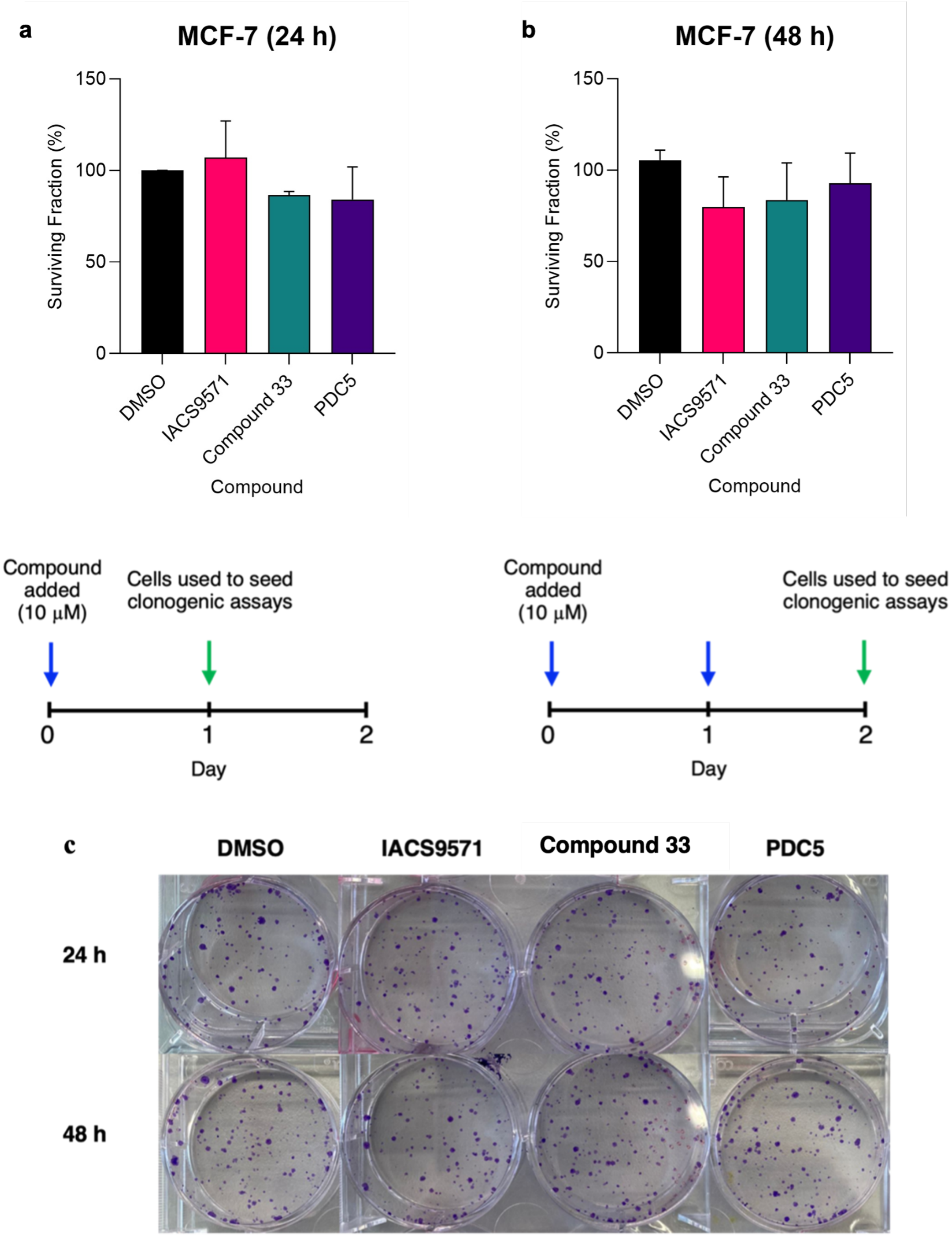

Figure S23: a) Bar graph showing the results of the single dose clonogenic assay for MCF-7 cells after treatment with the indicated compound (10 μM). Cells were treated with compound on day 0 only and after 24 h were used to seed plates for the clonogenic assay; b) Bar graph showing the results of the double dose clonogenic assay for MCF-7 cells after treatment with the indicated compound (10 μM). Cells were treated with compound on day 0 and day 1; and after a total of 48 h were used to seed plates for the clonogenic assay. The data are represented as the mean of means for two biological repeats (*n* = 2), each consisting of three technical replicates. Error bars indicate the standard error of the mean; c) Representative images of the colonies from the MCF-7 clonogenic assays.

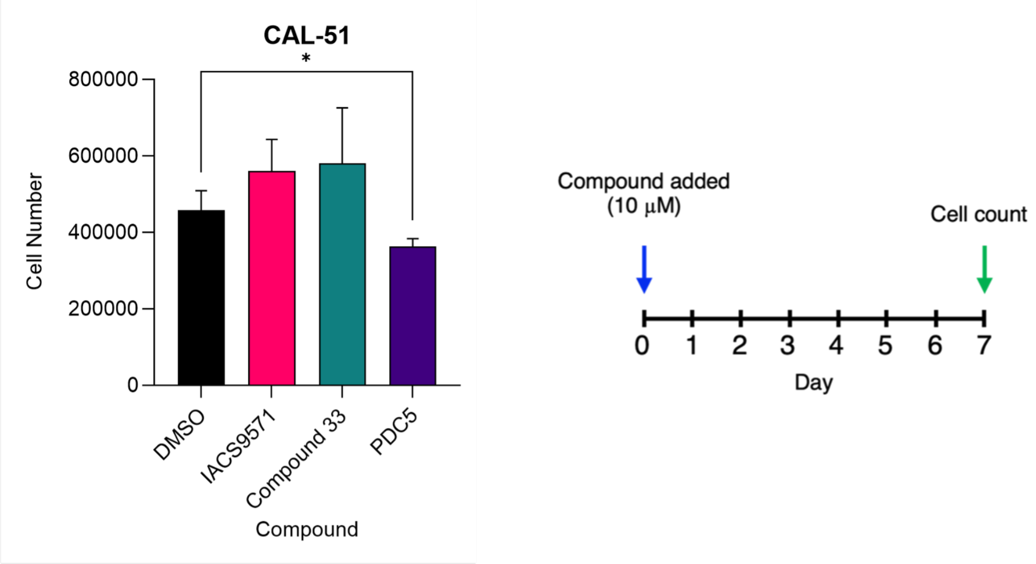

Figure S24: Bar graph showing the results of the proliferation assay for CAL-51 cells treated with the indicated compound (10 μM) for 7 days. Cells were treated with compound on day 0 only. The data are represented as the mean of triplicate data from one biological repeat, with error bars indicating the standard deviation. Statistical significance compared to the vehicle control was evaluated using an unpaired parametric t-test (*; $p \leq 0.05$).

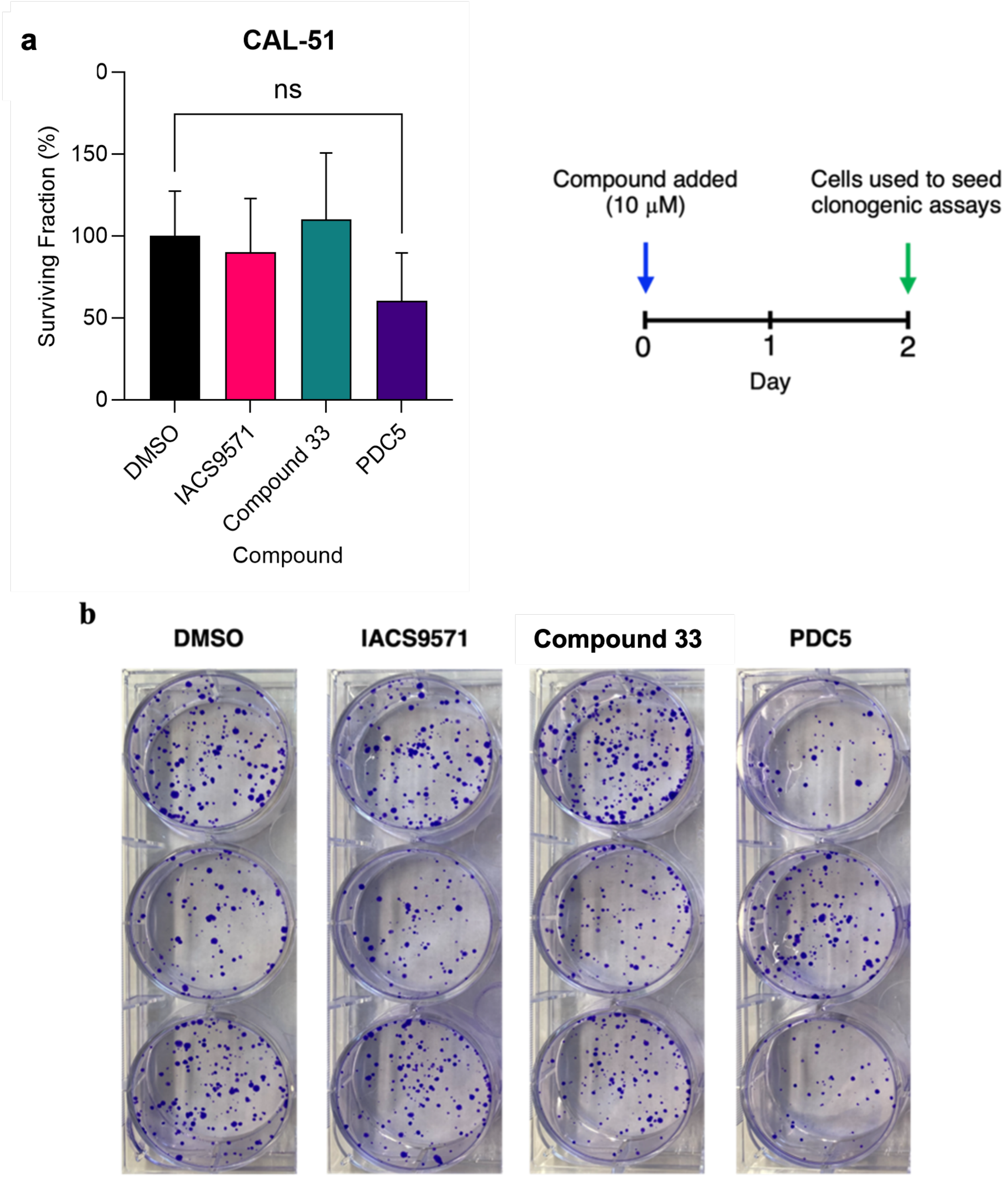

Figure S25: a) Bar graph showing the results of the clonogenic assay for CAL-51 cells after treatment with the indicated compound (10 μM) for 2 days. Cells were treated with compound on day 0 only and after 2 days were used to seed plates for the clonogenic assay. The data are represented as the mean of triplicate data from one biological repeat with error bars indicating the standard deviation. Statistical significance compared to the vehicle control was evaluated using an unpaired parametric t-test (ns; $p > 0.05$); b) Images of the colonies from the CAL-51 clonogenic assay. Each horizontal line of plates represents a technical replicate.

Figure S26: AlphaScreen® dose-response curves for the inhibition of TRIM24-H3K9Me3 (B1) binding by H3K9Me3-mimicking peptides **P1**–**P16**. $H3_{(1-27)}K9Me_3$ and $H3_{(1-15)}K4Me_3$ are the positive and negative controls, respectively. Dose-response curves were obtained in triplicate with error bars indicating the standard deviation.

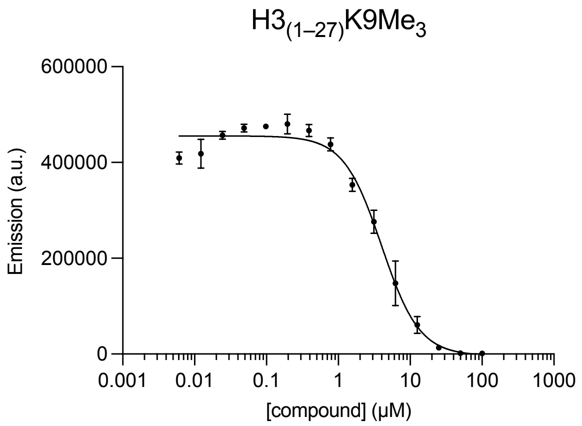

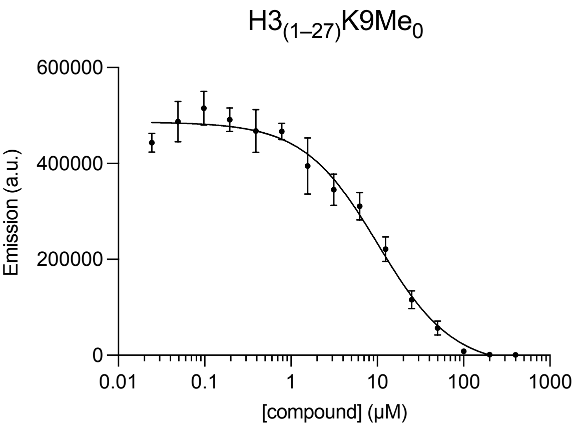

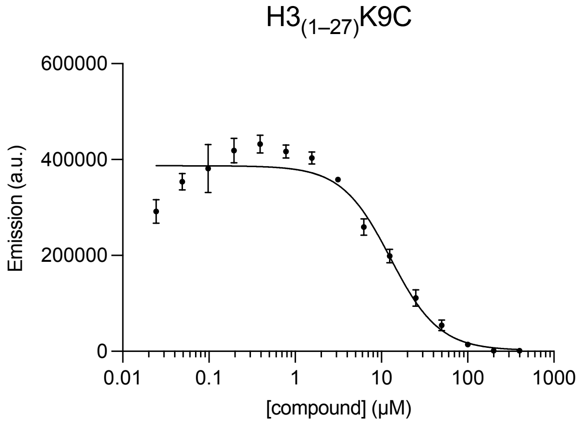

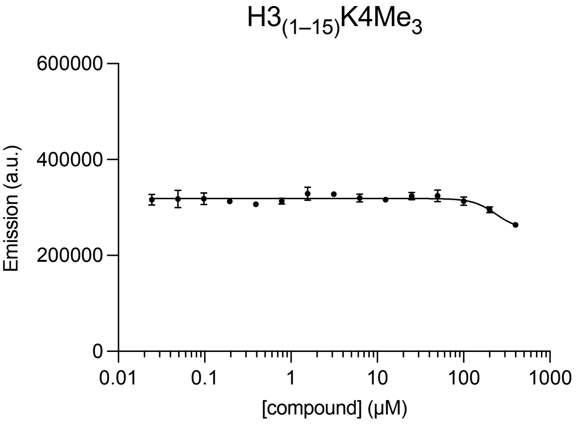

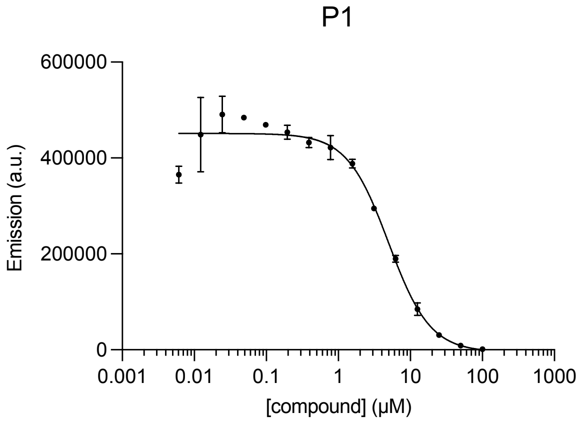

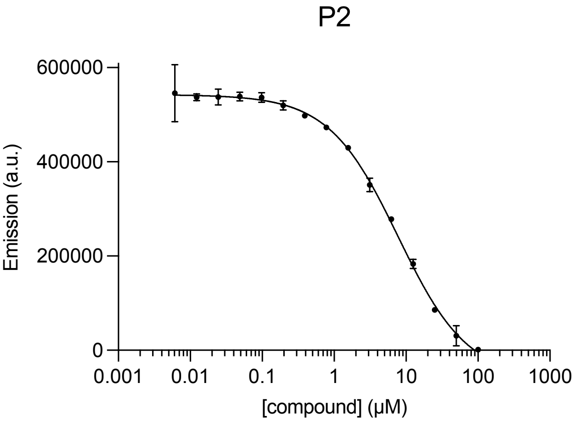

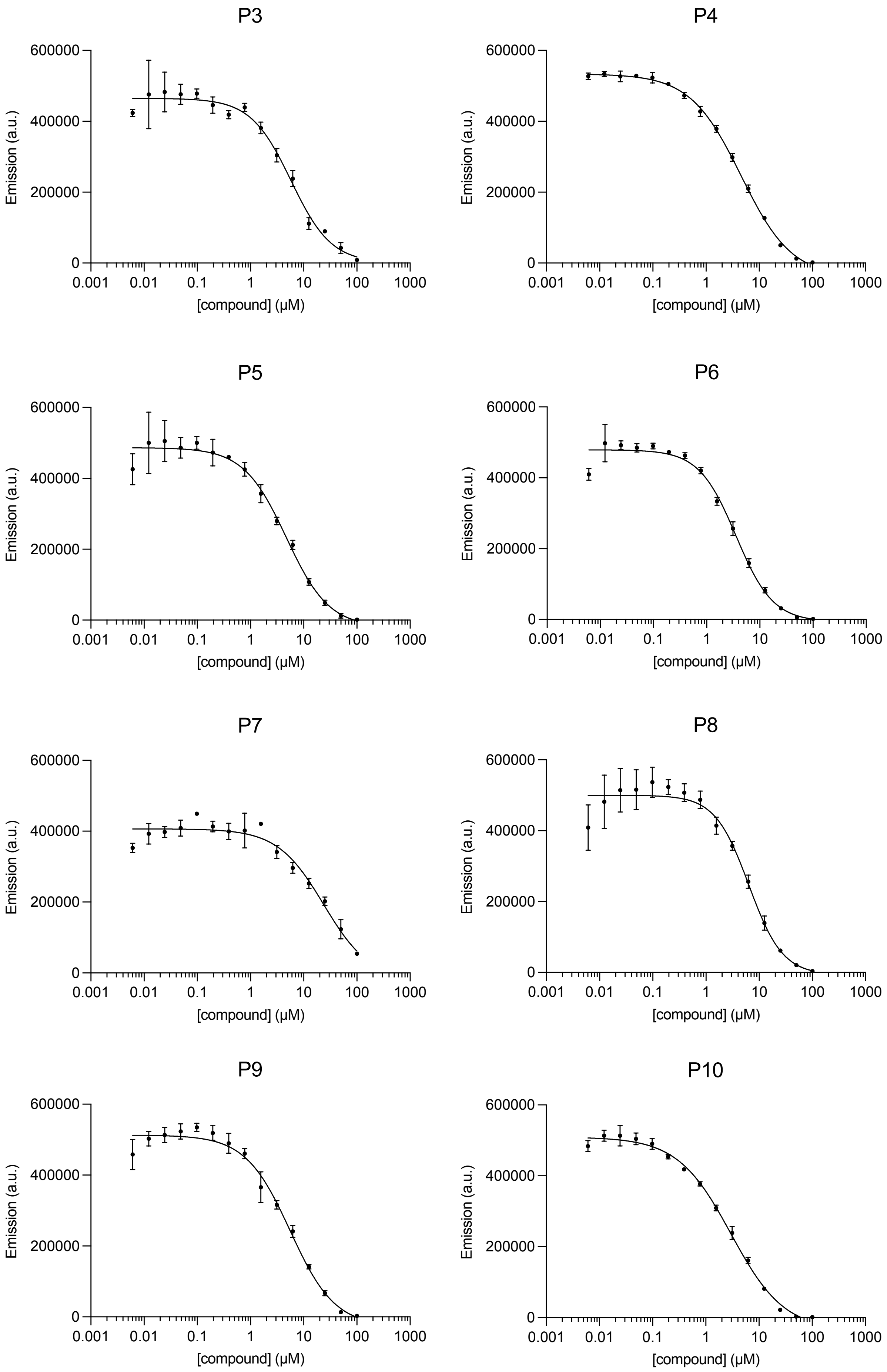
P3
P4
P5
P6
P7
P8
P9
P10
Emission (a.u.)
600000
400000
200000
0
0.001
0.01
0.1
1
10
100
1000
[compound] (µM)

P11

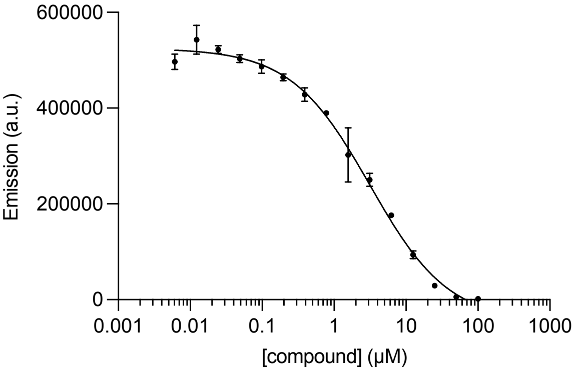

P12

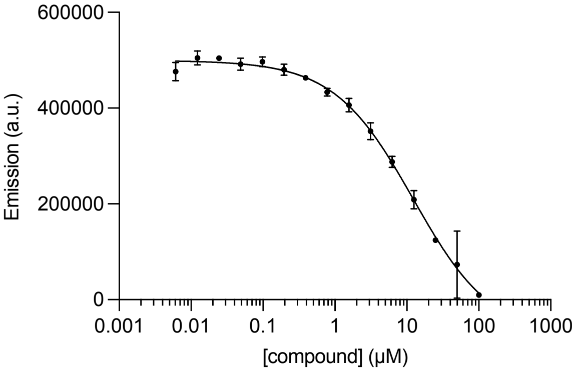

P13

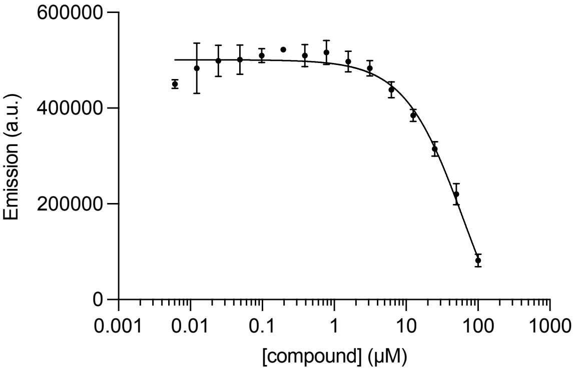

P14

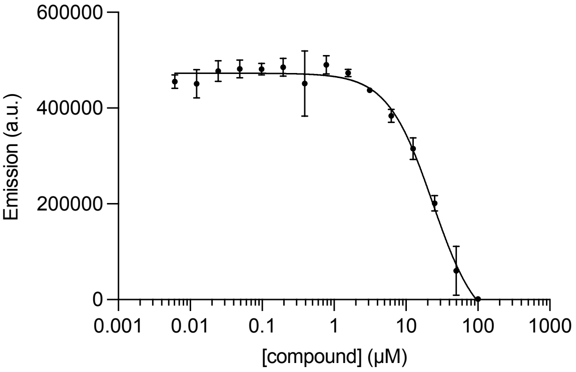

P15

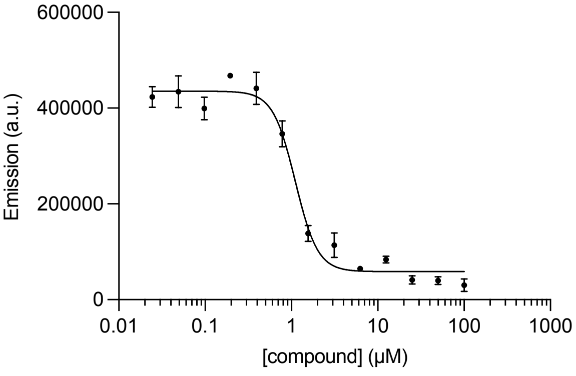

P16

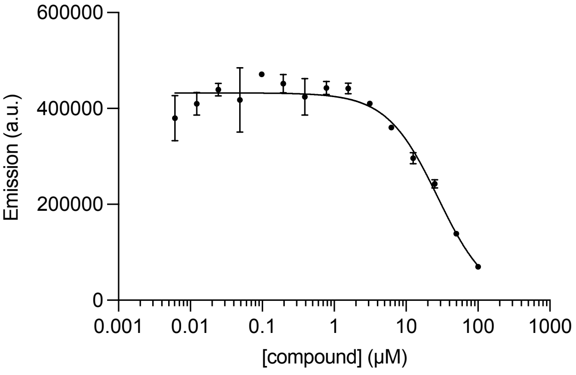

Figure S27: AlphaScreen® dose-response curves for the inhibition of TRIM24-H3K9Me₃ (B1) binding by the PDCs and control compounds. $H3_{(1\text{–}27)}K9Me_3$ and $H3_{(1\text{–}15)}K4Me_3$ are the positive and negative controls, respectively. Dose-response curves were obtained in triplicate with error bars indicating the standard deviation.

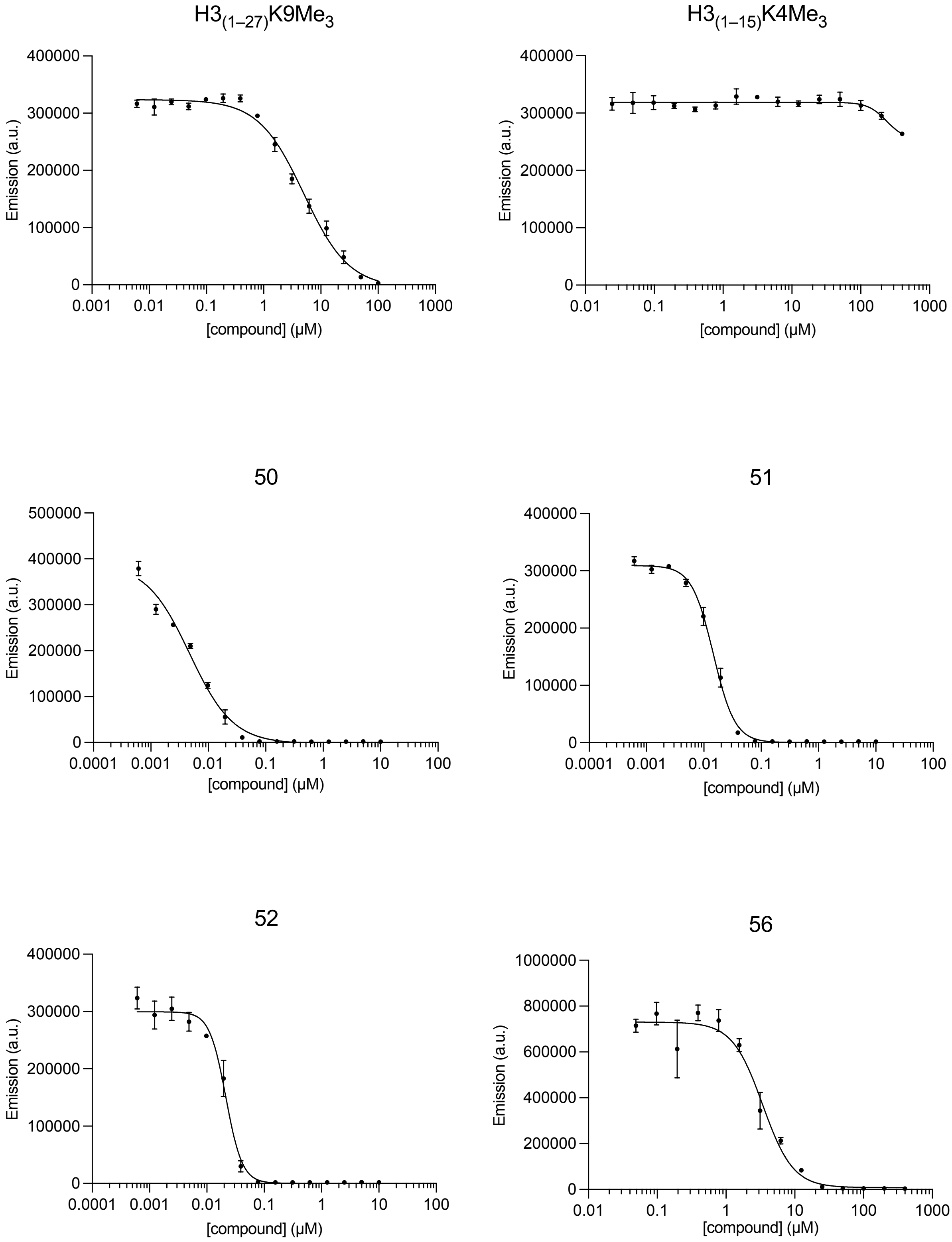

Figure S28: AlphaScreen® dose-response curves for the inhibition of TRIM24-H3K18Ac (B2) binding by the PDCs and control compounds. $H3_{(1-27)}K18Ac$ and $H3_{(1-15)}K4Me_3$ are the positive and negative controls, respectively. Dose-response curves were obtained in triplicate with error bars indicating the standard deviation.

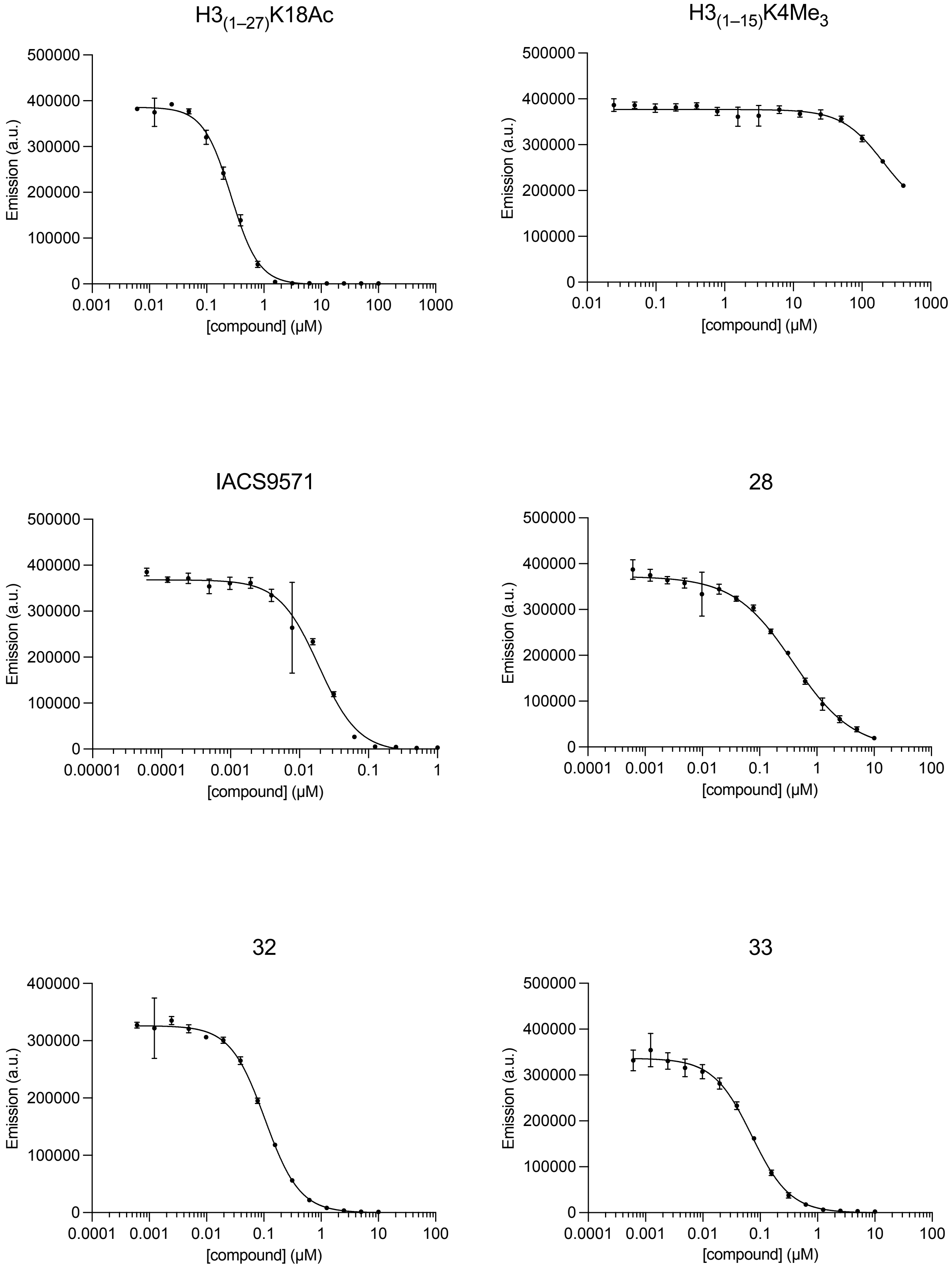

34

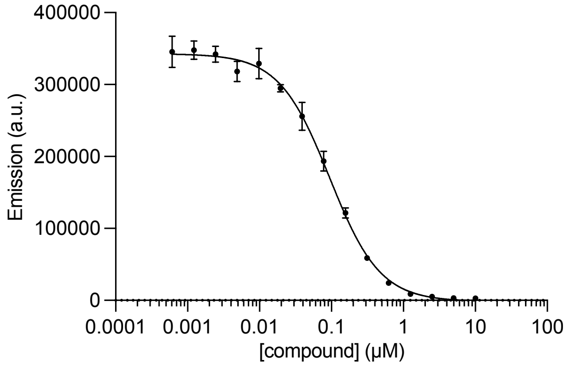
400000
300000
200000
100000
0
Emission (a.u.)
0.0001
0.001
0.01
0.1
1
10
100
[compound] (µM)

50

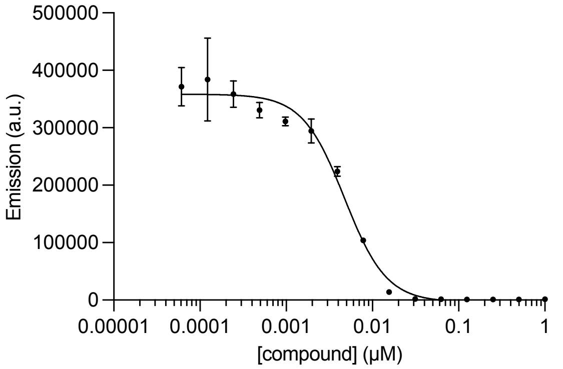
500000
400000
300000
200000
100000
0
Emission (a.u.)
0.00001
0.0001
0.001
0.01
0.1
1
[compound] (µM)

51

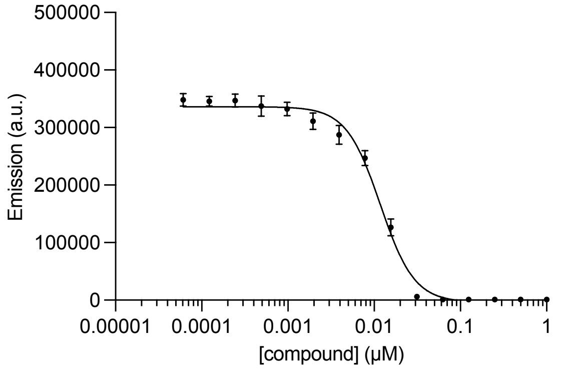
500000
400000
300000
200000
100000
0
Emission (a.u.)
0.00001
0.0001
0.001
0.01
0.1
1
[compound] (µM)

52

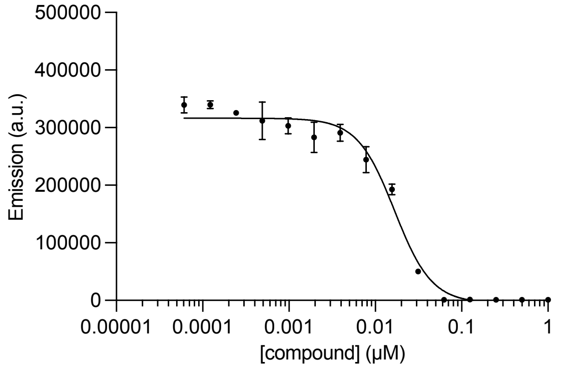
500000
400000
300000
200000
100000
0
Emission (a.u.)
0.00001
0.0001
0.001
0.01
0.1
1
[compound] (µM)

Figure S29: AlphaScreen® dose-response curves for the inhibition of TRIM24-H3K9Me3K18Ac (B3) binding by the PDCs and control compounds. $H3_{(1-27)}K9Me_3K18Ac$ and $H3_{(1-15)}K4Me_3$ are the positive and negative controls, respectively. Dose-response curves were obtained in triplicate with error bars indicating the standard deviation.

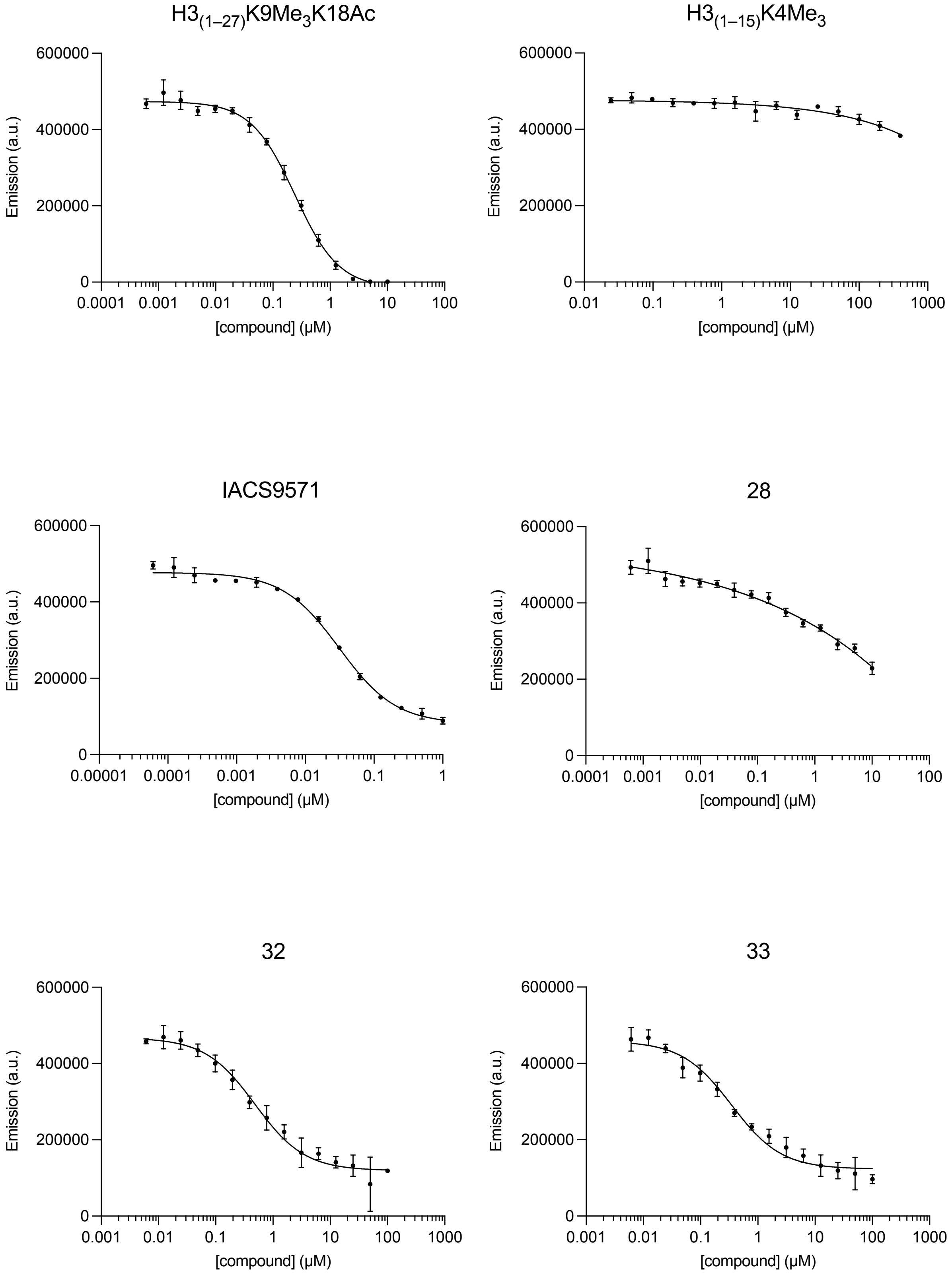

34

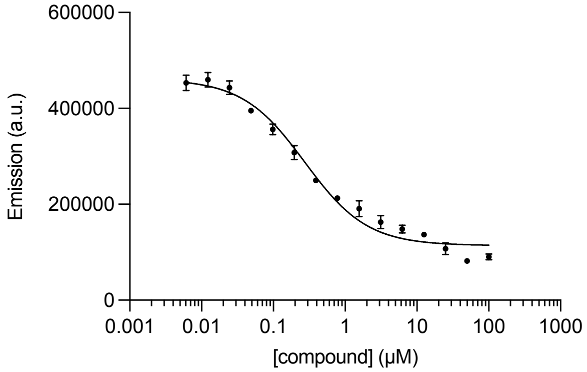

50

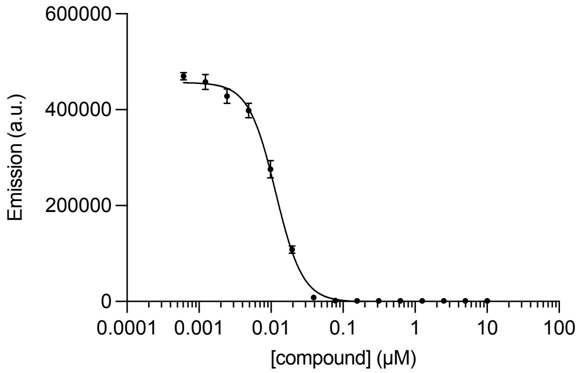

51

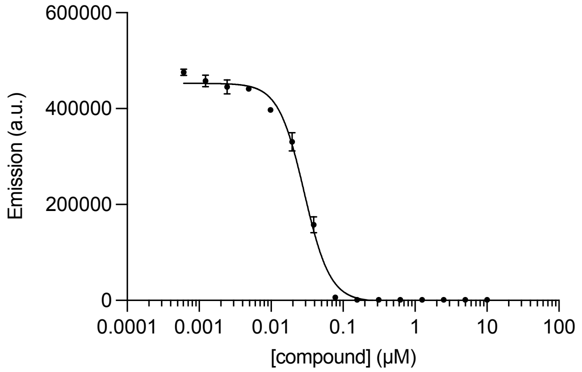

52

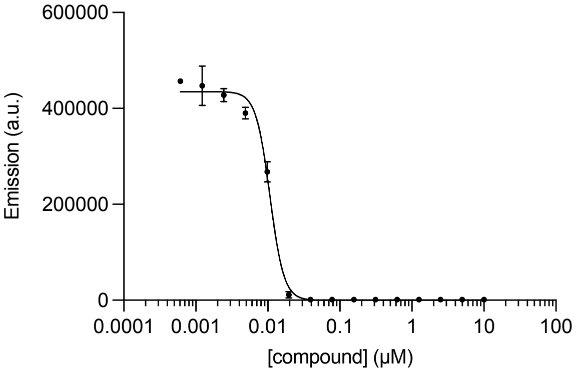

56

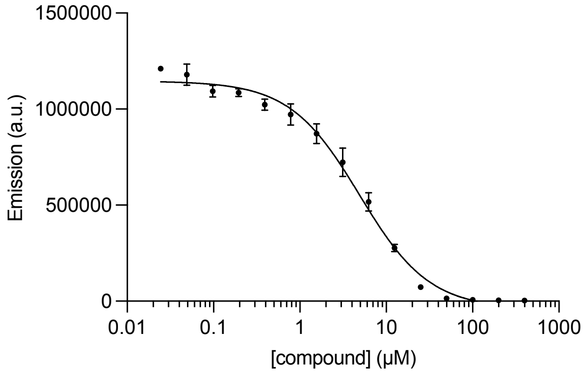

32 + 56

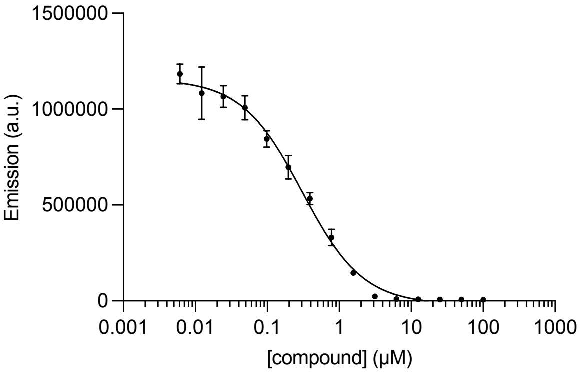

33 + 56

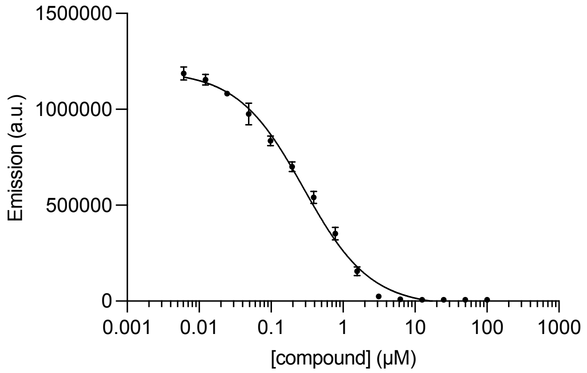

34 + 56

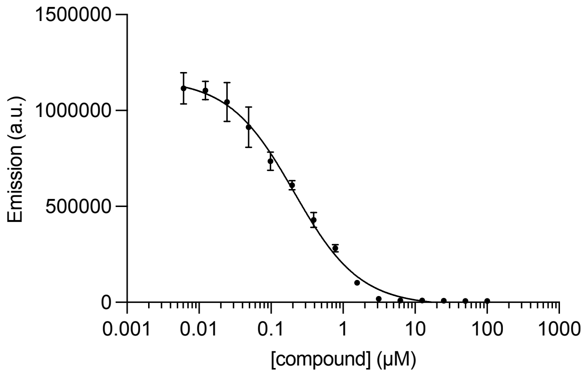

Figure S30: AlphaScreen® ternary complex assay. Dose-response curves were obtained in triplicate with error bars indicating the standard deviation.

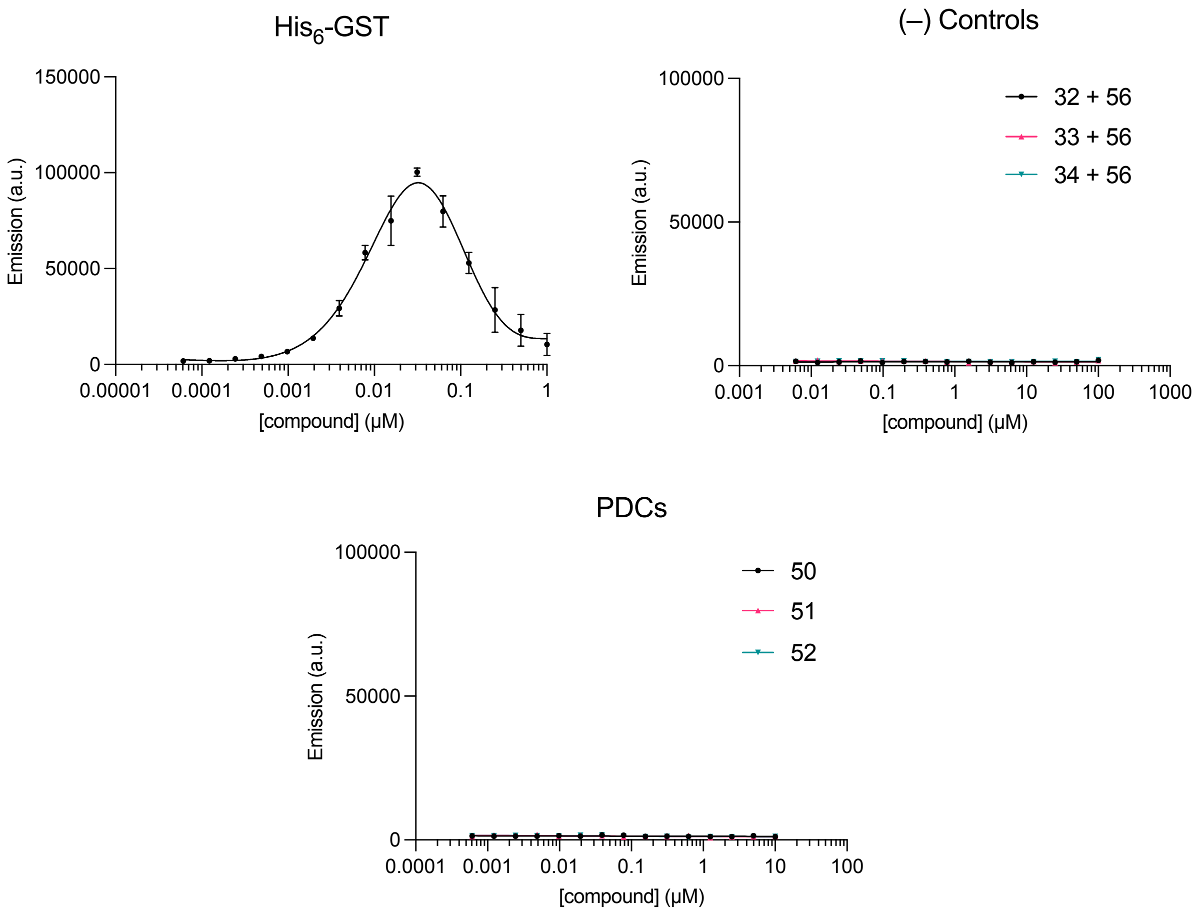

Figure S31: AlphaScreen® signal response experiments for biotinylated histone-mimicking peptides binding to His_6-TRIM24. Dose-response curves were obtained in triplicate with error bars indicating the standard deviation.

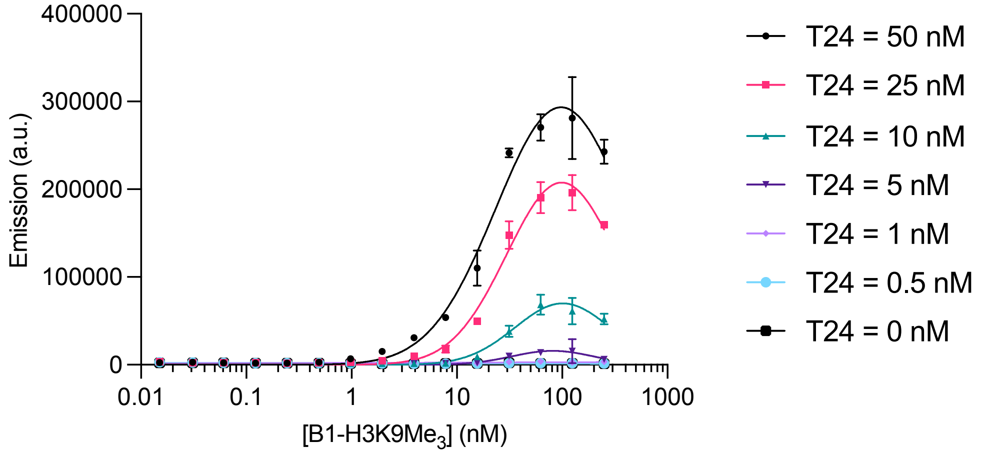

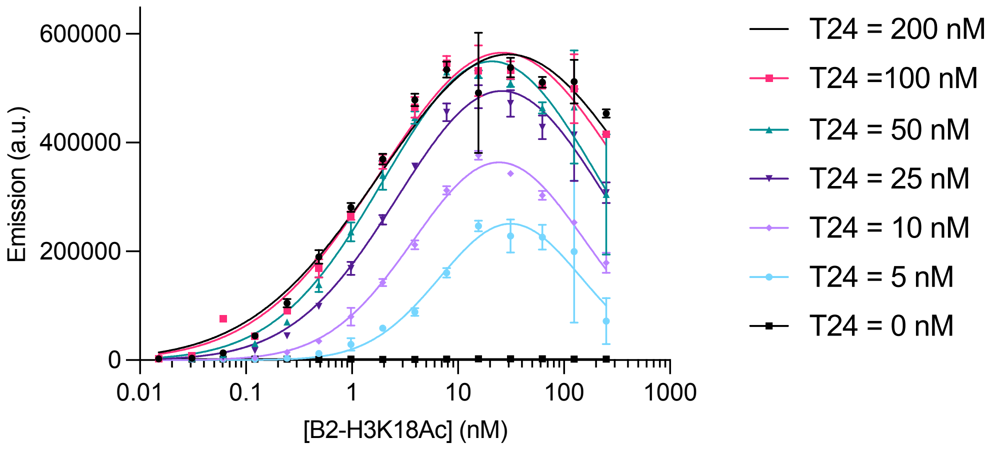

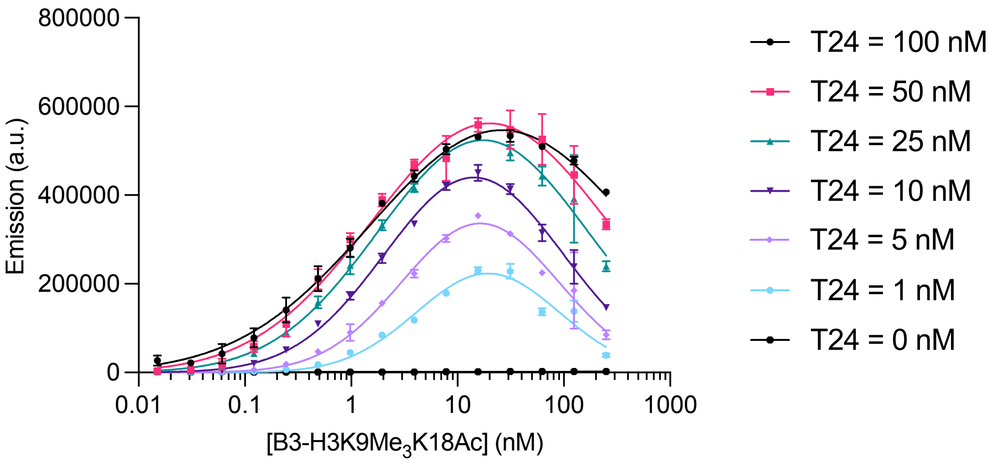

Figure S32: AlphaScreen® signal response experiments for biotinylated histone-mimicking peptides binding to GST-TRIM24. Dose-response curves were obtained in triplicate with error bars indicating the standard deviation.

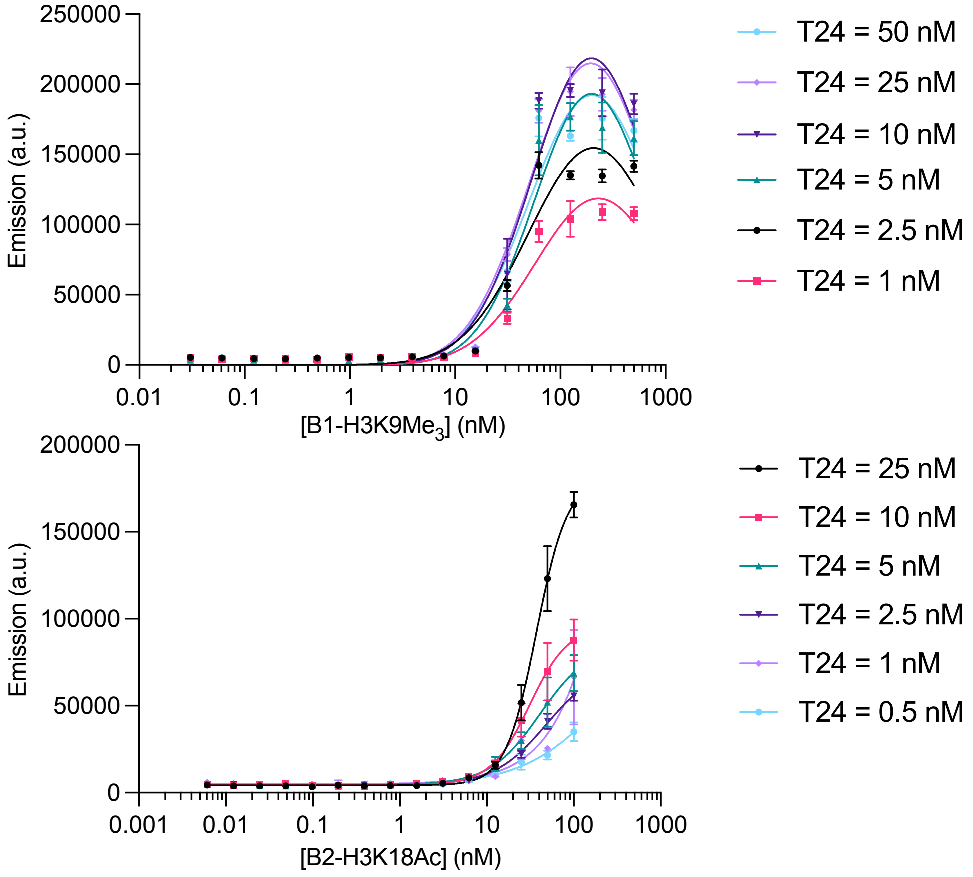

Figure S33: BROMOscan dose-response curves for binding to BRPF1. Curves produced by Eurofins DiscoverX Corporation (San Diego, USA).

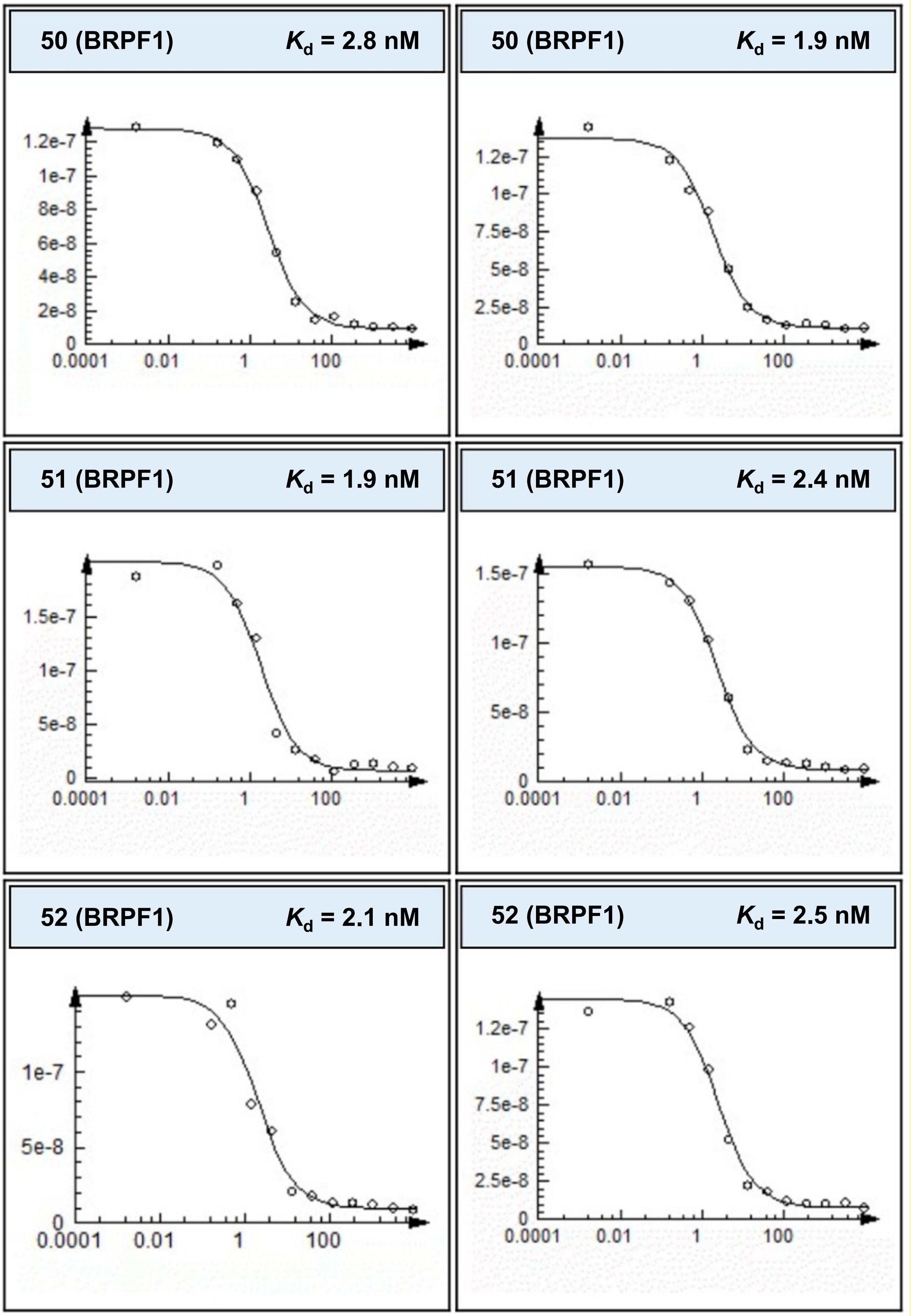

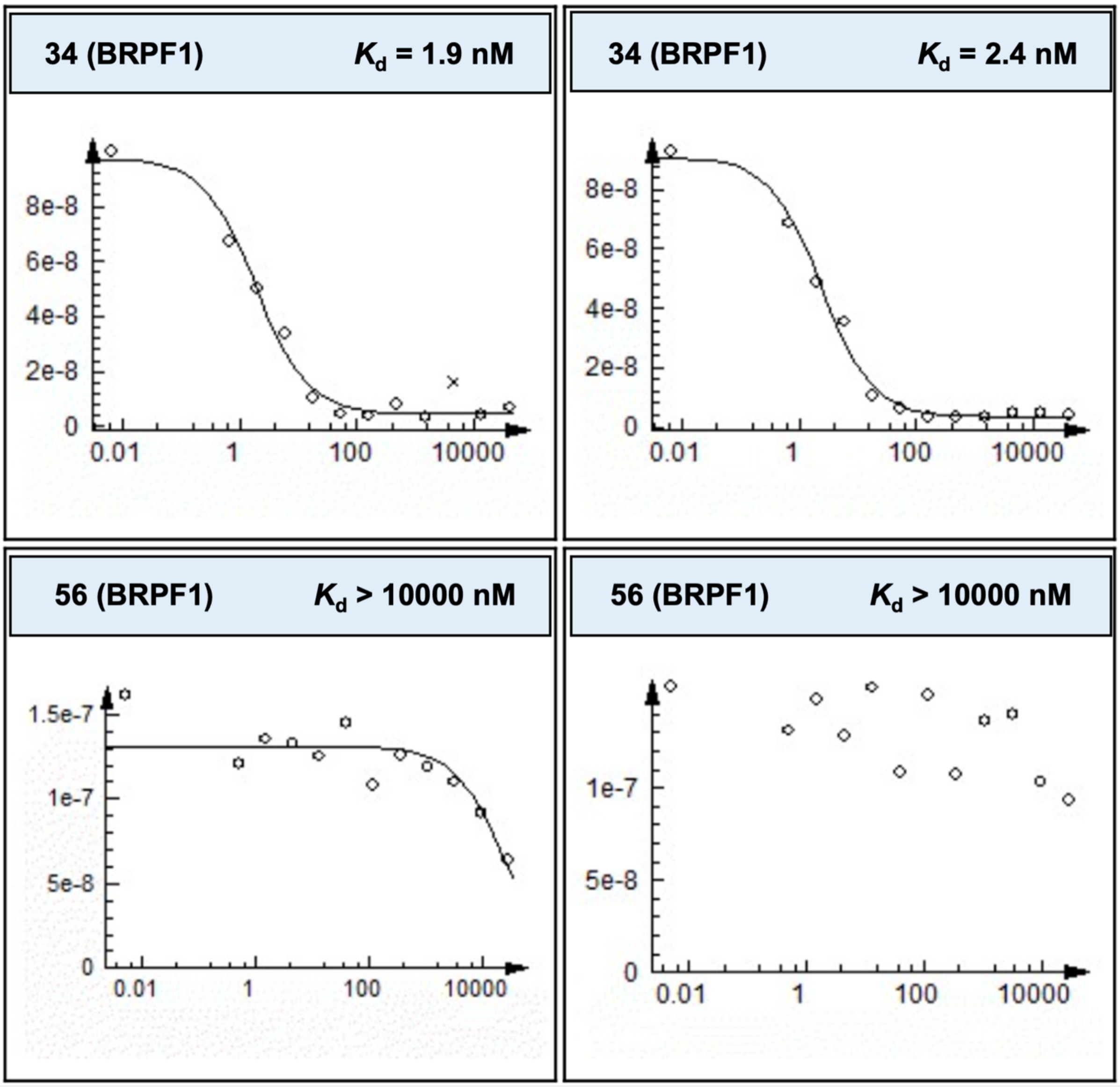
34 (BRPF1)
K_d = 1.9 nM
8e-8
6e-8
4e-8
2e-8
0
0.01
1
100
10000
34 (BRPF1)
K_d = 2.4 nM
8e-8
6e-8
4e-8
2e-8
0
0.01
1
100
10000
56 (BRPF1)
K_d > 10000 nM
1.5e-7
1e-7
5e-8
0
0.01
1
100
10000
56 (BRPF1)
K_d > 10000 nM
1e-7
5e-8
0
0.01
1
100
10000

Figure S34: BROMOscan dose-response curves for binding to TRIM24 (PHD-BRD). Curves produced by Eurofins DiscoverX Corporation (San Diego, USA).

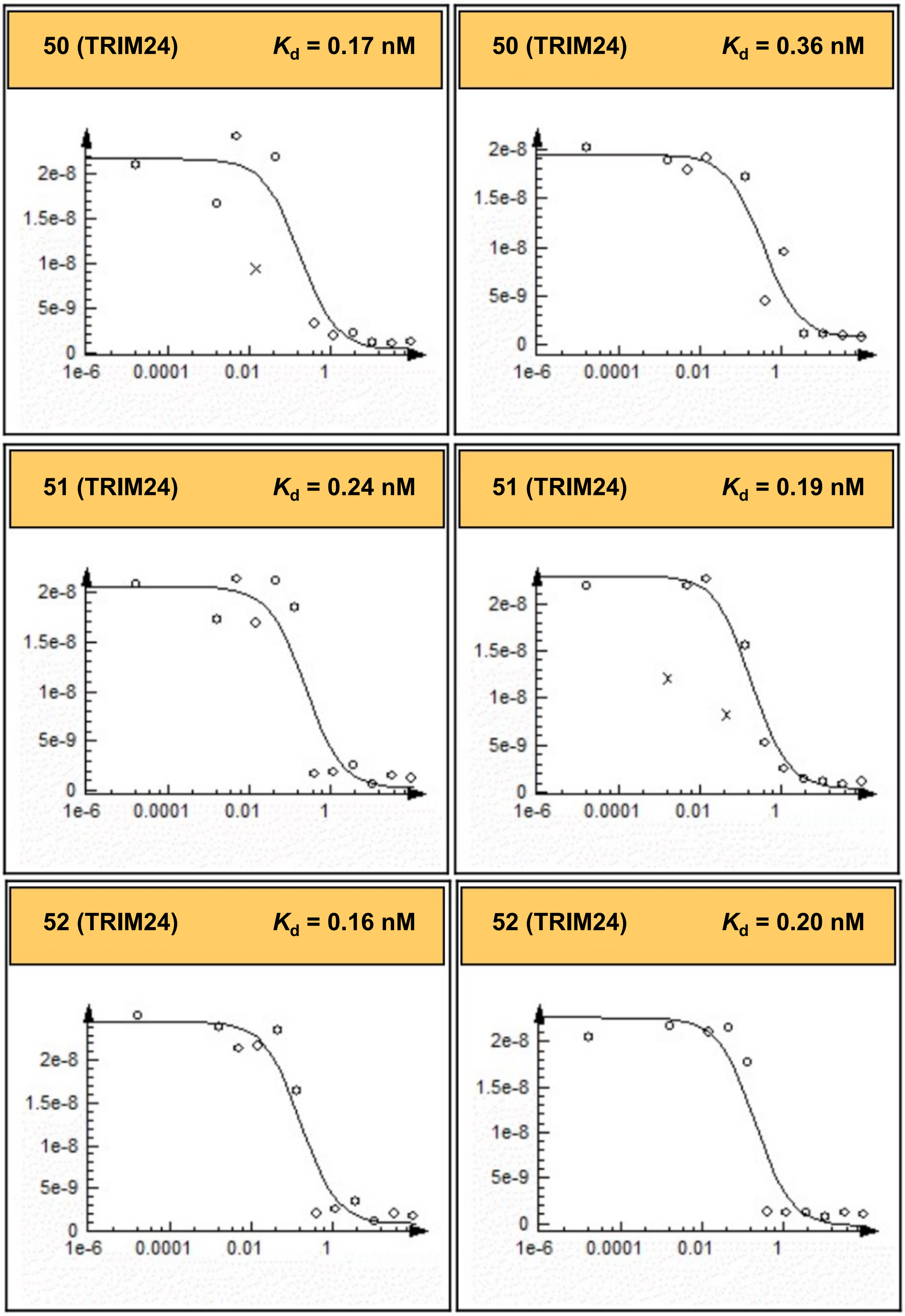

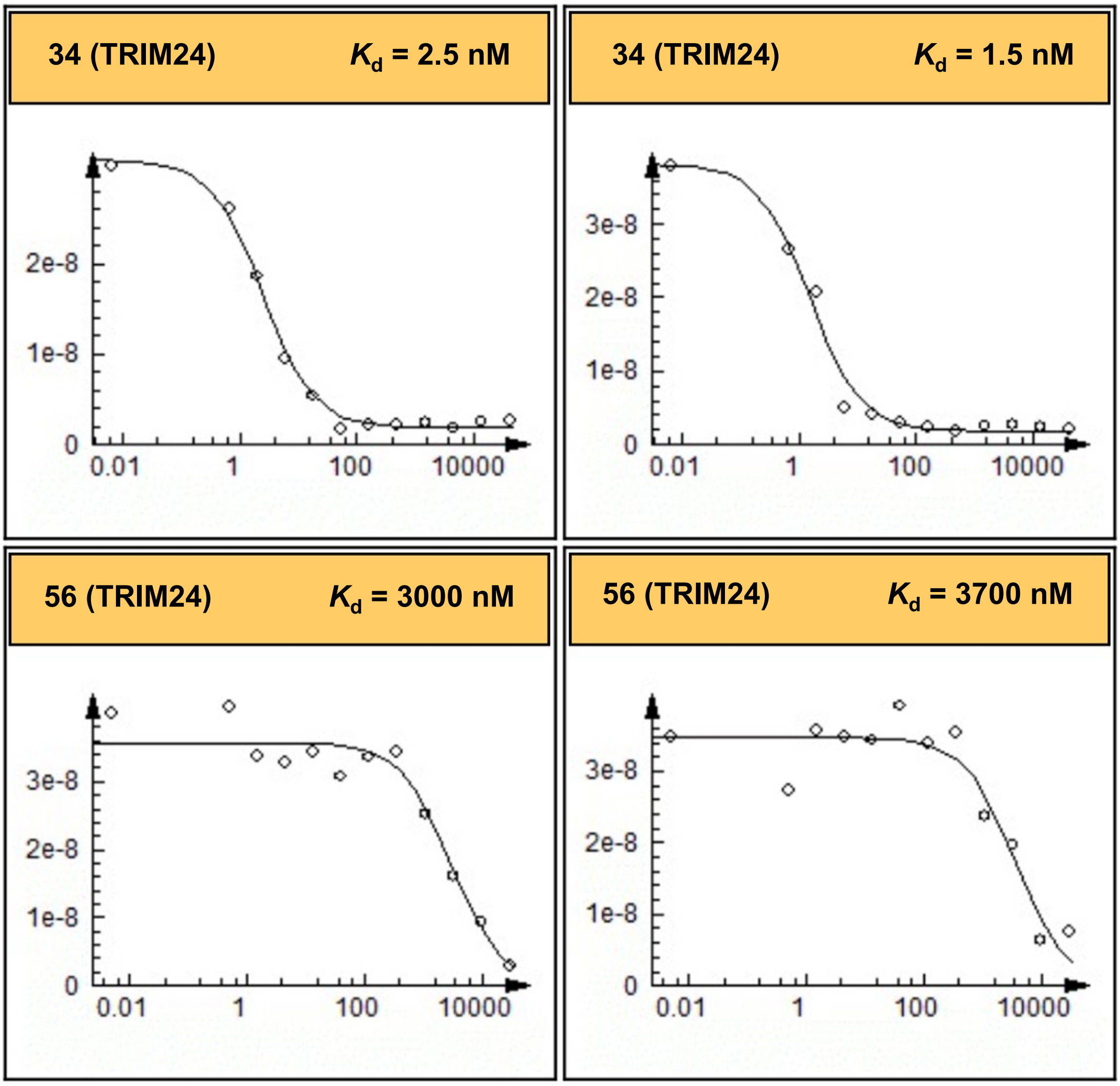
34 (TRIM24)
K_d = 2.5 nM
34 (TRIM24)
K_d = 1.5 nM
56 (TRIM24)
K_d = 3000 nM
56 (TRIM24)
K_d = 3700 nM
3e-8
2e-8
1e-8
0
0.01
1
100
10000

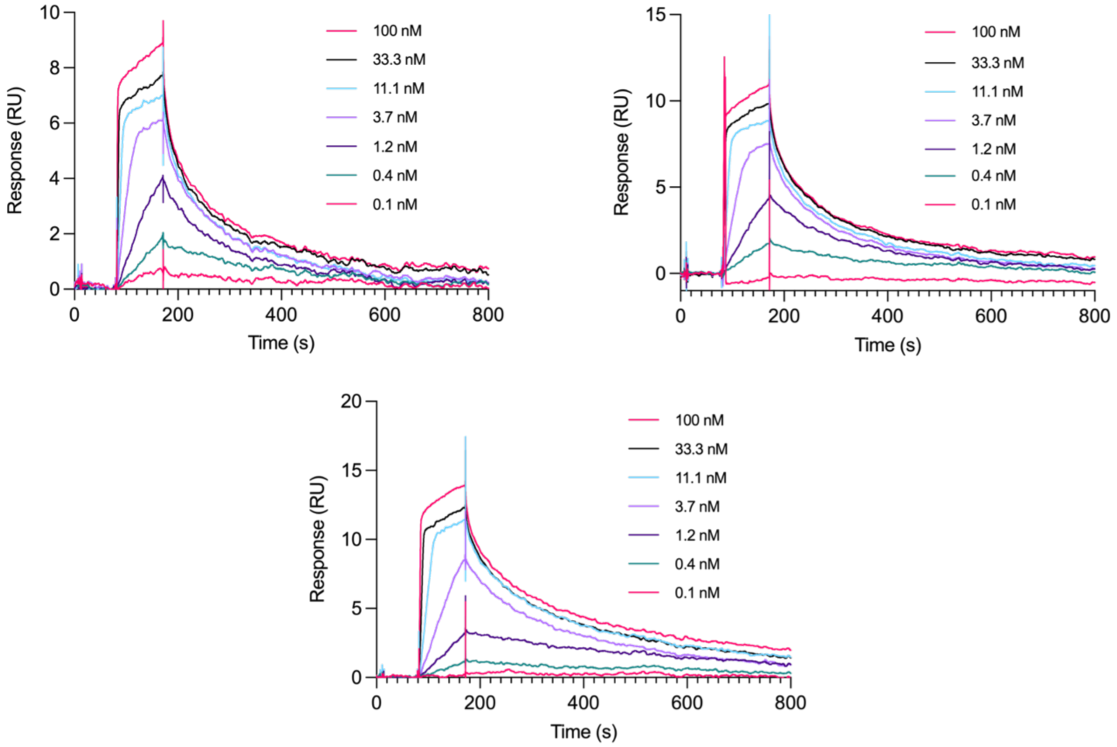

Figure S35: Raw sensogram data for PDC6 (52) binding to TRIM24 (PHD-BRD). Sensograms are reference cell subtracted.

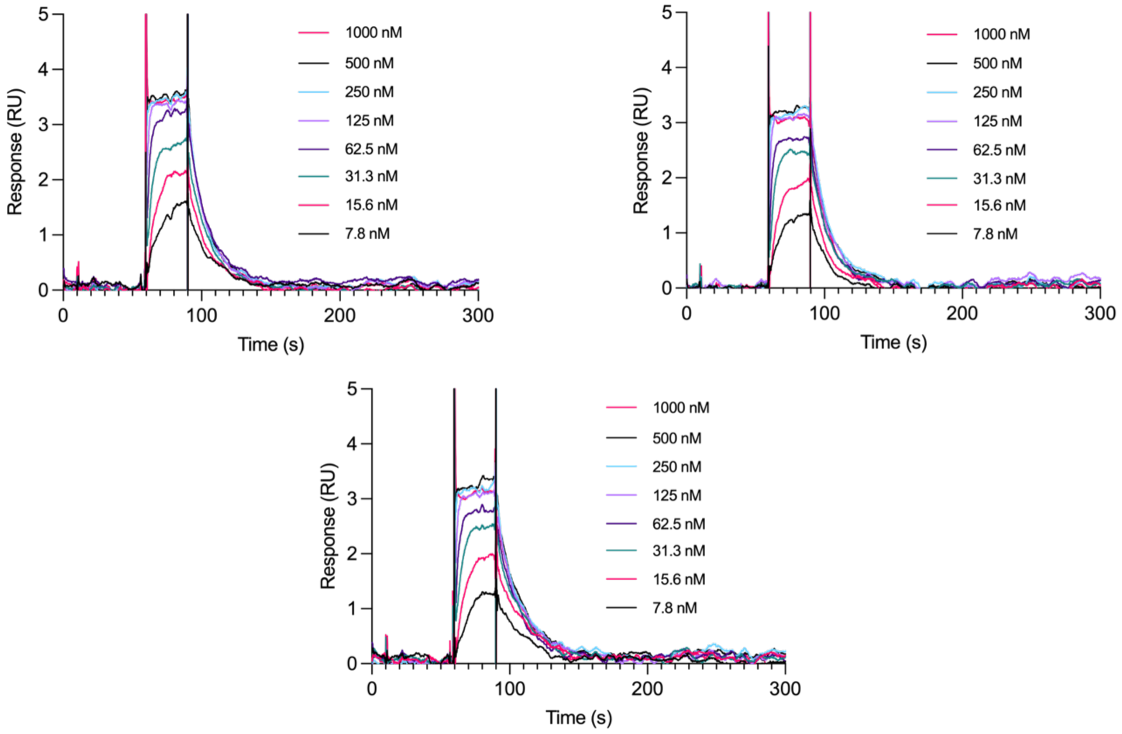

Figure S36: Raw sensogram data for TRIM24 BRD ligand 34 binding to TRIM24 (PHD-BRD). Sensograms are reference cell subtracted.

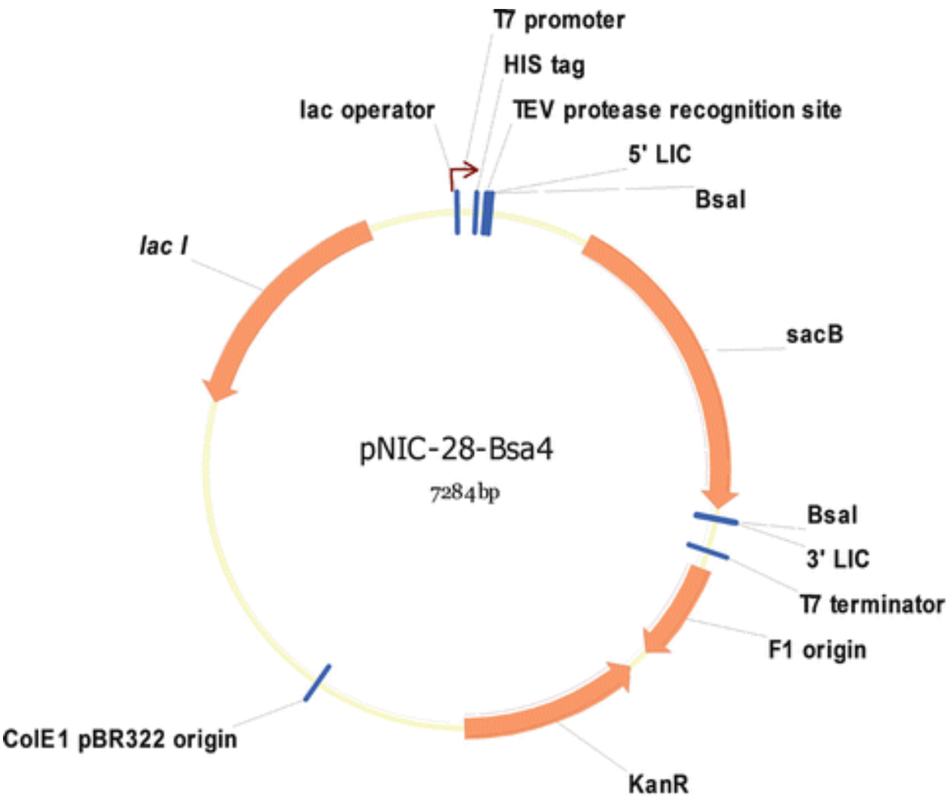

Figure S37: Plasmid map for pNIC-28-Bsa4

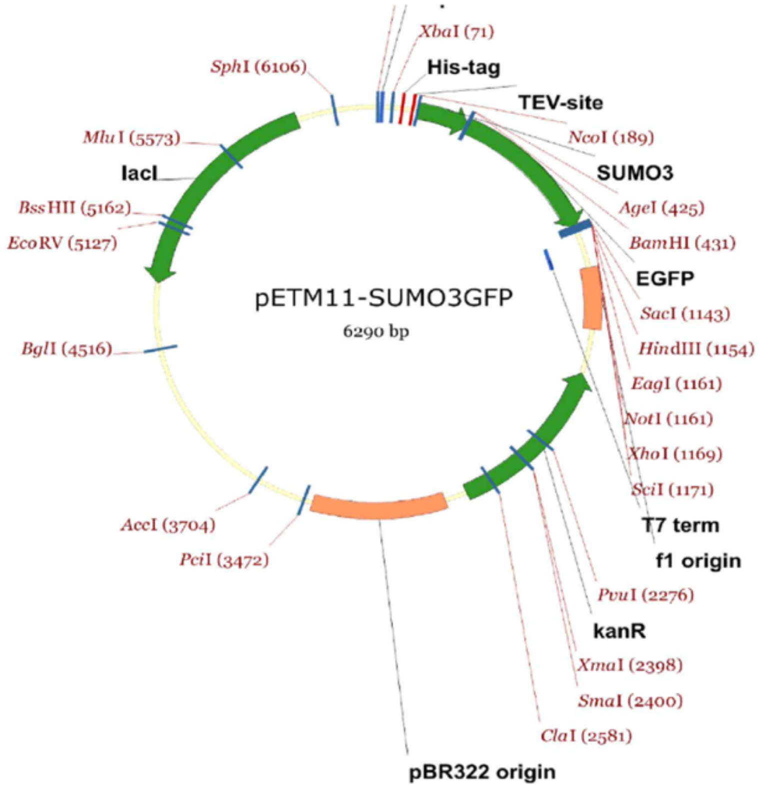

Figure S38: Plasmid map for pETM11-SUMO3GFP.

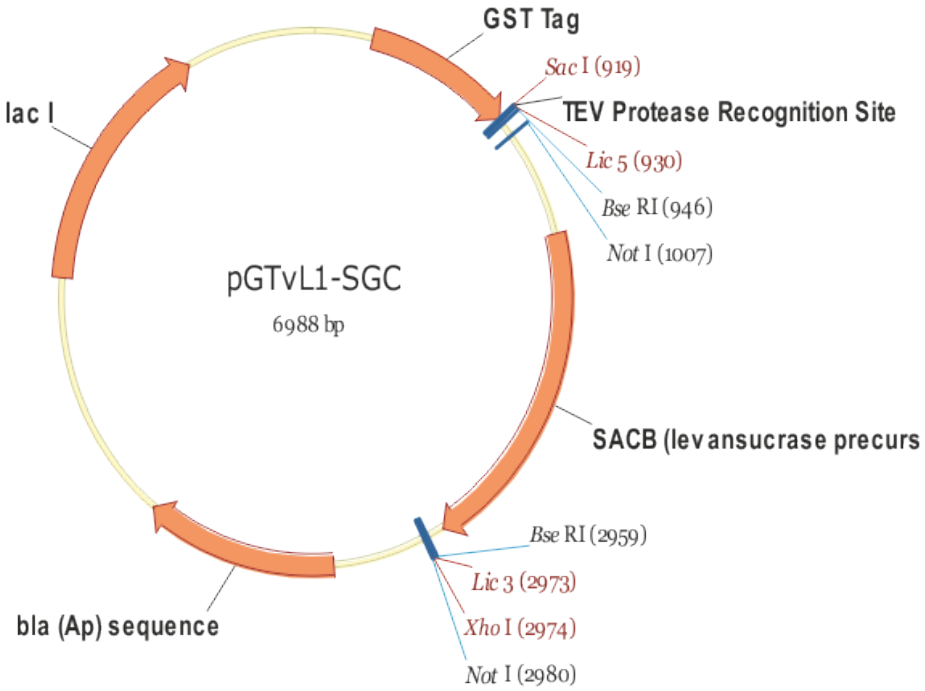

Figure S39: Plasmid map for pGTvL1-SGC.

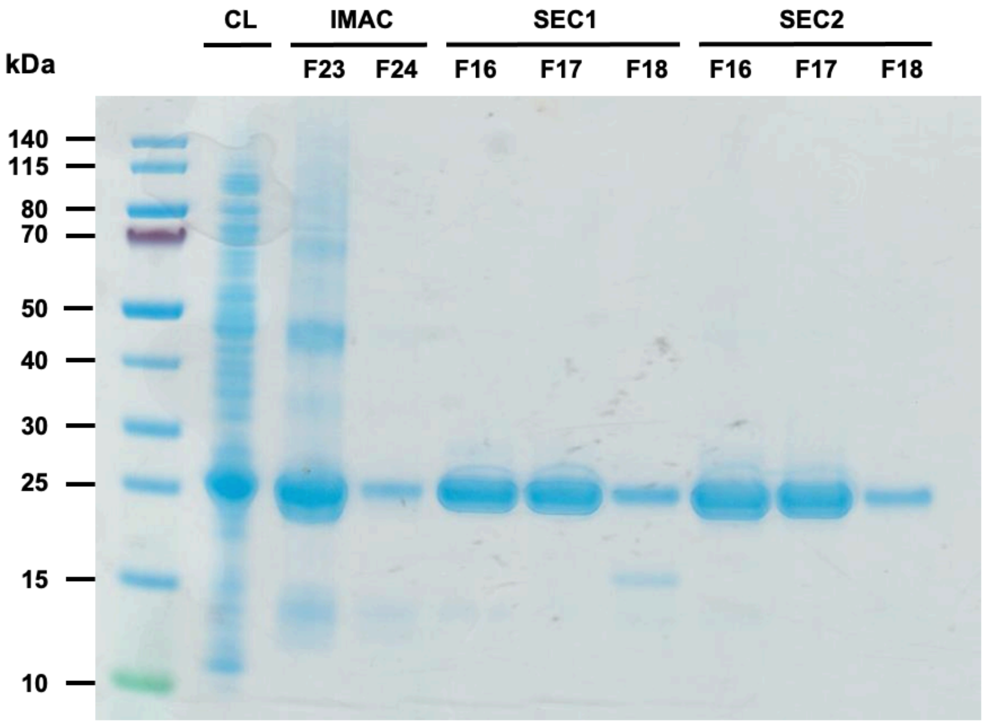

Figure S40: SDS-PAGE gel for the purification of His_6-TRIM24 (CL = cell lysate).

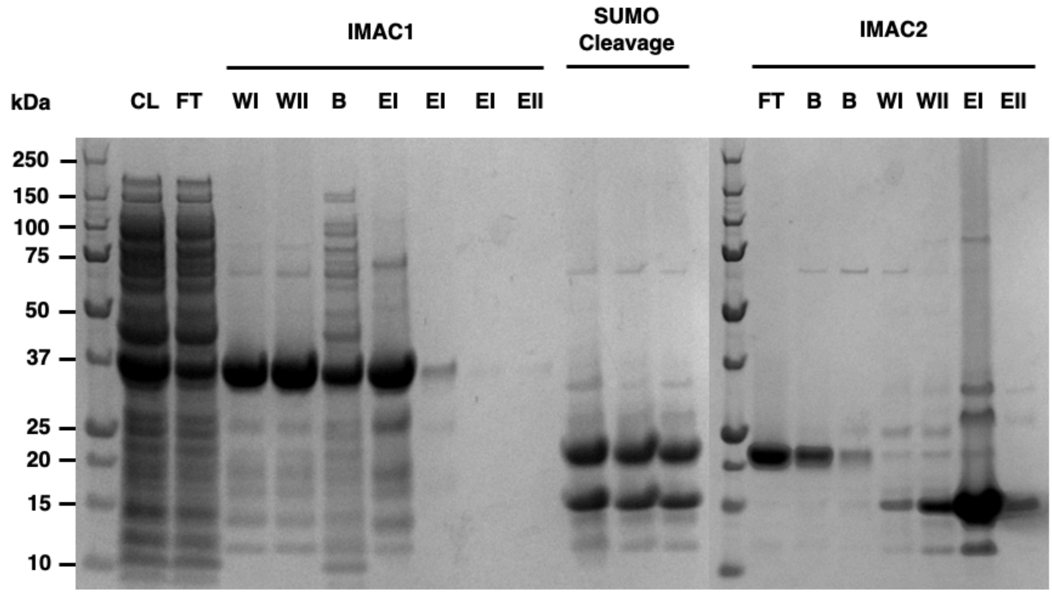

Figure S41: SDS-PAGE gels for the IMAC purification of His$_6$SUMO-TRIM24 and SUMO tag cleavage (CL, cell lysate; FT, flowthrough; W, wash buffer; B, binding buffer; E, elution buffer).

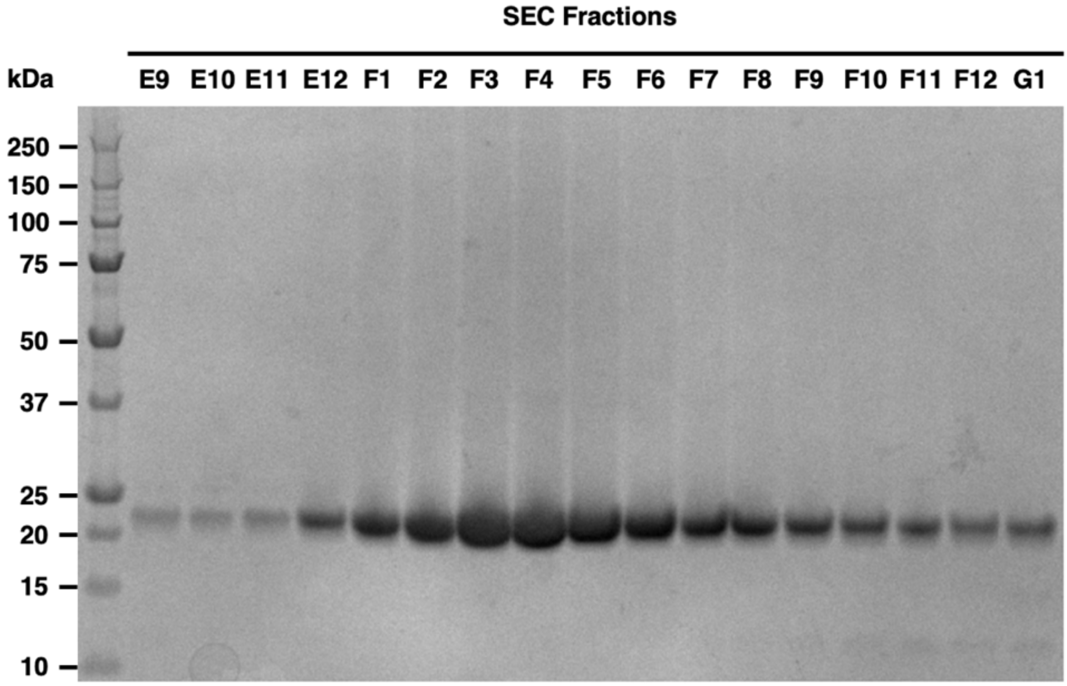

Figure S42: SDS-PAGE gel for purification of untagged TRIM24 by size-exclusion chromatography (SEC).

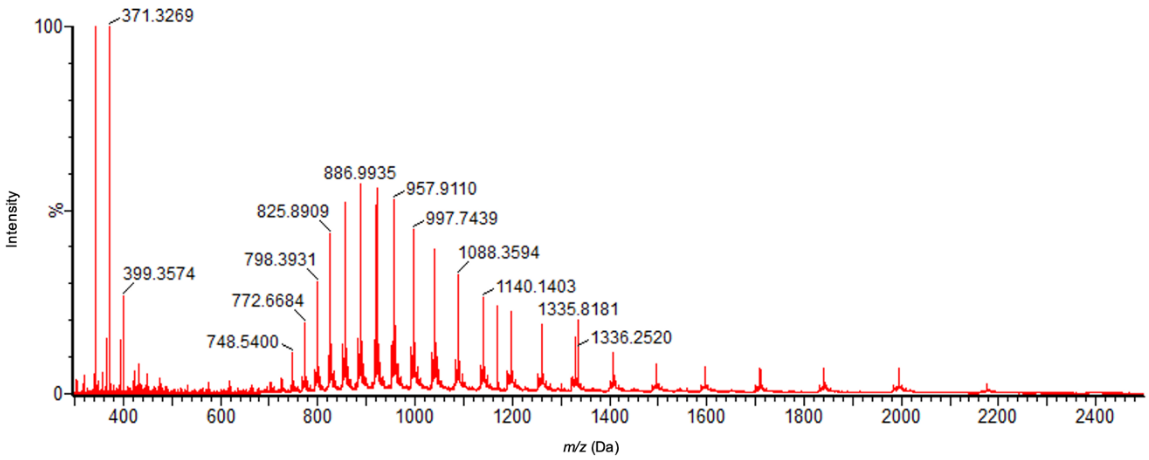

Figure S43: ESI$^+$-MS spectrum for His$_6$-TRIM24

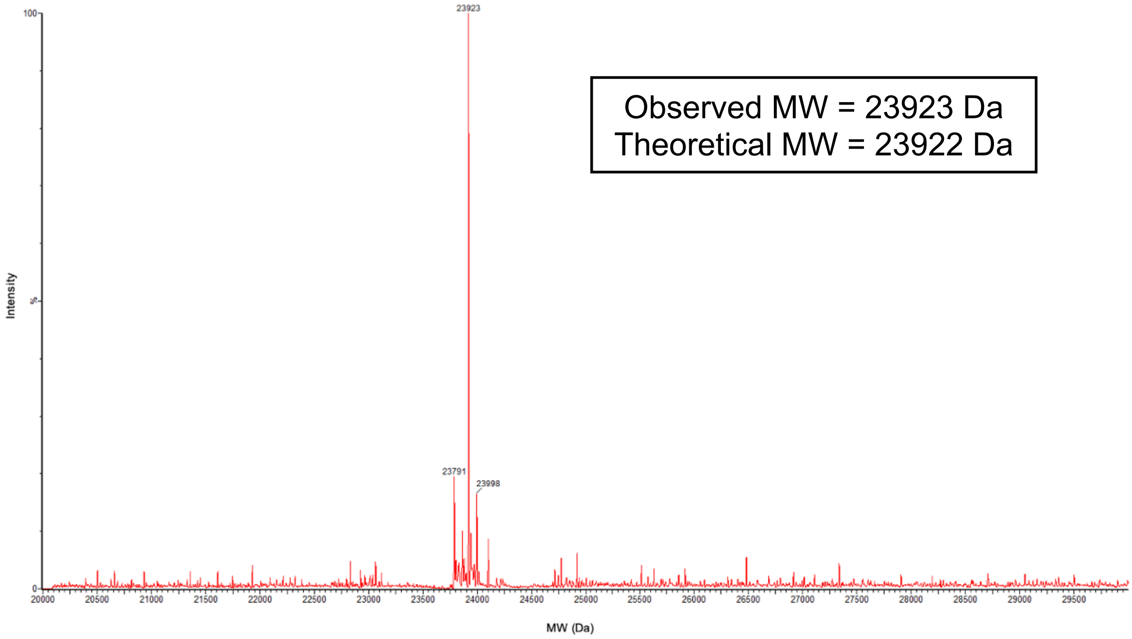

Figure S44: Deconvoluted MS spectrum for His$_6$-TRIM24

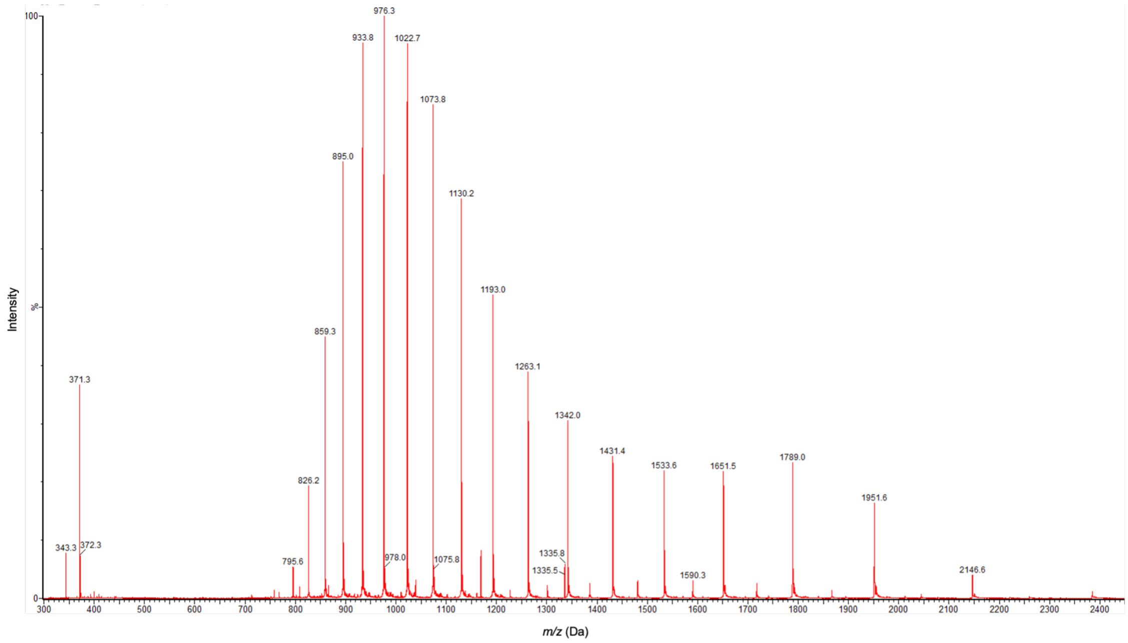

Figure S45: ESI$^+$-MS spectrum for untagged TRIM24.

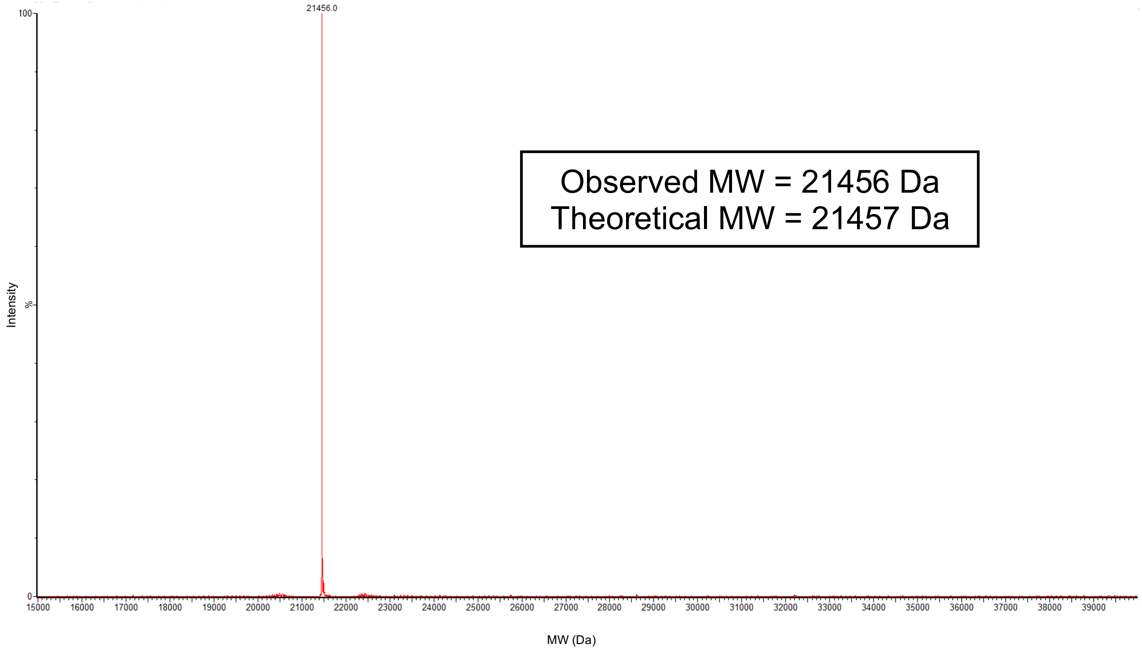

Figure S46: Deconvoluted MS spectrum for untagged TRIM24.

2 Supplementary Tables

Table S1: Summary table of peptide molecular weights after deconvolution (ESI$^+$-MS).

Peptide	Peptide Modification	Theoretical MW (Da)	Observed MW (Da)
P1	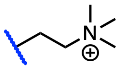	3134.73	3134.76
P2	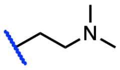	3119.70	3119.93
P3	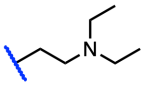	3147.75	3147.90
P4	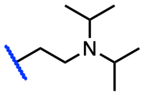	3175.81	3175.86
P5	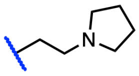	3145.74	3145.82
P6	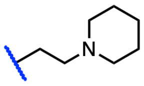	3159.76	3159.89
P7	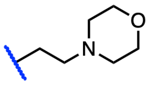	3161.74	3161.93
P8	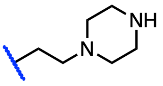	3160.75	3161.00
P9	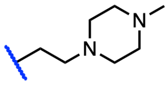	3174.78	3175.10
P10	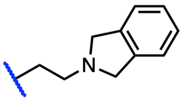	3193.78	3193.75
P11	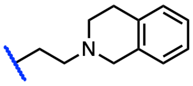	3207.81	3207.89
P12	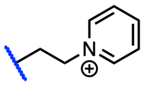	3154.72	3154.13
P13	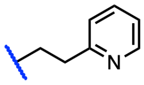	3153.72	3154.03
P14	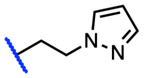	3142.69	3142.94
P15	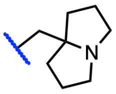	3171.78	3171.60
P16	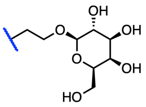	3254.77	3254.90

Table S2: NanoLC-MS/MS data for peptides P1 and P2 (Unalkylated peptide sequence: ARTKQTARCSTGGKAPRKQLATKAARKY).

Peptide and Modification	Fragment Peptide Amino Acid Sequences	*m/z*	Charge (*z*)	Theoretical Mass (Da)	Error (ppm)	-10log(*p*-value)	Modification site(s)
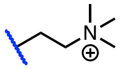 P1	QTARC(+86.10)STGGK	365.5308	3	1093.5789	-5.5	42.68	C9
	APRKQLATK	506.8168	2	1011.6189	2.3	65.33	–
	QLATKAARKY	575.3401	2	1148.6665	1.4	69.04	–
	QLATKAARKY	383.8959	3	1148.6665	1.6	68.09	–
	QTARCSTGGK	504.7487	2	1007.4818	3.1	44.98	–
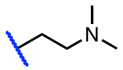 P2	QLATKAARKY	575.3378	2	1148.6665	-4.8	96.44	–
	QLATKAARKY	383.8938	3	1148.6665	-6.0	63.75	–
	ARTKQTARC(+71.07)STGGK	384.7150	4	1534.8362	-3.5	70.29	C9
	ARTKQTARC(+71.07)STGGK	512.6183	3	1534.8362	-2.1	66.64	C9
	QTARCSTGGK	504.7491	2	1007.4818	1.7	60.67	–
	QTARC(+71.07)STGGKAPRK	383.7159	4	1530.8413	-4.3	57.17	C9
	QTARC(+71.07)STGGKAPRK	766.4248	2	1530.8413	-4.1	49.14	C9
	QTARC(+71.07)STGGK	540.2843	2	1078.5553	-1.2	56.96	C9
	QTARC(+71.07)STGGK	360.5251	3	1078.5553	-1.8	52.59	C9
	A(+71.07)(+71.07)RTKQTARC(+71.07)STGGK	420.2516	4	1676.9832	-3.4	50.82	A1 (N-terminus) ×2, C9
	APRKQLATK	506.8165	2	1011.6189	-0.4	46.74	–
	APRKQLATK(+71.07)	361.9046	3	1082.6924	-0.4	46.61	K23
	QLATK(+71.07)AARKY	610.8752	2	1219.7400	-3.3	39.84	K23
	QTARC(+71.07)S(+71.07)T(+71.07)GGK	407.9077	3	1220.7023	-0.8	45.20	C9, S10, T11
	QLAT(+71.07)KAARKY	610.8772	2	1219.7400	-0.1	45.07	T22
	QLAT(+71.07)K(+71.07)AARKY	431.2800	3	1290.8136	3.5	41.30	T22, K23
	APRKQLAT(+71.07)K(+71.07)	385.5951	3	1153.7659	-2.1	38.84	T22, K23
	QTARC(+71.07)S(+71.07)T(+71.07)GGKAPRK	419.2528	4	1672.9883	-3.7	36.88	C9, S10, T11
	QTARC(+71.07)S(+71.07)TGGK	575.8159	2	1149.6288	-10.1	33.86	C9, S10

Table S3: AlphaScreen IC_{50} values for the inhibition of TRIM24-H3K9Me$_3$ (B1) binding by H3K9Me$_3$-mimicking peptides. IC_{50} values are quoted as the mean of triplicate data ± standard error of the mean. Hotter colours indicate stronger binders and colder colours indicate weaker binders. Predicted pKaH values (MolGpKa) are shown for the most basic N atom.[10]

Compound	Peptide Modification	AlphaScreen® IC_{50} (μM)	Predicted pKaH
P1	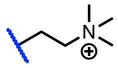	4.99 ± 0.52	–
P2	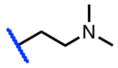	7.75 ± 0.72	10.0
P3	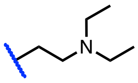	5.79 ± 0.83	10.3
P4	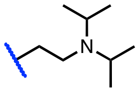	4.55 ± 0.21	10.5
P5	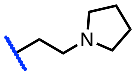	4.71 ± 0.66	10.9
P6	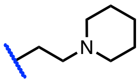	3.56 ± 0.30	10.1
P7	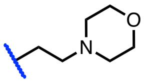	23.8 ± 8.9	7.6
P8	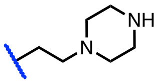	6.38 ± 0.92	9.6
P9	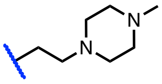	5.56 ± 0.70	8.2
P10	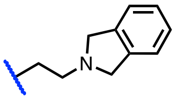	2.97 ± 0.21	7.3
P11	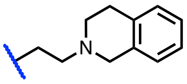	3.14 ± 0.33	8.7
P12	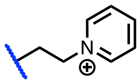	12.2 ± 2.4	–
P13	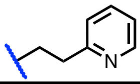	63.5 ± 34.9	5.9
P14	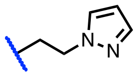	24.1 ± 4.0	2.7
P15	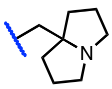	1.13 ± 0.08	11.5
P16	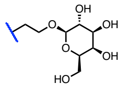	27.6 ± 8.4	–

Table S4: AlphaScreen IC_{50} values for the inhibition of TRIM24-H3K9Me$_3$ (B1) binding by the control compounds. IC_{50} values are quoted as the mean of triplicate data ± standard error of the mean. Hotter colours indicate stronger binders and colder colours indicate weaker binders.

Compound	AlphaScreen® IC_{50} (μM)
H3$_{(1–27)}$K9Me$_3$	4.21 ± 0.59
H3$_{(1–27)}$K9Me$_0$	9.98 ± 1.22
H3$_{(1–27)}$K9C	12.7 ± 1.6
H3$_{(1–15)}$K4Me$_3$	>400

Table S5: AlphaScreen IC_{50} values for the inhibition of TRIM24-H3K9Me$_3$ (B1) binding by the PDCs and control compounds. IC_{50} values are quoted as the mean of triplicate data ± standard error of the mean. Hotter colours indicate stronger binders and colder colours indicate weaker binders.

Compound	AlphaScreen® IC_{50} (nM)
H3$_{(1–27)}$K9Me$_3$	5030 ± 400
H3$_{(1–15)}$K4Me$_3$	>400000
PDC4 (50)	4.70 ± 0.5
PDC5 (51)	14.6 ± 0.3
PDC6 (52)	21.5 ± 0.8
56	3410 ± 290

Table S6: AlphaScreen IC_{50} values for the inhibition of TRIM24-H3K18Ac (B2) binding by the PDCs and control compounds. IC_{50} values are quoted as the mean of triplicate data ± standard error of the mean. Hotter colours indicate stronger binders and colder colours indicate weaker binders.

Compound	AlphaScreen® IC_{50} (nM)
$H3_{(1-27)}K18Ac$	266.4 ± 8.6
$H3_{(1-15)}K4Me_3$	>400000
IACS9571	19.0 ± 1.7
28	388.4 ± 46.6
32	104.7 ± 5.3
33	71.0 ± 4.1
34	94.2 ± 4.6
PDC4 (50)	4.8 ± 0.3
PDC5 (51)	11.8 ± 0.5
PDC6 (52)	16.7 ± 1.1

Table S7: AlphaScreen IC_{50} values for the inhibition of TRIM24-H3K9Me$_3$K18Ac (B3) binding by the PDCs and control compounds. IC_{50} values are quoted as the mean of triplicate data ± standard error of the mean. Hotter colours indicate stronger binders and colder colours indicate weaker binders.

**Compound**	**AlphaScreen® IC_{50} (nM)**
H3$_{(1–27)}$K9Me$_3$K18Ac	241.7 ± 13.7
H3$_{(1–15)}$K4Me$_3$	>400000
IACS9571[a]	31.2 ± 2.0
28[a]	>100000
32[a]	483.9 ± 47.8
33[a]	347.7 ± 65.7
34[a]	274.6 ± 51.8
PDC4 (50)	11.6 ± 0.3
PDC5 (51)	28.8 ± 0.8
PDC6 (52)	10.7 ± 0.2
56	4904 ± 346
32 + 56	305.2 ± 24.6
33 + 56	284.0 ± 18.1
34 + 56	209.8 ± 18.9

[a] AlphaScreen signal did not decrease to baseline.

Table S8: BROMO*scan* K_d values for TRIM24 and BRPF1. K_d values are quoted as the mean of duplicate data ± 95% confidence interval.

Compound	TRIM24 K_d (nM)	BRPF1 K_d (nM)
PDC4 (50)	0.27 ± 0.19	2.35 ± 0.88
PDC5 (51)	0.22 ± 0.05	2.15 ± 0.49
PDC6 (52)	0.18 ± 0.04	2.30 ± 0.39
34	2.00 ± 0.98	2.15 ± 0.49
56	3350 ± 686	>10000

Table S9: SPR K_d values (equilibrium/kinetic analysis) and rate constants for the binding of $H3_{(1–27)}K9Me_3K18Ac$, PDC6, and BRD ligand 34 to TRIM24. Values are quoted as the mean of means for triplicate data ± standard error of the mean.

Compound	K_d – equilibrium (nM)	K_d – kinetic (nM)	k_{on} (M^{-1} s^{-1})	k_{off} (s^{-1})
$H3_{(1–27)}K9Me_3K18Ac$	1144 ± 204	–	–	–
PDC6 (52)	–	0.6 ± 0.2	$1.3{\times}10^{7} \pm 1.4{\times}10^{6}$	$8.2{\times}10^{-3} \pm 3.1{\times}10^{-4}$
34	11.0 ± 2.3	10.0 ± 7.0	$6.1{\times}10^{6} \pm 4.6{\times}10^{5}$	$6.1{\times}10^{-2} \pm 3.2{\times}10^{-3}$

Table S10: His_6-TRIM24 (PHD-BRD) construct information.

**Gene**	His_6-TRIM24 (PHD-BRD)
**Uniprot ID**	O15164
**Residues**	N825–E1007
**DNA sequence**	ATGCACCATCATCATCATCATTCTTCTGGTGTAGATCTGGGT ACCGAGAACCTGTACTTCCAATCCATGAATGAGGACTGGTG TGCAGTTTGTCAAAACGGAGGGGAACTCCTCTGCTGTGAAA AGTGCCCCAAAGTATTCCATCTTTCTTGTCATGTGCCCACAT TGACAAATTTTCCAAGTGGAGAGTGGATTTGCACTTTCTGC CGAGACTTATCTAAACCAGAAGTTGAATATGATTGTGATGCT CCCAGTCACAACTCAGAAAAAAAGAAAACTGAAGGCCTTGT TAAGTTAACACCTATAGATAAAAGGAAGTGTGAGCGCCTACT TTTATTTCTTTACTGCCATGAAATGAGCCTGGCTTTTCAAGA CCCTGTTCCTCTAACTGTGCCTGATTATTACAAAATAATTAAA AATCCAATGGATTTGTCAACCATCAAGAAAAGACTACAAGAA GATTATTCCATGTACTCAAAACCTGAAGATTTTGTAGCTGATT TTAGATTGATCTTTCAAAACTGTGCTGAATTCAATGAGCCTG ATTCAGAAGTAGCCAATGCTGGTATAAAACTTGAAAATTATTT TGAAGAACTTCTAAAGAACCTCTATCCAGAATGA
**Amino Acid Sequence**	MHHHHHHSSGVDLGTENLYFQSMNEDWCAVCQNGGELLCC EKCPKVFHLSCHVPTLTNFPSGEWICTFCRDLSKPEVEYDCD APSHNSEKKKTEGLVKLTPIDKRKCERLLLFLYCHEMSLAFQD PVPLTVPDYYKIIKNPMDLSTIKKRLQEDYSMYSKPEDFVADFR LIFQNCAEFNEPDSEVANAGIKLENYFEELLKNLYPE
**MW (Da)**	23922
**ε (M^{-1} cm^{-1})**	25160

Table S11: His_6SUMO-TRIM24 (PHD-BRD) construct information.

**Gene**	His_6SUMO-TRIM24 (PHD-BRD)
**Uniprot ID**	O15164
**Residues**	N825–E1007
**DNA sequence**	ATGAAACATCACCATCACCATCACCCCATGAGCGATTACGAC ATCCCCACTACTGAGAATCTTTATTTTCAGGGCGCCATGGGC AACGATCACATTAACCTGAAAGTGGCCGGTCAAGACGGTAG CGTAGTCCAGTTTAAAATCAAACGCCACACCCCTCTGTCGA

	AACTGATGAAAGCCTATTGTGAACGCCAAGGTCTGTCTATG CGTCAGATCCGTTTTCGCTTCGATGGACAGCCGATTAACGA AACCGACACTCCAGCACAGCTGGAAATGGAAGATGAGGAC ACCATTGACGTGTTCCAGCAACAGACCGGTGGATCCATGAA TGAGGACTGGTGTGCAGTTTGTCAAAACGGAGGGGAACTC CTCTGCTGTGAAAAGTGCCCCAAAGTATTCCATCTTTCTTGT CATGTGCCCACATTGACAAATTTTCCAAGTGGAGAGTGGATT TGCACTTTCTGCCGAGACTTATCTAAACCAGAAGTTGAATAT GATTGTGATGCTCCCAGTCACAACTCAGAAAAAAAGAAAAC TGAAGGCCTTGTTAAGTTAACACCTATAGATAAAAGGAAGTG TGAGCGCCTACTTTTATTTCTTTACTGCCATGAAATGAGCCT GGCTTTTCAAGACCCTGTTCCTCTAACTGTGCCTGATTATTA CAAAATAATTAAAAATCCAATGGATTTGTCAACCATCAAGAAA AGACTACAAGAAGATTATTCCATGTACTCAAAACCTGAAGAT TTTGTAGCTGATTTTAGATTGATCTTTCAAAACTGTGCTGAAT TCAATGAGCCTGATTCAGAAGTAGCCAATGCTGGTATAAAAC TTGAAAATTATTTTGAAGAACTTCTAAAGAACCTCTATCCAGA ATGA
**Amino Acid Sequence (| = cleavage site)**	MKHHHHHHPMSDYDIPTTENLYFQGAMGNDHINLKVAGQDG SVVQFKIKRHTPLSKLMKAYCERQGLSMRQIRFRFDGQPINET DTPAQLEMEDEDTIDVFQQQTGG | SMNEDWCAVCQNGGELL CCEKCPKVFHLSCHVPTLTNFPSGEWICTFCRDLSKPEVEYD CDAPSHNSEKKKTEGLVKLTPIDKRKCERLLLFLYCHEMSLAF QDPVPLTVPDYYKIIKNPMDLSTIKKRLQEDYSMYSKPEDFVAD FRLIFQNCAEFNEPDSEVANAGIKLENYFEELLKNLYPE
**MW (Da)**	33804; 21457 (cleaved)
**ε (M^{-1} cm^{-1})**	28140; 23670 (cleaved)

Table S12: GST-TRIM24 (PHD-BRD) construct information.

**Gene**	GST-TRIM24 (PHD-BRD)
**Uniprot ID**	O15164
**Residues**	N825–E1007
**DNA sequence**	AAATTAACACAGTCTATGGCCATCATACGTTATATAGCTGACA AGCACAACATGTTGGGTGGTTGTCCAAAAGAGCGTGCAGA GATTTCAATGCTTGAAGGAGCGGTTTTGGATATTAGATACGG TGTTTCGAGAATTGCATATAGTAAAGACTTTGAAACTCTCAAA

	GTTGATTTTCTTAGCAAGCTANNNGAAATGCTGAAAATGTTC<br>GAAGATCGTTTATGTCATAAAACATATTTAAATGGTGATCATG<br>TAACCCATCCTGACTTCATGTTGTATGACGCTCTTGATGTTG<br>TTTTATACATGGACCCAATGTGCCTGGATGCGTTCCCAAAAT<br>TAGTTTGTTTTAAAAAACGTATTGAAGCTATCCCACAAATTGA<br>TAAGTACTTGAAATCCAGCAAGTATATAGCATGGCCTTTGCA<br>GGGCTGGCAAGCCACGTTTGGTGGTGGCGACCATCCTCCA<br>AAATCGAGCTCAGAGAACCTGTACTTCCAATCCATGAATGAG<br>GACTGGTGTGCAGTTTGTCAAAACGGAGGGGAACTCCTCT<br>GCTGTGAAAAGTGCCCCAAAGTATTCCATCTTTCTTGTCATG<br>TGCCCACATTGACAAATTTTCCAAGTGGAGAGTGGATTTGC<br>ACTTTCTGCCGAGACTTATCTAAACCAGAAGTTGAATATGATT<br>GTGATGCTCCCAGTCACAACTCAGAAAAAAAGAAAACTGAA<br>GGCCTTGTTAAGTTAACACCTATAGATAAAAGGAAGTGTGAG<br>CGCCTACTTTTATTTCTTTACTGCCATGAAATGAGCCTGGCT<br>TTTCAAGACCCTGTTCCTCTAACTGTGCCTGATTATTACAAAA<br>TAATTAAAAATCCAATGGATTTGTCAACCATCAAGAAAAGACT<br>ACAAGAAGATTATTCCATGTACTCAAAACCTGAAGATTTTGTA<br>GCTGATTTTAGATTGATCTTTCAAAACTGTGCTGAATTCAATG<br>AGCCTGATTCAGAAGTAGCCAATGCTGGTATAAAACTTGAAA<br>ATTATTTTGAAGAACTTCTAAAGAACCTCTATCCAGAATGA
**Amino Acid Sequence**	MSPILGYWKIKGLVQPTRLLLEYLEEKYEEHLYERDEGDKWRN<br>KKFELGLEFPNLPYYIDGDVKLTQSMAIIRYIADKHNMLGGCPK<br>ERAEISMLEGAVLDIRYGVSRIAYSKDFETLKVDFLSKLPEMLK<br>MFEDRLCHKTYLNGDHVTHPDFMLYDALDVVLYMDPMCLDAF<br>PKLVCFKKRIEAIPQIDKYLKSSKYIAWPLQGWQATFGGGDHP<br>PKSSSENLYFQSMNEDWCAVCQNGGELLCCEKCPKVFHLSC<br>HVPTLTNFPSGEWICTFCRDLSKPEVEYDCDAPSHNSEKKKT<br>EGLVKLTPIDKRKCERLLLFLYCHEMSLAFQDPVPLTVPDYYKII<br>KNPMDLSTIKKRLQEDYSMYSKPEDFVADFRLIFQNCAEFNEP<br>DSEVANAGIKLENYFEELLKNLYPE
**MW (Da)**	47993
**ε (M^{-1} cm^{-1})**	68270

Table S13: X-ray diffraction data collection and refinement statistics. Data for the highest resolution shell are shown in parentheses.

**Crystal**	**TRIM24 (PHD-BRD) residues 825–1007 in the *apo* state**	**TRIM24 (PHD-BRD) residues 825–1007 in complex with ligand 32**
PDB ID	9GD5	9GDG
Beamline	I03 (Diamond Light Source)	I03 (Diamond Light Source)
Wavelength (Å)	0.9763	0.9763
Space group	C121	C121
Unit Cell		
a, b, c (Å)	88.90, 36.91, 129.36	90.94, 36.65, 65.46
α, β, γ (°)	90.00, 110.04, 90.00	90.00, 111.33, 90.00
Resolution (Å)	44.45–1.68 (1.71–1.68)	60.97–1.46 (1.64–1.46)
No. of reflections	282838 (6530)	153510 (5178)
No. of unique reflections	43525 (1485)	23849 (1192)
I/σ (I)	18.1 (1.2)	18.5 (2.0)
Completeness (%)	95.7 (66.1)	84.9 (32.6)
Multiplicity	6.5 (4.4)	6.4 (4.3)
R_{meas}	0.144 (2.469)	0.051 (0.623)
R_{factor}/R_{free}	0.21/0.24	0.15/0.21
Number of atoms (non-H)		
Protein (Chain A)	1439	1431
Protein (Chain B)	1439	–
Water	130	119
Zinc	4	2
Ligand	–	48
Average B-factors (Å^2)		
Protein (Chain A)	25.596	25.127
Protein (Chain B)	26.667	–
Water	27.278	29.831
Zinc	21.690	17.093
Ligand	–	53.996
RMS deviations		
Bond lengths (Å)	0.0090	0.0139
Bond Angles (°)	1.6020	2.1750
Ramachandran favoured (%)	99.4	98.8
Ramachandran allowed (%)	0.6	1.2
Ramachandran outliers (%)	0.0	0.0

3 Supplementary Schemes

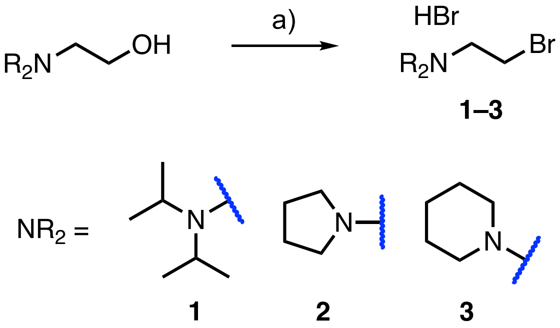

Scheme S1: Synthesis of alkylating agents **1**–**3**. *Reagents and conditions*: a) Conc. aqueous HBr, 0–135 °C, 20 h, (**1**) 34%, (**2**) 52%, (**3**) 39%.

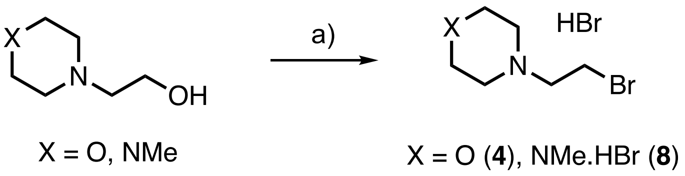

Scheme S2: Synthesis of alkylating agents **4** and **8**. *Reagents and conditions*: a) PPh_3Br_2, CH_2Cl_2, 0 °C–rt, 6–20 h, (**4**) 70%, (**8**) 44%.

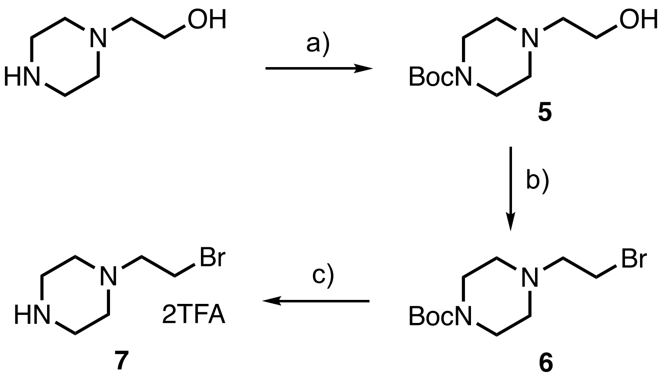

Scheme S3: Synthesis of alkylating agent **7**. *Reagents and conditions*: a) Boc_2O, THF, 0 °C, 2 h, 99%; b) CBr_4, PPh_3, CH_2Cl_2, 0 °C–rt, 20 h, 90%; c) TFA, CH_2Cl_2, 0 °C–rt, 2 h, 84%.

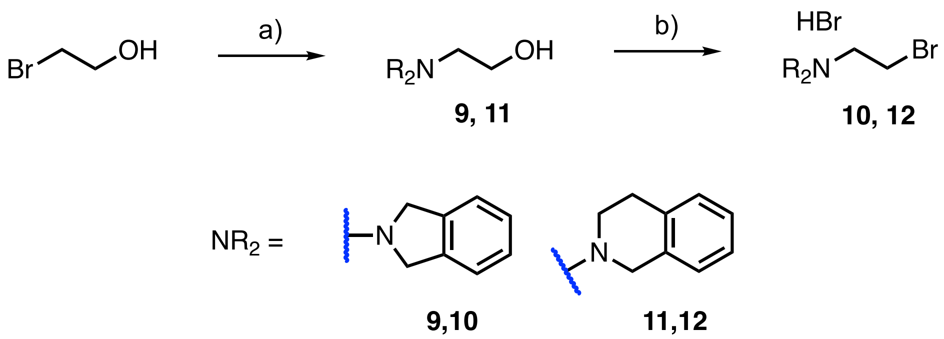

Scheme S4: Synthesis of alkylating agents **10** and **12**. *Reagents and conditions*: a) R_2NH, 2-bromoethanol, NEt_3, toluene, rt–120 °C, 6–20 h, (**9**) 44%, (**11**) 74%; b) conc. aqueous HBr, 0–135 °C, 20 h, (**10**) 61%, (**12**) 78%.

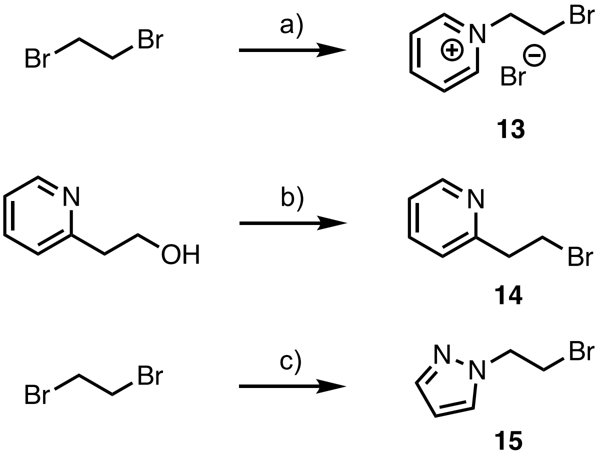

Scheme S5: Synthesis of alkylating agents **13**–**15**. *Reagents and conditions*: a) Pyridine, acetone, rt, 60 °C, 16 h, 74%; (b) CBr_4, PPh_3, THF, 0 °C–rt, 20 h, 45%; b) Pyrazole, TBAB (10 mol%), aqueous NaOH, rt, 24 h, 62%.

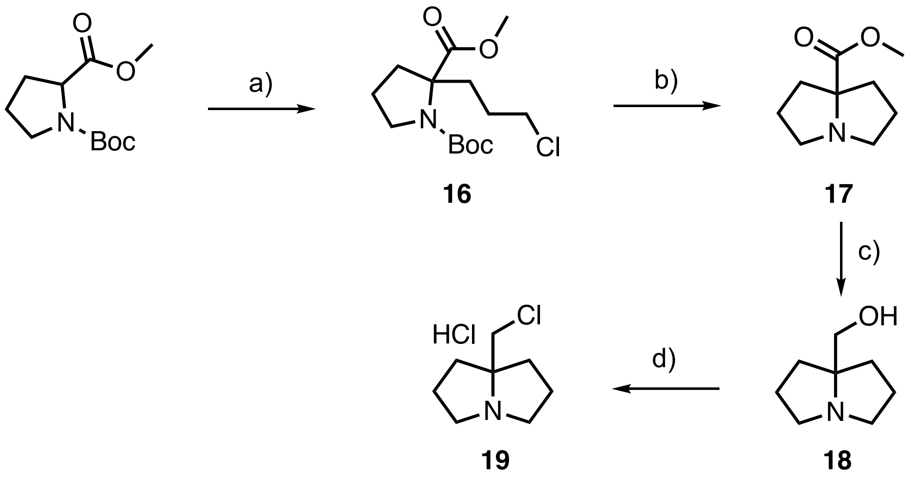

Scheme S6: Synthesis of alkylating agent **19**. *Reagents and conditions*: a) LDA, THF, −78 °C, 2 h, then $ClCH_2CH_2CH_2I$, −78 °C–rt, 2 h, 68%; b) TMSCl, MeOH, 0 °C–rt, 16 h, 79%; c) $LiAlH_4$, THF, −10 °C–rt, 1 h, 88%; d) $SOCl_2$, toluene, 0–80 °C, 3 h, 91%.

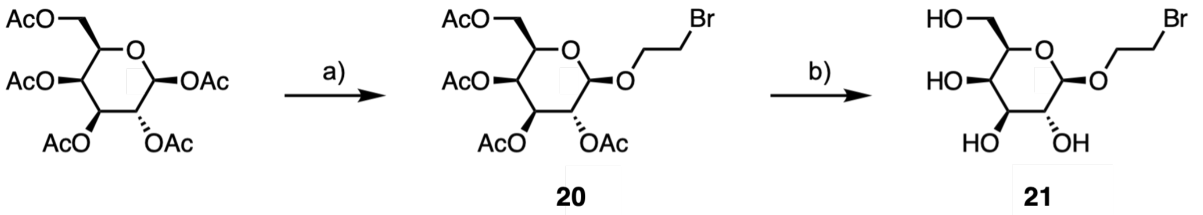

Scheme S7: Synthesis of alkylating agent **21**. *Reagents and conditions*: a) $BF_3{\cdot}OEt_2$, 2-bromoethanol, CH_2Cl_2, 0 °C, 3 h, 58%; b) NaOMe, MeOH, rt, 4 h, 57%.

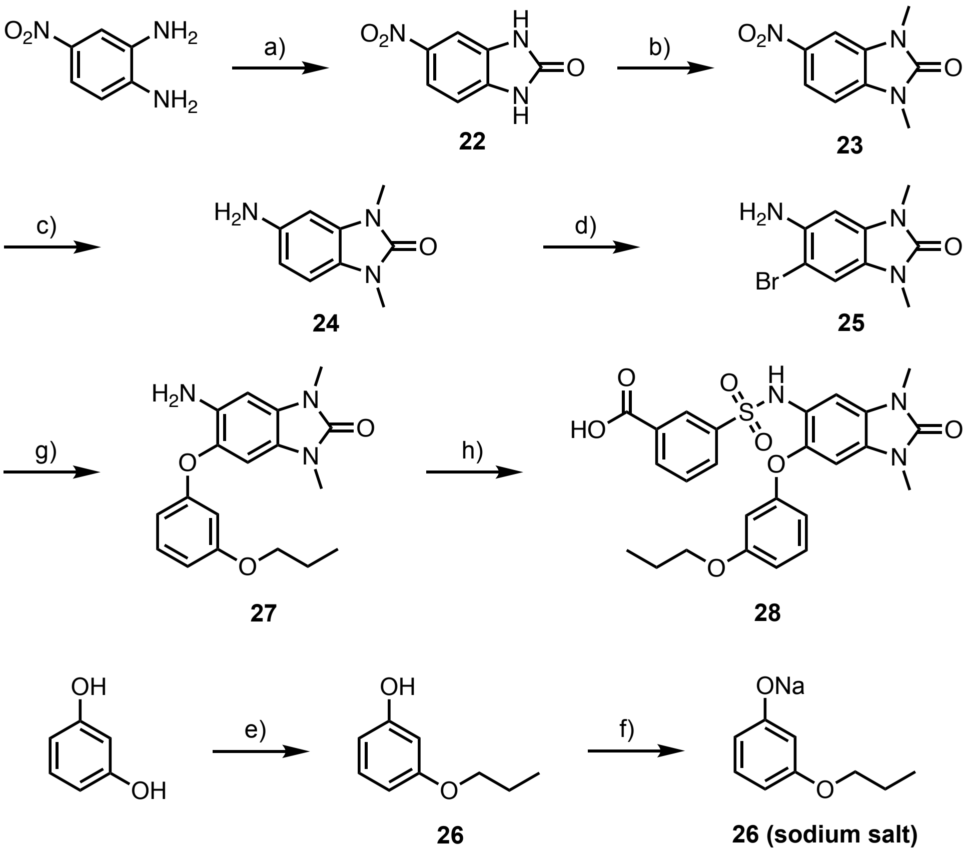

Scheme S8: Synthesis of TRIM24 BRD ligand **28**.[11,12] *Reagents and conditions:* a) CDI, DMF, rt, 20 h, >99%; b) MeI, K_2CO_3, DMF, 0 °C–rt, 16 h, 97%; c) 10% Pd/C (10 mol%), $H_{2(g)}$ 1 atm., conc. HCl (aq), EtOH, rt, 16 h, 97%; d) Br_2, AcOH, $CHCl_3$, –20 °C, 20 min, 73%; e) $CH_3CH_2CH_2Br$, K_2CO_3, DMF, rt–50 °C, 16 h, 69%; f) NaOH, MeOH, rt, 1 h, 100%; g) Sodium 3-propoxy phenolate (from **26**), CuI (10 mol%), Cs_2CO_3, 8-hydroxyquinoline, diglyme, 130 °C, 72 h, 62%; h) 3-(Chlorosulfonyl)benzoic acid, pyridine, CH_2Cl_2, rt, 24 h, 71%.

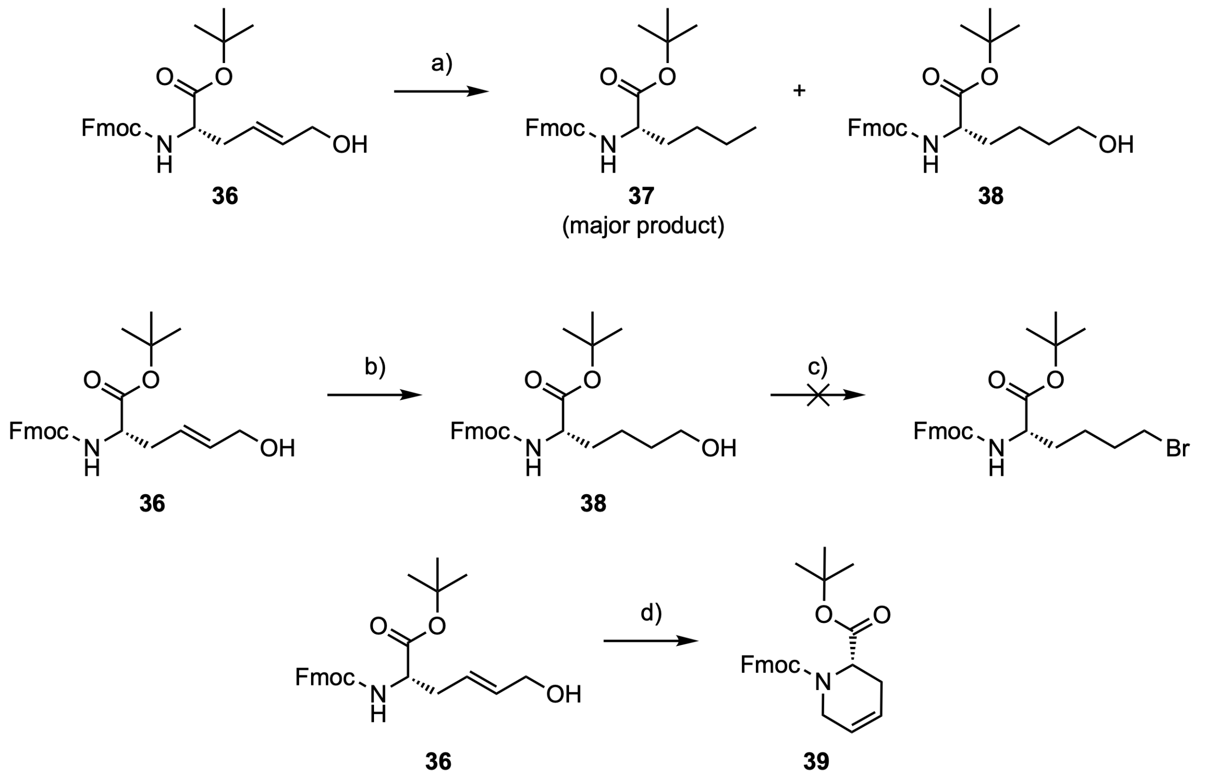

Scheme S9: Unsuccessful routes towards the synthesis of unnatural amino acid **42**. *Reagents and conditions:* a) 10% Pd/C (10 mol%), $H_{2(g)}$ 1 atm., EtOH, rt, 16 h, **37**: 37%; **38**: 5%; b) PtO_2 (10 mol%), $H_{2(g)}$ 1 atm., EtOAc, rt, 20 h, 75%; c) PPh_3, CBr_4, CH_2Cl_2, 0 °C–rt, 20 h; d) PPh_3, CBr_4, CH_2Cl_2, 0 °C–rt, 2 h, 63%.

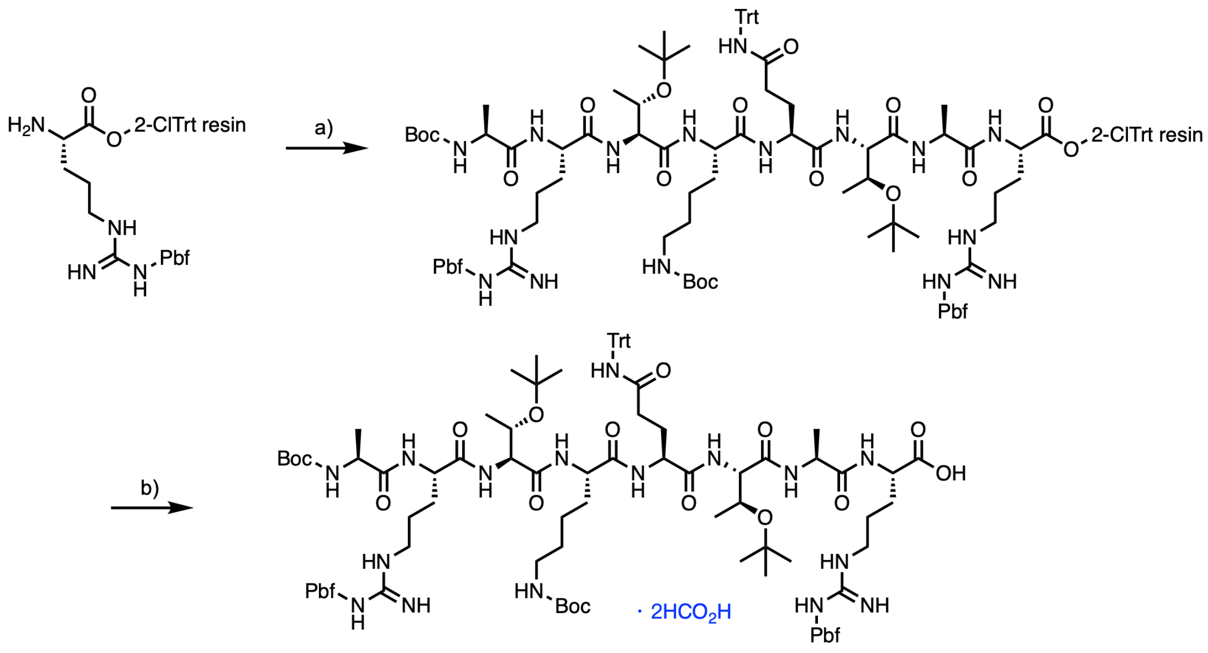

46

Scheme S10: Solid phase peptide synthesis (SPPS) of protected histone 3-mimicking peptide **46** using a Liberty Blue™ peptide synthesiser. *Reagents and conditions*: a) (Coupling) Fmoc-protected amino acid, DIC, Oxyma Pure, DIPEA, DMF, rt; (Fmoc deprotection) piperidine, DMF, rt; b) (Resin cleavage) HFIP, CH_2Cl_2, rt, 1 h.

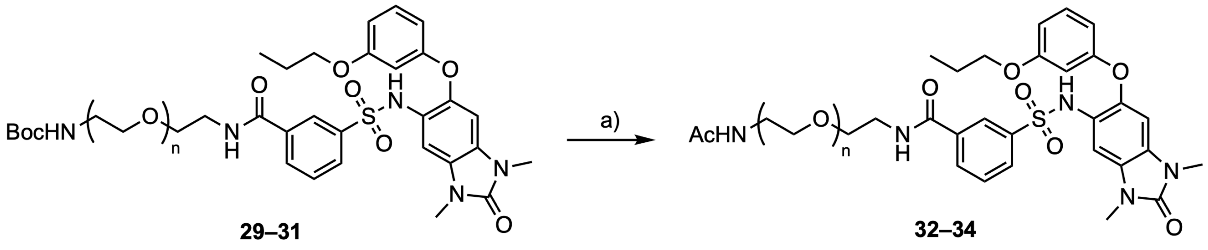

Scheme S11: Synthesis of TRIM24 BRD ligands **32**–**34**. *Reagents and conditions:* a) HCl in dioxane (4 M), rt, 2 h; then AcCl, NEt_3, CH_2Cl_2, 0 °C–rt, 20 min, **32**: n=2, 72%; **33**: n=3, 63%; **34**: n=4, 43%.

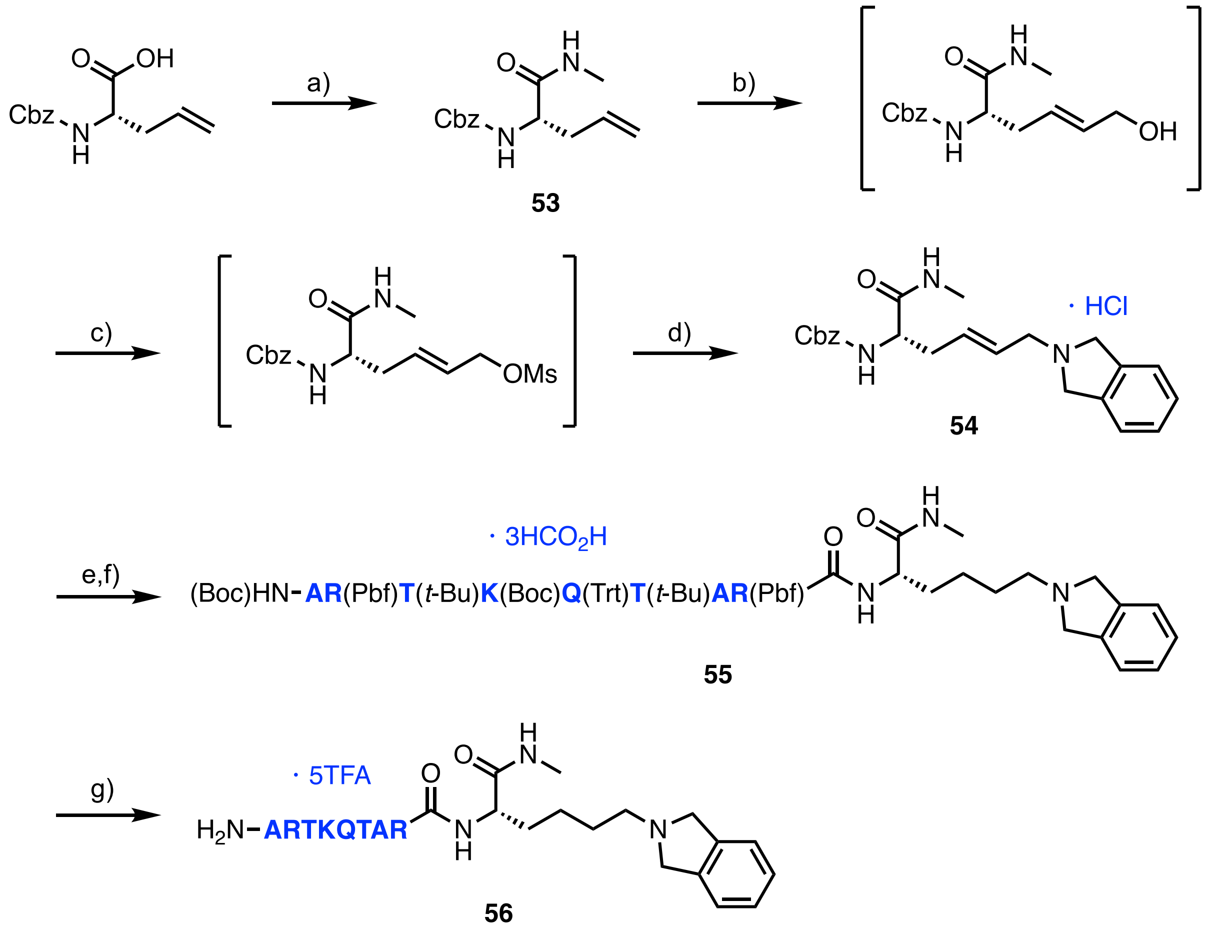

Scheme S12: Synthesis of the parent PHD-binding peptide (**56**). *Reagents and conditions:* a) $MeNH_2$ (2 M in THF), HATU, DIPEA, DMF, rt, 2 h (94%); b) *cis*-Butene-1,4-diol, Grubbs **II** (5 mol%), CH_2Cl_2, reflux, 40 °C, 6 h; c) MsCl, NEt_3, CH_2Cl_2, 0 °C–rt, 1 h; d) Isoindoline, DMF, rt, 1 h, 36% over 3 steps; e) 10% Pd/C (10 mol%), $H_{2(g)}$ 1 atm., MeOH, rt, 6 h; f) **46**, HATU, DIPEA, DMF, rt, 6 h, 28% over 2 steps; g) TFA:TIPS:H_2O (38:1:1), rt, 4 h, 70%.

4 Biological and Biochemical Methods

General Biological Methods

Purified (Milli-Q®) Water was obtained from a Millipore Elix® Reverse Osmosis system and was further purified by a Millipore Milli-Q® Synthesis system with a 0.22 µm filter on the outlet.

pH Measurements were carried out using a PH 550 Benchtop pH Meter Kit (Oakton). Calibration was performed with standard solutions at pH 4.0 (solution of potassium acid phthalate, cetylpyridinium chloride and methyl red), pH 7.0 (solution of potassium dihydrogen phosphate, cetylpyridinium chloride, sodium hydrogen phosphate and methyl orange), and pH 10.0 (solution of sodium carbonate, sodium bicarbonate and thymol blue). The pH probe was stored in 4 M potassium chloride solution.

Peptides were purchased from GenScript at >98% purity. A terminal tyrosine was included for accurate quantification of peptide concentrations using absorbance at 280 nm.

Table S14: Unbiotinylated peptide (>98% purity, Genscript) amino acid sequences.

Peptide	Amino Acid Sequence (N–C)
$H3_{(1–27)}K18Ac$	ARTKQTARKSTGGKAPRK(Ac)QLATKAARKY
$H3_{(1–27)}K9Me_3$	ARTKQTARK(Me_3)STGGKAPRKQLATKAARKY
$H3_{(1–27)}K9Me_3K18Ac$	ARTKQTARK(Me_3)STGGKAPRK(Ac)QLATKAARKY
$H3_{(1–27)}K9Me_0$	ARTKQTARKSTGGKAPRKQLATKAARKY
$H3_{(1–27)}K9C$	ARTKQTARCSTGGKAPRKQLATKAARKY
$H3_{(1-15)}K4Me_3$	ARTK(Me_3)QTARKSTTGKA

Table S15: Biotinylated peptide (>98% purity, Genscript) amino acid sequences (b=biotin).

Peptide	Amino Acid Sequence (N–C)
$H3_{(1–27)}K9Me_3$ (B1)	ARTKQTARK(Me_3)STGGKAPRKQLATKAARK(b)Y
$H3_{(1–27)}K18Ac$ (B2)	ARTKQTARKSTGGKAPRK(Ac)QLATKAARK(b)Y
$H3_{(1–27)}K9Me_3K18Ac$ (B3)	ARTKQTARK(Me_3)STGGKAPRK(Ac)QLATKAARK(b)Y

Protein/Peptide Absorbance at 280 nm (A_{280}) was measured on a NanoDrop™ Lite (Thermo Fisher Scientific) using the Protein A280 setting.

Protein/Peptide Concentrations were calculated using the Beer-Lambert law with the path length set at 1 cm (Equation 1). H3 peptide extinction coefficients (ε) were approximated as being equal to that of tyrosine (1490 M^{-1} cm^{-1}). Protein molecular weights and molar extinction coefficients were calculated using ProtParam on the ExPASy Bioinformatics Resource Portal.

$$c = \frac{A_{280}}{\varepsilon} \qquad \text{(Eq. 1)}$$

Sodium Dodecyl Sulfate-Polyacrylamide Gel Electrophoresis (SDS-PAGE) was carried out on precast Invitrogen NuPAGE™ 4–12% Bis-Tris precast gels (Thermo Fisher Scientific) using a mini-PROTEAN Tetra Electrophoresis System (Bio-Rad). Protein samples were mixed with 4× Laemmli sample loading buffer in a 3:1 ratio (sample:buffer) and incubated at 100 ºC for 3 min. Denatured samples were loaded onto the gel and the gels were run in 1× MES buffer at 180 V for 50 min. Following electrophoresis, gels were stained with InstantBlue™ Coomassie for 15 min and rinsed with water, then destained in water overnight.

Lyophilisation was carried out using a Christ Alpha 2-4 LSCBasic Laboratory freeze dryer. Samples were flash frozen in liquid nitrogen, before being lyophilised overnight.

Liquid Chromatography-Mass Spectrometry (LC-MS) was carried out using a Waters Xevo G2-S QTOF mass spectrometer, equipped with a Waters Acquity UPLC or an Agilent 1260 Infinity II system with a Quaternary LC Pump, DADWR, and InfinityLab LC/MSD. For histone-mimicking peptide characterisation, an ACE® Equivalence™ C18 [3 µm, 2.1 × 50 mm] column (ACE) was used with a constant flow rate of 0.3 mL/min and gradient method of 7 min from H_2O:MeCN (99:1) to H_2O:MeCN (50:50). Mass spectra were deconvoluted using Waters MassLynx™ software (MaxEnt1 algorithm, 3–5 kDa range, 0.01 Da resolution). Theoretical peptide molecular weights were calculated using ChemDraw 20.0. For the cell permeability assay, a Poroshell 120 EC-C18 [4 µm, 4.6 × 100 mm] column was used with a constant flow rate of 1.0 mL/min and a gradient method of 10 min from H_2O:MeCN (95:5) to H_2O:MeCN (5:95) with a 5 min hold. All samples were run with 0.1% formic acid in the elution solvents.

NanoLC-MS/MS was carried out after enzymatic digestion with endoproteinase LysC (1 µg enzyme/100 µg peptide sample for 2 h at 37 °C), using an UltiMate™ 3000 RSLCnano System (Thermo Scientific) coupled in line with a Q Exactive™ mass spectrometer, equipped with an Easy-Spray source (Thermo Scientific). Peptide samples were trapped onto a C18 PepMac100 precolumn (300 µm x 5 mm, 100 Å, Thermo Scientific) using Solvent A (0.1% formic acid, HPLC grade water). The peptides were further separated onto an Easy-Spray RSLC C18 column (75 um × 50 cm, Thermo Scientific) using a 15 min linear gradient [15–35% solvent B (0.1% formic acid in MeCN)] at a flow rate 200 nL/min. The raw data were acquired on the mass spectrometer in data-dependent acquisition mode (DDA). Full-scan MS spectra were acquired in the Orbitrap (Scan range 350-1500 *m/z*; resolution: 70,000; AGC target: 3×10^6; maximum injection time: 50 ms). The 5 most intense peaks were selected for higher-energy collision dissociation (HCD) fragmentation at 30% of normalized collision energy. HCD spectra were acquired using the Orbitrap (resolution: 17,500; AGC target: 5×10^4; maximum

injection time: 120 ms with fixed mass at 180 *m/z*). Charge exclusion was selected for unassigned and 1^+ ions. The dynamic exclusion was set to 5 s. The data were processed using the PEAKS software and results were filtered using a False Discovery Rate (FDR) <1%.

MALDI-TOF Mass Spectrometry was carried out on a Bruker Autoflex™ Speed MALDI-TOF mass spectrometer. Sample plates were prepared using the dried droplet method. Peptide samples were diluted in MeCN:H_2O (30:70) (0.1% TFA) to a final concentration of ~1 μM and premixed in a 1:1 ratio with a 10 mg/mL matrix solution of α-cyano-4-hydroxycinnamic acid (CHCA) in MeCN:0.1% TFA in H_2O (70:30); before being spotted (0.5 μL) onto an MTP 384 ground steel target plate (Bruker Daltonics). Mass spectra were acquired by manual operation in positive reflectron mode with a laser intensity of 38%. Spectra were processed using Bruker flexAnalysis software.

Intact Protein Mass Spectrometry was carried out using a Waters Xevo G2-XS QTOF mass spectrometer, equipped with a Waters Acquity UPLC. A ProSwift™ RP-2H monolithic 4.6 mm × 50 mm column (Thermo Scientific™) was used with a constant flow rate of 0.4 mL/min and a gradient method of 10 min from H_2O:MeCN (95:5) to H_2O:MeCN (5:95) with a 5 min hold. All samples were run with 0.1% formic acid in the elution solvents. Mass spectra were deconvoluted using Waters MassLynx™ software (MaxEnt1 algorithm, 20–30 kDa range, 1.0 Da resolution).

Analytical HPLC was carried out using a PerkinElmer Flexar system with a Binary LC Pump, and UV/VIS LC Detector set at 220 nm. For purification of peptides a Dionex Acclaim® 120 C18 [5 μm, 120 Å, 4.6 mm × 150 mm] reverse phase column was used with a constant flow rate of 1.0 mL/min and a gradient method of 25 min from H_2O:MeCN (95:5) to H_2O:MeCN (35:65). All samples were run with 0.1% TFA in the elution solvents. Peaks corresponding to monoalkylated peptides were collected and rerun with an identical gradient for purity determination. HPLC data were processed using Chromera 3.4.4 software.

Synthesis of Histone-mimicking Peptides P1–P16

Table S16: Alkylation Buffer (pH 7.8) composition.

Reagent	Mass required per 100 mL	Concentration
HEPES	6.46 g	0.27 M
HEPES sodium salt	18.98 g	0.73 M
D/L-Methionine	149 mg	10 mM
Guanidine hydrochloride	38.21 g	4 M

HEPES, HEPES sodium salt, and D/L-methionine were dissolved with Milli-Q® water (50 mL) in a 100 mL Duran bottle. Guanidine hydrochloride was added portion wise over 5 min with stirring. After all solids had dissolved, the pH was adjusted to pH 7.8. The resulting solution was transferred to a 100 mL measuring cylinder and topped up to 100 mL with Milli-Q® water. The solution was filtered through 0.20 μm nylon filter paper under vacuum and stored at 4 °C. The alkylation buffer was degassed for 30 min prior to use.

Alkylation reactions were carried out using adapted versions of protocols reported by Simon *et al.* and Pieters *et al.*.[13,14] Lyophilised $H3_{(1\text{-}27)}$K9C peptides (5 mg) were dissolved in alkylation buffer (980 μL) and mixed by gentle inversion. Dithiothreitol (1 M, 20 μL) was added, and the solutions were incubated at 37 °C with shaking at 350 rpm for 1 h. Alkylating agents were then added directly to the reduced peptides in the specified molar ratios (Table S17). **P10**, **P11**, **P13**, and **P14** reaction mixtures were supplemented with DMSO (20 μL) to improve the solubility of the reagents. Reaction mixtures were flicked gently, covered with aluminium foil, and incubated at the specified temperatures (Table S17) with shaking at 350 rpm for 2.5 h. Dithiothreitol (1 M, 10 μL) was added and the reactions were allowed to proceed for a further 2.5 h. Reactions were quenched by addition of β-mercaptoethanol (50 μL) and incubated at rt for 30 min. Peptides were desalted using Pierce™ polyacrylamide desalting columns (1.8K MWCO, Thermo Fisher), and eluted with an aqueous solution of β-mercaptoethanol (2 mM). Samples (10 μL) from each collected fraction were diluted in 90 μL of β-mercaptoethanol (2 mM) and analysed using LC-MS and nanoLC-MS/MS with Lys-C digest. Peptide-containing fractions were combined, purified using analytical HPLC, lyophilised, and stored at −80 °C. After purification peptides were characterised using MALDI-TOF MS.

Table S17: Optimised alkylation reaction conditions.

Peptide	Alkylating Reagent	Temperature	Molar Ratio (alkylating agent : peptide)
**P1**	(2-bromoethyl)-trimethylammonium bromide	50 °C	300:1
**P2**	(2-bromoethyl)-dimethylammonium bromide	rt	50:1
**P3**	(2-bromoethyl)-diethylammonium bromide	rt	50:1
**P4**	*N*-(2-bromoethyl)-*N*-isopropylpropan-2-amine hydrobromide **(1)**	rt	50:1
**P5**	1-(2-bromoethyl)pyrrolidine hydrobromide **(2)**	rt	50:1
**P6**	1-(2-bromoethyl)piperidine hydrobromide **(3)**	rt	50:1
**P7**	4-(2-bromoethyl)morpholine hydrobromide **(4)**	rt	50:1
**P8**	1-(2-bromoethyl)piperazine ditrifluoroacetic acid **(7)**	rt	50:1
**P9**	1-(2-bromoethyl)-4-methylpiperazine dihydrobromide **(8)**	rt	50:1
**P10**	2-(2-bromoethyl)isoindoline hydrobromide **(10)**	rt	50:1
**P11**	2-(2-bromoethyl)-1,2,3,4-tetrahydroisoquinoline hydrobromide **(12)**	rt	50:1
**P12**	1-(2-bromoethyl)pyridinium bromide **(13)**	50 °C	300:1
**P13**	2-(2-bromoethyl)pyridine **(14)**	50 °C	300:1
**P14**	1-(2-bromoethyl)-1*H*-pyrazole **(15)**	50 °C	300:1
**P15**	7a-(chloromethyl)hexahydro-1*H*-pyrrolizine hydrochloride **(19)**	50 ºC	300:1
**P16**	(2*R*,3*R*,4*S*,5*R*,6*R*)-2-(2-bromoethoxy)-6-(hydroxymethyl)tetrahydro-2*H*-pyran-3,4,5-triol **(21)**	50 ºC	300:1

Bacterial Transformation

His-tagged human TRIM24 PHD-Bromo WT was expressed from the plasmid pNIC28–Bsa4, provided by the Structural Genomics Consortium, in competent *Escherichia coli* BL21-Gold (DE3) (Agilent). His_6-SUMO-tagged TRIM24 PHD-Bromo was expressed from the plasmid pETM11-SUMO3GFP, provided by the Structural Genomics Consortium, in competent *E. coli* Mach1-T1^R (Thermo Fisher). GST-tagged human TRIM24 PHD-Bromo was expressed from the plasmid pGTvL1-SGC and purified by.

Table S18: 2× YT medium composition (1 L).

Media	Reagent	Amount required (g/L)
2× YT	Tryptone	16.0
	Yeast extract	10.0
	NaCl	5.0

All media and equipment were sterilised by autoclaving at 121 °C for 20 min prior to use, and standard aseptic technique was used throughout. Competent *E. coli* (40 µL) were thawed on ice and the TRIM24 plasmid (1 µL) was added. The tube was flicked 10 times and incubated on ice for 30 min. The cells were heat shocked in a 42 °C water bath for 45 s and then returned to ice for 5 min. Super optimal medium with catabolic repressor (SOC) (100 µL) was added and the bacteria were incubated at 37 °C for 90 min. Autoclaved 2× YT medium plates containing bacteriological agar (15 g/L) and kanamycin (50 µg/mL) were streaked with cells (140 µL). Plates were inverted, incubated overnight at 37 °C, and then stored at 4 °C until required.

Protein Expression

Autoclaved 2× YT medium (100 mL), containing kanamycin (50 µg/mL), was inoculated with one colony from an agar plate; and incubated overnight at 37 °C with shaking at 250 rpm. 6 × 2 L PYREX® narrow-mouth baffled Tunair™ flasks, containing autoclaved 2× YT medium (1 L) and kanamycin (50 µg/mL), were inoculated with 1% by volume (10 mL) of the overnight starter culture; and incubated at 37 °C with shaking at 180 rpm until an OD_{600} of 0.6–0.8 was reached. Optical density measurements were carried out at regular intervals using a Novaspec® II spectrophotometer at 600 nm, with 2× YT media (supplemented with 50 µg/mL kanamycin) used as the reference. After reaching the desired optical density, cultures were equilibrated to 18 °C, supplemented with aqueous $ZnCl_2$ (0.1 mM), induced by addition of isopropyl-1-thio-β-D-galactopyranoside (IPTG) (0.25 mM), and incubated overnight at 18 °C with shaking at 180 rpm. 20–24 h after induction, bacteria were harvested by centrifugation using an Avanti™ JXN-26 centrifuge (6227 ×g, 4 °C for 20 min).

Protein Purification

His$_6$-TRIM24:

Table S19: IMAC and SEC buffer composition for His$_6$-TRIM24 purification. All buffers were filtered through 0.2 μm filter paper under vacuum prior to use. Gel filtration buffer was degassed for 30 min prior to use. Buffers were stored at 4 ºC.

Buffer (pH 7.6)	[Reagent]			
	HEPES	NaCl	Imidazole	Glycerol
Binding	50 mM	500 mM	5 mM	10% *v/v*
Elution	50 mM	500 mM	500 mM	10% *v/v*
Gel Filtration	50 mM	500 mM	–	–

Table S20: IMAC step gradient elution method 1 for the purification of His$_6$-TRIM24 (flow rate = 1.5 mL/min).

Eluent	% Elution buffer (in binding buffer)	Volume (mL)	[Imidazole] (mM)
1	0	50 (10 CV)	5
2	5	50 (10 CV)	30
3	11	50 (10 CV)	60
4	60	20 (4 CV)	300
5	100	10 (2 CV)	500

Pellets were thawed, resuspended in extraction buffer [1 Sigma*FAST*™ protease inhibitor cocktail tablet EDTA free (Sigma Aldrich) dissolved in 100 mL binding buffer per 20 g pellet), and sonicated (35% amplitude] on ice for 15 min *via* a 5 s burst/10 s pause cycle (5 min total sonication time). The lysate was supplemented with polyethylenimine (PEI) (0.15% *w/v*) and incubated on ice for 15 min. The lysate was clarified by centrifugation (48298 ×g, 4 °C for 30 min), and the supernatant was filtered through a 0.45 μm syringe filter.

The protein was purified using nickel immobilised metal affinity chromatography (Ni-IMAC) at 4 °C on an ÄKTA pure™ system (Cytiva) using a HisTrap™ FF 5 mL column (Cytiva), preloaded with Ni^{2+}. The cell lysate was loaded onto the column (1 mL/min flow rate) and purified protein was eluted in 5 mL fractions using step gradient method 1 (Table S20). The protein was further purified using size exclusion chromatography (SEC) at 4 °C on an ÄKTA pure™ system (Cytiva) using an XK 16/600 gel filtration column (Cytiva) packed with 120 mL Superdex 75 resin. Purified protein was eluted in 5 mL fractions with degassed gel filtration buffer (150 mL) using a flow rate of 1 mL/min.

Untagged TRIM24 (Produced from His$_6$-SUMO-TRIM24):

Table S21: IMAC and SEC buffer composition for His$_6$-SUMO-TRIM24 purification and SUMO tag cleavage. All buffers were filtered through 0.2 µm filter paper under vacuum before use. Gel filtration buffer was degassed for 30 min before use. Buffers were stored at 4 ºC.

**Buffer (pH 8.0)**	**[Reagent] (mM)**			
	**Tris base**	**NaCl**	**Imidazole**	**DTT**
Binding	20	500	20	–
Wash I	20	500	45	–
Wash II	20	500	60	–
Elution I	20	500	300	–
Elution II	20	500	500	–
SUMO cleavage	20	150	–	1
Gel Filtration	10	100	–	5

Table S22: IMAC step gradient elution method 2 for the purification of His$_6$-SUMO-TRIM24.

**Eluent**	**Buffer**	**Volume (mL)**	**[Imidazole] (mM)**
1	Binding	40 (20 CV)	20
2	Wash I	20 (10 CV)	45
3	Wash II	20 (10 CV)	60
4	Elution I	7 (3.5 CV)	300
5	Elution I	7 (3.5 CV)	300
6	Elution I	7 (3.5 CV)	300
7	Elution II	7 (3.5 CV)	500

Table S23: IMAC step gradient elution method 3 for the purification of cleaved TRIM24.

**Eluent**	**Buffer**	**Volume (mL)**	**[Imidazole] (mM)**
1	Binding	7 (3.5 CV)	20
2	Binding	7 (3.5 CV)	20
3	Wash I	7 (3.5 CV)	45
4	Wash II	7 (3.5 CV)	60
5	Elution I	7 (3.5 CV)	300
6	Elution II	7 (3.5 CV)	500

Pellets were thawed, resuspended in binding buffer (200 mL), and supplemented with benzonase nuclease (40 µL) and protease inhibitor cocktail set III (200 µL). The cells were sonicated (35% amplitude) on ice for 15 min *via* a 5 s burst/10 s pause cycle (5 min total sonication time) and the lysate was clarified by centrifugation (53248 ×g, 4 °C for 1 h).

The protein was purified using manual Ni-IMAC at 4 ºC using Ni Sepharose™ 6 FF resin (Cytiva). The supernatant was added to the resin (2 mL, pre-equilibrated with 40 mL binding buffer) and incubated for 2 h before loading onto a gravity flow column. Purified protein was eluted using step gradient method 2 (Table S22). Fractions containing purified protein were combined, supplemented with His_6-SENP1 protease (1:300, protease:protein), and dialysed into SUMO cleavage buffer overnight using a Slide-A-Lyzer™ G3 dialysis cassette (3.5K MWCO, Thermo Fisher Scientific). Fractions containing the cleaved protein were combined and repurified using Ni-IMAC as previously described using step gradient method 3 (Table S23). The protein was then further purified by SEC, as previously described above.

Protein Crystallography

TRIM24 Crystallisation in the free State

Purified untagged TRIM24 (produced from His_6-SUMO-TRIM24) was concentrated to 21.8 mg/mL and crystallisation was carried out using the sitting-drop vapour-diffusion technique. Crystallisation plates were prepared using a mosquito® HTS Nanolitre Liquid Handler with a drop size of 150 nL and drop ratios of 2:1, 1:1, and 1:2 (protein:reservoir). Crystals were grown at 20 °C using a fine screen based on previous crystallisation conditions.[15]

TRIM24 Co-crystallisation

Compounds (40 mM DMSO stock) were mixed with purified untagged TRIM24 (21.8 mg/mL) in a 1:15 ratio (compound : protein). Crystallisation was carried out using the sitting-drop vapour-diffusion technique. Protein compound mixtures were incubated on ice for 1 h, before being clarified by centrifugation (15000 rpm, 4 °C for 10 min). Crystallisation was carried out using the sitting-drop vapour-diffusion technique with a reservoir solution of 1.8 M ammonium sulfate, 0.1 M HEPES (pH 7.3), and 5% *v/v* PEG-400. Crystallisation plates were prepared using a mosquito® HTS Nanolitre Liquid Handler with a drop size of 150 nL and drop ratios of 2:1, 1:1, and 1:2 (protein:reservoir) with crystal seeding (20 µL) carried out as required. Crystals were grown for 2 months at 20 °C.

Diffraction Data Collection and Structure Refinement

Crystals of interest were mounted and vitrified in liquid nitrogen. Diffraction data were collected at Diamond Light Source (DLS) beamline I03. Diffraction data were processed at DLS using xia2-DIALS auto processing. Crystal structures were solved by molecular replacement using the published apo crystal structure (PDB ID: 3O33) and refined using the REFMAC5 and Coot programmes within the CCP4*i*2 software.

AlphaScreen®

General AlphaScreen® Experimental

Table S24: AlphaScreen® buffer (1×, pH 7.6) composition.

Reagent	Mass required per 50 mL	Concentration
HEPES	298 mg	25 mM
NaCl	292 mg	100 mM
CHAPS	25 mg	0.05% *w/v*
BSA	50 mg	0.1% *w/v*

AlphaScreen® buffer was prepared as a 5× stock solution without bovine serum albumin (BSA) and stored at 4 °C until required. Prior to carrying out the assay, an aliquot of buffer was adjusted to pH 7.6 using aqueous NaOH (1 M), supplemented with BSA (0.1% *w/v*), diluted to a 1× solution, and filtered through a 0.22 µm syringe filter. An electronic multi-channel pipette (Thermo Fisher) was used to dispense the protein, peptides, and compounds into an AlphaPlate™-384SW (Perkin Elmer) microplate. A PHERAstar FS (BMG LABTECH) microplate reader was used to read the plate (Excitation: 680 nm, 0.30 s; Emission: 570 nm, 0.60 s; Temperature: 25 °C; Gain: 3000). Ni^{2+} chelate/Anti-GST acceptor and streptavidin donor beads (Perkin Elmer) were used for the signal response and dose response assays. Ni^{2+} chelate acceptor and glutathione donor beads (Perkin Elmer) were used for the ternary complex assay. Incubation steps involve sealing the plate with an aluminium plate seal and shaking on a plate oscillator at 300 rpm in the dark for a specified duration at rt. Dose response curves were obtained in triplicate by fitting the data to a four-parameter equation (Equation 2) in GraphPad Prism.

$$Emission = Bottom + \frac{Top - Bottom}{1 + 10^{[(Hill\ slope)(log\ IC_{50} - log\ x)]}} \qquad \text{(Eq. 2)}$$

$$(x = [compound])$$

Signal-response Assay

Biotinylated peptides (5 μL) were dispensed into wells as 1:2 serial dilutions in AlphaScreen® buffer. His_6-TRIM24 (7 μL) or GST-TRIM24 (7 μL) was dispensed into wells to give the desired concentrations and plates were incubated for 1 h. Assay beads (8 μL, 10 μg/mL FAC) were subsequently added under dark conditions to attain a final assay volume of 20 μL. Plates were incubated in the plate reader at 25 °C for 1 h before being read.

Dose-response Assay

Compounds (5 μL) were dispensed into wells as 1:2 serial dilutions in AlphaScreen® buffer supplemented with DMSO (1% FAC). His_6-TRIM24–biotinylated peptide mixes (7 μL, 1% DMSO, Table S25) were dispensed into wells and plates were incubated for 1 h. Assay beads (8 μL, 10 μg/mL FAC, 1% DMSO) were subsequently added under dark conditions to attain a final assay volume of 20 μL. Plates were incubated in the plate reader at 25 °C for 1 h before being read.

Table S25: TRIM24-biotinylated peptide mix FACs determined from signal response assays.

Peptide	Peptide FAC (nM)	TRIM24 FAC (nM)
$H3_{(1–27)}K9Me_3$ (B1)	30	50
$H3_{(1–27)}K18Ac$ (B2)	8	25
$H3_{(1–27)}K9Me_3K18Ac$ (B3)	8	25

Ternary Complex Detection Assay

Compounds (5 μL) were dispensed into wells as 1:2 serial dilutions in AlphaScreen® buffer supplemented with DMSO (1% FAC). TRIM24 protein mixes (7 μL, 25 nM His_6-TRIM24, 25 nM GST-TRIM24, 1% DMSO) were dispensed into wells and plates were incubated for 1 h. Assay beads (8 μL, 10 μg/mL FAC, 1% DMSO) were subsequently added under dark conditions to attain a final assay volume of 20 μL. Plates were incubated in the plate reader at 25 °C for 1 h before being read. An His_6-GST construct was used as a technical positive control. 7 μL of buffer, supplemented with 1% DMSO, was used in place of the TRIM24 protein mix for the technical positive control.

TruHits Assay

Compounds (5 μL) were dispensed into wells as 1:2 serial dilutions in AlphaScreen® buffer supplemented with DMSO (1% FAC). TruHits assay beads (15 μL, 4.7 μg/mL FAC, 1% DMSO) were subsequently added under dark conditions to attain a final assay volume of 20 μL. Plates were incubated in the plate reader at 25 °C for 30 min before being read.

BROMO*scan*

This assay was carried out by Eurofins DiscoverX Corporation (San Diego, USA) using the following protocol:

T7 phage strains displaying proteins of interest were grown in parallel in 24-well blocks in an *E. coli* host derived from the BL21 strain. *E. coli* were grown to log-phase and infected with T7 phage from a frozen stock (multiplicity of infection = 0.4) and incubated with shaking at 32 °C until lysis (90–150 min). The lysates were centrifuged (5000 ×*g*) and filtered (0.2 µm) to remove cell debris. Streptavidin-coated magnetic beads were treated with biotinylated small molecule or acetylated peptide ligands for 30 min at room temperature to generate affinity resins for bromodomain assays. The liganded beads were blocked with excess biotin and washed with blocking buffer (SeaBlock (Pierce), 1 % BSA, 0.05 % Tween 20, 1 mM DTT) to remove unbound ligand and to reduce non-specific phage binding. Binding reactions were assembled by combining bromodomains, liganded affinity beads, and test compounds in 1× binding buffer (17% SeaBlock, 0.33× PBS, 0.04% Tween 20, 0.02% BSA, 0.004% Sodium azide, 7.4 mM DTT). Test compounds were prepared as 1000× stocks in 100% DMSO. K_d values were determined using an 11-point 3-fold compound dilution series with one DMSO control point. All compounds for K_d measurements are distributed by acoustic transfer (non-contact dispensing) in 100% DMSO. The compounds were then diluted directly into the assays such that the final concentration of DMSO was 0.09%. All reactions were performed in polypropylene 384-well plates with a final assay volume of 20 µL. The assay plates were incubated at room temperature with shaking for 1 h and the affinity beads were washed with wash buffer (1× PBS, 0.05% Tween 20). The beads were then re-suspended in elution buffer (1× PBS, 0.05% Tween 20, 2 µM non-biotinylated affinity ligand) and incubated at room temperature with shaking for 30 min. The protein concentration in the eluates was measured using qPCR.

$$Response = Background + \frac{Signal - Background}{1 + (\frac{K_d}{x})^{Hill\ slope}} \qquad \text{(Eq. 3)}$$

$$(x = [compound])$$

Binding constants (K_d) were calculated with a standard dose-response curve using the Hill equation (Equation 3). The Hill Slope was set to −1. Curves were fitted using a non-linear least square fit with the Levenberg-Marquardt algorithm.

Surface Plasmon Resonance (SPR)

General SPR Experimental

Table S26: 1× HBS-EP buffer (pH 7.4) composition. The buffer was filtered through 0.2 µm filter paper under vacuum before use.

Reagent	Concentration
HEPES	10 mM
NaCl	150 mM
EDTA	3 mM
P20 surfactant	0.005% (*v*/*v*)

SPR was carried out on a Biacore™ T200 sensor using a C1 or CM5 sensor chip (Cytiva) and 1× HBS-EP (pH 7.4, Cytiva) as the running/analyte binding buffer. Protein (50 µg/mL) was covalently immobilised onto flow cell (FC) surfaces in aqueous NaOAc buffer (10 mM, pH 4.0) at 25 ºC and a flow rate of 10 µL/min. Immobilisation was achieved by activating carboxymethyl groups on the chip surface using a 1:1 ratio of 1-ethyl-3-(3-dimethylaminopropyl)carbodiimide (EDC) (400 mM) : *N*-hydroxysuccinimide (NHS) (100 mM) for 600 s, followed by covalent capture of the protein on the surface for 12–100 s, and capping of excess activated carboxylates with ethanolamine-HCl (1 M, pH 8.5) for 600 s. A reference FC was activated using EDC/NHS and directly capped with ethanolamine. Each analyte sample concentration was injected continuously for a set amount of time and allowed to flow over the chip surface (association phase). Injection was then stopped and replaced with a continuous flow of running buffer (dissociation phase). Lastly a conditioning step was carried out, using an injection of running buffer before the next analyte injection. Sensogram report points were collected at 10 Hz. For all experiments, the reference FC and the previous conditioning step were subtracted from the active FC sensograms to obtain the bound ligand concentration, expressed in response units (RU), as a function of time.

Binding Saturation Experiment (Equilibrium Analysis)

Two-fold serial dilutions of the analyte were prepared in HBS-EP buffer from an aqueous stock solution. His_6-TRIM24 was immobilised (>1000 RU) onto the surface of 3 flow cells. Samples were injected and allowed to reach equilibrium before dissociation (association: 30 s, dissociation: 120 s, flow rate: 50 µL/min, 15 ºC). Binding saturation curves were obtained by plotting the equilibrium response (curve plateau) against the analyte concentration and fitting the data using a one-site (1:1) total binding model (Equation 4) in GraphPad Prism.

$$Response = (\frac{B_{max}x}{K_d + x}) + x(NS) + Background \quad (\text{Eq. }4)$$

$$(x = [compound], B_{max} = maximum\ signal, NS = non-specific\ binding)$$

Multi-cycle Kinetics Experiment (Kinetic Analysis)

Two or three-fold serial dilutions (as indicated) of the analyte were prepared in HBS-EP buffer from a DMSO stock solution (0.1 % DMSO FAC). His_6-TRIM24 was immobilised (500–1000 RU) onto the surface of three flow cells, followed by sample injection and dissociation (34 s - association: 30 s, dissociation: 300 s, flow rate: 75 µL/min, 15 ºC; PDC6 (compound **52**) - association: 90 s, dissociation: 900 s, flow rate: 75 µL/min, 15 ºC). One-phase association (Equation 5) and dissociation (Equation 6) models were used to fit the data in GraphPad Prism to obtain values for k_{off} (s^{-1}) and k_{obs} (s^{-1}) for each concentration. These values were then substituted into Equation 7 to obtain values for k_{on} ($M^{-1}\ s^{-1}$) for each concentration. Mean k_{off} and k_{on} values were obtained by taking the values from the fitted curves for each concentration, before averaging across the replicates. The law of mass action (Equation 8) was used to calculate a K_d (M).

$$Response = (R_0 - NS)exp(-k_{off}t) + NS \quad (\text{Eq. }5)$$

$$Response = R_0 + (R_{max} - R_0)(1 - exp(-k_{obs}t)) \quad (\text{Eq. }6)$$

$$k_{on} = \frac{k_{obs} - k_{off}}{[compound]} \quad (\text{Eq. }7)$$

$$K_d = \frac{k_{off}}{k_{on}} \quad (\text{Eq. }8)$$

$$(R_0 = response\ at\ t_0,\ R_{max} = plateau\ response, NS = non-specific\ binding)$$

In vitro Cellular Assays

Cell Lines

Table S27: Description and origin of cell lines used in the cell permeability assay.

Cell Line	Description	Origin
**MCF-7**	Human Breast adenocarcinoma, luminal A, ER positive	ATCC® (ATCC-HB-22)

Table S28: Description and origin of cell lines used in the MTT, proliferation, and clonogenic assays.

Cell Line	Description	Origin
**CAL-51**	Human Breast carcinoma, triple negative	Prof. Eileen Parkes, University of Oxford
**MCF-7**	Human Breast adenocarcinoma, luminal A, ER positive	Prof. Katherine Vallis, University of Oxford
**BT-474**	Human Breast adenocarcinoma, triple positive	Prof. Adrian Harris, University of Oxford

Cell Permeability Assay

This assay was carried out using an adapted version of a previously reported procedure.[16,17]

Table S29: 1× HBS buffer (pH 7.4) + protease inhibitors. The buffer was filtered through 0.2 μm filter paper under vacuum before use.

Reagent	Concentration
HEPES	10 mM
NaCl	150 mM
Sigma*FAST*™ protease inhibitor cocktail tablet EDTA free (Sigma Aldrich)	1 tablet per 400 mL

MCF7 cells were cultured in Dulbecco's modified Eagle's medium (DMEM), supplemented with 10% fetal bovine serum (FBS) and 1% penicillin-streptomycin (PS) at 37°C, 5% CO_2 in a humidified incubator. Cells were routinely mycoplasma tested using a HEK-Blue™ detection kit (Invivogen). 100000 cells were seeded in cell culture glass dishes (60 × 15 mm) and allowed to adhere for 24 h, before changing the medium to DMEM. Cells were treated with the compounds (20 μM) or DMSO control and incubated for 6 h. Cells were washed three times with HEPES-buffered saline (HBS) (pH 7.4), supplemented with a Sigma*FAST*™ protease inhibitor cocktail tablet EDTA free (Sigma Aldrich) (washes were collected as a control), and centrifuged at 15,000 rpm for 15 min (the supernatant was collected as a control). The remaining cell pellet was stored at −80 °C overnight. Cell pellets were resuspended in MeCN/H_2O (1:1, 200 uL) and transferred to 15 mL Falcon tubes, before being sonicated (35% amplitude) on ice for 15 min *via* a 5 s burst/10 s pause cycle (5 min total sonication time). The cell lysates were transferred to 1.5 mL Eppendorf tubes and clarified by centrifugation at 15,000 rpm for 15 min. The supernatant was then analysed using LC-MS (50 μL injection). Blank subtraction was carried out using the DMSO control sample.

Cell Culture

Cells were grown in high glucose DMEM medium (Life Technologies Ltd., 41965062) supplemented with 10% FBS (Merck Life Sciences U.K. Ltd., F7524). Unless otherwise stated, cells were cultured in a humidified incubator at 37 °C and 5% CO_2. Cell lines were passaged by trypsinisation (Trypsin-EDTA solution 1×, Merck Life Sciences U.K. Ltd., T3924), and by diluting the cells into a culture flask at the desired density in complete media. Cell lines were routinely mycoplasma tested (MycoAlert Mycoplasma Detection Kit, Lonza, LT07-418; MycoAlert Assay Control Set, Lonza, LT07-518) and were found to be negative.

MTT Assay

Cells were seeded and allowed to adhere for 6 h in plastic clear-bottomed 96-well plates in a total volume of 200 µL/well complete medium. Optimal seeding numbers for each cell line were established (BT-474 8000 cells/well, CAL-51 4000 cells/well). The media was replaced with 200 µL of fresh media containing the compound of interest at 10 µM concentration, and the plates were incubated at 37 °C for 16 h. At the end of treatment, without removing the media, 0.5 mg/mL MTT reagent (Life technologies Ltd., M6494) was added, and incubated for 3 h at 37 °C, protected from light. The resulting formazan (purple) crystals were solubilised by replacing the media with 100 µL of DMSO (Merck Life Sciences U.K. Ltd., D8418), and leaving for 15 min at 37 °C, protected from light. Absorbance at 570 nm was measured using a POLARstar Omega plate reader (BMG LABTECH). Data were expressed as percentage cell viability relative to the DMSO control.

Cell Proliferation Assay

Cells were seeded and allowed to adhere for 6 h in 24-well plates (CAL-51 4000 cell/well, MCF-7 10000 cells/well). The media was replaced with 300 µL of fresh media containing the compound of interest (10 µM), and the plates were incubated at 37 °C. For single treatment assays, counting was repeated each day in triplicate without media replacement. For multiple treatment assays, counting was repeated each day in triplicate before replacing the media with 300 µL of fresh media containing the compound of interest (10 µM).

Clonogenic Assay

Pretreated cells from the proliferation assays were seeded at low density (CAL51 250 cells/well, MCF7 500 cells/well) in 6 well plates and incubated at 37 °C until colony formation. Colonies were stained with 2% crystal violet diluted in 50% methanol and 20% ethanol, and counted manually. Plating efficiency was determined by dividing the total number of colonies by the seeding number. The surviving fraction was then calculated by dividing the plating efficiency for treatment by the plating efficiency for the DMSO control.

5 Chemistry Experimental Section

General Chemistry Experimental

Compound Names were generated by ChemDraw 22.0.0 following IUPAC nomenclature.

Reagents and Solvents used were of commercially available reagent grade and were used without further purification, unless stated. All non-aqueous reactions were carried out under a nitrogen atmosphere in a flame-dried flask unless otherwise stated. Anhydrous solvents were obtained from an MBRAUN Solvent Purification System 5 and stored under an argon atmosphere over 3 Å molecular sieves. Degassed solvents were obtained by passing a steady stream of argon through the liquid, under sonication for 30 min. Where possible reactions were quenched/worked up after TLC analysis indicated complete consumption of the starting material. Concentration *in vacuo* was performed using a Buchi™ rotary evaporator with a water bath set at 40 °C, unless otherwise stated. Brine refers to saturated aqueous NaCl. Celite® refers to Celite® 545 filter aid, treated with sodium carbonate, flux-calcined (Sigma Aldrich). Lyophilisation was carried out on a Christ Alpha 3-4 LSCbasic Laboratory freeze dryer. Samples were dissolved in H_2O/MeCN and flash frozen in liquid nitrogen, before being lyophilised overnight.

^{1}H NMR spectra were measured on a Bruker AVIIIHD 400 (400 MHz), Bruker AVIIIHD 500 (500 MHz), or Bruker NEO 600 (600 MHz) spectrometer. Chemical shifts (δ) are quoted in parts per million (ppm) relative to tetramethylsilane (δ_H = 0.00 ppm). Spectra were calibrated using residual protic solvent peaks and data provided by Fulmer *et al.*.[18] NMR data were processed using Bruker Topspin 4.3.0 software. ^{1}H NMR spectra are reported as follows: chemical shift (number of protons, multiplicity (s (singlet); br s (broad singlet); d (doublet); t (triplet); q (quartet); m (multiplet) or combinations thereof), coupling constants *J* (to the nearest 0.1 Hz, with identical coupling constants averaged), assignment). Spectra were assigned using COSY, HSQC, and HMBC experiments.

^{13}C NMR spectra were measured on a Bruker AVIIIHD 500 (126 MHz) or Bruker NEO600 (151 MHz) spectrometer and are broadband proton decoupled. Chemical shifts (δ) are quoted in parts per million (ppm) relative to tetramethylsilane (δ_C = 0.00 ppm). The spectra were calibrated using the solvent peak and data provided by Fulmer *et al.*.[18] NMR data were processed using Bruker Topspin 4.3.0 software. The multiplicity of each signal is singlet unless otherwise stated. Spectra were assigned using HSQC and HMBC experiments.

^{19}F NMR spectra were recorded on a Bruker AVIIIHD 500 (470 MHz) or Bruker NEO600 (565 MHz) spectrometer and are broadband proton decoupled. Chemical shifts (δ) are quoted in parts per million (ppm). NMR data were processed using Bruker Topspin 4.3.0 software. The multiplicity of each signal is singlet unless otherwise stated.

Mass Spectra were acquired on either an Agilent 6120 (low resolution), Waters LCT Premier XE bench-top orthogonal acceleration TOF LCMS (low resolution), Bruker microToF (high resolution), or Waters BioAccord LCMS (high resolution) spectrometer using electrospray ionisation (ESI) from solutions of MeOH, MeCN or H_2O. *m/z* values are reported in Daltons and followed by their percentage abundance in parentheses.

Liquid Chromatography-Mass Spectrometry (LCMS) was carried out on an Agilent 1260 Infinity II system with a Quaternary LC Pump, DADWR, and InfinityLab LC/MSD. An Agilent InfinityLab Poroshell 120 SB-C18 [2.7 μm, 2.1 mm × 50 mm] column was used with a constant flow rate of 0.4 mL min^{-1} and a gradient method of 10 min from 5–95% MeCN/H_2O +0.1% formic acid with a 5 min hold. Samples were prepared by dissolving in MeOH, MeCN or H_2O, and filtered. LCMS data were processed using Agilent OpenLab ChemStation software.

Melting Points were determined using a Griffin capillary tube melting point apparatus and are uncorrected. The crystallisation solvent is given in parentheses.

Infrared Spectra were obtained from thin films or neat samples, using a diamond ATR module. The spectra were recorded on a Bruker Tensor 27 spectrometer. Absorption maxima ($\tilde{\nu}_{max}$) are reported in wavenumbers (cm^{-1}) and are reported as s (strong), m (medium), w (weak) or br (broad).

Specific Optical Rotations were measured using a Schmidt Haensch Unipol polarimeter, using a sodium lamp at 589 nm and a path length of 1.0 dm. The concentration (*c*) is expressed in g/100 mL (equivalent to g/0.1 dm^3). Specific rotations are quoted as an average of 10 measurements. They are denoted $[\alpha]_D^T$ and are given in implied units of 10^{-1} ° cm^2 g^{-1} (where T = 25 °C).

Analytical HPLC was performed on an Agilent 1260 Infinity II system with a Quaternary LC Pump, DADWR and Agilent Infinity Fraction Collector. For determination of purity, an Agilent InfinityLab Poroshell 120 EC-C18 [4 μm, 4.6 mm × 150 mm] reverse phase column was used with a constant flow rate of 1.0 mL min^{-1} and gradient method of 10 min from 5–95% MeCN/H_2O +0.1% formic acid with a 5 min hold. Samples were prepared by dissolving in MeOH, MeCN or H_2O, and filtered. HPLC data were processed using Agilent OpenLab ChemStation software.

Semi-Preparative Reverse Phase HPLC was performed on an Agilent 1260 Infinity II Semi-Prep system with a binary LC pump, DADWR and Agilent Infinity Fraction Collector. Samples were prepared by dissolving in MeOH, MeCN, H_2O, or DMF, and filtered. Injection volumes of 500–1500 μL were used depending upon the resolution of the chromatograms.

Table S30: Gradient Method 1.

Time (min)	%H_2O (+0.1% formic acid)	%MeCN (+0.1% formic acid)
0.00	95	5
1.00	95	5
11.00	5	95
18.00	5	95

Table S31: Gradient Method 2.

Time (min)	%H_2O (+0.1% formic acid)	%MeCN (+0.1% formic acid)
0.00	95	5
1.00	95	5
11.00	5	95
15.00	5	95

Table S32: Gradient Method 3.

Time (min)	%H_2O (+0.1% TFA)	%MeCN (+0.1% TFA)
0.00	95	5
2.00	95	5
2.01	80	20
7.00	60	40
10.00	5	95

Table S33: Gradient Method 4.

Time (min)	%H_2O (+0.1% formic acid)	%MeCN (+0.1% formic acid)
0.00	95	5
4.00	95	5
4.01	50	50
10.00	5	95
15.00	5	95

Analytical Thin-Layer Chromatography (TLC) was carried out on normal phase or reverse phase Merck silica (SiO_2) 60 F_{254} aluminium-supported thin layer chromatography sheets. Visualisation was carried out by absorption of UV light (λ_{max} = 254 nm) or thermal development after staining with an ethanolic ninhydrin solution, basic aq. $KMnO_4$ or acidic aq. 2,4-dinitrophenylhydrazine.

Normal Phase Flash Column Chromatography was carried out manually on Merck Geduran® silica gel 60 (40–63 µm) or on an automated Biotage® Selekt system with Biotage® Sfär silica columns.

General Procedures

General Bromination Procedure: The denoted amine (1.0 eq.) was added dropwise to aqueous HBr (48% *w/w*) at 0 °C. The reaction solution was stirred and heated under reflux at 135 °C for 20 h. Excess HBr was removed by distillation at atmospheric pressure under a stream of nitrogen. The residue was cooled to rt, dissolved in MeOH (10 mL), and concentrated *in vacuo*. The residue was washed with ^{i}PrOH (30 mL) to yield the brominated product.

General Amide Coupling Procedure 1: Anhydrous DIPEA (3.0 eq.) and HATU (1.0 eq.) were added to a solution of the carboxylic acid (1.0 eq.) and the denoted amine (1.0 eq.) in anhydrous DMF (5 mL) at rt. The reaction mixture was stirred at rt for 24 h. The reaction mixture was diluted with EtOAc (25 mL) and washed with aqueous LiCl (0.5 M, 25 mL), aqueous citric acid (10% *w/v*, 25 mL), and brine (25 mL). The organic component was dried (Na_2SO_4), filtered, and concentrated *in vacuo* to yield the crude amide coupled product.

General Amide Coupling Procedure 2: Anhydrous DIPEA (5.0 eq.) and HATU (1.0 eq.) were added to a stirring solution of the corresponding crude carboxylic acid (1.0 eq.) in anhydrous DMF (3 mL). A solution of the corresponding crude amine (1.0 eq.) in anhydrous DMF (2 mL) was added dropwise and the reaction mixture was stirred at rt for 24 h. The reaction mixture was diluted with EtOAc (25 mL) and washed with aqueous LiCl (0.5 M, 25 mL), aq. citric acid (10% *w/v*, 25 mL), and brine (25 mL). The organic component was dried (Na_2SO_4), filtered, and concentrated *in vacuo* to yield the crude amide coupled product.

General Amide Coupling Procedure 3: HATU (1.0 eq.) was added to a stirring solution of the deprotected amine (1.0 eq.), peptide **46** (1.0 eq.), and anhydrous DIPEA (13.2 µL, 75.5 µmol, 5.0 eq.) in anhydrous CH_2Cl_2 (1 mL) at rt. The reaction solution was stirred at rt for 24 h. The reaction solution was concentrated *in vacuo*.

General Boc Deprotection Procedure: The corresponding Boc protected amine (1.0 eq.) was dissolved in a solution of HCl in dioxane (4 M, 1 mL) and stirred at rt for 24 h. The reaction mixture was concentrated *in vacuo* to yield the crude amine as a HCl salt.

General Acetylation Procedure: The crude amine was dissolved in CH_2Cl_2 (2 mL) and NEt_3 (2.0 eq.) was added. The reaction solution was cooled to 0 °C and acetyl chloride (1.0 eq.) was added dropwise. The reaction solution was warmed to rt and stirred for 20 min. The reaction solution was quenched with H_2O (500 µL) and concentrated *in vacuo* to yield the crude acetylated product.

General ^{t}Bu Ester Deprotection Procedure: The denoted ^{t}Bu carboxylate ester (1.0 eq.) was dissolved in a solution of HCl in dioxane (4 M, 1 mL) at rt. The reaction solution was stirred at rt for 24 h and concentrated *in vacuo.* The residue was dried overnight under high vacuum to yield the crude carboxylic acid.

General Fmoc Deprotection Procedure: The denoted Fmoc protected amine was dissolved in a solution of piperidine in anhydrous DMF (20% *v/v*, 1 mL) and stirred at rt for 1 h. The reaction solution was concentrated *in vacuo*. The crude amine was purified using semi-preparative reverse phase HPLC (gradient method 2, 20 mL min^{-1}, Agilent Prep C18 column [5 µm, 21.2 mm × 50 mm]) and lyophilised to yield the purified amine as a diformate acid salt.

Global Deprotection Procedure: The denoted protected PDC (1 eq.) was dissolved in a solution of TFA/TIPS/H_2O (38:1:1, 1 mL) and stirred at rt for 4 h. The reaction solution was concentrated *in vacuo* to yield the crude product.

Synthesis and Characterisation of Compounds 1–56

(1) *N*-(2-Bromoethyl)-*N*-isopropylpropan-2-amine hydrobromide

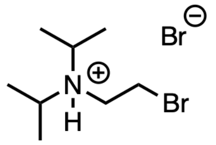

N,N-Diisopropylaminoethanol (1.00 mL, 5.69 mmol, 1.0 eq.) was reacted with aqueous HBr (48% *w/w*, 20 mL) according to the general bromination procedure, to yield the title compound as a colourless crystalline solid (647 mg, 39%): R_f 0.28 (MeOH/CH_2Cl_2, 1:9); m.p. 129–132 °C (from MeOH) [lit.[19] 141 °C (from acetone), lit.[20] 140–142 °C]; ^{1}H NMR (600 MHz, $CDCl_3$) δ_H 10.97 (br s, 1H), 4.02–3.92 (m, 2H), 3.78–3.62 (m, 2H), 3.38–3.28 (m, 2H), 1.61 (d, *J* 6.1 Hz, 6H), 1.49 (d, *J* 6.1 Hz, 6H); ^{13}C NMR (151 MHz, $CDCl_3$) δ_C 55.7, 49.0, 24.6, 18.8, 17.2; $\overline{\nu}_{max}$ (thin film)/cm^{-1} 2925 (w), 2612 (w), 2506 (w); LRMS (ESI$^+$) *m/z* 208 ([M(^{79}Br)–HBr+H]$^+$, 100%), 210 ([M(^{81}Br)–HBr+H]$^+$, 93%); HRMS (ESI$^+$) *m/z* [Found: 208.0696, $C_8H_{19}N^{79}Br$ requires [M–HBr+H]$^+$ 208.0695].

(2) 1-(2-Bromoethyl)pyrrolidine hydrobromide

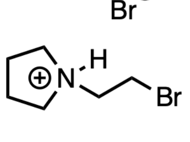

1-(2-Hydroxyethyl)pyrrolidine (1.00 mL, 8.55 mmol, 1.0 eq.) was reacted with aqueous HBr (48% *w/w*, 20 mL) according to the general bromination procedure to yield the title compound as a colourless crystalline solid (1.35 g, 61%): R_f 0.42 (MeOH:CH_2Cl_2, 1:9); m.p. 162–165 °C (from

MeOH); ^{1}H NMR (600 MHz, $CDCl_3$) δ_H 11.87 (br s, 1H), 3.95–3.81 (m, 4H), 3.60–3.50 (m, 2H), 3.00–2.86 (m, 2H), 2.32–2.06 (m, 4H); ^{13}C NMR (151 MHz, $CDCl_3$) δ_C 56.2, 54.3, 23.4, 23.0; $\overline{\nu}_{max}$ (thin film)/cm^{-1} 2947 (w), 2846 (w), 2666 (w), 2583 (w), 2490 (w), 1453 (w); LRMS (ESI$^+$) *m/z* 178 ([M(^{79}Br)–HBr+H]$^+$, 96%), 180 ([M(^{81}Br)–HBr+H]$^+$, 100%); HRMS (ESI$^+$) *m/z* [Found: 178.0227, $C_6H_{13}N^{79}Br$ requires [M–HBr+H]$^+$ 178.0226], [Found: 180.0205, $C_6H_{13}N^{81}Br$ requires [M–HBr+H]$^+$ 180.0205].

(3) 1-(2-Bromoethyl)piperidine hydrobromide

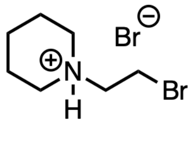

1-(2-Hydroxyethyl)piperidine (1.00 mL, 7.53 mmol, 1.0 eq.) was reacted with aqueous HBr (48% *w/w*, 20 mL) according to the general bromination procedure to yield the title compound as a colourless crystalline solid (1.61 g, 78%): R_f 0.55 (MeOH:CH_2Cl_2, 1:9); m.p. 226–229 °C (from MeOH) [lit.[21] 238 °C, lit.[22] 242–244 (from EtOH)]; ^{1}H NMR (600 MHz, $CDCl_3$) δ_H 11.65 (br s, 1H), 3.94–3.88 (t, *J* 7.3 Hz, 2H), 3.68–3.60 (m, 2H), 3.46–3.38 (m, 2H), 2.84–2.74 (m, 2H), 2.39–2.26 (m, 2H), 1.98–1.86 (m, 3H), 1.52–1.42 (m, 1H); ^{13}C NMR (151 MHz, $CDCl_3$) δ_C 58.3, 53.9, 22.6, 22.0, 21.9; $\overline{\nu}_{max}$ (thin film)/cm^{-1} 2922 (w); 2660 (w), 2575 (w), 2547 (w), 1459 (w); LRMS (ESI$^+$) *m/z* 192 ([M(^{79}Br)–HBr+H]$^+$, 30%), 194 ([M(^{81}Br)–HBr+H]$^+$, 51%); HRMS (ESI$^+$) *m/z* [Found: 192.0382, $C_7H_{15}N^{79}Br$ requires [M–HBr+H]$^+$ 192.0382], [Found: 194.0361, $C_7H_{15}N^{81}Br$ requires [M–HBr+H]$^+$ 194.0362].

(4) 4-(2-Bromoethyl)morpholine hydrobromide

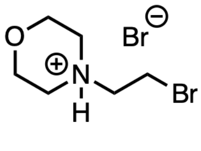

4-(2-Hydroxyethyl)morpholine (1.00 mL, 8.26 mmol, 1.0 eq.) was added dropwise to a solution of triphenylphosphine dibromide (4.18 g, 9.91 mmol, 1.2 eq.) in anhydrous CH_2Cl_2 (50 mL) at 0 °C. The reaction solution was warmed to rt and stirred for 6 h. The precipitate was collected by filtration and washed with CH_2Cl_2 (30 mL) to yield the title compound as an off-white solid (1.59 g, 70%): R_f 0.59 (MeOH/CH_2Cl_2, 1:9); m.p. 201–204 °C (from CH_2Cl_2); ^{1}H NMR (600 MHz, D_6-DMSO) δ_H 10.09 (br s, 1H), 4.04–3.91 (m, 2H), 3.82 (t, *J* 7.1 Hz, 2H), 3.78–3.56 (m, 4H), 3.25–3.06 (m, 2H), 3.16* (m, 2H); ^{13}C NMR (151 MHz, D_6-DMSO) δ_C 63.1, 56.2, 51.1, 23.8; $\overline{\nu}_{max}$ (thin film)/cm^{-1} 2971 (w), 1114 (s), 873 (s); LRMS (ESI$^+$) *m/z* 194 ([M(^{79}Br)–HBr+H]$^+$, 64%), 196 ([M(^{81}Br)–HBr+H]$^+$, 67%); HRMS (ESI$^+$) *m/z* [Found: 194.0174, $C_6H_{13}NO^{79}Br$ requires [M–HBr+H]$^+$ 194.0175], [Found: 196.0152, $C_6H_{13}NO^{81}Br$ requires [M–

HBr+H]+ 196.0155]. The spectroscopic data are in good agreement with the literature values.[23]

*Peak obscured by NMR solvent peak and was assigned by HSQC.

(5) *tert*-Butyl 4-(2-hydroxyethyl)piperazine-1-carboxylate

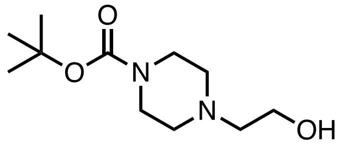

Di-*tert*-butyl dicarbonate (3.56 g, 16.3 mmol, 1.0 eq.) was added portion wise to a solution of 1-(2-hydroxyethyl)piperazine (2.00 mL, 16.3 mmol, 1.0 eq.) in THF (30 mL) at 0 °C. The reaction solution was stirred at 0 °C for 2 h after which time the solvent was evaporated *in vacuo* and the residue was triturated with petroleum ether 40–60 °C. The mixture was filtered, and the filtrate was concentrated *in vacuo*. The residue was crystalised overnight at –20 °C to yield the title compound as a colourless crystalline solid (3.71 g, 99%): R_f 0.35 (MeOH:CH_2Cl_2, 1:9); m.p. 31–34 °C (from petroleum ether 40–60 °C) [lit.[24] 45–52 °C]; ^{1}H NMR (600 MHz, $CDCl_3$) δ_H 3.62 (t, *J* 5.4 Hz, 2H), 3.43 (t, *J* 4.8 Hz, 4H), 2.54 (t, *J* 5.4 Hz, 2H), 2.44 (t, *J* 4.8 Hz, 4H), 1.45 (s, 9H); ^{13}C NMR (151 MHz, $CDCl_3$) δ_C 154.8, 79.8, 59.5, 57.9, 52.8, 44.2*, 43.4*, 28.6; LRMS (ESI$^+$) *m/z* 231 ($[M+H]^+$, 57%), 253 ($[M+Na]^+$, 58%), 483 ($[2M+Na]^+$, 100%); HRMS (ESI$^+$) *m/z* [Found: 231.1705, $C_{11}H_{23}N_2O_3$ requires $[M+H]^+$ 231.1703]. The spectroscopic data are in good agreement with the literature values.[24,25]

*Peaks coalesce to form a singlet at 363 K.

(6) *tert*-Butyl 4-(2-bromoethyl)piperazine-1-carboxylate

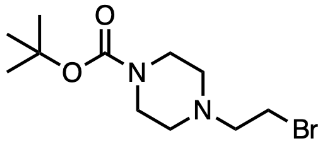

A solution of tetrabromomethane (1.58 g, 4.76 mmol, 1.1 eq.) in anhydrous CH_2Cl_2 (10 mL) was added dropwise to a solution of **5** (1.00 g, 4.34 mmol, 1.0 eq.) and triphenylphosphine (1.23 g, 4.69 mmol, 1.1 eq.) in anhydrous CH_2Cl_2 (10 mL) at 0 °C. The reaction solution was warmed to rt and stirred for 20 h. The solvent was evaporated *in vacuo* and the product was purified using flash silica column chromatography (gradient elution with 10–80% EtOAc/petroleum ether 40–60 °C) to yield the title compound as a yellow crystalline solid (1.15 g, 90%): R_f 0.42 (EtOAc:petroleum ether 40–60 °C, 2:3); m.p. 45–48 °C (from CH_2Cl_2); ^{1}H NMR (600 MHz, $CDCl_3$) δ_H 3.47–3.38 (m, 6H), 2.79 (t, *J* 7.4 Hz, 2H), 2.45 (t, *J* 5.1 Hz, 4H), 1.45 (s, 9H); ^{13}C NMR (151 MHz, $CDCl_3$) δ_C 154.8, 79.9, 60.0, 52.9, 44.1*, 43.2*, 28.9, 28.6;

$\overline{\nu}_{max}$ (thin film)/cm^{-1} 2980 (w), 2814 (w), 1685 (s), 1415 (m), 1243 (m), 1165 (s), 1130 (s), 1000 (m), 772 (w), 657 (w); LRMS (ESI$^+$) *m/z* 293 ([M(^{79}Br)+H]$^+$, 99%), 295 ([M(^{81}Br)+H]$^+$, 100%); HRMS (ESI$^+$) *m/z* [Found: 293.0856, $C_{11}H_{22}N_2O_2{}^{79}Br$ requires [M+H]$^+$ 293.0859], [Found: 295.0836, $C_{11}H_{22}N_2O_2{}^{81}Br$ requires [M+H]$^+$ 295.0839]. The spectroscopic data are in good agreement with the literature values.[26]

*Peaks coalesce to form a singlet at 363 K.

(7) 1-(2-Bromoethyl)piperazine ditrifluoroacetate

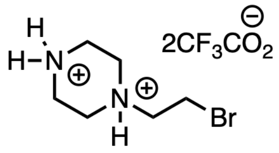

Trifluoroacetic acid (5 mL) was added dropwise to a solution of **7** (1.00 g, 3.41 mmol, 1.0 eq.) in CH_2Cl_2 at 0 °C. The reaction solution was warmed to rt and stirred for 2 h. The reaction solution was concentrated *in vacuo* and the residue was dried under high vacuum overnight. The product was crystalised from Et_2O and collected by filtration to yield the title compound as an off-white solid (1.21 g, 84%): R_f 0.22 (MeOH/CH_2Cl_2, 1:9); m.p. 104–107 °C (from Et_2O); ^{1}H NMR (600 MHz, D_6-DMSO) δ_H 8.98–8.56 (m, 3H), 3.61 (t, *J* 7.1 Hz, 2H), 3.21–3.10 (m, 4H), 2.97 (t, *J* 7.1 Hz, 2H), 2.90–2.77 (m, 4H); ^{13}C NMR (151 MHz, D_6-DMSO) δ_C 57.8, 48.7, 42.2, 28.8; ^{19}F NMR (565 MHz, D_6-DMSO) δ_F –74.40 (s, TFA CF_3); $\overline{\nu}_{max}$ (thin film)/cm^{-1} 3033 (w), 2471 (w), 1668 (s), 1119 (s), 796 (s), 721 (s); LRMS (ESI$^+$) *m/z* 193 ([M(^{79}Br)–2CF_3CO_2H+H]$^+$, 99%), 195 ([M(^{81}Br)–2CF_3CO_2H+H]$^+$, 100%); HRMS (ESI$^+$) *m/z* [Found: 193.0336, $C_6H_{14}N_2{}^{79}Br$ requires [M–2CF_3CO_2H+H]$^+$ 193.0335], [Found: 195.0315, $C_6H_{14}N_2{}^{81}Br$ requires [M–2CF_3CO_2H+H]$^+$ 195.0314].

(8) 1-(2-Bromoethyl)-4-methylpiperazine dihydrobromide

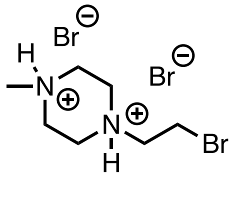

1-(2-Hydroxyethyl)-4-methylpiperazine (1.00 g, 6.93 mmol, 1.0 eq.) was added dropwise to a solution of triphenylphosphine dibromide (3.51 g, 8.32 mmol, 1.2 eq.) in CH_2Cl_2 (50 mL) at 0 °C. The reaction mixture was warmed to rt and stirred for 20 h. The reaction mixture was filtered, and the precipitate was washed with CH_2Cl_2 (30 mL) and MeOH (50 mL). The product was crystalised from hot MeOH to yield the title compound as a colourless crystalline solid (630 mg, 44%): R_f 0.15 (MeOH:CH_2Cl_2, 1:9); m.p. > 250 °C (from MeOH); ^{1}H NMR (600 MHz,

D_6-DMSO) δ_H 3.90–3.58 (m, 12H), 3.26 (s, 3H); ^{13}C NMR (151 MHz, D_6-DMSO) δ_C 54.7, 52.1, 51.4, 43.1, 24.6; $\overline{\nu}_{max}$ (thin film)/cm^{-1} 2975 (w), 1454 (m), 1021 (m), 957 (s); LRMS (ESI$^+$) *m/z* 207 ([M(^{79}Br)–2HBr+H]$^+$, 45%), 209 ([M(^{81}Br)–2HBr+H]$^+$, 46%); HRMS (ESI$^+$) *m/z* [Found: 207.0490, $C_7H_{16}N_2{}^{79}Br$ requires [M–2HBr+H]$^+$ 207.0491], [Found: 209.0469, $C_7H_{16}N_2{}^{81}Br$ requires [M–2HBr+H]$^+$ 209.0471].

(9) 2-(Isoindolin-2-yl)ethan-1-ol

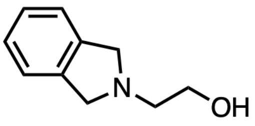

Isoindoline (952 µL, 8.39 mmol, 1.0 eq.) was added dropwise to a solution of 2-bromoethanol (595 µL, 8.39 mmol, 1.0 eq.), and NEt_3 (2.30 mL, 16.8 mmol, 2.0 eq.) in toluene (50 mL) at rt. The reaction solution was stirred and heated under reflux at 120 °C for 6 h. The reaction mixture was cooled to rt, filtered, and concentrated *in vacuo*. The residue was dissolved in EtOAc (30 mL) and washed with H_2O (50 mL). The organic component was dried (Na_2SO_4), filtered, and concentrated *in vacuo*. The product was purified using flash silica column chromatography (gradient elution on a Biotage® system with 2–20% MeOH/$CHCl_3$) to yield the title compound as a brown oil (600 mg, 44%): R_f 0.29 (MeOH:$CHCl_3$, 1:9); ^{1}H NMR (600 MHz, $CDCl_3$) δ_H 7.24–7.20 (m, 4H), 4.03 (s, 4H), 3.73 (t, *J* 5.4, 2H), 2.96 (t, *J* 5.4, 2H), 2.64 (br s, 1H); ^{13}C NMR (151 MHz, $CDCl_3$) δ_C 139.7, 127.1, 122.4, 59.9, 59.1, 57.6; LRMS (ESI$^+$) *m/z* 164 ([M+H]$^+$, 89%), 349 ([2M+Na]$^+$, 100%). The spectroscopic data are in good agreement with the literature values.[27]

(10) 2-(2-Bromoethyl)isoindoline hydrobromide

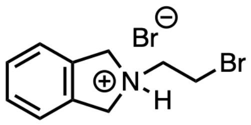

Compound **9** (600 mg, 3.68 mmol, 1.0 eq.) was reacted with aqueous HBr (48% *w/w*, 20 mL) according to the general bromination procedure and then crystalised from hot MeOH to yield the title compound as a beige crystalline solid (389 mg, 34%): R_f 0.85 (MeOH:CH_2Cl_2, 1:9); m.p. 182–185 °C (from MeOH); ^{1}H NMR (600 MHz, $CDCl_3$) δ_H 13.01 (br s, 1H), 7.43–7.37 (m, 2H), 7.35–7.29 (m, 2H), 5.13 (dd, *J* 13.9, 5.8 Hz, 2H), 4.37 (dd, *J* 13.9 5.8 Hz, 2H), 3.96 (t, *J* 7.1 Hz, 2H), 3.79–3.71 (m, 2H); ^{13}C NMR (151 MHz, $CDCl_3$) δ_C 132.3, 129.8, 123.2, 59.0, 56.9, 22.7; $\overline{\nu}_{max}$ (thin film)/cm^{-1} 3381 (br, w), 2464 (w), 767 (s); LRMS (ESI$^+$) *m/z* 226 ([M(^{79}Br)–HBr+H]$^+$, 57%), 228 ([M(^{81}Br)–HBr+H]$^+$, 59%); HRMS (ESI$^+$) *m/z* [Found: 226.0228, $C_{10}H_{13}N^{79}Br$ requires [M–HBr+H]$^+$ 226.0226].

(11) 2-(3,4-Dihydroisoquinolin-2(1*H*)-yl)ethan-1-ol

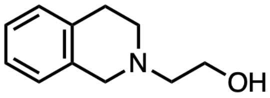

1,2,3,4-Tetrahydroisoquinoline (940 µL, 7.51 mmol, 1.0 eq.) was added dropwise to a solution of 2-bromoethanol (532 µL, 7.51 mmol, 1.0 eq.), and NEt_3 (2.10 mL, 15.0 mmol, 2.0 eq.) in toluene (50 mL) at rt. The reaction solution was stirred and heated under reflux at 120 °C for 20 h. The reaction mixture was cooled to rt, filtered, and concentrated *in vacuo*. The residue was dissolved in EtOAc (30 mL) and washed with H_2O (50 mL). The organic components were dried (Na_2SO_4), filtered, and concentrated *in vacuo*. The product was purified using flash silica column chromatography (gradient elution on a Biotage® system with 2–20% MeOH/$CHCl_3$) to yield the title compound as a yellow oil (978 mg, 74%): R_f 0.48 (MeOH:$CHCl_3$, 1:9); ^{1}H NMR (600 MHz, $CDCl_3$) δ_H 7.18–7.07 (m, 3H), 7.05–7.00 (m, 1H), 3.73–3.68 (m, 4H), 2.92 (t, *J* 5.9 Hz, 2H), 2.82 (t, *J* 5.9 Hz, 2H), 2.74–2.70 (m, 2H), 2.43 (br s, 1H); ^{13}C NMR (151 MHz, $CDCl_3$) δ_C 134.6, 134.4, 128.8, 126.7, 126.4, 125.9, 59.2, 58.2, 55.9, 50.8, 29.2; $\bar{\nu}_{max}$ (thin film)/cm^{-1} 3389 (br, w), 2922 (w), 741 (s); LRMS (ESI$^+$) *m/z* 178 ([M+H]$^+$, 39%), 222 ([M+2Na-H], 41%); HRMS (ESI$^+$) *m/z* [Found: 178.1228, $C_{11}H_{16}NO$ requires [M+H]$^+$ 178.1226]. The spectroscopic data are in good agreement with the literature values.[28]

(12) 2-(2-Bromoethyl)-1,2,3,4-tetrahydroisoquinoline hydrobromide

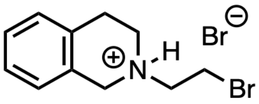

Compound **11** (978 mg, 5.52 mmol, 1.0 eq.) was reacted with aq. HBr (48% *w/w*, 20 mL) according to the general bromination procedure and crystallised from hot MeOH to yield the title compound as a colourless crystalline solid (929 mg, 52%): R_f 0.85 (MeOH/CH_2Cl_2, 1:9); m.p. 229–232 °C (from MeOH); ^{1}H NMR (600 MHz, D_6-DMSO) δ_H 10.17 (br s, 1H), 7.34–7.23 (m, 3H), 7.22–7.16 (m, 1H), 4.61 (d, *J* 15.5 Hz, 1H), 4.47–4.35 (m, 1H), 3.96–3.64 (m, 5H), 3.42* (m, 1H), 3.25–3.00 (m, 2H); ^{13}C NMR (151 MHz, D_6-DMSO) δ_C 131.1, 128.6, 128.0, 127.9, 126.7, 126.5, 55.5, 52.0, 49.1, 24.7, 24.2; $\bar{\nu}_{max}$ (thin film)/cm^{-1} 3658 (w), 2980 (s), 753 (w); LRMS (ESI$^+$) *m/z* 240 ([M(^{79}Br)–HBr+H]$^+$, 64%), 242 ([M(^{81}Br)–HBr+H]$^+$, 66%); HRMS (ESI$^+$) *m/z* [Found: 240.0383, $C_{11}H_{15}N^{79}Br$ requires [M–HBr+H]$^+$ 240.0382], [Found: 242.0362, $C_{11}H_{15}N^{81}Br$ requires [M–HBr+H]$^+$ 242.0362].

*Peak obscured by NMR solvent peak and was assigned by HSQC.

(13) 1-(2-Bromoethyl)pyridinium bromide

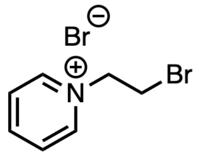

1,2-Dibromoethane (2.15 mL, 25.0 mmol, 1.0 eq.) was added dropwise to a solution of anhydrous pyridine (2.00 mL, 25.0 mmol, 1.0 eq.) in acetone (15 mL) at rt. The reaction solution was stirred and heated under reflux at 60 °C for 16 h. The resulting suspension was cooled to rt and the solvent was evaporated *in vacuo*. The precipitated crystals were washed with cold acetone (10 mL) and dried in a desiccator under high vacuum to yield the title compound as a colourless solid (4.89 g, 74%): R_f 0.07 (MeOH/CH_2Cl_2, 1:9); m.p. 114–117 °C (from acetone) [lit.[29] 95–102 °C (from ethanol/acetone)]; ^{1}H NMR (600 MHz, CD_3OD) δ_H 9.13–9.08 (m, 2H), 8.74–8.67 (m, 1H), 8.25–8.16 (m, 2H), 5.12 (t, *J* 5.8 Hz, 2H), 4.07 (t, *J* 5.8 Hz, 2H); ^{13}C NMR (151 MHz, CD_3OD) δ_C 147.9, 146.4, 129.5, 63.3, 31.1; $\overline{\nu}_{max}$ (thin film)/cm^{-1} 2981 (w), 1633 (m), 1486 (m), 773 (s), 692 (s), 676 (s); LRMS (ESI$^+$) *m/z* 186 ([M(^{79}Br)–Br]$^+$, 34%), 188 ([M(^{81}Br)–Br]$^+$, 53%). The spectroscopic data are in good agreement with the literature values.[13]

(14) 2-(2-Bromoethyl)pyridine

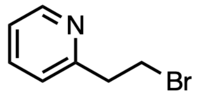

Tetrabromomethane (3.55 g, 10.7 mmol, 1.2 eq.) and triphenylphosphine (2.81 g, 10.7 mmol, 1.2 eq.) were added to a solution of 2-(2-hydroxyethyl)pyridine (1.00 mL, 8.88 mmol, 1.0 eq.) in THF (20 mL) at 0 °C. The reaction mixture was warmed to rt and stirred for 20 h; before being diluted with Et_2O (50 mL). The resulting suspension was filtered, and the filtrate was concentrated *in vacuo*. The product was purified using flash silica column chromatography (gradient elution on a Biotage® system with 7–60% EtOAc/petroleum ether 40–60 °C) to yield the title compound as a yellow oil (740 mg, 45%): R_f 0.35 (EtOAc/petroleum ether 40–60 °C, 3:7); ^{1}H NMR (600 MHz, $CDCl_3$) δ_H 8.59–8.53 (m, 1H), 7.66–7.59 (m, 1H), 7.22–7.13 (m, 2H), 3.77 (t, *J* 7.1 Hz, 2H), 3.33 (t, *J* 7.1 Hz, 2H); ^{13}C NMR (151 MHz, $CDCl_3$) δ_C 158.6, 149.8, 136.6, 123.7, 122.0, 41.3, 31.8; $\overline{\nu}_{max}$ (thin film)/cm^{-1} 3010 (w), 2970 (w), 1592 (m), 1474 (m), 1437 (m), 752 (s); LRMS (ESI$^+$) *m/z* 186 ([M(^{79}Br)+H]$^+$, 100%), 188 (M(^{81}Br)+H]$^+$, 100%); HRMS (ESI$^+$) *m/z* [Found: 185.9913, $C_7H_9N^{79}Br$ requires [M+H]$^+$ 185.9913], [Found: 187.9891, $C_7H_9N^{81}Br$ requires [M+H]$^+$ 187.9892]. The spectroscopic data are in good agreement with the literature values.[30]

(15) 1-(2-Bromoethyl)-1*H*-pyrazole

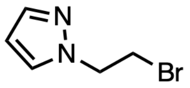

1,2-Dibromoethane (50.7 mL, 588 mmol, 20.0 eq.) was added dropwise to a solution of pyrazole (2.00 g, 29.4 mmol, 1.0 eq.) and tetrabutylammonium bromide (948 mg, 2.94 mmol, 0.1 eq.) in aq. NaOH (40% *w/v*, 15 mL) at rt. The reaction mixture was stirred at rt for 24 h. H_2O (30 mL) was added, and the organic phase was separated and washed with H_2O (50 mL). The organic phase was dried (Na_2SO_4), filtered, and concentrated *in vacuo*. The product was purified using flash silica column chromatography (gradient elution on a Biotage® system with 5-40% EtOAc/petroleum ether 40–60 °C) to yield the title compound as a pale yellow oil (3.17 g, 62%): R_f 0.36 (EtOAc/petroleum ether 40–60 °C, 1:4); ^{1}H NMR (400 MHz, $CDCl_3$) δ_H 7.55 (d, *J* 1.9 Hz, 1H), 7.46 (d, *J* 2.2 Hz, 1H), 6.26 (dd, *J* 2.2 1.9 Hz, 1H), 4.50 (t, *J* 6.4 Hz, 2H), 3.72 (t, *J* 6.4 Hz, 2H); LRMS (ESI$^+$) *m/z* 175 ([M(^{79}Br)+H]$^+$, 98%), 177 ([M(^{81}Br)+H]$^+$, 100%). The spectroscopic data are in good agreement with the literature values.[31,32]

(16) 1-(*tert*-Butyl) 2-methyl 2-(3-chloropropyl)pyrrolidine-1,2-dicarboxylate

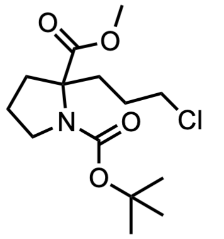

A solution of 1-(*tert*-butyl) 2-methyl pyrrolidine-1,2-dicarboxylate (10.0 g, 43.6 mmol, 1.0 eq.) in anhydrous THF (30 mL) was added dropwise to a solution of LDA (1 M in THF, 65.4 mL, 65.4 mmol, 1.5 eq.) at −78 °C under an argon atmosphere. The reaction solution was stirred at −78 °C for 2 h. 1-Chloro-3-iodopropane (8.89 mL, 82.8 mmol, 1.9 eq.) was added dropwise; and the reaction mixture was warmed to rt, and stirred for 2 h. The reaction mixture was cooled to 0 °C and sat. aq. NH_4Cl (100 mL) was added dropwise. The aqueous phase was extracted with EtOAc (3 × 100 mL); and the organic components were combined, washed with brine (100 mL), dried (Na_2SO_4), filtered, and concentrated *in vacuo*. The product was purified using flash silica column chromatography (gradient elution with 7–60% EtOAc/petroleum ether 40–60 °C) to yield the title compound as a yellow oil (9.02 g, 68%): R_f 0.32 (EtOAc/petroleum ether 40–60 °C, 1:4); ^{1}H NMR (600 MHz, D_6-DMSO) δ_H 3.75–3.44 (m, 7H), 2.28–2.16 (m, 1H), 2.12–1.70 (m, 6H), 1.61–1.46 (m, 1H), 1.32 (s, 9H); $\bar{\nu}_{max}$ (thin film)/cm^{-1} 2976 (w), 1742 (s), 1698 (s), 1391 (s), 1164 (s); LRMS (ESI$^+$) *m/z* 328 ([M(^{35}Cl)+Na]$^+$, 58%), 330 ([M(^{37}Cl)+Na]$^+$,

33%), 633 ([2M(^{35}Cl)+Na]$^+$, 100%), 635 ([2M(^{37}Cl)+Na]$^+$, 92%). The spectroscopic data are in good agreement with the literature values.[33]

(17) Methyl tetrahydro-1*H*-pyrrolizine-7a(5*H*)-carboxylate

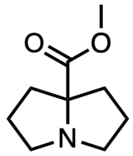

TMSCl (18.8 mL, 148 mmol, 5.0 eq.) was added dropwise to a solution of **16** (9.02 g, 29.6 mmol, 1.0 eq.) in MeOH (100 mL) at 0 °C. The reaction solution was warmed to rt and stirred for 16 h. The reaction solution was basified to pH 8.0 with sat. aq. $NaHCO_3$ and the reaction solution was concentrated *in vacuo*. The aqueous phase was extracted with CH_2Cl_2 (3 × 100 mL); and the organic components were combined, dried (Na_2SO_4), filtered, and concentrated *in vacuo*. The product was purified using flash silica column chromatography (gradient elution with 5–15% MeOH/CH_2Cl_2) to yield the title compound as a brown oil (3.95 g, 79%): R_f 0.23 (MeOH/CH_2Cl_2, 1:11.5); ^{1}H NMR (400 MHz, $CDCl_3$) δ_H 3.86 (s, 3H), 3.70–3.40 (m, 4H), 2.55–1.60 (m, 8H); $\bar{\nu}_{max}$ (thin film)/cm^{-1} 2953 (C-H, w), 1744 (C=O, s), 1222 (m), 732 (s); LRMS (ESI$^+$) *m/z* 170 ([M+H$^+$], 100%). The spectroscopic data are in good agreement with the literature values.[33]

(18) (Tetrahydro-1*H*-pyrrolizin-7a(5*H*)-yl)methanol

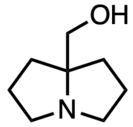

$LiAlH_4$ (4.38 g, 116 mmol, 5.0 eq.) was added portionwise to a solution of **17** (3.90 g, 23.1 mmol, 1.0 eq.) in anhydrous THF (25 mL) at −10 °C. The reaction mixture was warmed to rt and stirred for 1 h. The reaction mixture was cooled to 0 °C and quenched sequentially with H_2O (4.38 mL), aqueous NaOH (15% *w/w*, 4.38 mL), and H_2O (13.1 mL). The mixture was warmed to rt and stirred for 15 min. Anhydrous Na_2SO_4 (5 g) was added, and the mixture was stirred at rt for 15 min. The mixture was filtered, and the filter cake was washed with THF (30 mL). The filtrate was concentrated *in vacuo* to yield the title compound as a yellow oil which was deemed pure enough for use in the next step without further purification (2.86 g, 88%): R_f 0.52 (MeOH:CH_2Cl_2, 1:9); ^{1}H NMR (400 MHz, CD_3OD) δ_H 3.43 (s, 2H), 3.20–3.09 (m, 2H), 2.88–2.78 (m, 2H), 2.10–1.55 (m, 8H); LRMS (ESI$^+$) *m/z* 142 ([M+H]$^+$, 100%); HRMS (ESI$^+$) *m/z* [Found 142.1227, $C_8H_{16}NO$ requires [M+H]$^+$ 142.1226]. The spectroscopic data are in good agreement with the literature values.[33]

(19) 7a-(Chloromethyl)hexahydro-1*H*-pyrrolizine hydrochloride

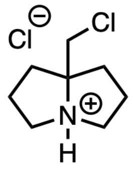

Thionyl chloride (15.5 mL, 212 mmol, 3.0 eq.) was added dropwise to a solution of **18** (10.0 g, 70.8 mmol, 1.0 eq.) in toluene (200 mL) at 0 °C. The reaction mixture was warmed to 80 °C and stirred for 3 h. The reaction mixture was concentrated *in vacuo*. The residue was redissolved in CH_2Cl_2 and triturated with Et_2O to yield the title compound as a beige solid (12.7 g, 91%): R_f 0.30 (MeOH/CH_2Cl_2, 1:9); m.p. 199–201 °C (from CH_2Cl_2/Et_2O); ^{1}H NMR (600 MHz, $CDCl_3$) δ_H 12.47 (br s, 1H), 3.94 (s, 2H), 3.84–3.72 (m, 2H), 3.06–2.95 (m, 2H), 2.34–2.24 (m, 2H), 2.18–2.03 (m, 4H), 2.02–1.90 (m, 2H); ^{13}C NMR (151 MHz, $CDCl_3$) δ_C 80.8, 55.8, 47.6, 35.6, 24.1; $\overline{\nu}_{max}$ (thin film)/cm^{-1} 3393 (m, br), 2953 (w), 742 (s); LRMS (ESI$^+$) *m/z* 160 ([M(^{35}Cl)+H]$^+$, 88%); HRMS (ESI$^+$) *m/z* [Found: 160.0889, $C_8H_{15}N^{35}Cl$ requires [M+H]$^+$ 160.0888].

(20) (2*R*,3*S*,4*S*,5*R*,6*R*)-2-(Acetoxymethyl)-6-(2-bromoethoxy)tetrahydro-2*H*-pyran-3,4,5-triyl triacetate

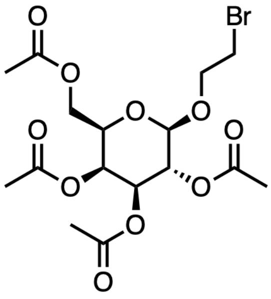

Boron trifluoride diethyl etherate (3.63 mL, 7.69 mmol, 1.2 eq.), followed by 2-bromoethanol (0.55 mL, 7.69 mmol, 1.2 eq.), were added dropwise to a solution of β-D-galactose pentaacetate (2.50 g, 6.40 mmol, 1.0 eq.) in anhydrous CH_2Cl_2 (20 mL) at 0 °C. The reaction solution was warmed to rt and stirred for 3 h. K_2CO_3 (1.33 g, 9.60 mmol, 1.5 eq.) was added and the reaction mixture was stirred at rt for 1 h. The reaction mixture was concentrated *in vacuo* and the residue was resuspended in brine (25 mL). The aqueous phase was extracted with $CHCl_3$ (3 × 50 mL), and the organic components were combined, dried (Na_2SO_4), filtered, and concentrated *in vacuo*. The product was purified by flash silica column chromatography (gradient elution with 20–50% EtOAc/petroleum ether 40–60 °C) to yield the title compound as a pale yellow oil which crystalised on standing to give a colourless amorphous solid (1.68 g, 58%): R_f 0.21 (EtOAc/petroleum ether 40–60 °C, 3:7); $[\alpha]_D^{25}$ = +19.9 (*c*=1.0 in $CHCl_3$); ^{1}H NMR

(600 MHz, $CDCl_3$) δ_H 5.39 (dd, *J* 5.7, 5.0 Hz, 1H), 5.22 (dd, *J* 10.5, 5.7 Hz, 1H), 5.02 (dd, *J* 10.5, 5.7 Hz, 1H), 4.53 (d, *J* 5.7 Hz, 1H), 4.20–4.05 (m, 3H), 3.91 (td, *J* 6.7, 5.0 Hz, 1H), 3.81 (ddd, *J* 11.3, 7.2, 6.6 Hz, 1H), 3.52–3.42 (m, 2H), 2.15 (s, 3H), 2.08 (s, 3H), 2.05 (s, 3H), 1.98 (s, 3H); ^{13}C NMR (151 MHz, $CDCl_3$) δ_C 170.5, 170.34, 170.26, 169.7, 101.7, 71.0, 70.9, 69.9, 68.7, 67.1, 61.4, 30.1, 21.0, 20.80, 20.78, 20.7; LRMS (ESI^+) *m/z* 477 ($[M(^{79}Br)+Na]^+$, 95%), 479 ($[M(^{81}Br)+Na]^+$, 96%). The spectroscopic data are in good agreement with the literature values.[34,35]

(21) (2*R*,3*R*,4*S*,5*R*,6*R*)-2-(2-Bromoethoxy)-6-(hydroxymethyl)tetrahydro-2*H*-pyran-3,4,5-triol

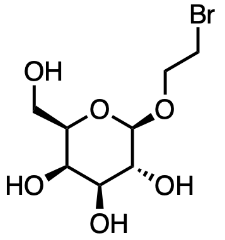

A solution of NaOMe in MeOH (25% *w/w*, 39 µL, 0.18 mmol, 0.05 eq.) was added dropwise to a stirring solution of **20** (1.67 g, 3.67 mmol, 1.0 eq.) in MeOH (20 mL) at rt. The reaction solution was stirred at rt for 4 h, before being filtered through Dowex® 50WX8 ion exchange resin (H^+ form, 50–100 mesh, 2 × 5 cm plug). The resin was washed with MeOH (3 × 10 mL), and the combined filtrates were concentrated *in vacuo*. The product was purified by flash silica column chromatography (isocratic elution with 30% EtOH/CH_2Cl_2) to yield the title compound as a pale yellow oil which crystalised on standing to form an amorphous off-white solid (766 mg, 57%): R_f 0.46 (EtOH/CH_2Cl_2, 3:7); $[\alpha]_D^{25}$ = –2.2 (*c*=1.0 in MeOH); ^1H NMR (600 MHz, CD_3OD) δ_H 4.32 (d, *J* 7.6 Hz, 1H), 4.18–4.10 (m, 1H), 3.99–3.91 (m, 1H), 3.88–3.84 (m, 1H), 3.82–3.71 (m, 2H), 3.68–3.52 (m, 4H), 3.50 (dd, *J* 9.7 3.4 Hz, 1H); ^{13}C NMR (151 MHz, CD_3OD) δ_C 105.2, 76.8, 74.9, 72.4, 70.9, 70.3, 62.5, 31.0; $\overline{\nu}_{max}$ (thin film)/cm^{-1} 3366 (br, s), 2892 (w), 1071 (s); LRMS (ESI^+) *m/z* 309 ($[M(^{79}Br)+Na]^+$, 78%), 311 ($[M(^{81}Br)+Na]^+$, 78%); HRMS (ESI^+) *m/z* [Found: 308.9943, $C_8H_{15}O_6{}^{79}BrNa$ requires $[M+Na]^+$ 308.9944], [Found: 310.9922, $C_8H_{15}O_6{}^{81}BrNa$ requires $[M+Na]^+$ 310.9924]. The spectroscopic data are in good agreement with the literature values.[36]

(22) 5-Nitro-1,3-dihydro-2*H*-benzo[*d*]imidazol-2-one

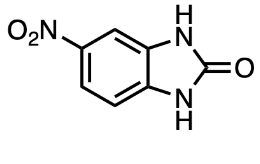

1,1'-Carbonyldiimidazole (10.6 g, 65.2 mmol, 2.0 eq.) was added portion wise to a solution of 4-nitro-*o*-phenyldiamine (5.00 g, 32.6 mmol, 1.0 eq.) in anhydrous DMF (50 mL) at rt. The reaction mixture was stirred at rt for 20 h, before being concentrated *in vacuo* to ~20 mL. The reaction mixture was diluted with H_2O (200 mL) and filtered. The precipitate was washed with H_2O (3 × 100 mL) and dried overnight under high vacuum to yield the title compound as a yellow solid (5.82 g, >99%): R_f 0.59 (1% NEt_3; MeOH:CH_2Cl_2, 1:9); m.p. >300 °C (from H_2O/MeCN) [lit.[37] 308–309 °C]; ^1H NMR (400 MHz, D_6-DMSO) δ_H 11.41 (br s, 1H), 11.18 (br s, 1H), 7.94 (dd, *J* 2.3 8.7 Hz, 1H), 7.71 (d, *J* 2.3 Hz, 1H), 7.10 (d, *J* 8.7 Hz, 1H); LRMS (ESI$^-$) *m/z* 178 ([M–H]$^-$, 100%). The spectroscopic data are in good agreement with the literature values.[37]

(23) 1,3-Dimethyl-5-nitro-1,3-dihydro-2*H*-benzo[*d*]imidazol-2-one

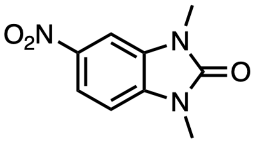

K_2CO_3 (6.17 g, 44.7 mmol, 4.0 eq.) and MeI (2.78 mL, 44.7 mmol, 4.0 eq.) were added to a solution of **22** (2.00 g, 11.2 mmol, 1.0 eq.) in anhydrous DMF (20 mL) at 0 °C. The reaction mixture was warmed to rt and stirred for 16 h. The reaction mixture was diluted with H_2O (100 mL) and filtered. The precipitate was washed with H_2O (3 × 50 mL) and dried overnight under high vacuum to yield the title compound as a yellow solid (2.25 g, 97%): R_f 0.76 (1% NEt_3; MeOH/CH_2Cl_2, 1:19); m.p. 200–202 °C (from H_2O) [lit.[38] 202–204 °C, lit.[39] 220 °C]; ^1H NMR (400 MHz, D_6-DMSO) δ_H 8.10–8.05 (m, 2H), 7.40–7.32 (m, 1H), 3.43 (s, 3H), 3.41 (s, 3H); $\overline{\nu}_{max}$ (thin film)/cm^{-1} 1708 (s), 1513 (s); LRMS (ESI$^+$) *m/z* 208 ([M+H$^+$], 100%). The spectroscopic data are in good agreement with the literature values.[38,39]

(24) 5-Amino-1,3-dimethyl-1,3-dihydro-2*H*-benzo[*d*]imidazol-2-one

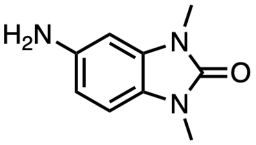

Conc. aqueous HCl (37% *w/w*, 200 μL) was added to a suspension of **23** (3.20 g, 15.5 mmol, 1.0 eq.) in EtOH (200 mL) under an argon atmosphere at rt. 10% Pd/C (1.65 g, 1.55 mmol, 0.1 eq.) was added and the flask was purged with hydrogen gas *via* 3 successive evacuate/fill cycles. The reaction mixture was stirred vigorously under a hydrogen atmosphere at rt for 16 h. The reaction mixture was filtered through Celite® and the filtrate was concentrated *in vacuo* to yield the title compound as a pink solid (2.67 g, 97%): R_f 0.35 (1% NEt_3; MeOH/CH_2Cl_2, 1:19);

m.p. 140–142 °C (from CH_2Cl_2) [lit.[39] 160 °C, lit.[38] 135–136 °C (from benzene)]; ^{1}H NMR (400 MHz, D_6-DMSO) δ_H 6.88 (d, *J* 8.2 Hz, 1H), 6.51 (d, *J* 2.1 Hz, 1H), 6.46 (dd, *J* 2.1 8.2 Hz, 1H), 6.28 (br s, 2H), 3.24 (s, 3H), 3.23 (s, 3H); LRMS (ESI$^+$) *m/z* 178 ([M+H]$^+$, 100%). The spectroscopic data are in good agreement with the literature values.[39]

(25) 5-Amino-6-bromo-1,3-dimethyl-1,3-dihydro-2*H*-benzo[*d*]imidazol-2-one

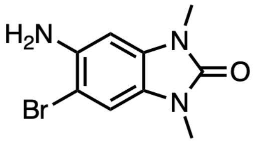

Bromine (290 µL, 5.65 mmol, 1.0 eq.) was added dropwise to a solution of **24** (1.00 g, 5.65 mmol, 1.0 eq.) in anhydrous $CHCl_3$ (20 mL) and AcOH (20 mL) at −20 °C. The reaction mixture was stirred at −20 °C for 20 min and quenched with aq. $Na_2S_2O_3$ (10% *w/v*, 25 mL). The mixture was stirred for 30 min and diluted with H_2O (25 mL). The aqueous phase was extracted with $CHCl_3$ (3 × 50 mL) and the organic components were combined, washed with sat. aq. $NaHCO_3$ (50 mL), brine (50 mL), dried (Na_2SO_4), filtered, and concentrated *in vacuo*. The product was purified by flash silica column chromatography (gradient elution with 20–50% EtOAc/CH_2Cl_2) to yield the title compound as an off-white solid (1.06 g, 73%): R_f 0.21 (EtOAc:CH_2Cl_2, 1:4), m.p. 183–185 °C (dec., from EtOAc) [lit.[40] 209–210 °C]; ^{1}H NMR (400 MHz, D_6-DMSO) δ_H 7.18 (s, 1H), 6.58 (s, 1H), 4.96 (br s, 2H), 3.22 (s, 3H), 3.21 (s, 3H); LRMS (ESI$^+$) *m/z* 256 ([M(^{79}Br)+H]$^+$, 100%), 258 ([M(^{81}Br)+H]$^+$, 99%). The spectroscopic data are in good agreement with the literature values.[1,41]

(26) 3-Propoxyphenol

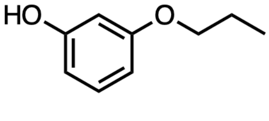

K_2CO_3 (13.8 g, 100 mmol, 3.0 eq.) was added to a solution of resorcinol (11.0 g, 100 mmol, 3.0 eq.) in DMF (30 mL) at rt. 1-Bromopropane (3.00 mL, 33.3 mmol, 1.0 eq.) was added dropwise over 5 min at rt. The reaction mixture was stirred vigorously at 50 °C for 16 h. The reaction mixture was quenched with aq. HCl (1 M, 150 mL) and the aqueous phase was extracted with EtOAc (4 × 100 mL). The organic components were combined, washed with aq. LiCl (0.5 M, 100 mL), brine (100 mL), dried ($MgSO_4$), filtered, and concentrated *in vacuo*. The product was purified by flash silica column chromatography (gradient elution on a Biotage® system with 7–60% EtOAc/petroleum ether 40–60 °C) to yield the title compound as a pale-yellow oil (3.50 g, 69%): R_f 0.61 (EtOAc/petroleum ether 40–60 °C, 3:7); ^{1}H NMR (400 MHz, $CDCl_3$) δ_H 7.16–7.08 (dd, *J* 8.4 8.4 Hz, 1H), 6.49 (ddd, *J* 1.0 2.3 8.4 Hz, 1H), 6.45–6.38 (m,

2H), 5.23 (s, 1H), 3.89 (t, *J* 6.6 Hz, 2H), 1.88–1.72 (m, 2H), 1.02 (t, *J* 7.4 Hz, 3H); LRMS (ESI$^+$) *m/z* 305 ([2M+H]$^+$, 100%). The spectroscopic data are in good agreement with the literature values.[41,42]

(27) 5-Amino-1,3-dimethyl-6-(3-propoxyphenoxy)-1,3-dihydro-2*H*-benzo[*d*]imidazol-2-one

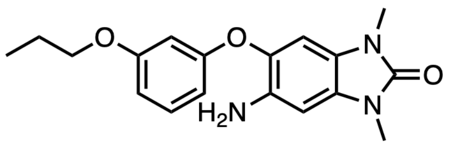

Sodium hydroxide (920 mg, 23.0 mmol, 1.0 eq.) was added to a solution of **26** (3.50 g, 23.0 mmol, 1.0 eq.) in MeOH (10 mL). The reaction mixture was stirred at rt for 1 h. The mixture was concentrated *in vacuo*, and the residue was dissolved in toluene and concentrated *in vacuo* to yield sodium 3-propoxyphenolate as a white solid (4.00 g, 100%). The product was dried by lyophilisation and used directly in the next step.

CuI and Cs_2CO_3 were dried in an Abderhalden's drying pistol under high vacuum at 160 °C for 24 h. A Schlenk tube was charged with **25** (300 mg, 1.17 mmol, 1.0 eq.), sodium 3-propoxyphenolate (306 mg, 1.76 mmol, 1.5 eq.), Cs_2CO_3 (1.14 g, 3.51 mmol, 3.0 eq.), CuI (22.3 mg, 0.12 mmol, 0.1 eq.), and 8-hydroxyquinoline (34.0 mg, 0.23 mmol, 0.2 eq.). The tube was sealed and purged with argon *via* 3 successive evacuate/fill cycles. Anhydrous diglyme (20 mL) was degassed by 3 successive freeze-pump-thaw cycles under argon before being added to the reaction mixture. The reaction mixture was stirred vigorously at 130 °C for 72 h. The reaction mixture was cooled to rt and filtered through a silica plug. The silica plug was washed with 10% MeOH/CH_2Cl_2 (100 mL) and the filtrate was concentrated *in vacuo* at 60 °C. The product was purified by flash silica column chromatography (gradient elution on a Biotage® system with 0–6% MeOH/$CHCl_3$) to yield the title compound as a brown solid (235 mg, 62%): R_f 0.26 (MeOH/$CHCl_3$, 3:97); m.p. 134–136 °C (from $CHCl_3$) [lit.[12] 137–139 °C (from CH_2Cl_2)]; ^{1}H NMR (400 MHz, $CDCl_3$) δ_H 7.17 (dd, *J* 8.6 8.6 Hz, 1H), 6.63 (s, 1H), 6.61–6.56 (m, 1H), 6.54–6.43 (m, 3H), 3.88 (t, *J* 6.6 Hz, 2H), 3.37 (s, 3H), 3.31 (s, 3H), 1.84–1.70 (m, 2H), 1.01 (t, *J* 7.5 Hz, 3H); LRMS (ESI$^+$) *m/z* 328 ([M+H]$^+$, 65%), 655 ([2M+H]$^+$, 100%). The spectroscopic data are in good agreement with the literature values.[1,12]

(28) 3-(*N*-(1,3-Dimethyl-2-oxo-6-(3-propoxyphenoxy)-2,3-dihydro-1*H*-benzo[*d*]imidazol-5-yl)sulfamoyl)benzoic acid

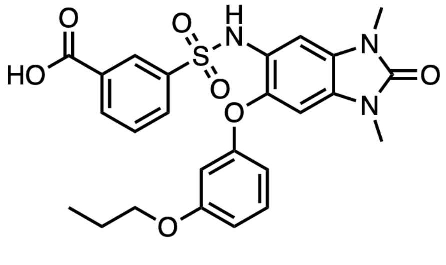

3-(Chlorosulfonyl)benzoic acid (324 mg, 1.47 mmol, 1.2 eq.) and anhydrous pyridine (198 μL, 2.44 mmol, 2.0 eq.) were added to a solution of **27** (400 mg, 1.22 mmol, 1.0 eq.) in anhydrous CH_2Cl_2 (20 mL) at rt. The rection solution was stirred at rt for 24 h and quenched by addition of aq. HCl (1 M, 20 mL). The aqueous phase was extracted with 25% MeOH in CH_2Cl_2 (3 × 50 mL); and the organic components were combined, dried (Na_2SO_4), filtered, and concentrated *in vacuo*. The product was purified by flash silica column chromatography (gradient elution with 0–10% MeOH/CH_2Cl_2) to yield the title compound as a brown solid (442 mg, 71%): R_f 0.14 (MeOH/CH_2Cl_2, 3:47); m.p. 120–122 °C (dec., from CH_2Cl_2) [lit.[12] 116–118 °C (from CH_2Cl_2)]; ^{1}H NMR (400 MHz, CD_3OD) δ_H 8.29 (t, *J* 1.8 Hz, 1H), 8.13–8.05 (m,1H), 7.80–7.72 (m, 1H), 7.42–7.32 (m, 2H), 7.02 (t, *J* 8.2 Hz, 1H), 6.59–6.50 (m, 2H), 6.11–6.04 (m, 1H), 6.01 (t, *J* 2.4 Hz, 1H), 3.80 (t, *J* 6.5 Hz, 2H), 3.43 (s, 3H), 3.23 (3H, s, 3H), 1.82–1.69 (m, 2H), 1.02 (t, *J* 7.4 Hz, 3H); LRMS (ESI$^+$) *m/z* 512 ([M+H]$^+$, 56%), 1045 ([2M+Na]$^+$, 100%). The spectroscopic data are in good agreement with the literature values.[12]

(29) *tert*-Butyl (1-(3-(*N*-(1,3-dimethyl-2-oxo-6-(3-propoxyphenoxy)-2,3-dihydro-1*H*-benzo[*d*]imidazol-5-yl)sulfamoyl)phenyl)-1-oxo-5,8-dioxa-2-azadecan-10-yl)carbamate

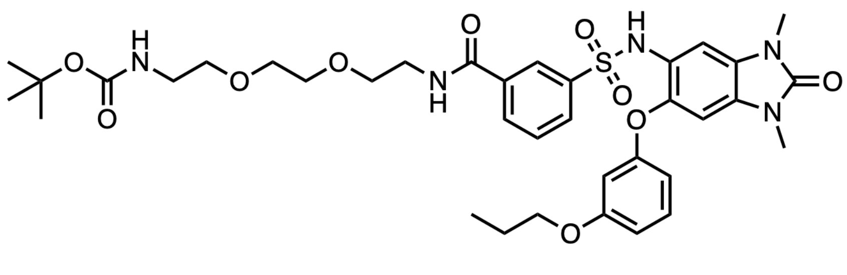

Carboxylic acid **28** (100 mg, 0.20 mmol, 1.0 eq.) and *tert*-butyl (2-(2-(2-aminoethoxy)ethoxy)ethyl)carbamate (48.5 mg, 0.20 mmol, 1.0 eq.) were reacted according to general amide coupling procedure 1. The product was purified by flash silica column chromatography (gradient elution with 1–5% MeOH/CH_2Cl_2) to yield the title compound as a brown oil (98.3 mg, 66%): R_f 0.28 (MeOH/CH_2Cl_2, 1:19); ^{1}H NMR (400 MHz, CD_3OD) δ_H 8.17 (t, *J* 1.8 Hz, 1H), 7.96–7.90 (m, 1H), 7.80–7.74 (m, 1H), 7.43–7.34 (m, 2H), 7.01 (t, *J* 8.5 Hz,

1H), 6.60–6.52 (m, 2H), 6.11–6.01 (m, 2H), 3.81 (t, *J* 6.5 Hz, 2H), 3.70–3.46 (m, 10H), 3.44 (s, 3H), 3.24 (s, 3H), 3.20 (t, *J* 5.7 Hz, 2H), 1.83–1.69 (m, 2H), 1.41 (s, 9H), 1.02 (t, *J* 7.4 Hz, 3H); LRMS (ESI$^+$) *m/z* 742 ([M+H]$^+$, 39%), 764 ([M+Na]$^+$, 100%). The spectroscopic data are in good agreement with previous work in the group.

(30) *tert*-Butyl (1-(3-(*N*-(1,3-dimethyl-2-oxo-6-(3-propoxyphenoxy)-2,3-dihydro-1*H*-benzo[*d*]imidazol-5-yl)sulfamoyl)phenyl)-1-oxo-5,8,11-trioxa-2-azatridecan-13-yl)carbamate

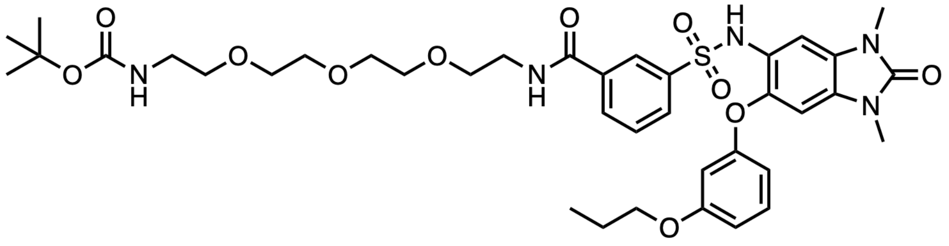

Carboxylic acid **28** (100 mg, 0.20 mmol, 1.0 eq.) and *tert*-butyl (2-(2-(2-(2-aminoethoxy)ethoxy)ethoxy)ethyl)carbamate (58.5 mg, 0.20 mmol, 1.0 eq.) were reacted according to general amide coupling procedure 1. The product was purified by flash silica column chromatography (gradient elution with 1–5% MeOH/CH_2Cl_2) to yield the title compound as a brown oil (126 mg, 80%): R_f 0.25 (MeOH/CH_2Cl_2, 1:19); ^{1}H NMR (400 MHz, CD_3OD) δ_H 8.17 (t, *J* 1.8 Hz, 1H), 7.98–7.90 (m, 1H), 7.81–7.75 (m, 1H), 7.44–7.34 (m, 2H), 7.02 (t, *J* 8.5 Hz, 1H), 6.60–6.52 (m, 2H), 6.08–6.02 (m, 2H), 3.81 (t, *J* 6.5 Hz, 2H), 3.66–3.50 (m, 12H), 3.48–3.42 (m, 5H), 3.24 (s, 3H), 3.18 (t, *J* 5.6 Hz, 2H), 1.82–1.70 (m, 2H), 1.41 (s, 9H), 1.03 (t, *J* 7.4 Hz, 3H); LRMS (ESI$^+$) *m/z* 786 ([M+H]$^+$, 37%), 808 ([M+Na]$^+$, 100%). The spectroscopic data are in good agreement with previous work in the group.

(31) *tert*-Butyl (1-(3-(*N*-(1,3-dimethyl-2-oxo-6-(3-propoxyphenoxy)-2,3-dihydro-1*H*-benzo[*d*]imidazol-5-yl)sulfamoyl)phenyl)-1-oxo-5,8,11,14-tetraoxa-2-azahexadecan-16-yl)carbamate

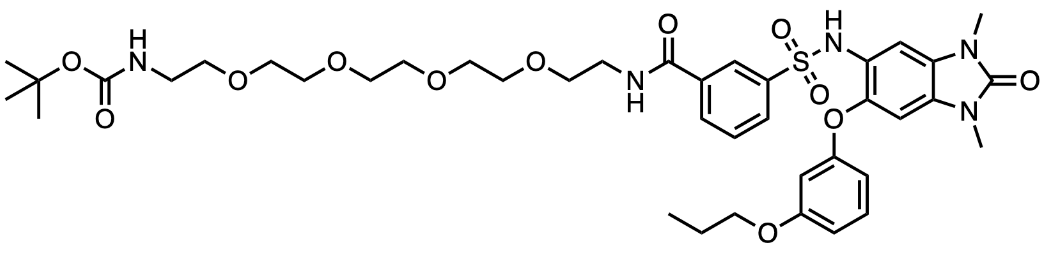

Carboxylic acid **28** (100 mg, 0.20 mmol, 1.0 eq.) and *tert*-butyl (14-amino-3,6,9,12-tetraoxatetradecyl)carbamate (67.3 mg, 0.20 mmol, 1.0 eq.) were reacted according to general amide coupling procedure 1. The product was purified by flash silica column chromatography (gradient elution with 2–6% MeOH/CH_2Cl_2) to yield the title compound as a

brown oil (146 mg, 88%): R_f 0.17 (MeOH/CH_2Cl_2, 1:19); ^{1}H NMR (600 MHz, CD_3OD) δ_H 8.17 (t, *J* 1.8 Hz, 1H), 7.96–7.92 (m, 1H), 7.80–7.76 (m, 1H), 7.40 (t, *J* 7.9 Hz, 1H), 7.36 (s, 1H), 7.02 (t, *J* 8.6 Hz, 1H), 6.59–6.53 (m, 2H), 6.08–6.03 (m, 2H), 3.81 (t, *J* 6.4 Hz, 2H), 3.66–3.50 (m, 16H), 3.46 (t, *J* 5.6 Hz, 2H), 3.43 (s, 3H), 3.24 (s, 3H), 3.19 (t, *J* 5.6 Hz, 2H), 1.80–1.71 (m, 2H), 1.42 (s, 9H), 1.02 (t, *J* 7.4 Hz, 3H); ^{13}C NMR (151 MHz, CD_3OD) δ_C 167.9, 161.7, 159.6, 158.4, 156.5, 146.7, 142.0, 136.2, 132.3 , 131.1, 131.1, 130.1, 129.9, 127.28, 127.25, 123.1, 110.6, 110.3, 108.2, 105.3, 100.8, 80.1, 71.59, 71.54, 71.52, 71.51, 71.3, 71.2, 71.0, 70.6, 70.4, 41.3, 41.1, 28.8, 27.6, 27.5, 23.6, 10.9; $\overline{\nu}_{max}$ (thin film)/cm^{-1} 2875 (w), 1698 (s), 1508 (s), 1460 (m), 1256 (m), 1173 (s), 736 (m); LRMS (ESI$^+$) *m/z* 830 ([M+H]$^+$, 79%), 852 ([M+Na]$^+$, 100%); HRMS (ESI$^+$) *m/z* [Found: 830.3673, $C_{40}H_{56}N_5O_{12}S$ requires [M+H]$^+$ 830.3641]; HPLC retention time: 9.6 min, 99.3% (250 nm), 96.6% (280 nm), >99.9% (310 nm).

(32) 3-(*N*-(1,3-Dimethyl-2-oxo-6-(3-propoxyphenoxy)-2,3-dihydro-1*H*-benzo[*d*]imidazol-5-yl)sulfamoyl)-*N*-(2-oxo-6,9-dioxa-3-azaundecan-11-yl)benzamide

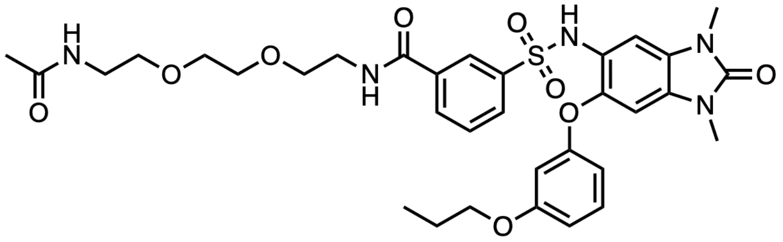

Compound **29** (58.3 mg, 78.6 μmol, 1.0 eq.) was deprotected according to the general Boc deprotection procedure to yield the crude amine which was directly acetylated according to the general acetylation procedure. The product was purified by semi-preparative reverse phase HPLC (gradient method 2, 20 mL min^{-1}, Agilent Prep C18 column [5 μm, 21.2 mm × 50 mm]) and lyophilised to yield the title compound as a colourless oil (38.9 mg, 72%): R_f 0.16 (MeOH/CH_2Cl_2, 1:19); ^{1}H NMR (600 MHz, CD_3OD) δ_H 8.20 (dd, *J* 1.7 1.8 Hz, 1H), 7.97 (ddd, *J* 1.1 1.7 7.8 Hz, 1H), 7.81 (ddd, *J* 1.1 1.8 7.8 Hz, 1H), 7.43 (dd, *J* 7.8 7.8 Hz, 1H), 7.39 (s, 1H), 7.05 (dd, *J* 8.4 8.5 Hz, 1H), 6.60 (s, 1H), 6.58 (ddd, *J* 1.0 2.3 8.4 Hz, 1H), 6.11–6.07 (m, 2H), 3.84 (t, *J* 6.5 Hz, 2H), 3.70–3.52 (m, 10H), 3.46 (s, 3H), 3.35* (m, 2H), 3.27 (s, 3H), 1.95 (s, 3H), 1.83–1.75 (m, 2H), 1.06 (t, *J* 7.4 Hz, 3H); ^{13}C NMR (151 MHz, CD_3OD) δ_C 173.4, 167.9, 161.7, 159.6, 156.5, 146.7, 142.0, 136.2, 132.3, 131.11, 131.10, 130.1, 129.9, 127.3, 127.2, 123.0, 110.6, 110.3, 108.2, 105.3, 100.8, 71.32, 71.31, 70.59, 70.56, 70.5, 41.0, 40.4, 27.6, 27.5, 23.6, 22.5, 10.9; $\overline{\nu}_{max}$ (thin film)/cm^{-1} 2938 (w), 2878 (w), 1693 (s), 1642 (s), 1453 (s), 1153 (s), 1132 (s); LRMS (ESI$^+$) *m/z* 706 ([M+Na]$^+$, 100%); HRMS (ESI$^+$) *m/z* [Found: 684.2706, $C_{33}H_{42}N_5O_9S$ requires [M+H]$^+$ 684.2698]; HPLC retention time: 8.4 min, >99.9% (220 nm), >99.9% (254 nm), >99.9% (280 nm).

* Peak obscured by NMR solvent peak and was assigned by HSQC.

(33) 3-(*N*-(1,3-Dimethyl-2-oxo-6-(3-propoxyphenoxy)-2,3-dihydro-1*H*-benzo[*d*]imidazol-5-yl)sulfamoyl)-*N*-(2-oxo-6,9,12-trioxa-3-azatetradecan-14-yl)benzamide

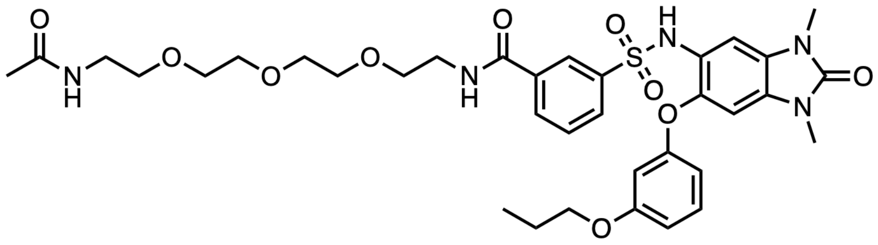

Compound **30** (79.8 mg, 102 μmol, 1.0 eq.) was deprotected according to the general Boc deprotection procedure to yield the crude amine which was directly acetylated according to the general acetylation procedure. The product was purified by semi-preparative reverse phase HPLC (gradient method 2, 20 mL min^{-1}, Agilent Prep C18 column [5 μm, 21.2 mm × 50 mm]) and lyophilised to yield the title compound as a colourless oil (46.7 mg, 63%): R_f 0.19 (MeOH/CH_2Cl_2, 1:19); ^{1}H NMR (600 MHz, CD_3OD) δ_H 8.20 (dd, *J* 1.9 1.9 Hz, 1H), 7.97 (ddd, *J* 1.2 1.9 7.8 Hz, 1H), 7.81 (ddd, *J* 1.2 1.9 7.8 Hz, 1H), 7.43 (dd, *J* 7.8 7.8 Hz, 1H), 7.39 (s, 1H), 7.09–7.03 (m, 1H), 6.63–6.56 (m, 2H), 6.11–6.06 (m, 2H), 3.84 (t, *J* 6.5 Hz, 2H), 3.72–3.50 (m, 14H), 3.47 (s, 3H), 3.34* (m, 2H), 3.27 (s, 3H), 1.95 (s, 3H), 1.83–1.74 (m, 2H), 1.06 (t, *J* 7.4 Hz, 3H); ^{13}C NMR (151 MHz, CD_3OD) δ_C 173.3, 167.9, 161.7, 159.6, 156.6, 146.7, 142.0, 136.2, 132.3, 131.1, 131.1, 130.1, 129.9, 127.3, 127.2, 123.1, 110.6, 110.3, 108.2, 105.3, 100.8, 71.59, 71.57, 71.3, 71.2, 70.6, 70.5, 70.4, 41.0, 40.4, 27.6, 27.5, 23.6, 22.5, 10.9; $\overline{\nu}_{max}$ (thin film)/cm^{-1} 2955 (w), 2881 (w), 1695 (s), 1643 (s), 1461 (s), 1151 (s), 1134 (s); LRMS (ESI$^+$) *m/z* 750 ([M+Na]$^+$, 100%); HRMS (ESI$^+$) *m/z* [Found: 728.2952, $C_{35}H_{46}N_5O_{10}S$ requires [M+H]$^+$ 728.2960]; HPLC retention time: 8.4 min, 97.1% (220 nm), 96.5% (254 nm), 95.1% (280 nm).

* Peak obscured by NMR solvent peak and was assigned by HSQC.

(34) 3-(*N*-(1,3-Dimethyl-2-oxo-6-(3-propoxyphenoxy)-2,3-dihydro-1*H*-benzo[*d*]imidazol-5-yl)sulfamoyl)-*N*-(2-oxo-6,9,12,15-tetraoxa-3-azaheptadecan-17-yl)benzamide

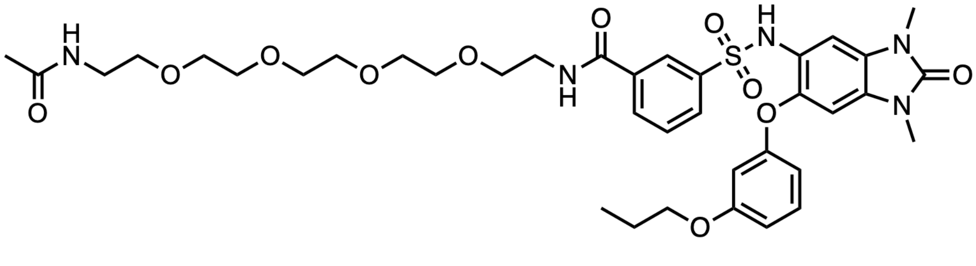

Compound **31** (77.2 mg, 93.0 μmol, 1.0 eq.) was deprotected according to the general Boc deprotection procedure to yield the crude amine which was directly acetylated according to the general acetylation procedure. The product was purified using semi-preparative reverse phase HPLC (gradient method 2, 20 mL min^{-1}, Agilent Prep C18 column [5 μm, 21.2 mm × 50 mm]) and lyophilised to yield the title compound as a colourless oil (31.1 mg, 43%): R_f 0.12 (MeOH/CH_2Cl_2, 1:19); ^{1}H NMR (600 MHz, CD_3OD) δ_H 8.21 (dd, *J* 1.8, 1.8 Hz, 1H), 7.97 (ddd, *J* 7.9, 1.8, 1.2 Hz, 1H), 7.82 (ddd, *J* 7.9, 1.8, 1.2 Hz, 1H), 7.44 (dd, *J* 7.9, 7.9 Hz, 1H), 7.39 (s, 1H), 7.09–7.03 (m, 1H), 6.64–6.55 (m, 2H), 6.12–6.06 (m, 2H), 3.84 (t, *J* 6.5 Hz, 2H), 3.69–3.50 (m, 18H), 3.47 (s, 3H), 3.35* (m, 2H), 3.27 (s, 3H), 1.96 (s, 3H), 1.84–1.74 (m, 2H), 1.06 (t, *J* 7.4 Hz, 3H); ^{13}C NMR (151 MHz, CD_3OD) δ_C 173.3, 167.9, 161.7, 159.6, 156.5, 146.7, 142.0, 136.2, 132.3, 131.1, 131.1, 130.1, 130.0, 127.3, 127.2, 123.1, 110.6, 110.3, 108.2, 105.3, 100.8, 71.57, 71.52, 71.52, 71.50, 71.3, 71.2, 70.6, 70.50, 70.45, 41.1, 40.5, 27.6, 27.5, 23.6, 22.5, 10.9; $\overline{\nu}_{max}$ (thin film)/cm^{-1} 2935 (w), 2878 (w), 1697 (s), 1642 (s), 1462 (s), 1152 (s), 1134 (s); LRMS (ESI$^+$) *m/z* 794 ([M+Na]$^+$, 100%); HRMS (ESI$^+$) *m/z* [Found: 772.3239, $C_{37}H_{50}N_5O_{11}S$ requires [M+H]$^+$ 772.3222]; HPLC retention time: 8.4 min, >99.9% (220 nm), >99.9% (254 nm), >99.9% (280 nm).

* Peak obscured by NMR solvent peak and was assigned by HSQC.

(35) *tert*-Butyl (*S*)-2-(((9*H*-fluoren-9-yl)methoxy)carbonyl)amino)pent-4-enoate

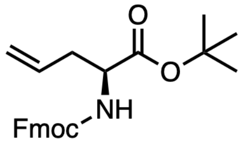

A solution of *tert*-butyl 2,2,2-trichloroacetimidate (10.6 mL, 59.3 mmol, 2.0 eq.) in cyclohexane (100 mL) was added dropwise over 30 min to a solution of Fmoc-L-allyl-glycine (10.0 g, 29.6 mmol, 1.0 eq.) in CH_2Cl_2 (100 mL) at rt. Boron trifluoride diethyl etherate (365 μL, 2.96 mmol, 0.1 eq.) was added and the reaction mixture was stirred at rt for 6 h. The reaction mixture was cooled to 0 °C and $NaHCO_3$ (8.20 g, 97.7 mmol, 3.3 eq.) was added. The reaction mixture was warmed to rt and stirred for 10 min. The reaction mixture was filtered through Celite® and the filtrate was concentrated *in vacuo*. The product was purified using flash silica column chromatography (gradient elution on a Biotage® system with 5–40% EtOAc/petroleum ether 40–60 °C) to yield the title compound as a colourless oil (8.08 g, 69%): R_f 0.48 (EtOAc/petroleum ether 40–60 °C, 1:4); $[\alpha]_D^{25}$ = +10.3 (*c*=1.0 in $CHCl_3$); ^{1}H NMR (400 MHz, $CDCl_3$) δ_H 7.80–7.74 (m, 2H), 7.63–7.57 (m, 2H), 7.44–7.37 (m, 2H), 7.35–7.28 (m, 2H), 5.80–5.64 (m, 1H), 5.36 (d, *J* 8.2 Hz, 1H), 5.19–5.08 (m, 2H), 4.51–4.31 (m, 3H), 4.23 (t, *J* 7.1 Hz,

1H), 2.66–2.44 (m, 2H), 1.48 (s, 9H); LRMS (ESI$^+$) *m/z* 416 ([M+Na]$^+$, 86%), 809 ([2M+Na]$^+$, 100%). The spectroscopic data are in good agreement with the literature values.[43]

(36) *tert*-Butyl (*S,E*)-2-((((9*H*-fluoren-9-yl)methoxy)carbonyl)amino)-6-hydroxyhex-4-enoate

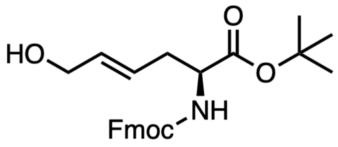

cis-Butene-1,4-diol (2.81 mL, 34.3 mmol, 3.00 eq.) and Grubbs 2nd generation catalyst (484 mg, 0.57 mmol, 0.05 eq.) were added to a solution of **35** (4.50 g, 11.4 mmol, 1.00 eq.) in anhydrous, degassed CH_2Cl_2 (50 mL) under an argon atmosphere at rt. The reaction solution was stirred and heated under reflux at 40 °C for 6 h. The reaction solution was cooled to rt and filtered through Celite®; and the filtrate was concentrated *in vacuo*. The product was purified using flash silica column chromatography (gradient elution using a Biotage® system with 12–100% EtOAc/petroleum ether 40–60 °C) to yield the title compound as a yellow oil (3.56 g, 70%): R_f 0.36 (EtOAc/petroleum ether 40–60 °C, 1:1); $[\alpha]_D^{25}$ = +14.4 (*c*=1.0 in $CHCl_3$); ^{1}H NMR (600 MHz, $CDCl_3$) δ_H 7.77 (d, *J* 7.6 Hz, 2H), 7.60 (d, *J* 7.6 Hz, 2H), 7.40 (t, *J* 7.6 Hz, 2H), 7.32 (t, *J* 7.6 Hz, 2H), 5.74 (dt, *J* 6.0 15.2 Hz, 1H), 5.66–5.56 (m, 1H), 5.38 (d, *J* 7.9 Hz, 1H), 4.44–4.29 (m, 3H), 4.23 (t, *J* 7.0 Hz, 1H), 4.09 (t, *J* 6.0 Hz, 2H), 2.66–2.42 (m, 2H), 1.48 (s, 9H); ^{13}C NMR (151 MHz, $CDCl_3$) δ_C 170.9, 155.8, 144.1, 144.0, 141.5, 133.7, 127.9, 127.2, 126.0, 125.3, 125.2, 120.14, 120.13, 82.5, 67.1, 63.4, 54.0, 47.3, 35.7, 28.2; $\overline{\nu}_{max}$ (thin film)/cm^{-1} 3327 (br, s), 2978 (w), 1718 (s), 1248 (m), 1155 (s), 1047 (m), 741 (s); LRMS (ESI$^+$) *m/z* 446 ([M+Na]$^+$, 85%), 869 ([2M+Na]$^+$, 100%); HRMS (ESI$^+$) *m/z* [Found: 446.1934, $C_{25}H_{29}NO_5Na$ requires [M+Na]$^+$ 446.1938]; HPLC retention time: 10.4 min, 93.5% (220 nm), 93.8% (254 nm), 93.7% (280 nm).

(37) *tert*-Butyl (*S*)-2-((((9*H*-fluoren-9-yl)methoxy)carbonyl)amino)hexanoate

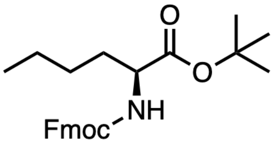

To a solution of **36** (3.65 g, 8.62 mmol, 1.0 eq.) in EtOH (100 mL) was added 10% Pd/C (916 mg, 0.86 mmol, 0.1 eq.) under an argon atmosphere at rt. The flask was purged with hydrogen gas *via* 3 successive evacuate/fill cycles and the reaction mixture was stirred vigorously under a hydrogen atmosphere at rt for 16 h. The reaction mixture was filtered through Celite® and the filtrate was concentrated *in vacuo*. The product was purified using

flash silica column chromatography (gradient elution on a Biotage® system with 2–20% EtOAc/petroleum ether 40–60 °C) to yield the title compound as a colourless oil (1.32 g, 37%): R_f 0.32 (EtOAc/petroleum ether 40–60 °C, 1:9); $[\alpha]_D^{25}$ = + 4.3 (*c*=1.0 in $CHCl_3$); ^1H NMR (600 MHz, $CDCl_3$) δ_H 7.77 (d, *J* 7.6 Hz, 2H), 7.64–7.58 (m, 2H), 7.40 (t, *J* 7.6 Hz, 2H), 7.32 (t, *J* 7.6 Hz, 2H), 5.31 (d, *J* 8.3 Hz, 1H), 4.39 (d, *J* 7.3 Hz, 2H), 4.31–4.21 (m, 2H), 1.90–1.62 (m, 2H), 1.48 (s, 9H), 1.42–1.24 (m, 4H), 0.91 (t, *J* 7.1 Hz, 3H); ^{13}C NMR (151 MHz, $CDCl_3$) δ_C 172.0, 156.0, 144.1, 144.0, 141.5, 127.8, 127.2, 125.3, 120.11, 120.09, 82.1, 67.0, 54.5, 47.4, 32.7, 28.2, 27.3, 22.5, 14.0; $\overline{\nu}_{max}$ (thin film)/cm^{-1} 2959 (w), 1724 (s), 1156 (s), 740 (s); LRMS (ESI$^+$) *m/z* 432 ([M+Na]$^+$, 18%), 841 ([2M+Na]$^+$, 100%); HRMS (ESI$^+$) *m/z* [Found: 432.2142, $C_{25}H_{31}NO_4Na$ requires [M+Na]$^+$ 432.2145]; HPLC retention time: 13.5 min, 96.0% (220 nm), 97.9% (254 nm), 96.7% (280 nm).

(38) *tert*-Butyl (*S*)-2-((((9*H*-fluoren-9-yl)methoxy)carbonyl)amino)-6-hydroxyhexanoate

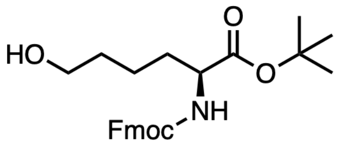

To a solution of **36** (4.32 g, 10.2 mmol, 1.0 eq.) in EtOAc (150 mL) was added PtO_2 (232 mg, 1.02 mmol, 0.1 eq.) under an argon atmosphere at rt. The flask was purged with hydrogen gas *via* 3 successive evacuate/fill cycles and the reaction mixture was stirred vigorously under a hydrogen atmosphere at rt for 20 h. The reaction mixture was filtered through Celite® and the filtrate was concentrated *in vacuo*. The product was purified using flash silica column chromatography (gradient elution on a Biotage® system with 12–100% EtOAc/petroleum ether 40–60 °C) to yield the title compound as a yellow oil (3.24 g, 75%): R_f 0.33 (EtOAc/petroleum ether 40–60 °C, 1:1); $[\alpha]_D^{25}$ = +3.0 (*c*=1.0 in $CHCl_3$); ^1H NMR (600 MHz, $CDCl_3$) δ_H 7.77 (d, *J* 7.6 Hz, 2H), 7.60 (d, *J* 7.6 Hz, 2H), 7.40 (t, *J* 7.6 Hz, 2H), 7.32 (t, *J* 7.6 Hz, 2H), 5.34 (d, *J* 8.1 Hz, 1H), 4.39 (d, *J* 7.2 Hz, 2H), 4.32–4.19 (m, 2H), 3.70–3.58 (m, 2H), 2.00–1.34 (m, 15H); ^{13}C NMR (151 MHz, $CDCl_3$) δ_C 171.8, 156.1, 144.1, 144.0, 141.5, 127.8, 127.2, 125.3, 120.13, 120.11, 82.3, 67.1, 62.7, 54.3, 47.4, 32.8, 32.3, 28.2, 21.5; $\overline{\nu}_{max}$ (thin film)/cm^{-1} (br, w), 2930 (w), 1727 (s), 1155 (s), 760 (s), 742 (s); LRMS (ESI$^+$) *m/z* 448 ([M+Na]$^+$, 27%), 873 ([2M+Na]$^+$, 100%); HRMS (ESI$^+$) *m/z* [Found: 448.2092, $C_{25}H_{31}NO_5Na$ requires [M+Na]$^+$ 448.2094]; HPLC retention time: 11.3 min, 96.1% (220 nm), 99.2% (254 nm), 99.1% (280 nm).

(39) 1-((9*H*-Fluoren-9-yl)methyl) 2-(*tert*-butyl) (*S*)-3,6-dihydropyridine-1,2(2*H*)-dicarboxylate

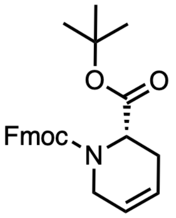

Tetrabromomethane (2.03 g, 6.13 mmol, 1.5 eq.) was added to a solution of **36** (1.73 g, 4.08 mmol, 1.0 eq.) and PPh_3 (1.61 g, 6.13 mmol, 1.5 eq.) in anhydrous CH_2Cl_2 (20 mL) at 0 °C. The reaction solution was warmed to rt and stirred for 2 h. The reaction solution was diluted with Et_2O (50 mL), and the resulting mixture was filtered. The filtrate was concentrated *in vacuo* and the product was purified by flash silica column chromatography (gradient elution on a Biotage® system with 5-40% EtOAc/petroleum ether 40–60 °C) to yield the title compound as a colourless oil (1.05 g, 63%): R_f 0.55 (EtOAc/petroleum ether 40–60 °C, 1:4); $[\alpha]_D^{25}$ = –16.2 (*c*=0.5 in $CHCl_3$); ^{1}H NMR (500 MHz, D_6-DMSO, T=393 K) δ_H 7.89–7.83 (m, 2H), 7.67–7.59 (m, 2H), 7.46–7.38 (m, 2H), 7.37–7.29 (m, 2H), 6.76–6.64 (m, 1H), 4.94–4.84 (m, 1H), 4.63–4.50 (m, 2H), 4.45 (dd, *J* 6.4 10.7 Hz, 1H), 4.30 (t, *J* 6.4 Hz, 1H), 2.26–2.16 (m, 1H), 2.06–1.72 (m, 3H), 1.40 (s, 9H); ^{13}C NMR (126 MHz, D_6-DMSO, T=393 K) δ_C 168.6, 152.1, 143.2, 143.1, 140.27, 140.25, 127.0, 126.5, 124.1, 123.5, 119.3, 104.8, 80.5, 66.8, 53.6, 46.3, 27.1, 22.7, 17.3; $\bar{\nu}_{max}$ (thin film)/cm^{-1} 2978 (w), 1741 (s), 1712 (s), 1658 (m), 1155 (s), 758 (s), 740 (s); LRMS (ESI$^+$) *m/z* 428 ([M+Na]$^+$, 47%), 833 ([2M+Na]$^+$, 100%); HRMS (ESI$^+$) *m/z* [Found: 428.1836, $C_{25}H_{27}NO_4Na$ requires [M+Na]$^+$ 428.1832]; HPLC retention time: 13.5 min, 96.1% (220 nm), 95.6% (254 nm), 95.8% (280 nm).

(40) *tert*-Butyl (*S*,*E*)-2-(((9*H*-fluoren-9-yl)methoxy)carbonyl)amino)-6-((methylsulfonyl)oxy)hex-4-enoate

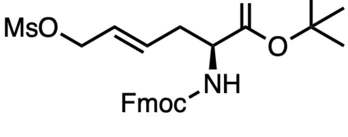

Methanesuflonyl chloride (1.11 mL, 14.3 mmol, 1.5 eq.) was added dropwise to a solution of **36** (4.05 g, 9.56 mmol, 1.0 eq.) and NEt_3 (2.66 mL, 19.1 mmol, 2.0 eq.) in CH_2Cl_2 (30 mL) at 0 °C. The reaction mixture was warmed to rt and stirred for 1 h. The reaction mixture was diluted with $CHCl_3$ (50 mL) and washed with sat. aq. $NaHCO_3$ (50 mL). The organic component was dried (Na_2SO_4), filtered, and concentrated *in vacuo*. The product was purified by flash silica column chromatography (gradient elution on a Biotage® system with 12–60% EtOAc/petroleum ether 40–60 °C) to yield the title compound as a colourless oil (3.96 g, 83%):

R_f 0.48 (EtOAc/petroleum ether 40–60 °C, 1:1); $[\alpha]_D^{25}$ = +20.0 (*c*=1.0 in $CHCl_3$); ^{1}H NMR (600 MHz, $CDCl_3$) δ_H 7.77 (d, *J* 7.5 Hz, 2H), 7.63–7.56 (m, 2H), 7.43–7.38 (m, 2H), 7.35–7.29 (m, 2H), 5.86–5.64 (m, 2H), 5.39 (d, *J* 7.8 Hz, 2H), 4.71–4.60 (m, 2H), 4.47–4.30 (m, 3H), 4.22 (t, *J* 6.8 Hz, 1H), 2.96 (s, 3H), 2.70–2.46 (m, 2H), 1.48 (s, 9H); ^{13}C NMR (151 MHz, $CDCl_3$) δ_C 170.5, 155.8, 144.0, 143.9, 141.5, 132.4, 127.9, 127.2, 126.7, 125.24, 125.20, 120.16, 120.15, 82.9, 69.8, 67.1, 53.7, 47.3, 38.3, 35.6, 28.2; $\bar{\nu}_{max}$ (thin film)/cm^{-1} 2980 (w), 1726 (s), 1354 (s), 1173 (s), 1157 (s), 927 (s), 742 (s); LRMS (ESI$^+$) *m/z* 524 ([M+Na]$^+$, 69%), 1025 ([2M+Na]$^+$, 100%); HRMS (ESI$^+$) *m/z* [Found: 524.1713, $C_{26}H_{31}NO_7SNa$ requires [M+Na]$^+$ 524.1714].

(41) *tert*-Butyl (*S*,*E*)-2-(((9*H*-fluoren-9-yl)methoxy)carbonyl)amino)-6-(isoindolin-2-yl)hex-4-enoate

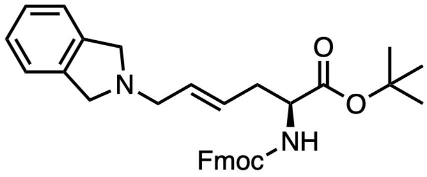

A solution of **40** (2.65 g, 5.28 mmol, 1.0 eq.) in anhydrous DMF (20 mL) was added dropwise to mixture of isoindoline hydrochloride (904 mg, 5.81 mmol, 1.1 eq.), K_2CO_3 (2.18 g, 15.8 mmol, 3.0 eq.), and KI (964 mg, 5.81 mmol, 1.1 eq.) in anhydrous DMF (30 mL) at rt. The reaction mixture was stirred at rt for 1 h and quenched with H_2O (10 mL). The reaction mixture was concentrated *in vacuo* and the residue was diluted with aq. LiCl (0.5 M, 50 mL). The aqueous phase was extracted with EtOAc (3 × 50 mL) and the organic components were combined, washed with aq. LiCl (0.5 M, 50 mL), brine (50 mL), dried (Na_2SO_4), filtered, and concentrated *in vacuo*. The product was purified by flash silica column chromatography (gradient elution with 1–3% 2 N NH_3 in MeOH/CH_2Cl_2) to yield the title compound as a yellow oil (1.97 g, 71%): R_f 0.63 (2 N NH_3 in MeOH/CH_2Cl_2, 1:9); $[\alpha]_D^{25}$ = +14.6 (*c*=1.0 in $CHCl_3$); ^{1}H NMR (600 MHz, $CDCl_3$) δ_H 7.76 (d, *J* 7.6 Hz, 2H), 7.61 (d, *J* 7.6 Hz, 2H), 7.39 (t, *J*, 7.6 Hz, 2H), 7.31 (t, *J* 7.6 Hz, 2H), 7.21–7.13 (m, 4H), 5.74 (dt, *J* 15.2 6.4 Hz, 1H), 5.63 (dt, *J* 15.2 7.2, 1H), 5.40 (d, *J* 8.0 Hz, 1H), 4.43–4.32 (m, 3H), 4.24 (t, *J* 7.2 Hz, 1H), 3.93 (s, 4H), 3.35 (d, *J* 6.4 Hz, 2H), 2.72–2.44 (m, 2H), 1.49 (s, 9H); ^{13}C NMR (151 MHz, $CDCl_3$) δ_C 171.0, 155.8, 144.1, 144.0, 141. 4, 140.0, 132.0, 127.8, 127.22, 127.19, 126.9, 125.3, 122.4, 120.1, 82.5, 67.2, 58.9, 57.8, 54.1, 47.3, 35.8, 28.2; $\bar{\nu}_{max}$ (thin film)/cm^{-1} 2932 (w), 1722 (s), 1156 (s), 743 (s); LRMS (ESI$^+$) *m/z* 525 ([M+H]$^+$, 100%); HRMS (ESI$^+$) *m/z* [Found: 547.2572, $C_{33}H_{36}N_2O_4Na$ requires [M+Na]$^+$ 547.2567]; HPLC retention time: 9.7 min, 93.8% (220 nm), 96.3% (254 nm), 93.0% (280 nm).

(42) *tert*-Butyl (*S*)-2-((((9*H*-fluoren-9-yl)methoxy)carbonyl)amino)-6-(isoindolin-2-yl)hexanoate

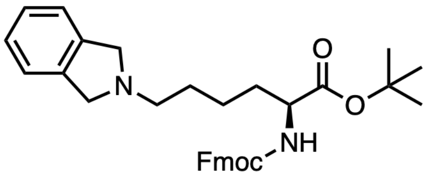

To a solution of **41** (253 mg, 0.48 mmol, 1.0 eq.) in EtOAc (25 mL) was added PtO_2 (11.4 mg, 0.05 mmol, 0.1 eq.) under an argon atmosphere at rt. The flask was purged with hydrogen gas *via* 3 successive evacuate/fill cycles and the reaction mixture was stirred vigorously under a hydrogen atmosphere at rt for 6 h. The reaction mixture was filtered through Celite® and the filtrate was concentrated *in vacuo*. The product was purified by flash silica column chromatography (gradient elution with 1–3% 2 M NH_3 in MeOH/CH_2Cl_2) to yield the title compound as a yellow oil (209 mg, 83%): R_f 0.60 (2 M NH_3 in MeOH/CH_2Cl_2, 1:9); $[\alpha]_D^{25}$ = +8.4 (*c*=1.0 in $CHCl_3$); ^{1}H NMR (600 MHz, $CDCl_3$) δ_H 7.76 (d, *J* 7.6 Hz, 2H), 7.63–7.57 (m, 2H), 7.42–7.37 (m, 2H), 7.34–7.28 (m, 2H), 7.22–7.13 (m, 4H), 5.39 (d, *J* 8.2 Hz, 1H), 4.38 (d, *J* 7.2 Hz, 2H), 4.32–4.25 (m, 1H), 4.22 (t, *J* 7.2 Hz, 1H), 3.95 (s, 4H), 2.74 (t, *J* 7.5 Hz, 2H), 1.94–1.84 (m, 1H), 1.78–1.32 (m, 14H); ^{13}C NMR (151 MHz, $CDCl_3$) δ_C 171.8, 156.0, 144.1, 144.0, 141.5, 139.9, 127.8, 127.2, 126.9, 125.3, 122.4, 120.12, 120.10, 82.2, 67.1, 59.2, 55.9, 54.4, 47.4, 32.8, 28.5, 28.2, 23.0; $\overline{\nu}_{max}$ (thin film)/cm^{-1} 2936 (w), 1722 (s), 1156 (s), 762 (s), 744 (s); LRMS (ESI$^+$) *m/z* 527 ([M+H]$^+$, 100%); HRMS (ESI$^+$) *m/z* [Found: 527.2904, $C_{33}H_{39}N_2O_4$ requires [M+H]$^+$ 527.2904]; HPLC retention time: 9.6 min, 92.1% (220 nm), 95.0% (254 nm), 94.1% (280 nm).

(43) (9*H*-Fluoren-9-yl)methyl (*S*)-(1-(3-(*N*-(1,3-dimethyl-2-oxo-6-(3-propoxyphenoxy)-2,3-dihydro-1*H*-benzo[*d*]imidazol-5-yl)sulfamoyl)phenyl)-17-(isoindolin-2-yl)-1,12-dioxo-5,8-dioxa-2,11-diazaheptadecan-13-yl)carbamate

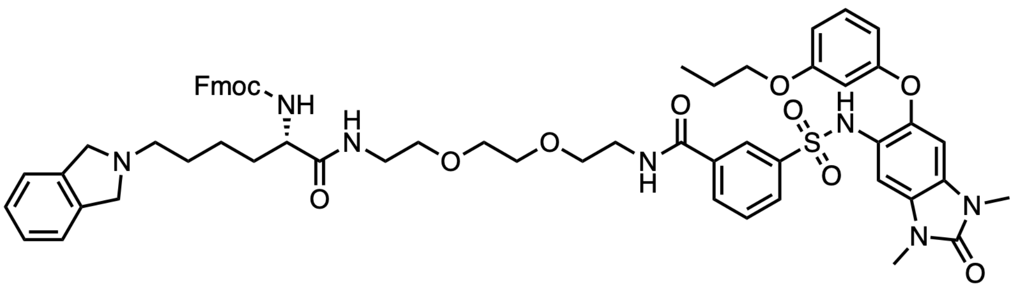

Compounds **29** (67.5 mg, 0.09 mmol, 1.0 eq.) and **42** (47.9 mg, 0.09 mmol, 1.0 eq.) were deprotected according to the general Boc and ^{t}Bu ester deprotection procedures. The crude amine and carboxylic acid were then reacted directly according to general amide coupling procedure 2. The product was purified by flash silica column chromatography (gradient elution

with 2–6% MeOH/CH_2Cl_2) to yield the title compound as a brown oil (62.7 mg, 64%): R_f 0.30 (MeOH/CH_2Cl_2, 1:11.5); $[\alpha]_D^{25}$ = +11.3 (*c*=0.1 in $CHCl_3$); ^{1}H NMR (600 MHz, $CDCl_3$) δ_H 8.26–8.19 (m, 1H), 7.96–7.90 (m, 1H), 7.77–7.63 (m, 3H), 7.57–7.51 (m, 2H), 7.42–7.21 (m, 5H), 7.20–7.12 (m, 5H), 7.07–6.95 (m, 1H), 6.55–6.42 (m, 1H), 6.34 (s, 1H), 6.10–5.95 (m, 2H), 4.42–4.24 (m, 2H), 4.19 (t, *J* 7.1 Hz, 1H), 4.13 (t, *J* 7.3 Hz, 1H), 4.00 (s, 4H), 3.77 (t, *J* 6.3 Hz, 2H), 3.67–3.30 (m, 15H), 3.19 (s, 3H), 2.83 (t, *J* 6.5 Hz, 2H), 1.90–1.58 (m, 6H), 1.54–1.39 (m, 2H), 0.99 (t, *J* 7.4 Hz, 3H); ^{13}C NMR (151 MHz, $CDCl_3$) δ_C 172.3, 165.7, 160.5, 158.2, 156.6, 154.9, 143.92, 143.87, 141.4, 141.3, 139.9, 138.6, 135.4, 132.0, 130.3, 129.9, 129.1, 128.7, 127.8, 127.4, 127.2, 126.5, 125.7, 125.3, 125.2, 122.5, 121.5, 120.1, 109.4, 109.1, 105.4, 104.2, 99.4, 70.3. 69.7, 67.1, 59.1, 55.7, 47.2, 40.1, 40.0, 39.3, 39.2, 32.5, 27.7, 27.5, 27.4, 23.1, 22.6, 10.7; $\bar{\nu}_{max}$ (thin film)/cm^{-1} 2935 (w), 1699 (s), 1653 (s), 1453 (s), 1151 (C-O, s), 846 (s), 741 (s); LRMS (ESI$^+$) *m/z* 1094 ([M+H]$^+$, 100%); HRMS (ESI$^+$) *m/z* [Found: 1116.4519, $C_{60}H_{67}N_7O_{11}SNa$ requires [M+Na]$^+$ 1116.4512]; HPLC retention time: 9.2 min, 83.8% (220 nm), 90.1% (254 nm), 92.2% (280 nm).

(44) (9*H*-Fluoren-9-yl)methyl (*S*)-(1-(3-(*N*-(1,3-dimethyl-2-oxo-6-(3-propoxyphenoxy)-2,3-dihydro-1*H*-benzo[*d*]imidazol-5-yl)sulfamoyl)phenyl)-20-(isoindolin-2-yl)-1,15-dioxo-5,8,11-trioxa-2,14-diazaicosan-16-yl)carbamate

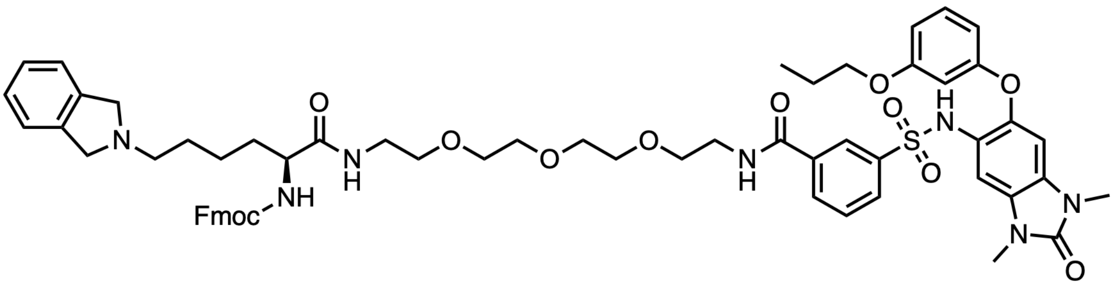

Compounds **30** (71.1 mg, 0.09 mmol, 1.0 eq.) and **42** (47.6 mg, 0.09 mmol, 1.0 eq.) were deprotected according to the general Boc and *t*-Bu deprotection procedures. The crude amine and carboxylic acid were then reacted directly according to general amide coupling procedure 2. The product was purified by flash silica column chromatography (gradient elution with 2–7% MeOH/CH_2Cl_2) to yield the title compound as a brown oil (50.2 mg, 49%): R_f 0.38 (MeOH/CH_2Cl_2, 1:9); $[\alpha]_D^{25}$ = −12.1 (*c*=0.1 in $CHCl_3$); ^{1}H NMR (600 MHz, $CDCl_3$) δ_H 8.26–8.19 (m, 1H), 7.99–7.92 (m, 1H), 7.72 (d, *J* 7.6 Hz, 2H), 7.67 (d, *J* 8.1 Hz, 1H), 7.57–7.52 (m, 2H), 7.41–7.30 (m, 3H), 7.29–7.23 (m, 2H), 7.22 (s, 1H), 7.20–7.12 (m, 4H), 7.02 (t, *J* 8.2 Hz, 1H), 6.56–6.48 (m, 1H), 6.34 (s, 1H), 6.10–6.00 (m, 2H), 4.45–4.24 (m, 2H), 4.20 (t, *J* 6.4 Hz, 1H), 4.15 (t, *J* 7.3 Hz, 1H), 3.99 (s, 4H), 3.78 (t, *J* 6.6 Hz, 2H), 3.66–3.42 (m, 14H), 3.38 (s, 3H), 3.36–3.30 (m, 2H), 3.20 (s, 3H), 2.79 (t, *J* 7.3 Hz, 2H), 1.91–1.54 (m, 6H), 1.50–1.39 (m, 2H), 1.00 (t, *J* 7.4 Hz, 3H); ^{13}C NMR (151 MHz, $CDCl_3$) δ_C 172.2, 165.7, 160.5, 158.1, 156.4, 154.9,

143.94, 143.89, 143.77, 141.4, 139.7, 138.9, 135.5, 132.1, 130.3, 129.8, 129.1, 128.7, 127.8, 127.24, 127.19, 126.5, 125.7, 125.24, 125.21, 122.5, 121.4, 120.1, 109.4, 109.3, 105.1, 104.2, 99.4, 70.43, 70.38, 70.2, 70.1, 69.73, 69.71, 67.1, 59.1, 55.7, 54.9, 54.8, 47.2, 40.1, 39.9, 39.3, 39.2, 32.8, 27.8, 27.5, 27.4, 23.0, 22.6, 10.6; $\overline{\nu}_{max}$ (thin film)/cm^{-1} 2943 (w), 1700 (s), 1654 (s), 1456 (s), 1152 (s), 847 (s), 744 (s); LRMS (ESI$^+$) *m/z* 1138 ([M+H]$^+$, 100%); HRMS (ESI$^+$) *m/z* [Found: 1138.4966, $C_{62}H_{72}N_7O_{12}S$ requires [M+H]$^+$ 1138.4954]; HPLC retention time: 9.2 min, 90.5% (220 nm), 93.6% (254 nm), 93.6% (280 nm).

(45) (9*H*-Fluoren-9-yl)methyl (*S*)-(1-(3-(*N*-(1,3-dimethyl-2-oxo-6-(3-propoxyphenoxy)-2,3-dihydro-1*H*-benzo[*d*]imidazol-5-yl)sulfamoyl)phenyl)-23-(isoindolin-2-yl)-1,18-dioxo-5,8,11,14-tetraoxa-2,17-diazatricosan-19-yl)carbamate

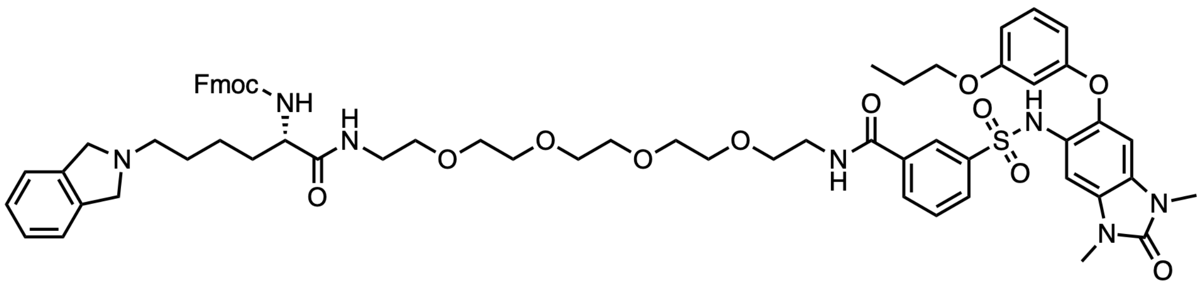

Compounds **31** (62.2 mg, 0.07 mmol, 1.0 eq.) and **42** (39.5 mg, 0.07 mmol, 1.0 eq.) were deprotected according to the general Boc and *t*-Bu deprotection procedures. The crude amine and carboxylic acid were then reacted directly according to general amide coupling procedure 2. The product was purified by flash silica column chromatography (gradient elution with 2-8% MeOH/CH_2Cl_2) to yield the title compound as a brown oil (63.7 mg, 77%): R_f 0.42 (MeOH/CH_2Cl_2, 1:9); $[\alpha]_D^{25}$ = –3.6 (*c*=0.1 in $CHCl_3$); ^{1}H NMR (600 MHz, $CDCl_3$) δ_H 8.28 (m, 1H), 7.99–7.93 (m, 1H), 7.73 (d, *J* 7.6 Hz, 2H), 7.69 (d, *J* 8.0 Hz, 1H), 7.59–7.52 (m, 2H), 7.37 (t, *J* 7.5 Hz, 2H), 7.32 (t, *J* 8.0 Hz, 1H), 7.30–7.24 (m, 2H), 7.23 (s, 1H), 7.20–7.12 (m, 4H), 7.02 (t, *J* 8.1 Hz, 1H), 6.58–6.49 (m, 1H), 6.36 (s, 1H), 6.10–6.00 (m, 2H), 4.43–4.27 (m, 2H), 4.26–4.20 (m, 1H), 4.17 (t, *J* 7.3 Hz, 1H), 3.96 (s, 4H), 3.79 (t, *J* 6.5 Hz, 2H), 3.68–3.31 (m, 23H), 3.21 (s, 3H), 2.80–2.71 (m, 2H), 1.92–1.52 (m, 6H), 1.50–1.38 (m, 2H), 1.00 (t, *J* 7.4 Hz, 3H); ^{13}C NMR (151 MHz, $CDCl_3$) δ_C 172.0, 165.6, 160.5, 158.1, 156.3, 154.9, 144.0, 143.9, 143.7, 141.4, 139.8, 139.2, 135.6, 132.1, 130.3, 129.8, 129.0, 128.7, 127.8, 127.18, 127.11, 126.6, 125.9, 125.25, 125.22, 122.4, 121.5, 120.1, 109.4, 109.2, 105.1, 104.2, 99.4, 70.5, 70.3, 70.2, 69.8, 69.73, 69.71, 67.0, 59.0, 55.8, 54.9, 47.2, 40.1, 39.4, 33.1, 28.0, 27.5, 27.4, 23.1, 22.6, 10.7; $\overline{\nu}_{max}$ (thin film)/cm^{-1} 2929 (w), 1703 (s), 1663 (s), 1462 (s), 1260 (s), 1132 (s), 741 (s); LRMS (ESI$^+$) *m/z* 1182 ([M+H]$^+$, 100%); HRMS (ESI$^+$) *m/z* [Found: 1182.5219, $C_{64}H_{76}N_7O_{13}S$ requires [M+H]$^+$ 1182.5216]; HPLC retention time: 9.3 min, 91.2% (220 nm), 95.1% (254 nm), 96.4% (280 nm).

(46) N^2-(N-(N^2-(N^6-(*tert*-Butoxycarbonyl)-N^2-(N-(N^2-((*tert*-butoxycarbonyl)-*L*-alanyl)-N^{ω}-((2,2,4,6,7-pentamethyl-2,3-dihydrobenzofuran-5-yl)sulfonyl)-*L*-arginyl)-*O*-(*tert*-butyl)-*L*-threonyl)-*L*-lysyl)-N^5-trityl-*L*-glutaminyl)-*O*-(*tert*-butyl)-*L*-threonyl-*L*-alanyl)-N^{ω}-((2,2,4,6,7-pentamethyl-2,3-dihydrobenzofuran-5-yl)sulfonyl)-*L*-arginine diformic acid (PHD peptide 1)

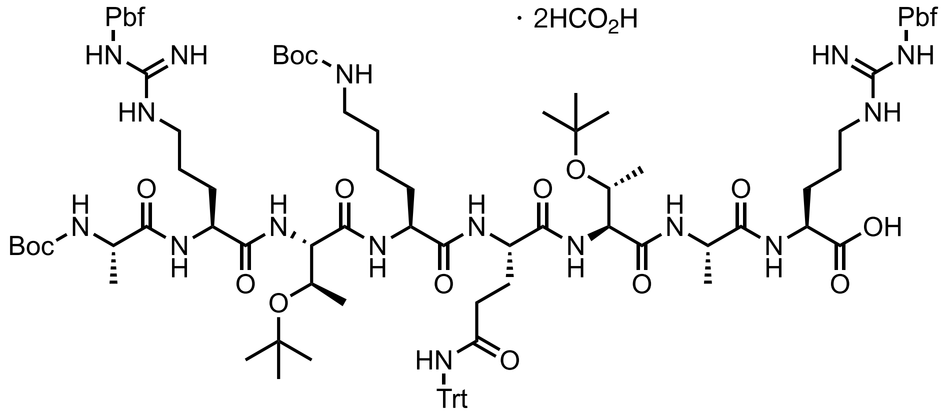

The title compound was synthesised *via* SPPS on H-Arg(Pbf)-2-ClTrt resin (1.00 g, 0.30–0.90 mmol/g loading, Novabiochem®) using a Liberty Blue™ 2.0 Automated Microwave Peptide Synthesiser (CEM). Amino acids (5 eq.) were coupled in the following order: Fmoc-Ala-OH, Fmoc-Thr(*t*-Bu)-OH, Fmoc-Gln(Trt)-OH, Fmoc-Lys(Boc)-OH, Fmoc-Thr(*t*-Bu)-OH, Fmoc-Arg(Pbf)-OH, Boc-Ala-OH. Coupling reactions were carried out using an activator solution of *N,N'*-diisopropylcarbodiimide (7.8 mL/100 mL) in ultrapure DMF and an additive solution of Oxyma Pure (14.2 g/100 mL) and DIPEA (1.5% *v/v*) in ultrapure DMF. Fmoc deprotection steps were carried out using a solution of piperidine in ultrapure DMF (20% *v/v*). After the final coupling step the resin was washed with CH_2Cl_2 (20 mL), MeOH (20 mL), and CH_2Cl_2 (20 mL). The peptide was cleaved from the resin with a solution of hexafluoro-2-propanol in CH_2Cl_2 (30% *v/v*) for 1 h. The resin was filtered and washed with CH_2Cl_2; and the filtrate was concentrated *in vacuo*. The product was purified by semi-preparative reverse phase HPLC (gradient method 1, 40 mL min^{-1}, Agilent Zorbax PrepHT column [7 µm, 21.2 mm × 250 mm]) and lyophilised to yield the title compound as a colourless solid (482 mg): R_f 0.22 (MeOH/CH_2Cl_2, 1:9); LRMS (ESI$^+$) *m/z* 996 ([M–2FA+2H]$^{2+}$, 100%), 1990 ([M–2FA+H]$^+$, 40%); HRMS (ESI$^+$) *m/z* [Found: 1990.0480, $C_{100}H_{149}N_{16}O_{22}S_2$ requires [M–2FA+H]$^+$ 1990.0468]; HPLC retention time: 13.6 min, 97.9% (220 nm), 96.7% (254 nm), 95.4% (280 nm).

(47) *tert*-Butyl ((13*S*,16*S*,19*S*,22*S*,25*S*,28*S*)-22-((*R*)-1-(*tert*-butoxy)ethyl)-28-((6*S*,9*S*,12*S*)-12-((*R*)-1-(*tert*-butoxy)ethyl)-2,2,6-trimethyl-4,7,10-trioxo-9-(3-(3-((2,2,4,6,7-pentamethyl-2,3-dihydrobenzofuran-5-yl)sulfonyl)guanidino)propyl)-3-oxa-5,8,11-triazatridecan-13-amido)-1-(3-(*N*-(1,3-dimethyl-2-oxo-6-(3-propoxyphenoxy)-2,3-dihydro-1*H*-benzo[*d*]imidazol-5-yl)sulfamoyl)phenyl)-13-(4-(isoindolin-2-yl)butyl)-19-methyl-1,12,15,18,21,24,27-heptaoxo-25-(3-oxo-3-(tritylamino)propyl)-16-(3-(3-((2,2,4,6,7-pentamethyl-2,3-dihydrobenzofuran-5-yl)sulfonyl)guanidino)propyl)-5,8-dioxa-2,11,14,17,20,23,26-heptaazadotriacontan-32-yl)carbamate (PDC1)

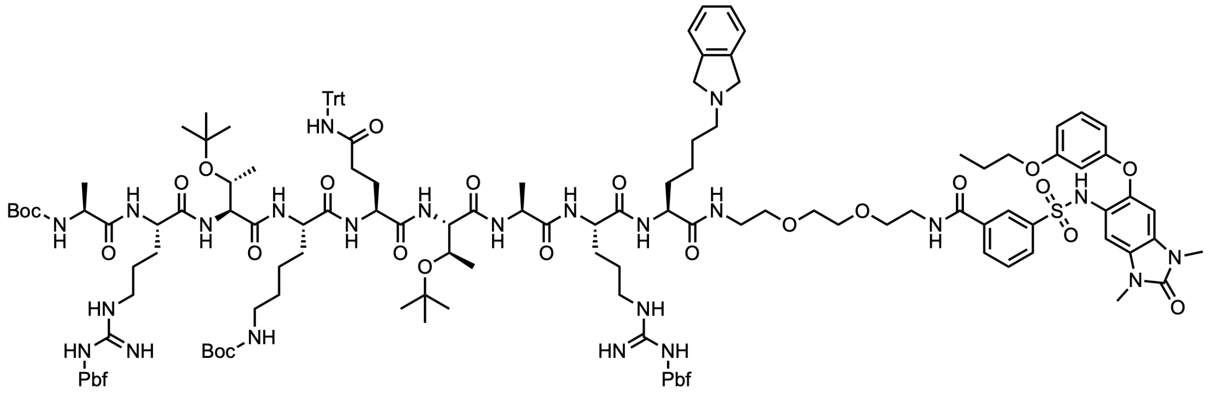

Compound **43** was reacted according to the general Fmoc deprotection procedure to yield the purified amine (diformate salt, 14.6 mg, 15.1 μmol, 1.0 eq.) which was reacted with **46** (31.4 mg, 15.1 μmol, 1.0 eq.) according to general amide coupling procedure 3. The product was purified by flash silica column chromatography (gradient elution with 2–6% (1% NH_4OH in MeOH)/CH_2Cl_2 then gradient elution on a Biotage® system with 2–20% MeOH/CH_2Cl_2) to yield the title compound as a colourless oil (22.5 mg, 52%): R_f 0.40 (1% NH_4OH in MeOH/CH_2Cl_2, 1:9); LRMS (ESI$^+$) *m/z* 1422 ([M+2H]$^{2+}$, 100%); 1433 ([M+H+Na]$^{2+}$, 54%); HRMS (ESI$^+$) *m/z* [Found: 2843.4341, $C_{145}H_{204}N_{23}O_{30}S_3$ requires [M+H]$^+$ 2843.4301]; HPLC retention time: 12.1 min, 95.4% (220 nm), 95.3% (254 nm), 96.5% (280 nm).

(48) *tert*-Butyl ((16*S*,19*S*,22*S*,25*S*,28*S*,31*S*,34*S*,37*S*,40*S*)-25,34-bis((*R*)-1-(*tert*-butoxy)ethyl)-31-(4-((*tert*-butoxycarbonyl)amino)butyl)-1-(3-(*N*-(1,3-dimethyl-2-oxo-6-(3-propoxyphenoxy)-2,3-dihydro-1*H*-benzo[*d*]imidazol-5-yl)sulfamoyl)phenyl)-16-(4-(isoindolin-2-yl)butyl)-22-methyl-1,15,18,21,24,27,30,33,36,39-decaoxo-28-(3-oxo-3-(tritylamino)propyl)-19,37-bis(3-(3-((2,2,4,6,7-pentamethyl-2,3-dihydrobenzofuran-5-yl)sulfonyl)guanidino)propyl)-5,8,11-trioxa-2,14,17,20,23,26,29,32,35,38-decaazahentetracontan-40-yl)carbamate (PDC2)

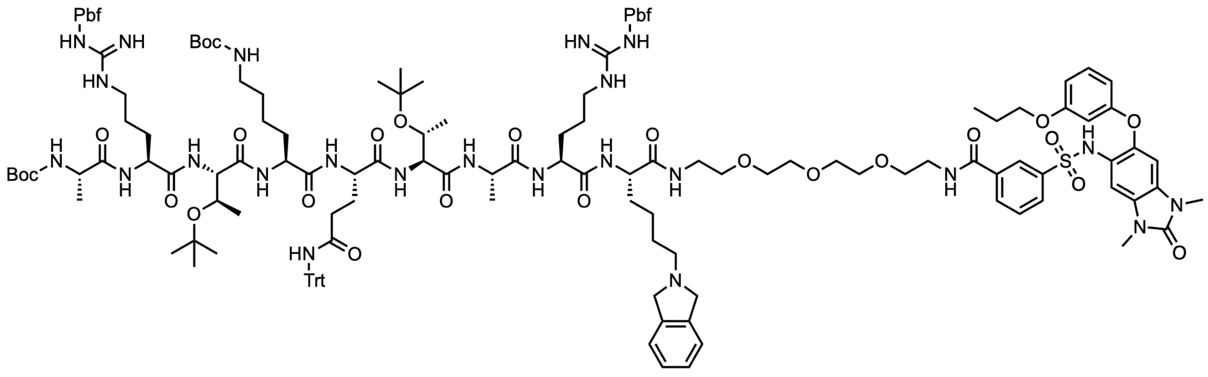

Compound **44** was reacted according to the general Fmoc deprotection procedure to yield the purified amine (diformate salt, 11.3 mg, 11.2 µmol, 1.0 eq.) which was reacted with **46** (23.3 mg, 11.2 µmol, 1.0 eq.) according to general amide coupling procedure 3. The product was purified by flash silica column chromatography (gradient elution with 2–6% (1% NH_4OH in MeOH)/CH_2Cl_2 then gradient elution on a Biotage® system with 2–20% MeOH/CH_2Cl_2) to yield the title compound as a colourless oil (18.3 mg, 57%): R_f 0.41 (1% NH_4OH in MeOH/CH_2Cl_2, 1:9); LRMS (ESI$^+$) *m/z* 1444 ([M+2H]$^{2+}$, 100%), 1455 ([M+H+Na]$^{2+}$, 34%); HRMS (ESI$^+$) *m/z* [Found: 2887.4678, $C_{147}H_{208}N_{23}O_{31}S_3$ requires [M+H]$^+$ 2887.4563]; HPLC retention time: 12.2 min, 97.3% (220 nm), 95.7% (254 nm), 95.4% (280 nm).

(49) *tert*-Butyl ((19*S*,22*S*,25*S*,28*S*,31*S*,34*S*)-28-((*R*)-1-(*tert*-butoxy)ethyl)-34-((6*S*,9*S*,12*S*)-12-((*R*)-1-(*tert*-butoxy)ethyl)-2,2,6-trimethyl-4,7,10-trioxo-9-(3-(3-((2,2,4,6,7-pentamethyl-2,3-dihydrobenzofuran-5-yl)sulfonyl)guanidino)propyl)-3-oxa-5,8,11-triazatridecan-13-amido)-1-(3-(*N*-(1,3-dimethyl-2-oxo-6-(3-propoxyphenoxy)-2,3-dihydro-1*H*-benzo[*d*]imidazol-5-yl)sulfamoyl)phenyl)-19-(4-(isoindolin-2-yl)butyl)-25-methyl-1,18,21,24,27,30,33-heptaoxo-31-(3-oxo-3-(tritylamino)propyl)-22-(3-(3-((2,2,4,6,7-pentamethyl-2,3-dihydrobenzofuran-5-yl)sulfonyl)guanidino)propyl)-5,8,11,14-tetraoxa-2,17,20,23,26,29,32-heptaazaoctatriacontan-38-yl)carbamate (PDC3)

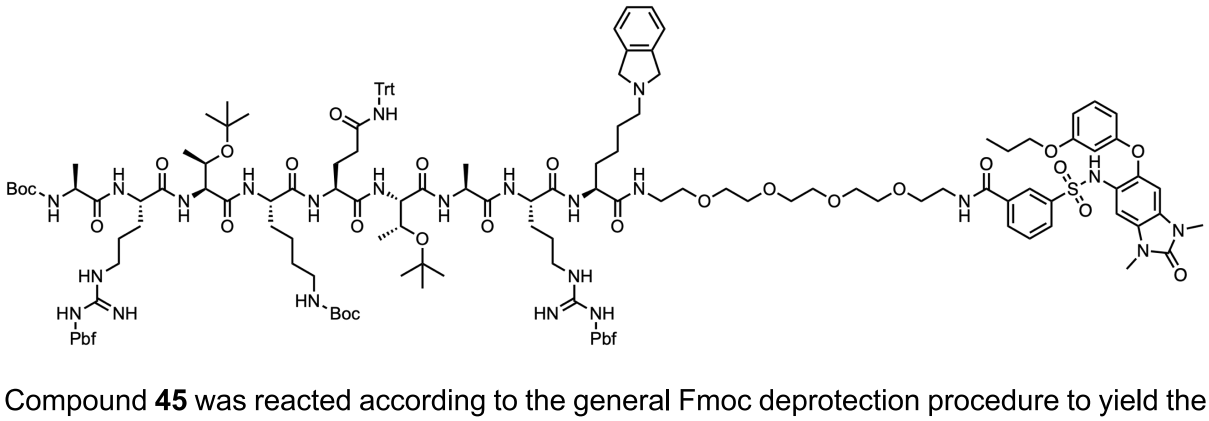

Compound **45** was reacted according to the general Fmoc deprotection procedure to yield the purified amine (diformate salt, 20.8 mg, 19.8 µmol, 1.0 eq.) which was reacted with **46**

(41.2 mg, 19.8 µmol, 1.0 eq.) according to general amide coupling procedure 3. The product was purified by flash silica column chromatography (gradient elution with 2–7% (1% NH_4OH in MeOH)/CH_2Cl_2 then gradient elution on a Biotage® system with 2–20% MeOH/CH_2Cl_2) to yield the title compound as a colourless oil (24.4 mg, 42%): R_f 0.41 (1% NH_4OH in MeOH/CH_2Cl_2, 1:9); LRMS (ESI$^+$) *m/z* 1466 ([M+2H]$^{2+}$, 22%), 1477 ([M+H+Na]$^{2+}$, 100%); HRMS (ESI$^+$) *m/z* [Found: 2931.4868, $C_{149}H_{212}N_{23}O_{32}S_3$ requires [M+H]$^+$ 2931.4826]; HPLC retention time: 12.1 min, 95.2% (220 nm), 95.3% (254 nm), 95.0% (280 nm).

(50) (*S*)-2-((*S*)-6-Amino-2-((2*S*,3*R*)-2-((*S*)-2-((*S*)-2-aminopropanamido)-5-guanidinopentanamido)-3-hydroxybutanamido)hexanamido)-*N*¹-((13*S*,16*S*,19*S*,22*S*,23*R*)-1-(3-(*N*-(1,3-dimethyl-2-oxo-6-(3-propoxyphenoxy)-2,3-dihydro-1*H*-benzo[*d*]imidazol-5-yl)sulfamoyl)phenyl)-16-(3-guanidinopropyl)-23-hydroxy-13-(4-(isoindolin-2-yl)butyl)-19-methyl-1,12,15,18,21-pentaoxo-5,8-dioxa-2,11,14,17,20-pentaazatetracosan-22-yl)pentanediamide pentatrifluoroacetic acid (PDC4)

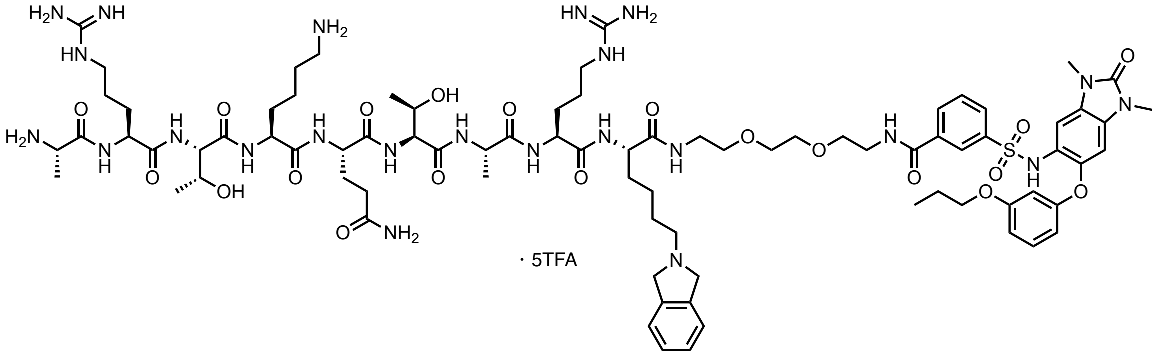

47 (22.5 mg, 7.91 µmol, 1.0 eq.) was reacted according to the global deprotection procedure. The product was purified by semi-preparative reverse phase HPLC (gradient method 3, 20 mL min^{-1}, Agilent Zorbax PrepHT column [5 µm, 21.2 mm × 50 mm]) and lyophilised to yield the title compound as a colourless solid (5.9 mg, 32%): LC-MS (ESI$^+$) retention time = 5.0 min, *m/z* = 358.2 ([M–5TFA+5H]$^{5+}$), 447.4 ([M–5TFA+4H]$^{4+}$), 596.0 ([M–5TFA+3H]$^{3+}$), 893.3 ([M–5TFA+2H]$^{2+}$); HRMS (ESI$^+$) *m/z* [Found: 1784.9306, $C_{82}H_{126}N_{23}O_{20}S$ requires [M–5TFA+H]$^+$ 1784.9265]; HPLC retention time: 5.8 min, 95.5% (220 nm), 95.2% (254 nm), 95.2% (280 nm).

(51) (*S*)-2-((*S*)-6-Amino-2-((2*S*,3*R*)-2-((*S*)-2-((*S*)-2-aminopropanamido)-5-guanidinopentanamido)-3-hydroxybutanamido)hexanamido)-*N*1-((16*S*,19*S*,22*S*,25*S*,26*R*)-1-(3-(*N*-(1,3-dimethyl-2-oxo-6-(3-propoxyphenoxy)-2,3-dihydro-1*H*-benzo[*d*]imidazol-5-yl)sulfamoyl)phenyl)-19-(3-guanidinopropyl)-26-hydroxy-16-(4-(isoindolin-2-yl)butyl)-22-methyl-1,15,18,21,24-pentaoxo-5,8,11-trioxa-2,14,17,20,23-pentaazaheptacosan-25-yl)pentanediamide pentatrifluoroacetic acid (PDC5)

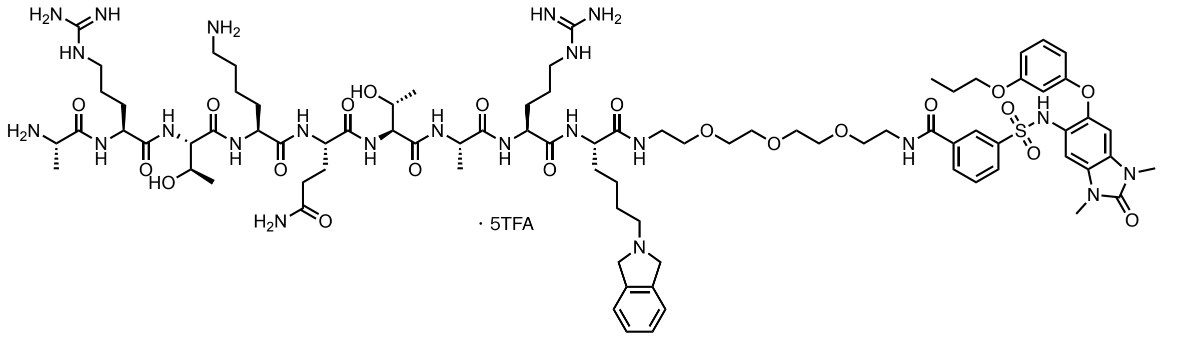

48 (18.3 mg, 6.34 µmol, 1.0 eq.) was reacted according to the global deprotection procedure. The product was purified by semi-preparative reverse phase HPLC (gradient method 3, 20 mL min^{-1}, Agilent Zorbax PrepHT column [5 µm, 21.2 mm × 50 mm]) and lyophilised to yield the title compound as a colourless solid (7.8 mg, 51%): LC-MS (ESI$^+$) retention time = 5.0 min, *m/z* = 366.9 ([M–5TFA+5H]$^{5+}$), 458.4 ([M–5TFA+4H]$^{4+}$), 610.7 ([M–5TFA+3H]$^{3+}$), 915.4 ([M–5TFA+2H]$^{2+}$); HRMS (ESI$^+$) *m/z* [Found: 1828.9561, $C_{84}H_{130}N_{23}O_{21}S$ requires [M–5TFA+H]$^+$ 1828.9527]; HPLC retention time: 5.7 min, 97.3% (220 nm), 97.1% (254 nm), 96.2% (280 nm).

(52) (*S*)-2-((*S*)-6-Amino-2-((2*S*,3*R*)-2-((*S*)-2-((*S*)-2-aminopropanamido)-5-guanidinopentanamido)-3-hydroxybutanamido)hexanamido)-*N*1-((19*S*,22*S*,25*S*,28*S*,29*R*)-1-(3-(*N*-(1,3-dimethyl-2-oxo-6-(3-propoxyphenoxy)-2,3-dihydro-1*H*-benzo[*d*]imidazol-5-yl)sulfamoyl)phenyl)-22-(3-guanidinopropyl)-29-hydroxy-19-(4-(isoindolin-2-yl)butyl)-25-methyl-1,18,21,24,27-pentaoxo-5,8,11,14-tetraoxa-2,17,20,23,26-pentaazatriacontan-28-yl)pentanediamide pentatrifluoroacetic acid (PDC6)

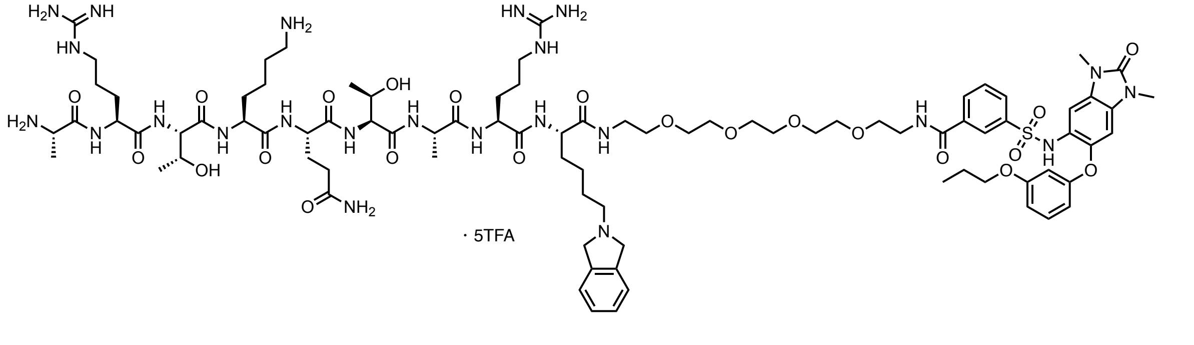

Compound **49** (18.8 mg, 6.41 µmol, 1.0 eq.) was reacted according to the global deprotection procedure. The product was purified by semi-preparative reverse phase HPLC (gradient method 3, 20 mL min^{-1}, Agilent Zorbax PrepHT column [5 µm, 21.2 mm × 50 mm]) and lyophilised to yield the title compound as a colourless solid (7.8 mg, 50%): LC-MS (ESI$^+$) retention time = 5.2 min, *m/z* = 375.7 ([M–5TFA+5H]$^{5+}$), 469.4 ([M–5TFA+4H]$^{4+}$), 625.3 ([M–5TFA+3H]$^{3+}$), 937.3 ([M–5TFA+2H]$^{2+}$); HRMS (ESI$^+$) *m/z* [Found: 1872.9797, $C_{86}H_{134}N_{23}O_{22}S$ requires [M–5TFA+H]$^+$ 1872.9789]; HPLC retention time: 5.9 min, 97.7% (220 nm), 97.6% (254 nm), 97.3% (280 nm).

(53) Benzyl (*S*)-(1-(methylamino)-1-oxopent-4-en-2-yl)carbamate

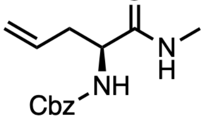

Methyl amine (2 M in THF, 5 mL, 10.0 mmol, 1.0 eq.) was added to a solution of (*S*)-2-(((benzyloxy)carbonyl)amino)pent-4-enoic acid (2.5 g, 10.0 mmol, 1.0 eq.) and DIPEA (3.48 mL, 20.0 mmol, 2.0 eq.) in anhydrous DMF (30 mL) at rt. HATU (3.80 g, 10.0 mmol, 1.0 eq.) was added and the reaction solution was stirred at rt for 2 h. The reaction mixture was concentrated *in vacuo* and the residue was dissolved with EtOAc (50 mL). The organic component was washed with aq. LiCl (0.5 M, 50 mL), aq. citric acid (10% *w/v*, 50 mL), sat. aq. $NaHCO_3$ (50 mL), brine (50 mL), dried (Na_2SO_4), filtered, and concentrated *in vacuo*. The product was purified by flash silica column chromatography (isocratic elution with 50% EtOAc/petroleum ether 40–60 °C) to yield the title compound as a white solid (2.47 g, 94%): R_f 0.20 (EtOAc/petroleum ether 40–60 °C, 1:1); m.p. 115–118 °C (from EtOAc); $[\alpha]_D^{25}$ = +3.6 (*c*=1.0 in MeOH); ^{1}H NMR (600 MHz, CD_3OD) δ_H 7.39–7.26 (m, 5H), 5.81–5.70 (m, 1H), 5.18–5.02 (m, 4H), 4.15–4.07 (m, 1H), 2.72 (s, 3H), 2.56–2.46 (m, 1H), 2.42–2.30 (m, 1H); ^{13}C NMR (151 MHz, CD_3OD) δ_C 174.7, 158.3, 138.1, 134.7, 129.5, 129.0, 128.9, 118.6, 67.7, 56.2, 37.6, 26.3; $\overline{\nu}_{max}$ (thin film)/cm^{-1} 3297 (m), 1693 (s), 1650 (s), 1261 (s), 862 (s); LRMS (ESI$^+$) *m/z* 263 ([M+H]$^+$, 100%); HRMS (ESI$^+$) *m/z* [Found: 285.1212, $C_{14}H_{18}N_2O_3Na$ requires [M+Na]$^+$ 285.1210]; HPLC retention time: 7.8 min, 97.4% (220 nm), 98.6% (250 nm), 95.8% (254 nm).

(54) Benzyl (*S*,*E*)-(6-(isoindolin-2-yl)-1-(methylamino)-1-oxohex-4-en-2-yl)carbamate hydrochloride

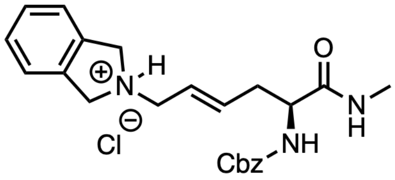

cis-Butene-1,4-diol (1.27 mL, 15.4 mmol, 3.00 eq.) and Grubbs 2nd generation catalyst (219 mg, 0.26 mmol, 0.05 eq.) were added to a solution of **53** (1.35 g, 5.15 mmol, 1.00 eq.) in anhydrous, degassed CH_2Cl_2 (50 mL) under an argon atmosphere at rt. The reaction solution was stirred at rt for 6 h. The reaction solution was filtered through Celite® and the filtrate was concentrated *in vacuo*. The product was purified by flash silica column chromatography (gradient elution with 2–5% MeOH/CH_2Cl_2) and then by semi-preparative reverse phase HPLC (gradient method 1, 40 mL min^{-1}, Agilent Zorbax PrepHT column [7 μm, 21.2 mm × 250 mm]) to yield the crude metathesis product as a colourless oil (988 mg crude yield, 66%): R_f 0.38 (MeOH:CH_2Cl_2, 1:9); LRMS (ESI$^+$): *m/z* 315 ($[M+Na]^+$, 100%). The product was taken forward crude to next step due to the presence of an impurity that was inseparable by both normal and reverse phase column chromatography.

Methanesuflonyl chloride (392 μL, 5.07 mmol, 1.5 eq.) was added dropwise to a solution of the crude metathesis product (988 mg, 9.56 mmol, 1.0 eq.) and NEt_3 (944 μL, 6.77 mmol, 2.0 eq.) in CH_2Cl_2 (20 mL) at 0 °C. The reaction mixture was warmed to rt and stirred for 1 h. The reaction mixture was diluted with $CHCl_3$ (20 mL) and washed with sat. aq. $NaHCO_3$ (50 mL), and brine (50 mL). The organic component was dried (Na_2SO_4), filtered, and concentrated *in vacuo* to yield the crude mesylate which was reacted directly in the next step. Isoindoline (755 μL, 6.77 mmol, 2.0 eq.) was added dropwise to a solution of the crude mesylate in anhydrous DMF (20 mL) and the reaction solution was stirred at rt for 1 h. The reaction solution was diluted with aq. LiCl (0.5 M, 50 mL) and the aqueous phase was extracted with EtOAc (3 × 50 mL). The organic components were combined, washed with brine (50 mL), dried (Na_2SO_4), filtered, and concentrated *in vacuo*. The product was purified by flash silica column chromatography (1–7% (1% NH_4OH in MeOH)/CH_2Cl_2). The purified product was dissolved in Et_2O (25 mL) and washed with aq. HCl (6 M, 3 × 25 mL). The aqueous components were combined and concentrated *in vacuo* to yield the title compound as a dark green solid (797 mg, 36% over 3 steps): R_f 0.30 (free base, 1% NH_4OH in MeOH/CH_2Cl_2, 1:9); m.p. 96–98 °C (from CH_2Cl_2); $[\alpha]_D^{25}$ = +12.9 (*c*=0.1 in MeOH); ^{1}H NMR (700 MHz, CD_3OD) δ_H 7.46–7.26 (m, 10H), 6.12–6.00 (dt, *J* 6.7 15.5 Hz, 1H), 5.74 (dt, *J* 7.2 15.5 Hz, 1H), 5.12 (d, *J* 12.5 Hz, 1H), 5.07 (d, *J* 12.5 Hz, 1H), 4.79–4.72 (m, 2H), 4.53–4.45 (m, 2H), 4.21 (dd, *J* 5.4 8.6 Hz, 1H), 3.94 (d, *J* 6.7 Hz, 2H), 2.78–2.62 (m, 4H), 2.50–2.41 (m, 1H); ^{13}C NMR (176 MHz, CD_3OD) δ_C 174.1, 158.3, 139.1, 138.1, 134.9, 130.2, 129.6, 129.2, 129.0, 124.1, 123.3, 67.9, 59.0, 57.1, 55.8, 36.2, 26.4; $\overline{\nu}_{max}$ (thin film)/cm^{-1} 3271 (m), 2941 (w), 1713 (s), 1663 (s), 1252 (s), 750 (s); LRMS (ESI$^+$) *m/z* 394 ($[M–HCl+H]^+$, 100%); HRMS (ESI$^+$) *m/z* [Found: 394.2137; $C_{23}H_{28}N_3O_3$ requires $[M–HCl+H]^+$ 394.2125]; HPLC retention time: 6.5 min, 98.3% (220 nm), 98.2% (250 nm), 96.1% (254 nm).

(55) *tert*-Butyl ((4*S*,7*S*,10*S*,13*S*,16*S*,19*S*)-13-((*R*)-1-(*tert*-butoxy)ethyl)-19-((6*S*,9*S*,12*S*)-12-((*R*)-1-(*tert*-butoxy)ethyl)-2,2,6-trimethyl-4,7,10-trioxo-9-(3-(3-((2,2,4,6,7-pentamethyl-2,3-dihydrobenzofuran-5-yl)sulfonyl)guanidino)propyl)-3-oxa-5,8,11-triazatridecan-13-amido)-4-(4-(isoindolin-2-yl)butyl)-10-methyl-3,6,9,12,15,18-hexaoxo-16-(3-oxo-3-(tritylamino)propyl)-7-(3-(3-((2,2,4,6,7-pentamethyl-2,3-dihydrobenzofuran-5-yl)sulfonyl)guanidino)propyl)-2,5,8,11,14,17-hexaazatricosan-23-yl)carbamate triformic acid (PHD Peptide 2)

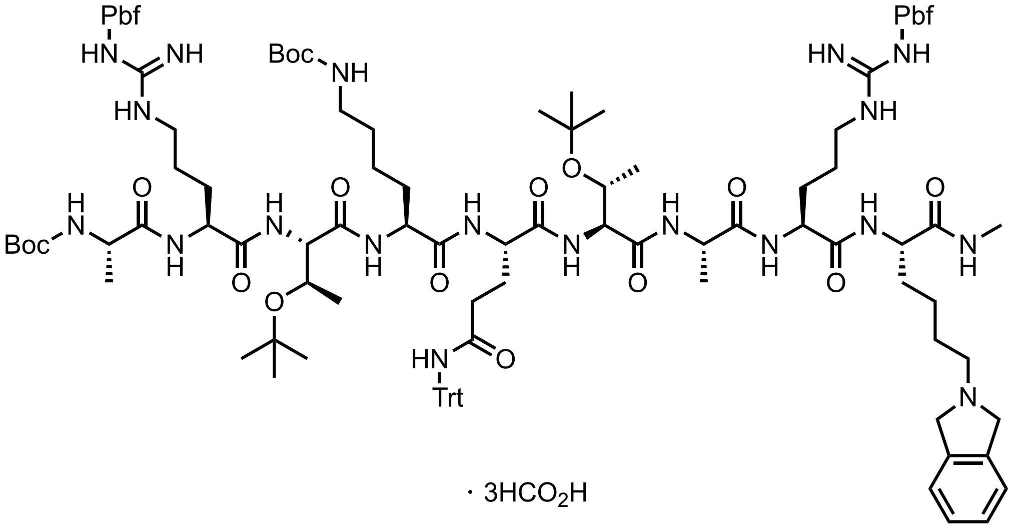

To a solution of **54** (46.0 mg, 107 µmol, 2.0 eq.) in MeOH (10 mL) was added 10% Pd/C (11.4 mg, 10.7 µmol, 0.2 eq.) under an argon atmosphere at rt. The flask was purged with hydrogen gas *via* 3 successive evacuate/fill cycles and the reaction mixture was stirred vigorously under a hydrogen atmosphere at rt for 6 h. The reaction mixture was filtered through Celite® and the filtrate was concentrated *in vacuo* to yield the crude amine which was used directly in the next step without further purification. HATU (20.3 mg, 53.3 µmol, 1.0 eq.) was added to a solution of the crude amine, **46** (111 mg, 53.3 µmol, 1.0 eq.), and DIPEA (46.5 µL, 267 µmol, 5.0 eq.) in anhydrous CH_2Cl_2 (5 mL) at rt. The reaction solution was stirred at rt for 6 h. The reaction solution was concentrated *in vacuo*. The product was purified by semi-preparative reverse phase HPLC (gradient method 4, 20 mL min^{-1}, Agilent Zorbax PrepHT column [5 µm, 21.2 mm × 50 mm]) and lyophilised to yield the title compound as a colourless solid (35.1 mg, 28% over 2 steps): LRMS (ESI$^+$) *m/z* 1117 ([M–3FA+2H]$^{2+}$, 100%) , 1128 ([M–3FA+H+Na]$^{2+}$, 66%); HRMS (ESI$^+$) *m/z* [Found: 2233.2301, $C_{115}H_{170}N_{19}O_{22}S_2$ requires [M–3FA+H]$^+$ 2233.2204], [Found: 2255.2088, $C_{115}H_{169}N_{19}O_{22}S_2Na$ requires [M–3FA+Na]$^+$ 2255.2023]; HPLC retention time: 11.5 min, 97.3% (220 nm), 97.9% (250 nm), 97.7% (254 nm).

(56) (*S*)-2-((*S*)-6-amino-2-((2*S*,3*R*)-2-((*S*)-2-((*S*)-2-aminopropanamido)-5-guanidinopentanamido)-3-hydroxybutanamido)hexanamido)-*N*¹-((4*S*,7*S*,10*S*,13*S*,14*R*)-7-(3-guanidinopropyl)-14-hydroxy-4-(4-(isoindolin-2-yl)butyl)-10-methyl-3,6,9,12-tetraoxo-2,5,8,11-tetraazapentadecan-13-yl)pentanediamide pentatrifluoroacetic acid (PHD Peptide 3)

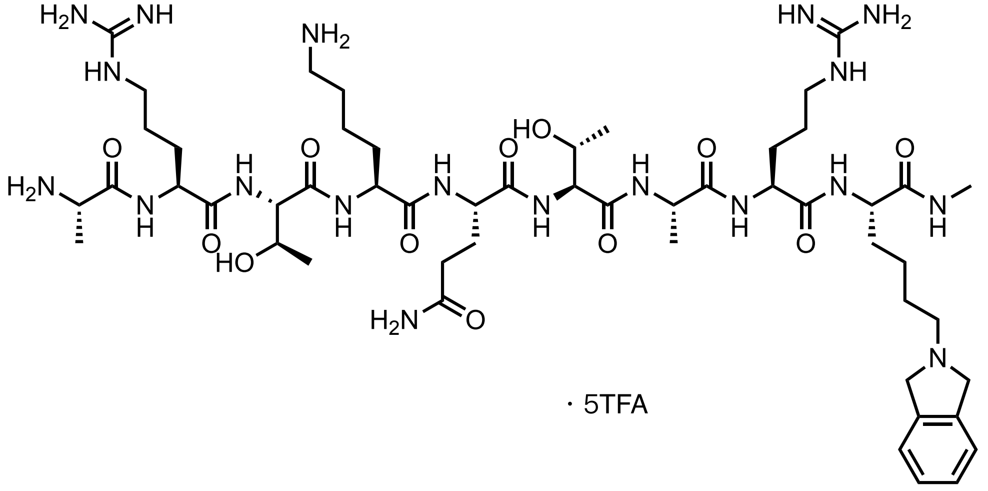

Compound **55** (8.00 mg, 3.37 μmol, 1.0 eq.) was dissolved in a solution of TFA/TIPS/H_2O (38:1:1, 1 mL) and stirred at rt for 4 h. The reaction solution was concentrated *in vacuo* and the residue was redissolved in H_2O (1 mL). The aqueous phase was washed with $CHCl_3$ (3 × 2 mL) and concentrated *in vacuo*. The product was purified by semi-preparative reverse phase HPLC (gradient method 3, 20 mL min^{-1}, Agilent Zorbax PrepHT column [5 μm, 21.2 mm × 50 mm]) and lyophilised to yield the title compound as a colourless solid (4.13 mg, 70%): LC-MS (ESI$^+$) retention time = 0.8 min, *m/z* 294.6 ($[M–5TFA+4H]^{4+}$), 392.5 ($[M–5TFA+3H]^{3+}$), 588.0 ($[M–5TFA+2H]^{2+}$), 1174.8 ($[M–5TFA+H]^+$), 97.6% (220 nm), 98.1% (254 nm), 97.3% (280 nm); HRMS (ESI$^+$) *m/z* [Found: 392.2438, $C_{52}H_{94}O_{12}N_{19}$ requires $[M–5TFA+3H]^{3+}$ 392.2438].

6 NMR Spectra of Novel Compounds

(10) 2-(2-Bromoethyl)isoindoline hydrobromide ^{1}H NMR

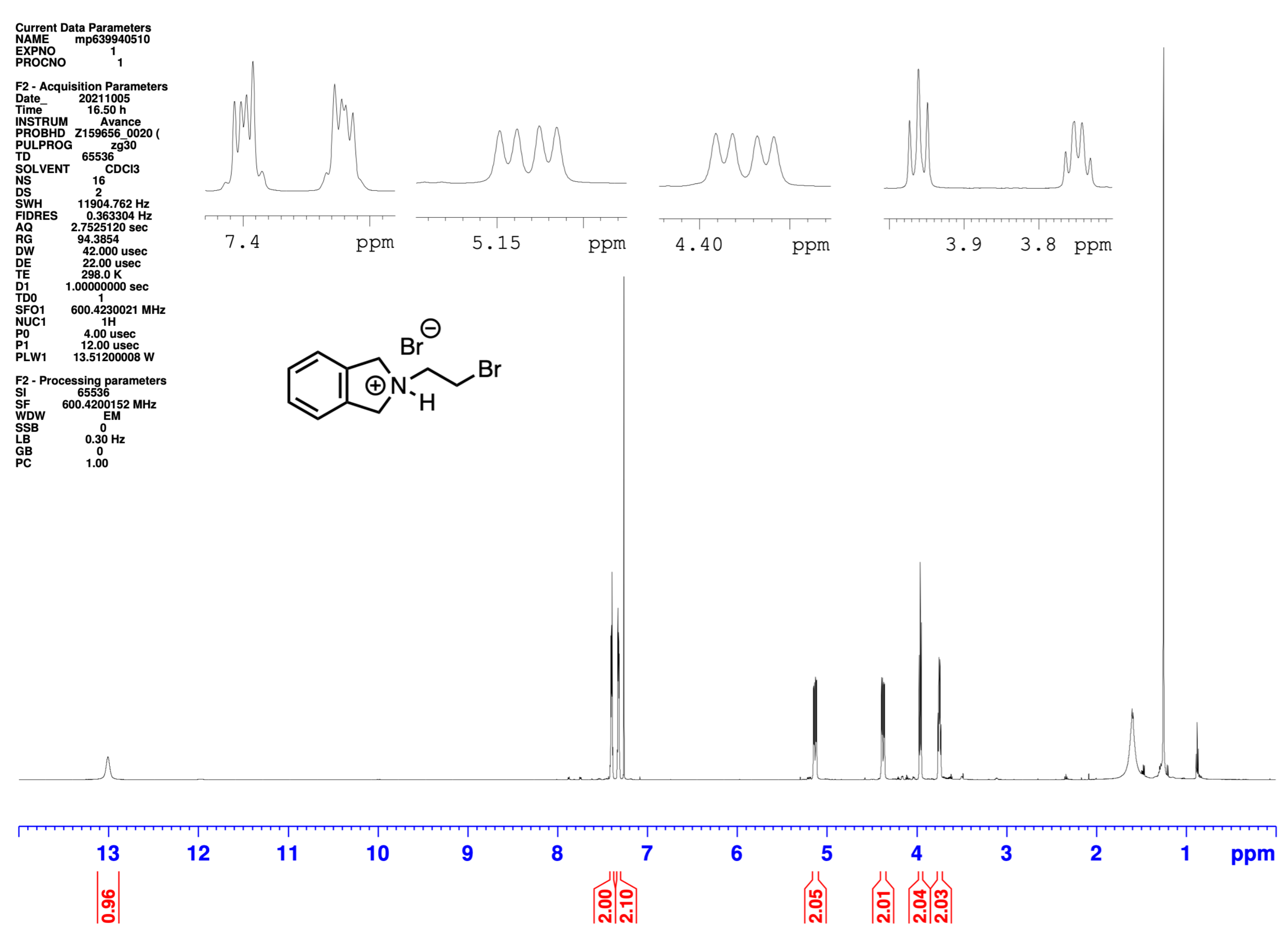

(10) 2-(2-Bromoethyl)isoindoline hydrobromide ^{13}C NMR

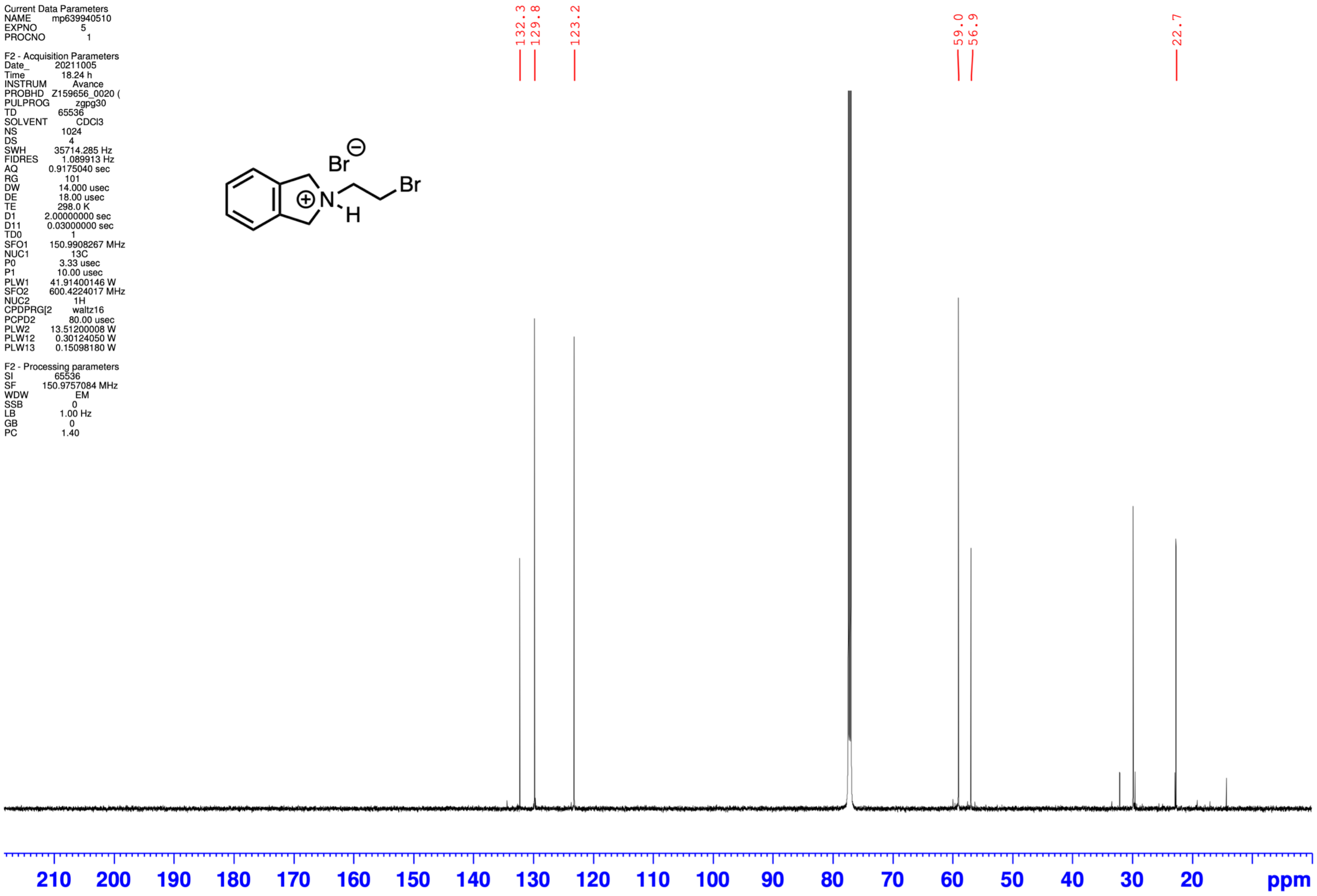

(12) 2-(2-Bromoethyl)-1,2,3,4-tetrahydroisoquinoline hydrobromide ^{1}H NMR

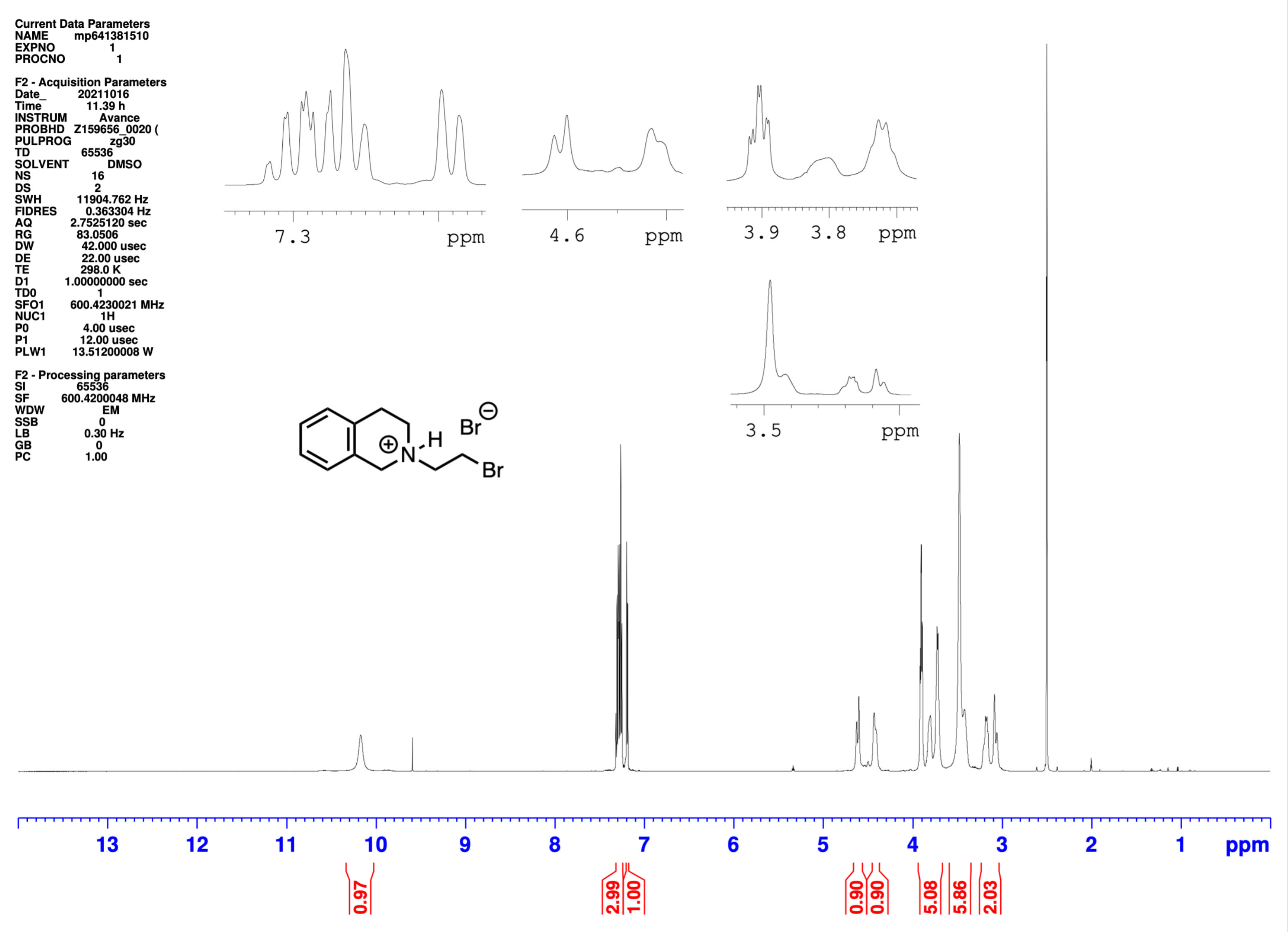

(12) 2-(2-Bromoethyl)-1,2,3,4-tetrahydroisoquinoline hydrobromide ^{13}C NMR

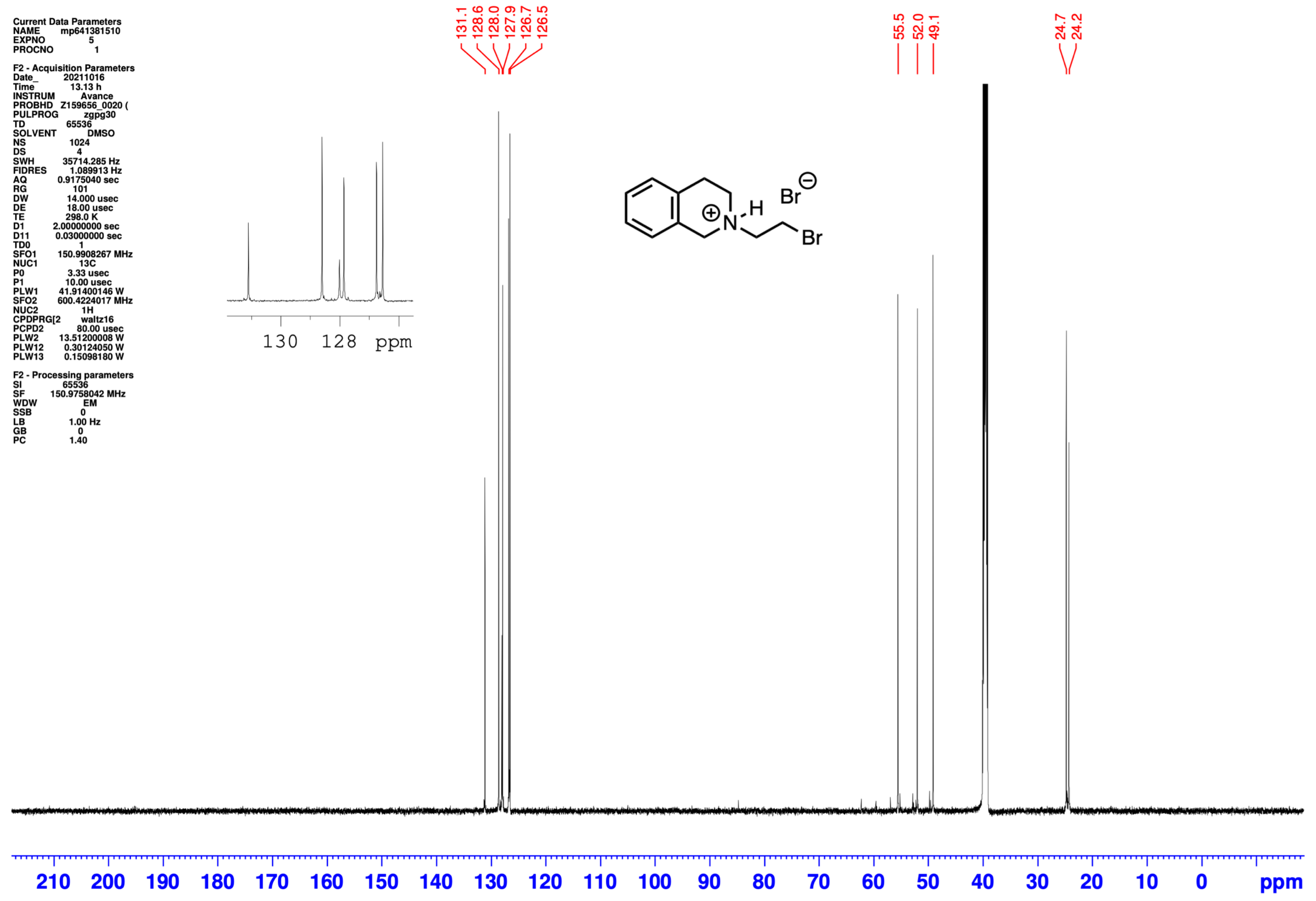

(19) 7a-(Chloromethyl)hexahydro-1*H*-pyrrolizine hydrochloride ^{1}H NMR

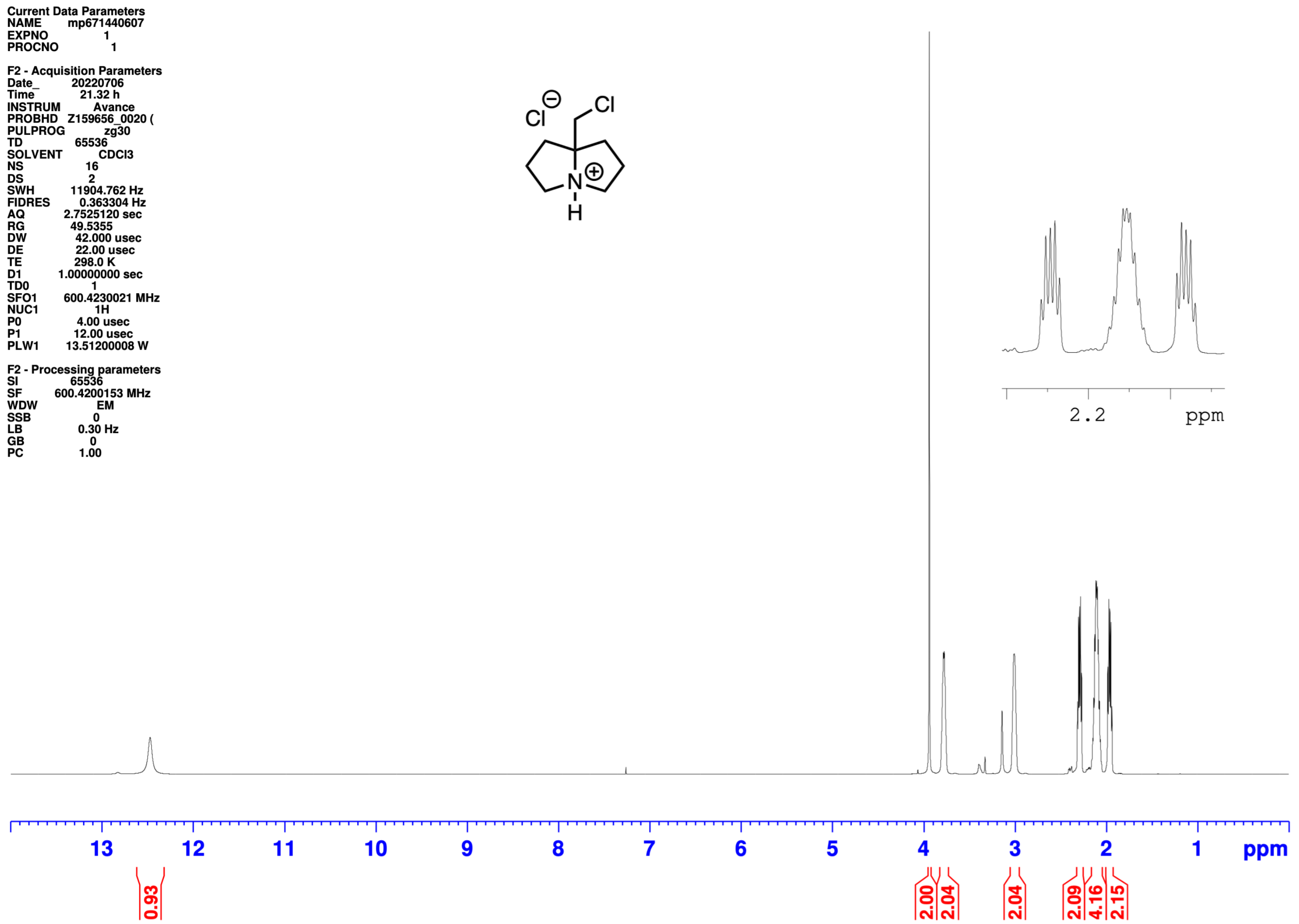

(19) 7a-(Chloromethyl)hexahydro-1*H*-pyrrolizine hydrochloride ^{13}C NMR

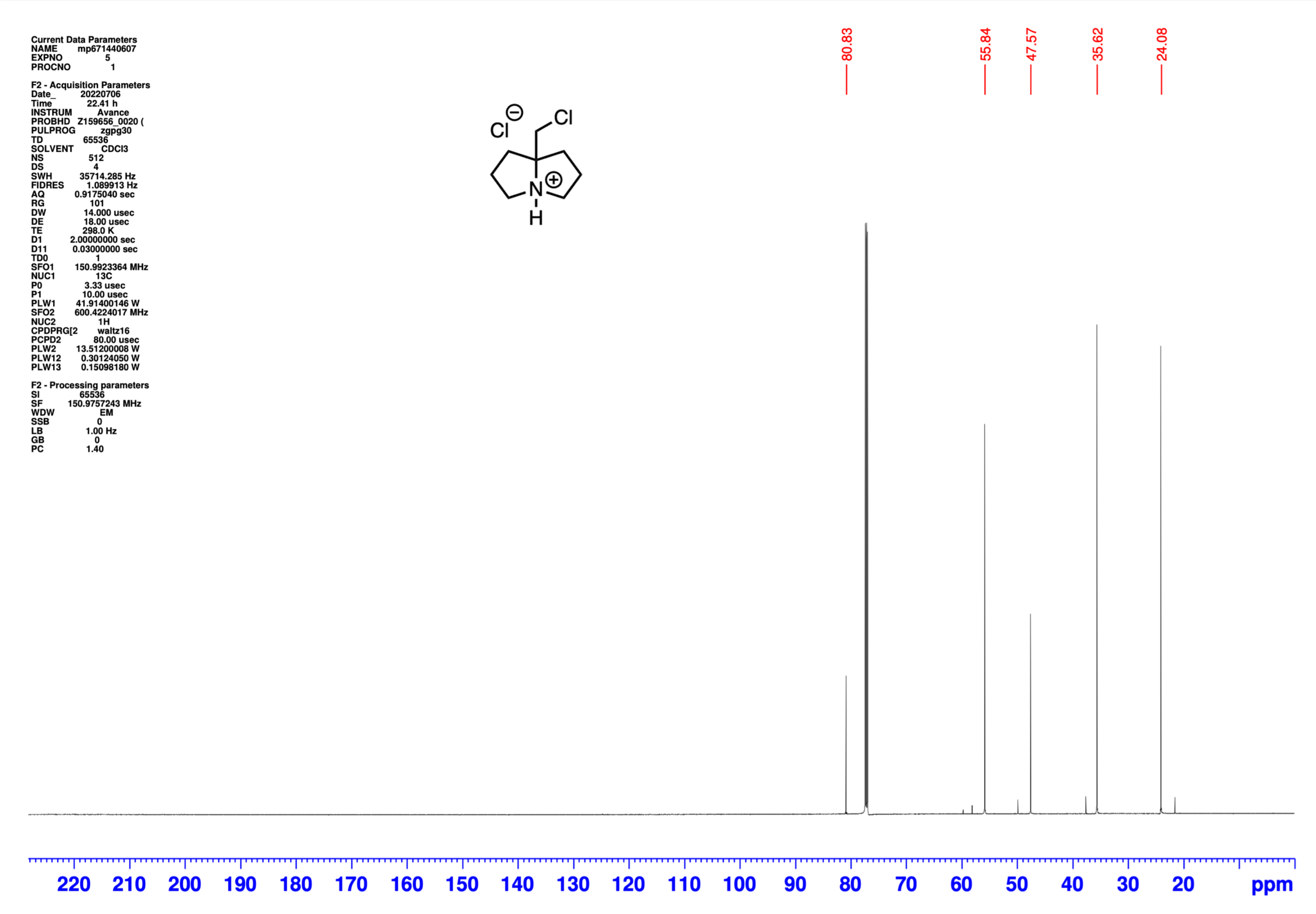

(31) ***tert*-Butyl (1-(3-(*N*-(1,3-dimethyl-2-oxo-6-(3-propoxyphenoxy)-2,3-dihydro-1*H*-benzo[*d*]imidazol-5-yl)sulfamoyl)phenyl)-1-oxo-5,8,11,14-tetraoxa-2-azahexadecan-16-yl)carbamate ^{1}H NMR**

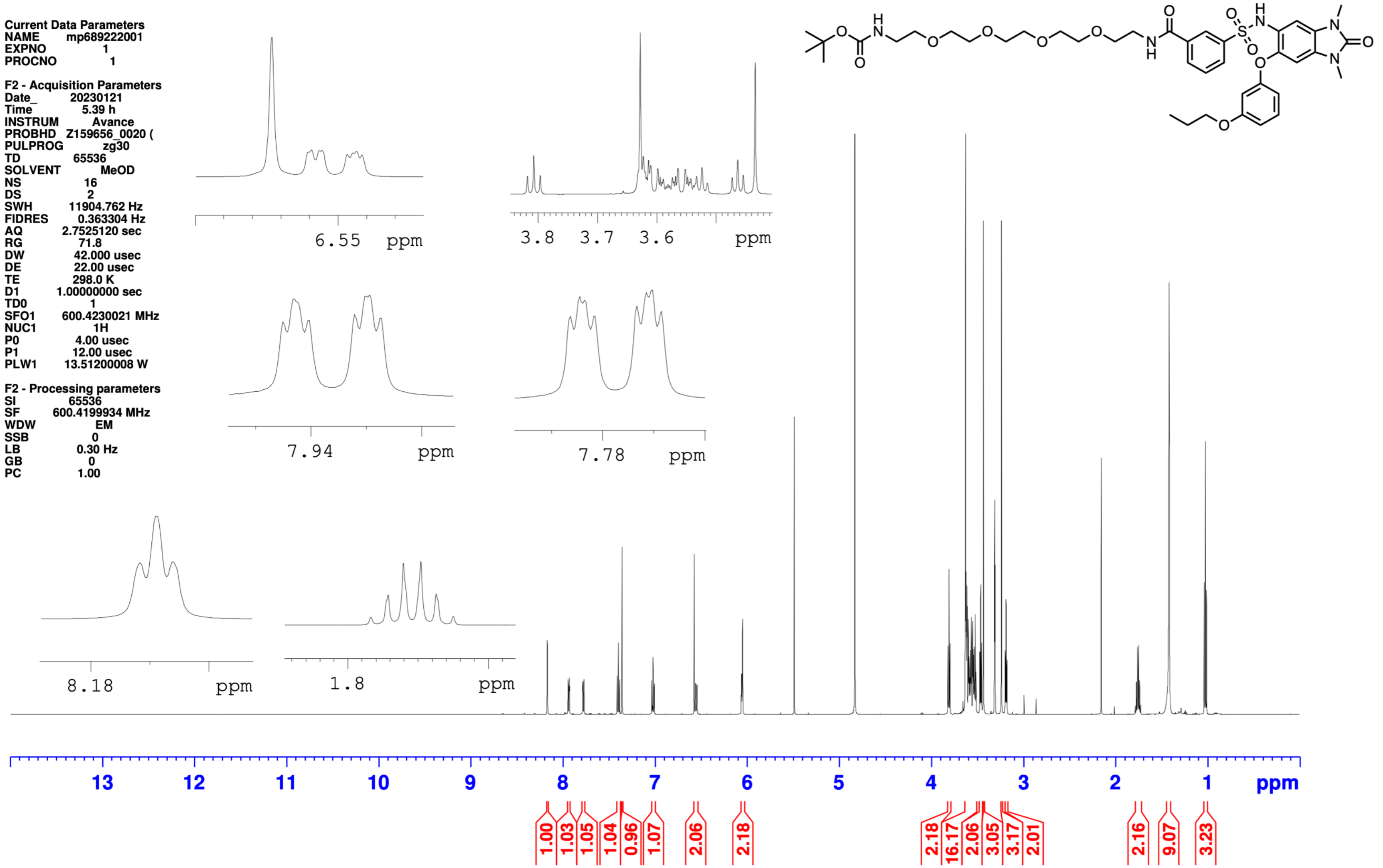

(31) ***tert*-Butyl (1-(3-(*N*-(1,3-dimethyl-2-oxo-6-(3-propoxyphenoxy)-2,3-dihydro-1*H*-benzo[*d*]imidazol-5-yl)sulfamoyl)phenyl)-1-oxo-5,8,11,14-tetraoxa-2-azahexadecan-16-yl)carbamate ^{13}C NMR**

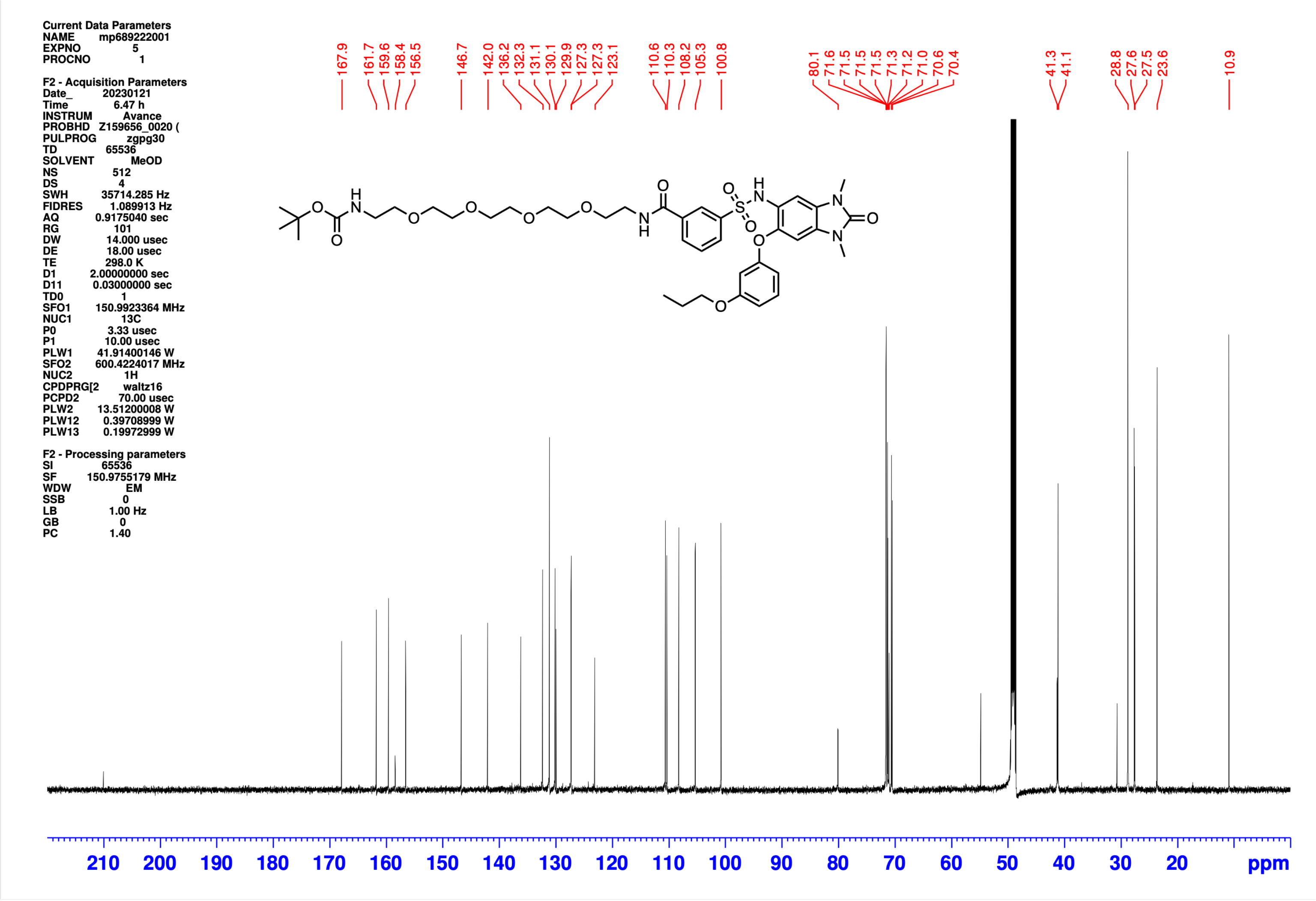

(32) 3-(*N*-(1,3-dimethyl-2-oxo-6-(3-propoxyphenoxy)-2,3-dihydro-1*H*-benzo[*d*]imidazol-5-yl)sulfamoyl)-*N*-(2-oxo-6,9-dioxa-3-azaundecan-11-yl)benzamide ^{1}H NMR

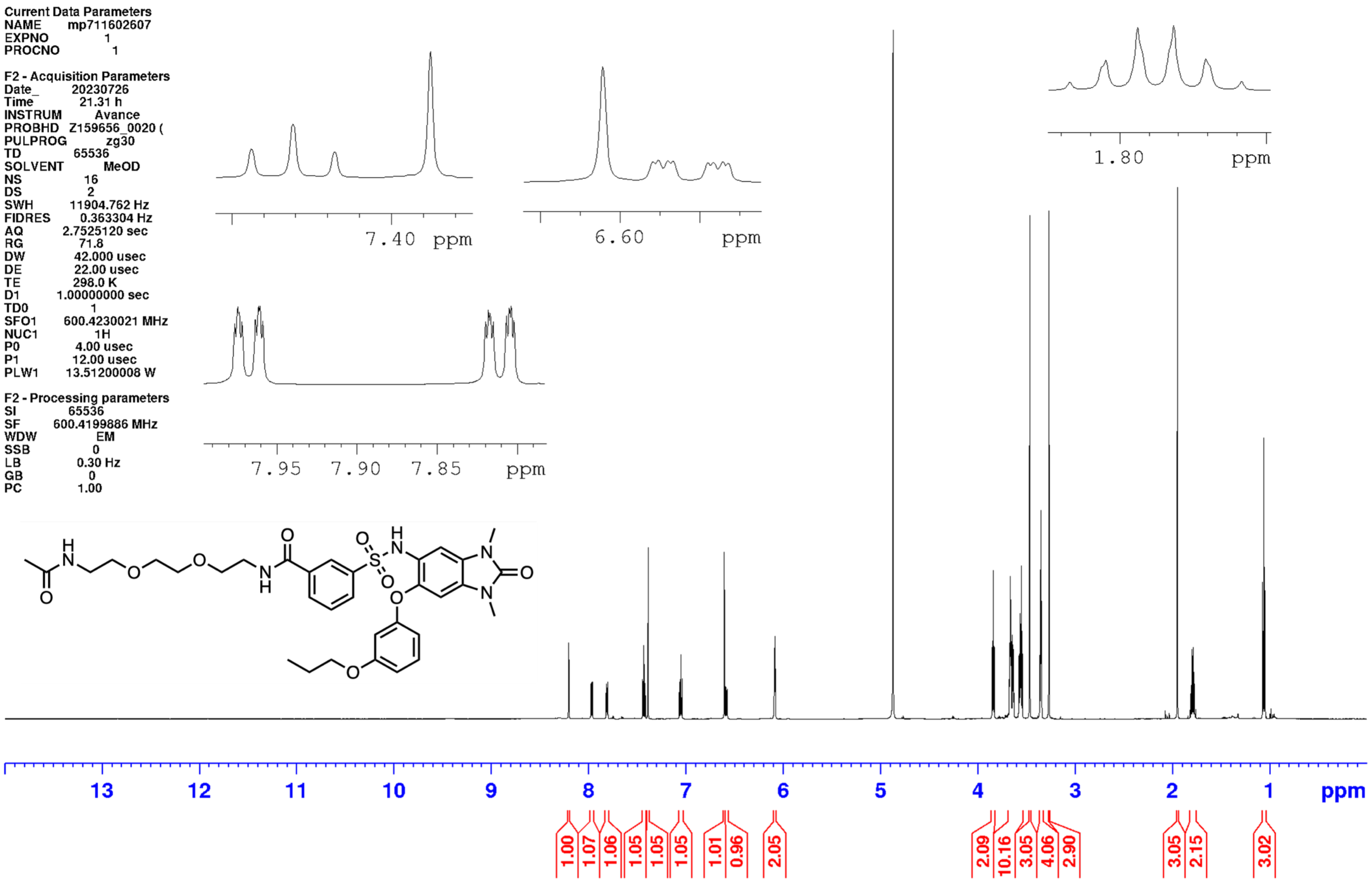

(32) 3-(*N*-(1,3-dimethyl-2-oxo-6-(3-propoxyphenoxy)-2,3-dihydro-1*H*-benzo[*d*]imidazol-5-yl)sulfamoyl)-*N*-(2-oxo-6,9-dioxa-3-azaundecan-11-yl)benzamide ^{13}C NMR

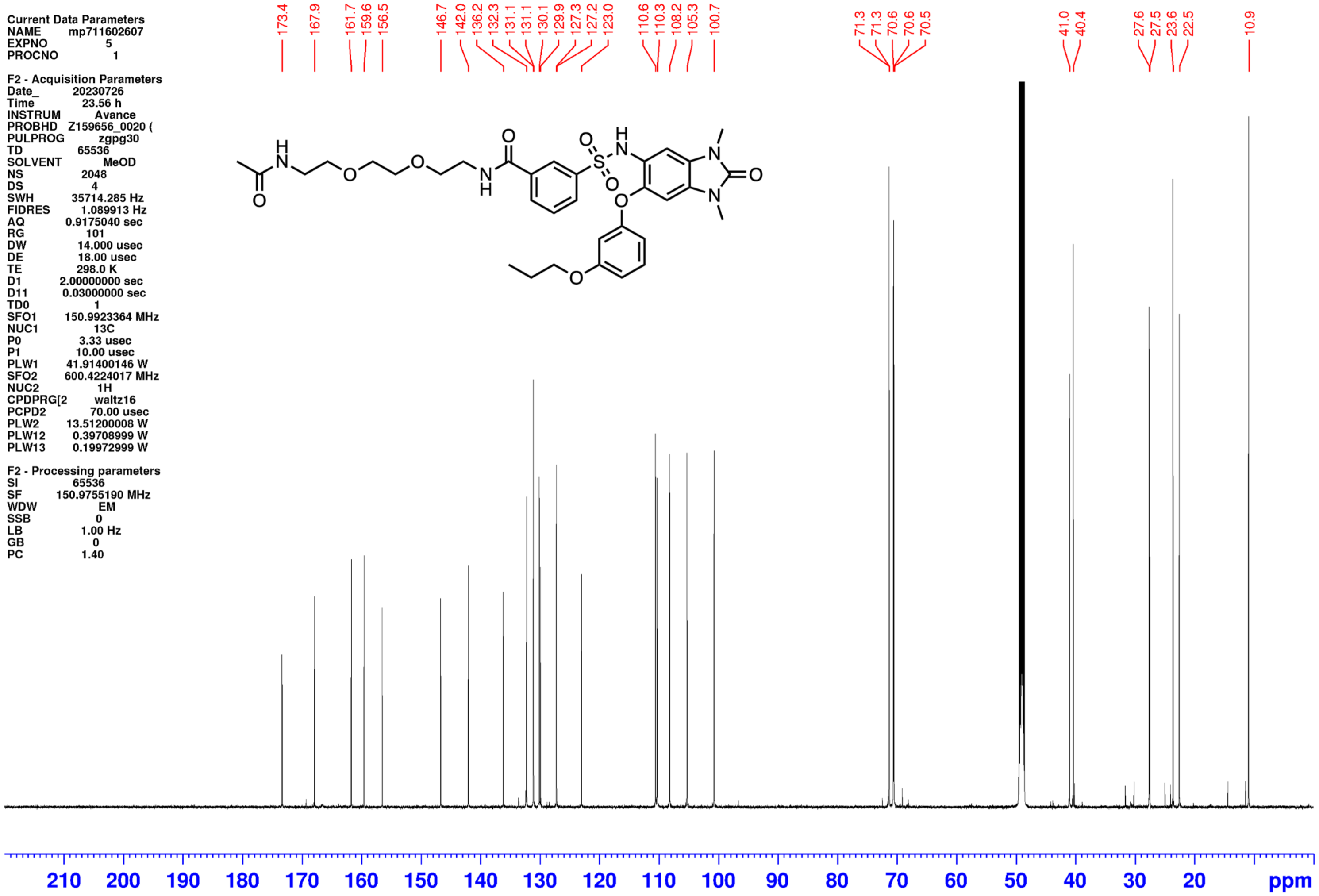

(33) 3-(*N*-(1,3-dimethyl-2-oxo-6-(3-propoxyphenoxy)-2,3-dihydro-1*H*-benzo[*d*]imidazol-5-yl)sulfamoyl)-*N*-(2-oxo-6,9,12-trioxa-3-azatetradecan-14-yl)benzamide ^{1}H NMR

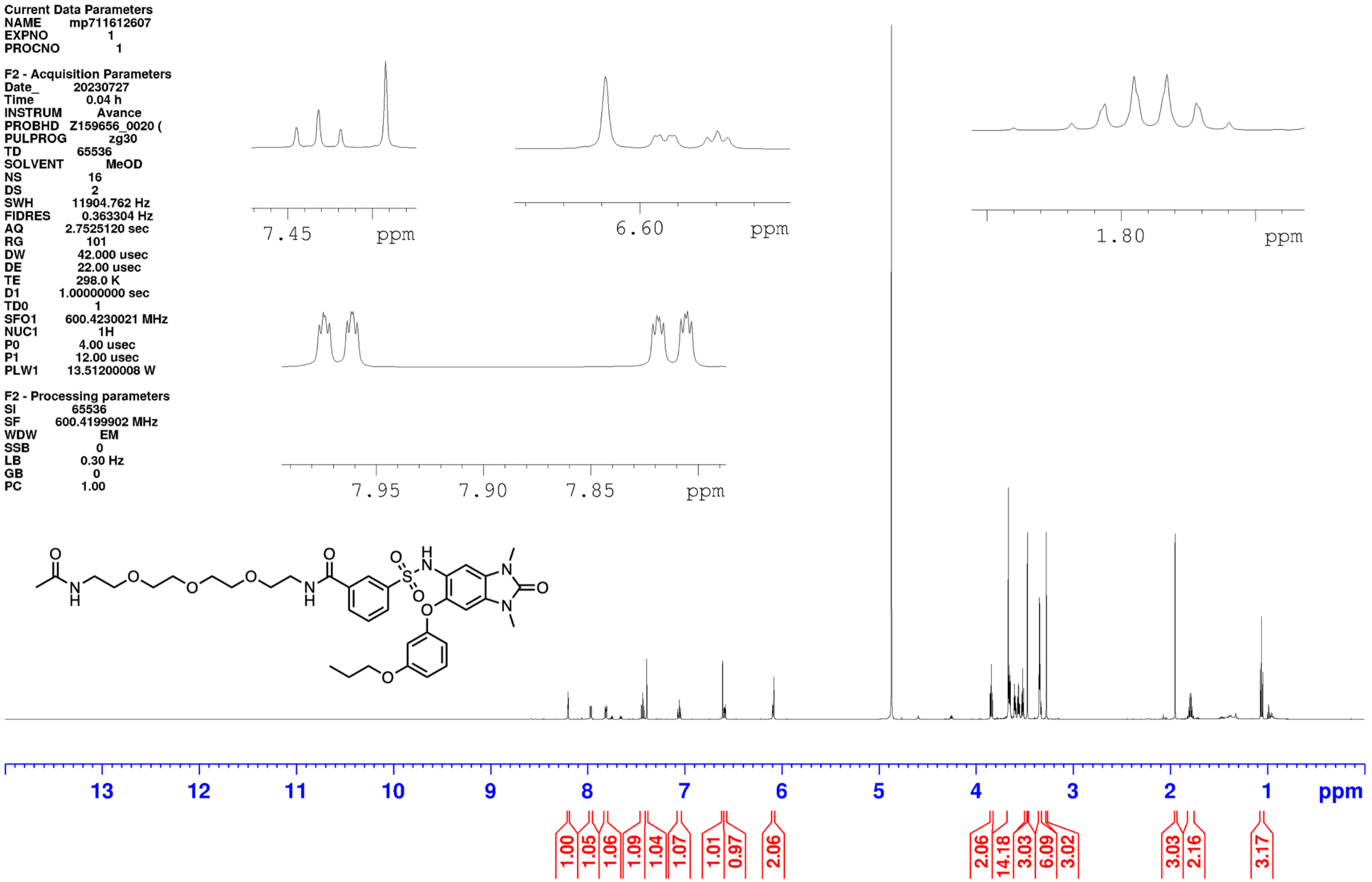

(33) 3-(*N*-(1,3-dimethyl-2-oxo-6-(3-propoxyphenoxy)-2,3-dihydro-1*H*-benzo[*d*]imidazol-5-yl)sulfamoyl)-*N*-(2-oxo-6,9,12-trioxa-3-azatetradecan-14-yl)benzamide ^{13}C NMR

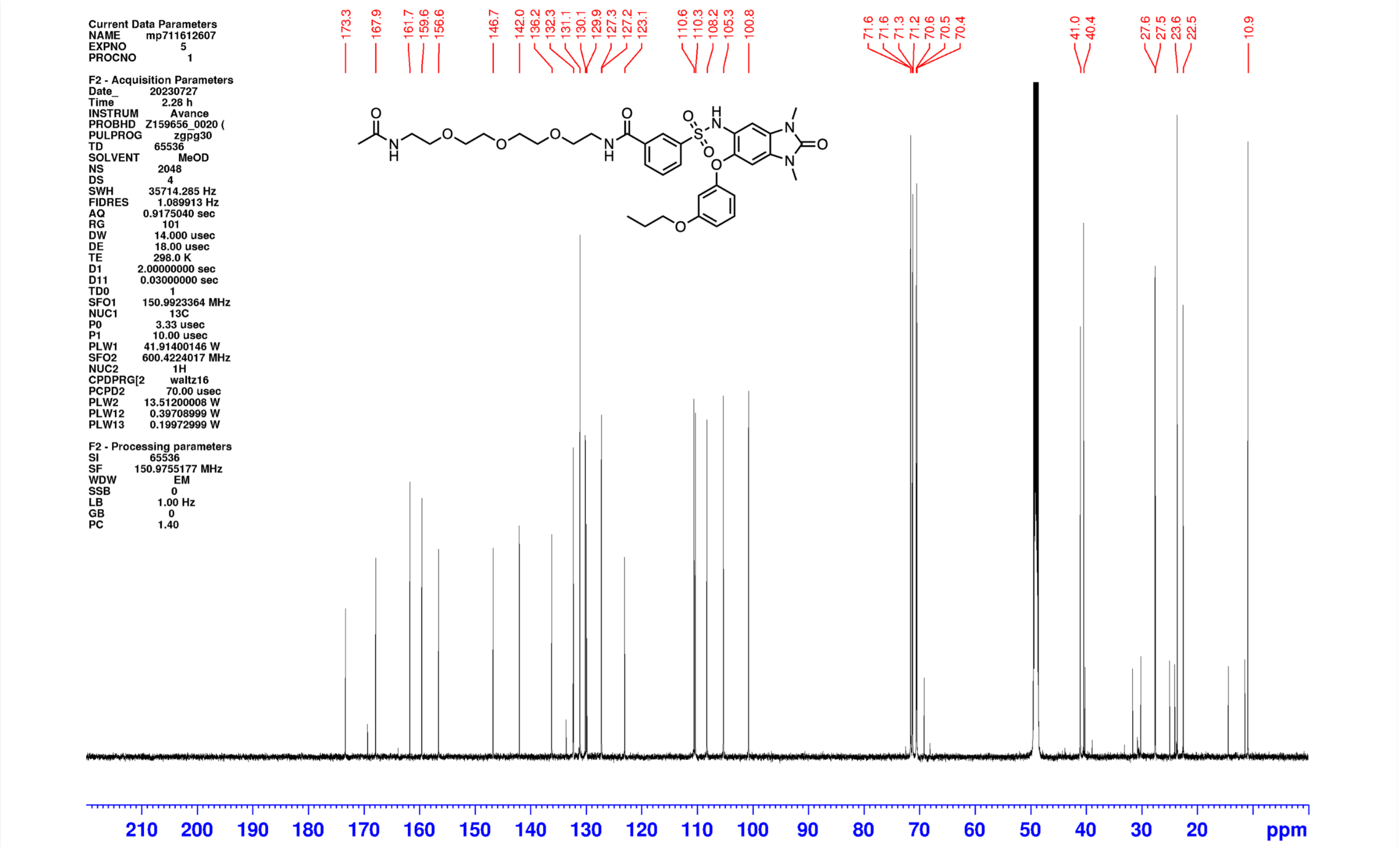

(34) **3-(*N*-(1,3-dimethyl-2-oxo-6-(3-propoxyphenoxy)-2,3-dihydro-1*H*-benzo[*d*]imidazol-5-yl)sulfamoyl)-*N*-(2-oxo-6,9,12,15-t etraoxa-3-azaheptadecan-17-yl)benzamide ^{1}H NMR**

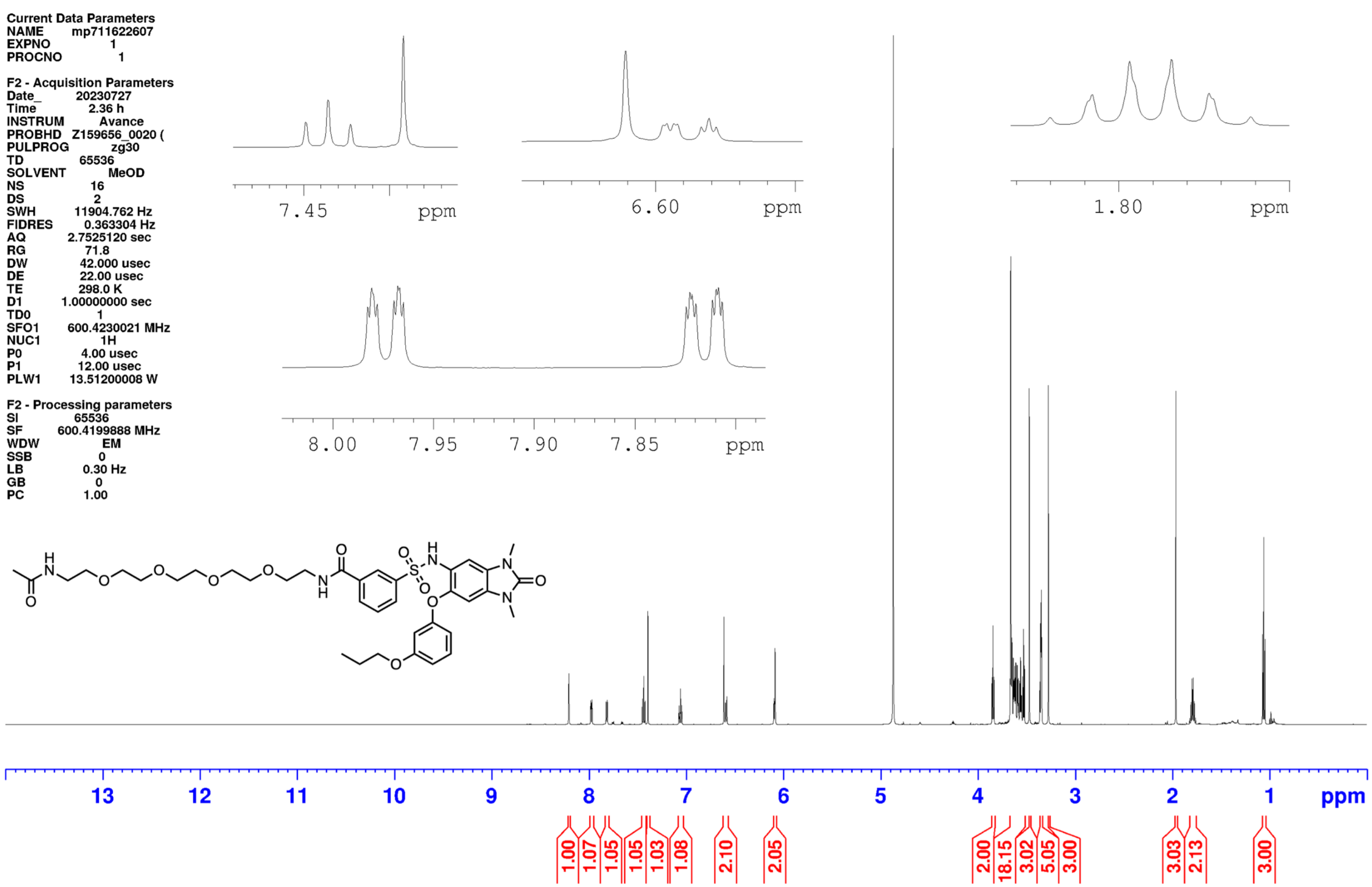

(34) 3-(*N*-(1,3-dimethyl-2-oxo-6-(3-propoxyphenoxy)-2,3-dihydro-1*H*-benzo[*d*]imidazol-5-yl)sulfamoyl)-*N*-(2-oxo-6,9,12,15-tetraoxa-3-azaheptadecan-17-yl)benzamide ^{13}C NMR

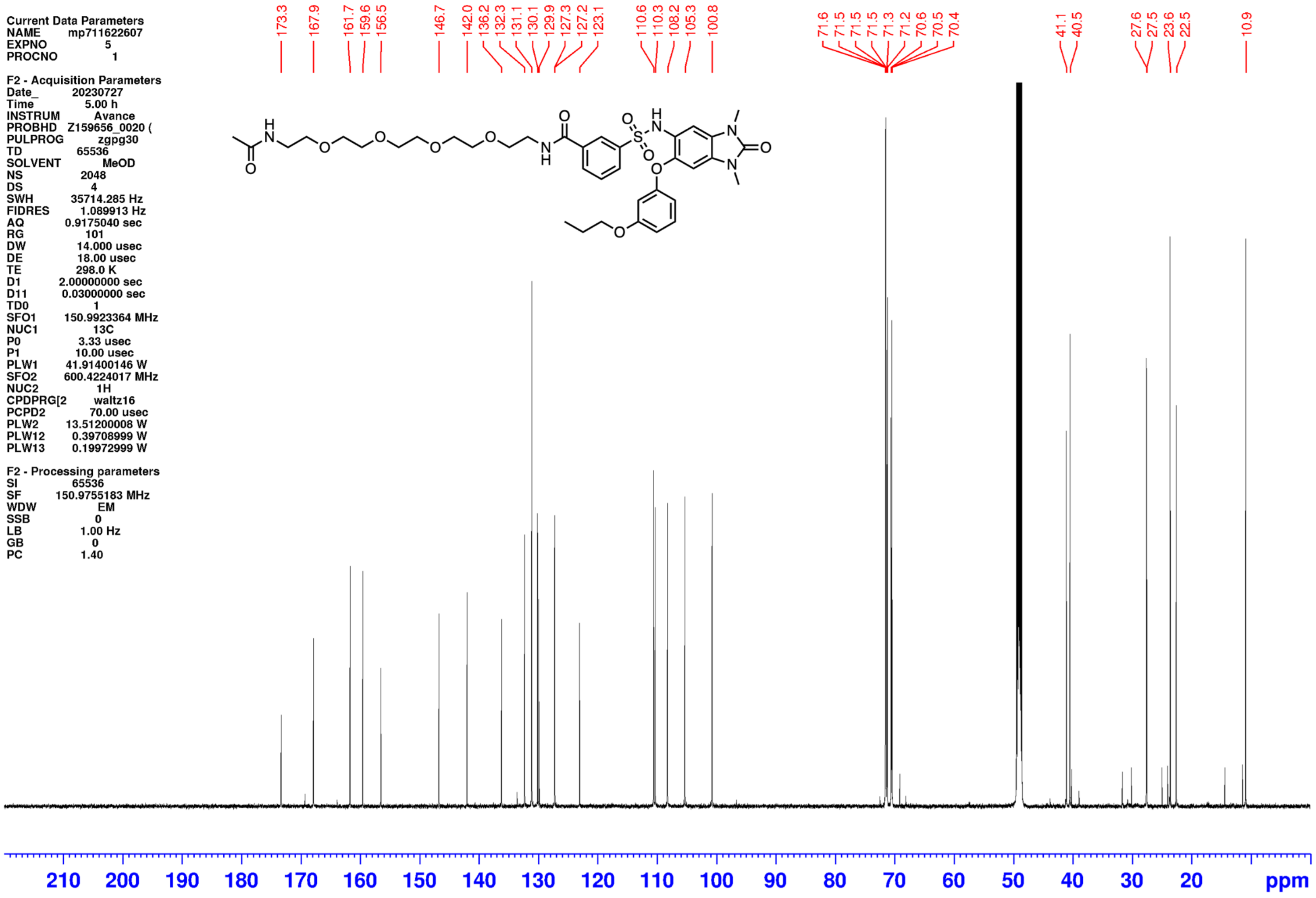

(36) ***tert*-Butyl (*S,E*)-2-((((9*H*-fluoren-9-yl)methoxy)carbonyl)amino)-6-hydroxyhex-4-enoate ^{1}H NMR**

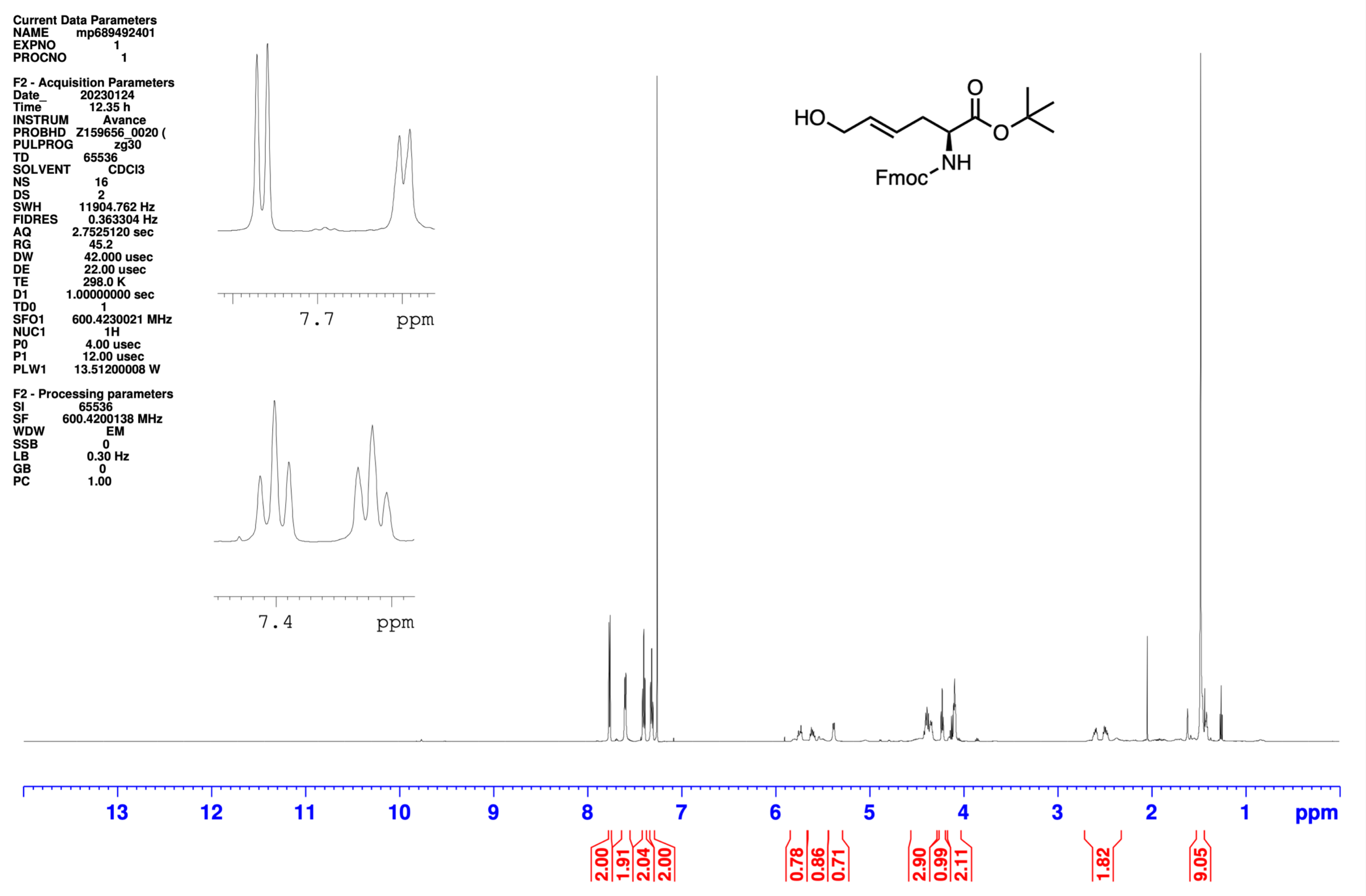

(36) *tert*-Butyl (*S,E*)-2-((((9*H*-fluoren-9-yl)methoxy)carbonyl)amino)-6-hydroxyhex-4-enoate ^{13}C NMR

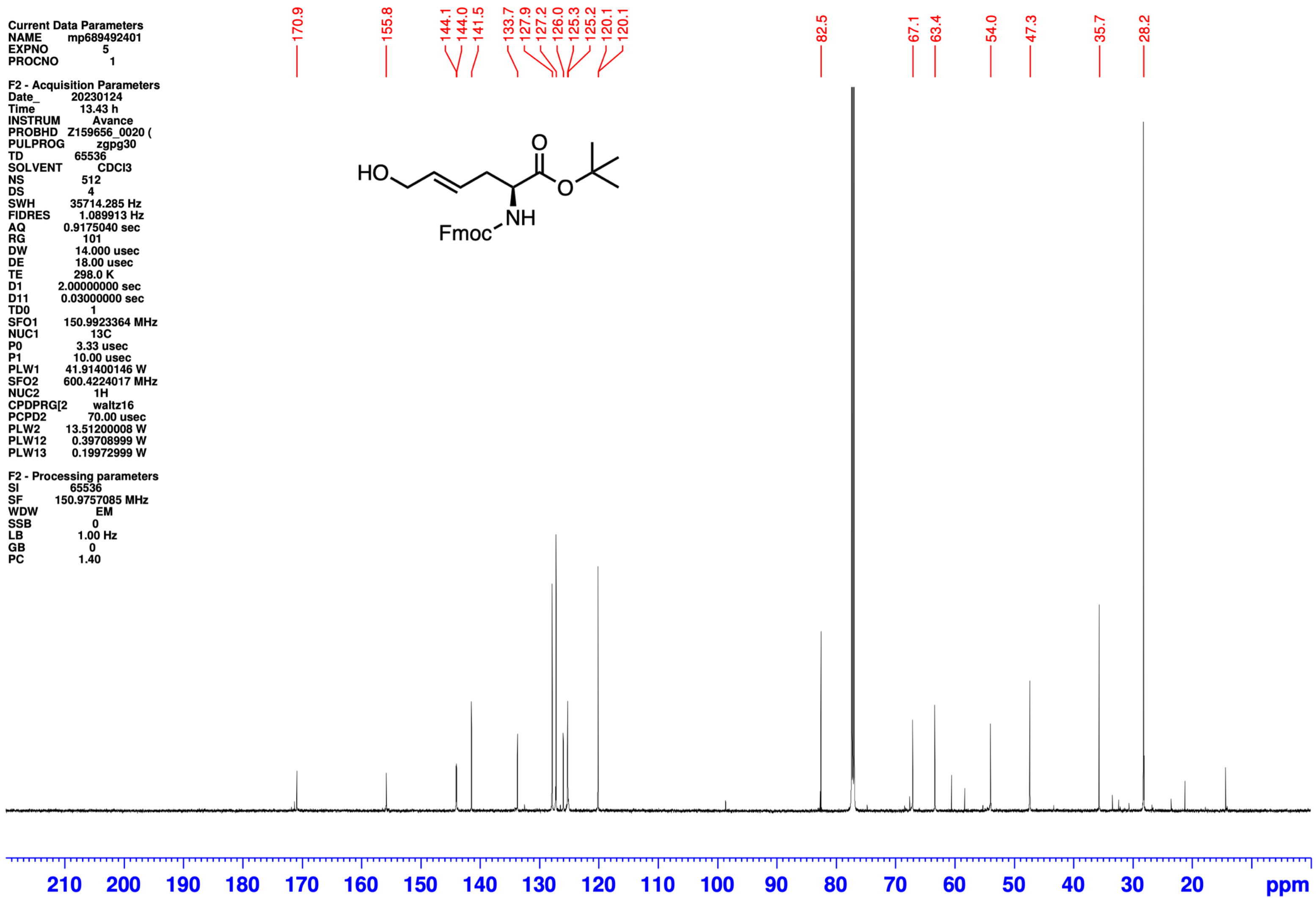

(37) *tert*-Butyl (*S*)-2-((((9*H*-fluoren-9-yl)methoxy)carbonyl)amino)hexanoate ^{1}H NMR

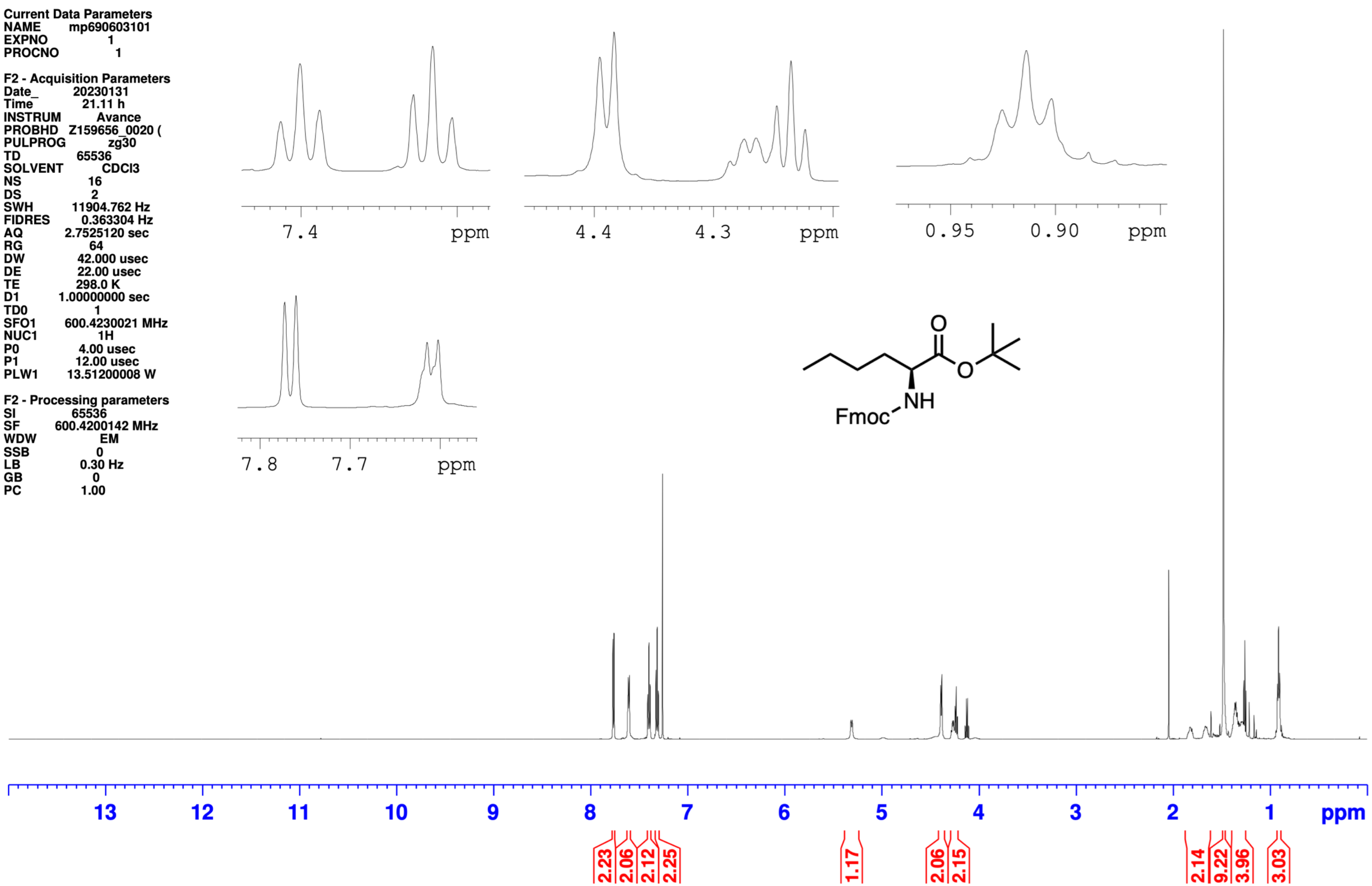

(37) *tert*-Butyl (*S*)-2-((((9*H*-fluoren-9-yl)methoxy)carbonyl)amino)hexanoate ^{13}C NMR

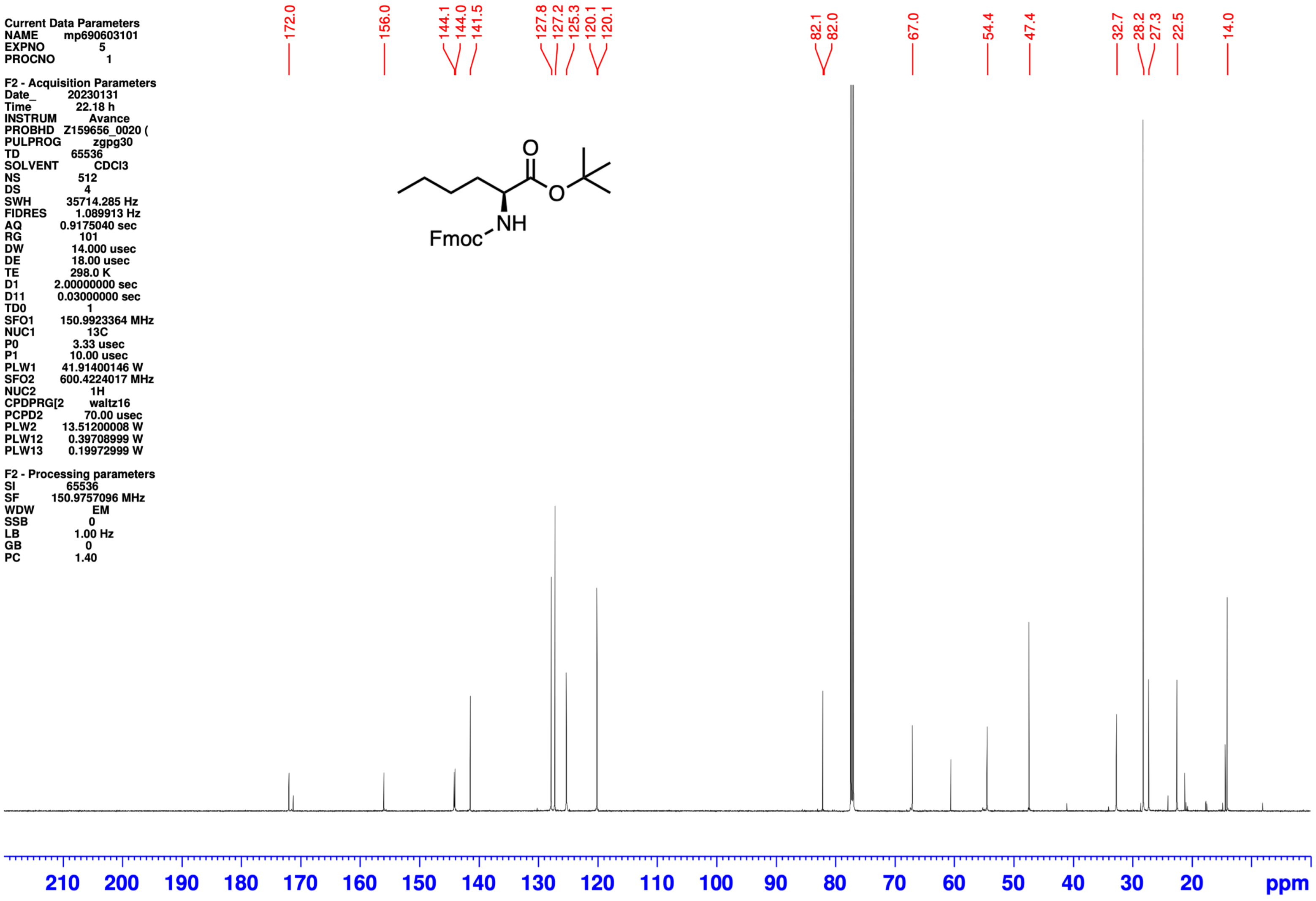

(38) *tert*-Butyl (*S*)-2-((((9*H*-fluoren-9-yl)methoxy)carbonyl)amino)-6-hydroxyhexanoate ^{1}H NMR

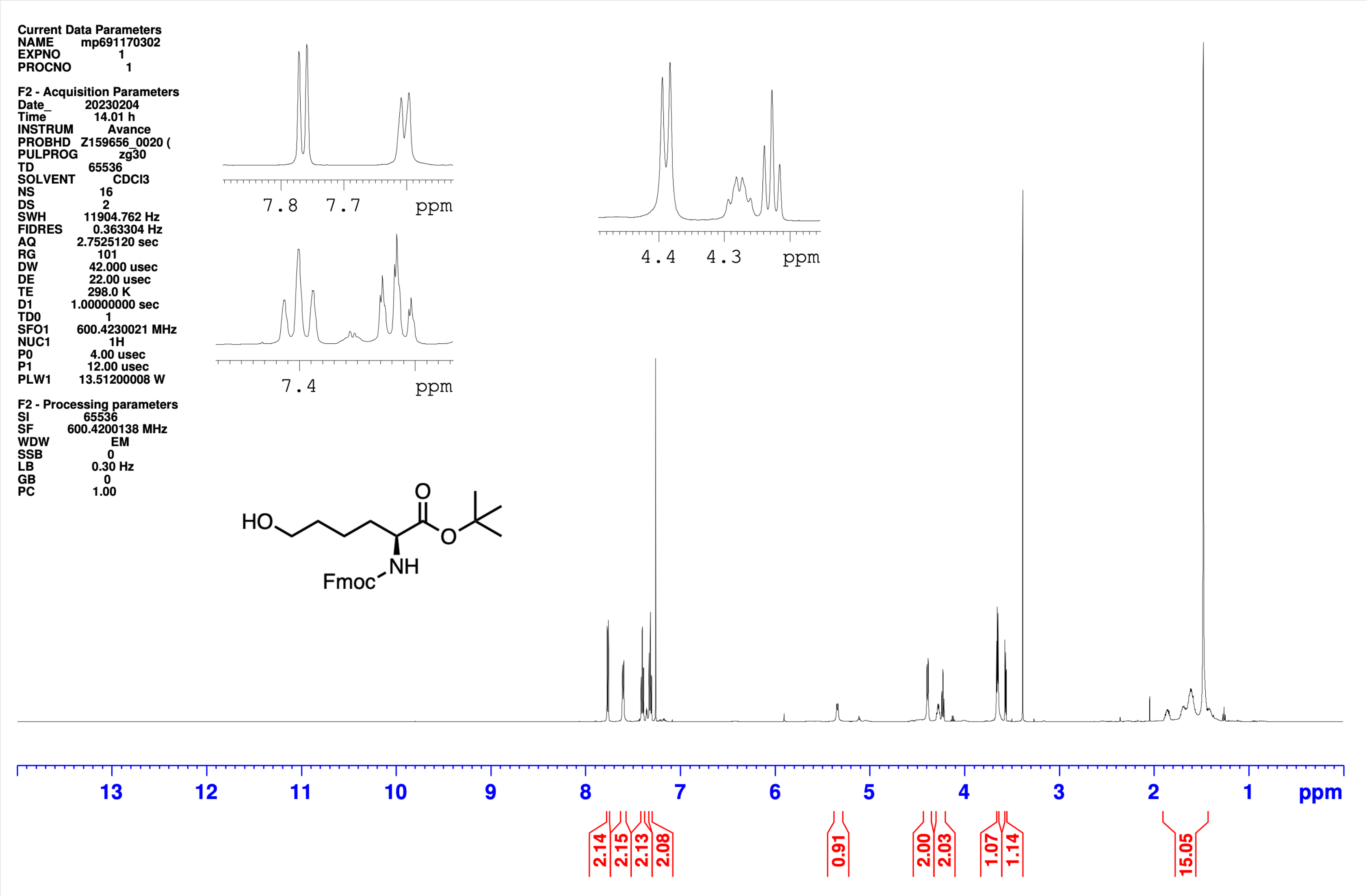

(38) *tert*-Butyl (*S*)-2-((((9*H*-fluoren-9-yl)methoxy)carbonyl)amino)-6-hydroxyhexanoate ^{13}C NMR

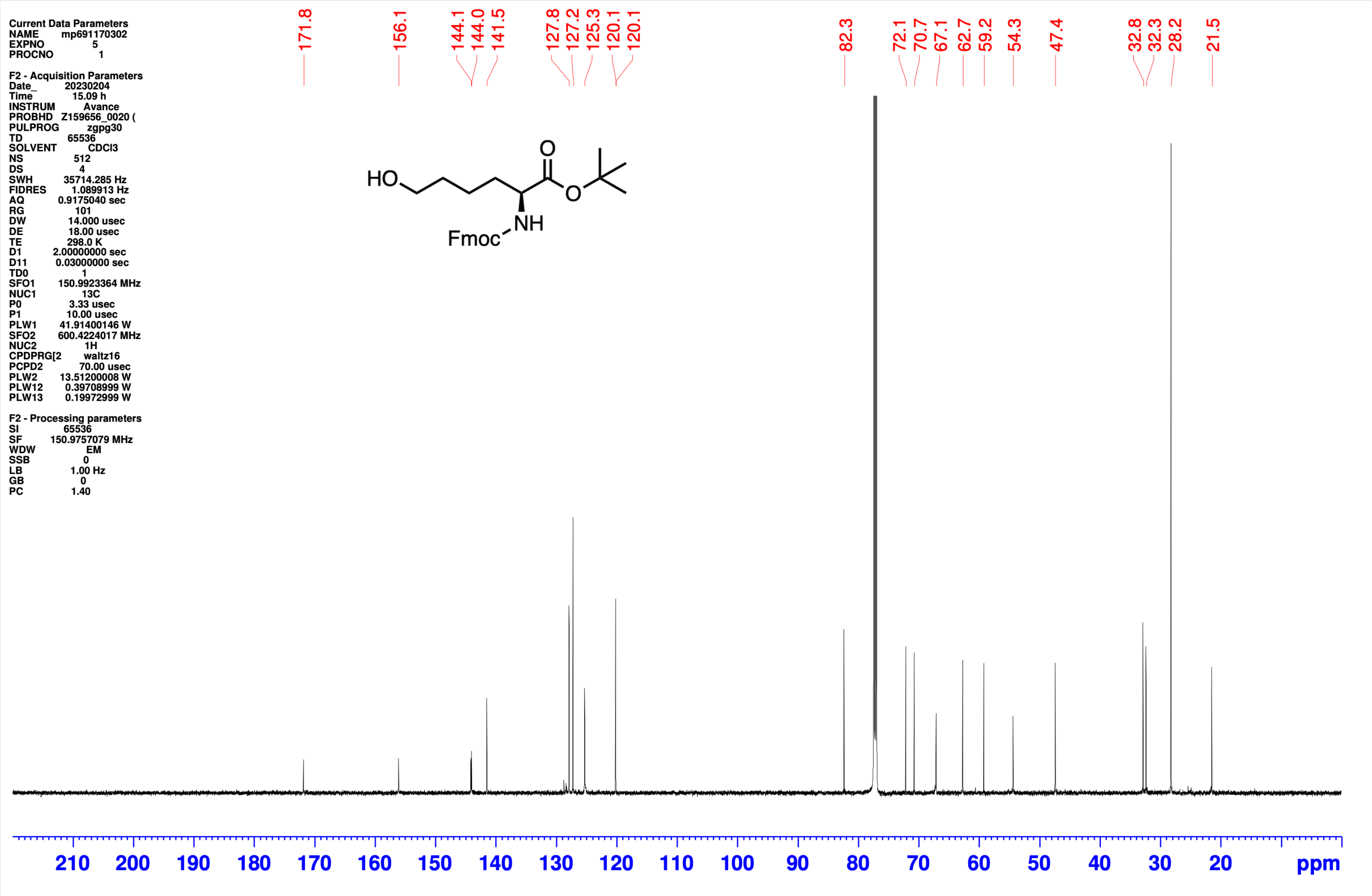

(39) 1-((9*H*-Fluoren-9-yl)methyl) 2-(*tert*-butyl) (*S*)-3,6-dihydropyridine-1,2(2*H*)-dicarboxylate ^{1}H NMR

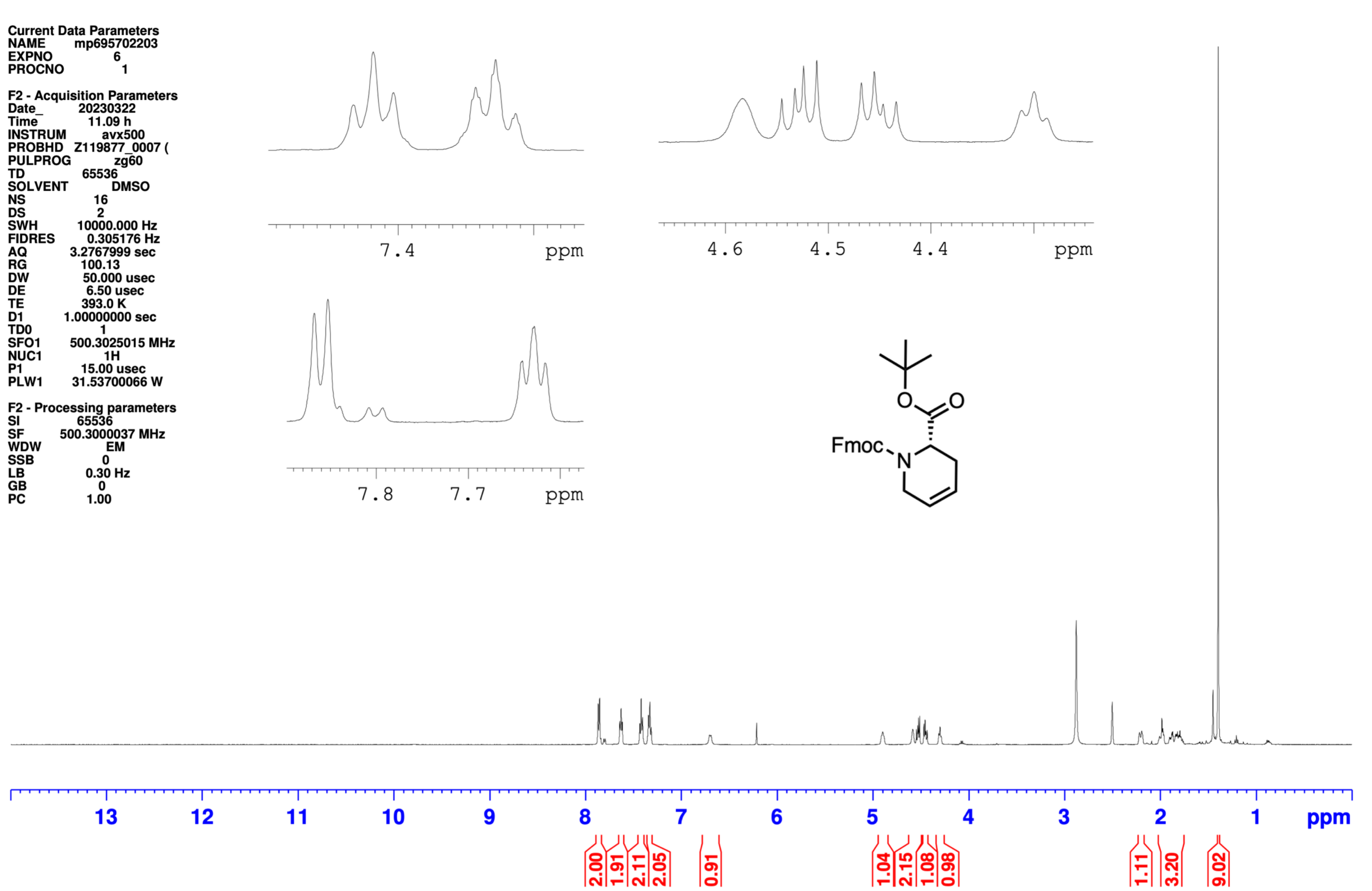

(39) 1-((9*H*-Fluoren-9-yl)methyl) 2-(*tert*-butyl) (*S*)-3,6-dihydropyridine-1,2(2*H*) -dicarboxylate ^{13}C NMR

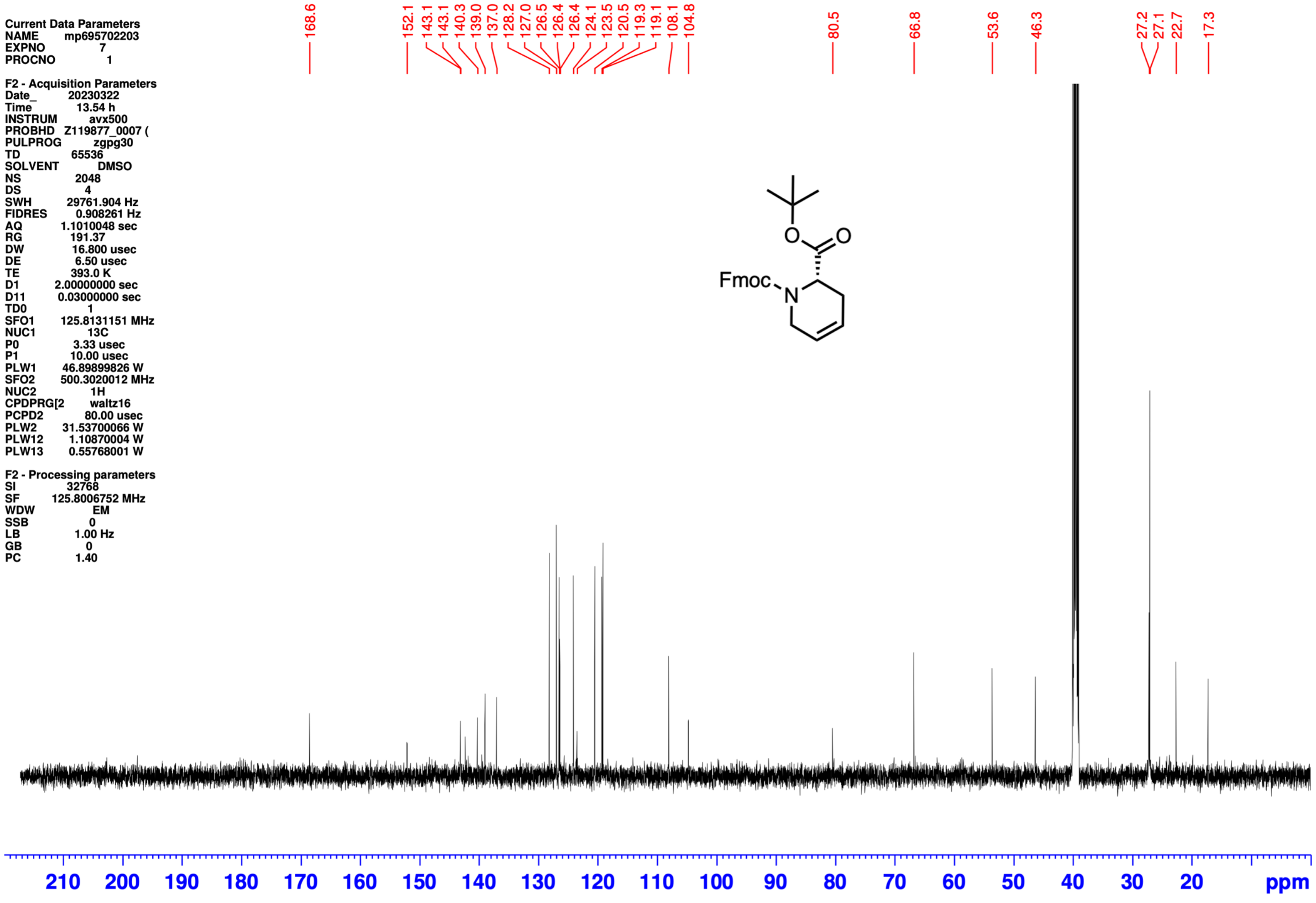

(40) *tert*-Butyl (*S*,*E*)-2-((((9*H*-fluoren-9-yl)methoxy)carbonyl)amino)-6-((methylsulfonyl)oxy)hex-4-enoate ^{1}H NMR

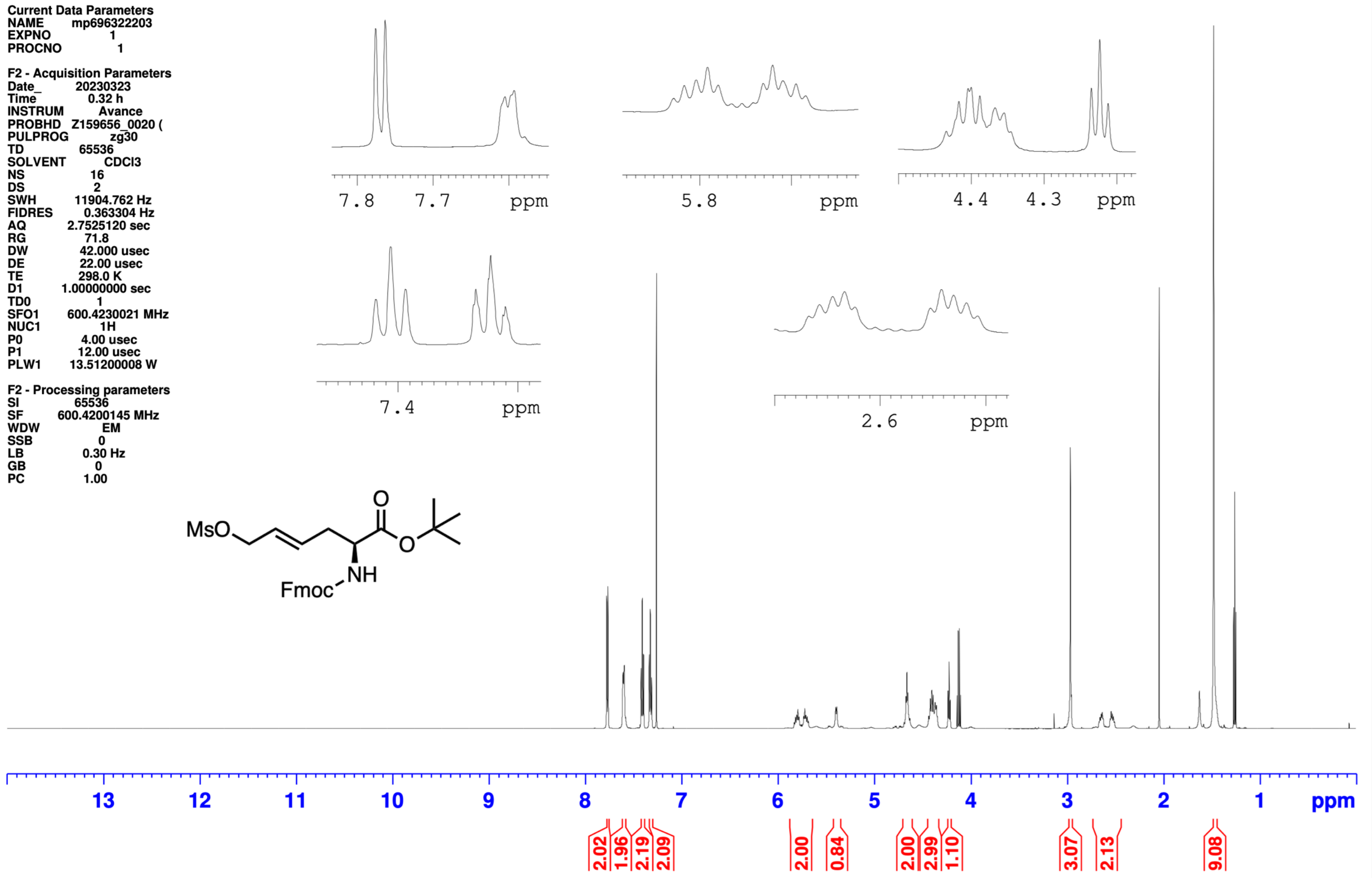

(40) *tert*-Butyl (*S*,*E*)-2-((((9*H*-fluoren-9-yl)methoxy)carbonyl)amino)-6-((methylsulfonyl)oxy)hex-4-enoate ^{13}C NMR

Current Data Parameters
NAME mp696322203
EXPNO 5
PROCNO 1

F2 - Acquisition Parameters
Date_ 20230323
Time 1.40 h
INSTRUM Avance
PROBHD Z159656_0020 (
PULPROG zgpg30
TD 65536
SOLVENT CDCl3
NS 512
DS 4
SWH 35714.285 Hz
FIDRES 1.089913 Hz
AQ 0.9175040 sec
RG 101
DW 14.000 usec
DE 18.00 usec
TE 298.0 K
D1 2.00000000 sec
D11 0.03000000 sec
TD0 1
SFO1 150.9923364 MHz
NUC1 13C
P0 3.33 usec
P1 10.00 usec
PLW1 41.91400146 W
SFO2 600.4224017 MHz
NUC2 1H
CPDPRG[2 waltz16
PCPD2 70.00 usec
PLW2 13.51200008 W
PLW12 0.39708999 W
PLW13 0.19972999 W

F2 - Processing parameters
SI 65536
SF 150.9757089 MHz
WDW EM
SSB 0
LB 1.00 Hz
GB 0
PC 1.40

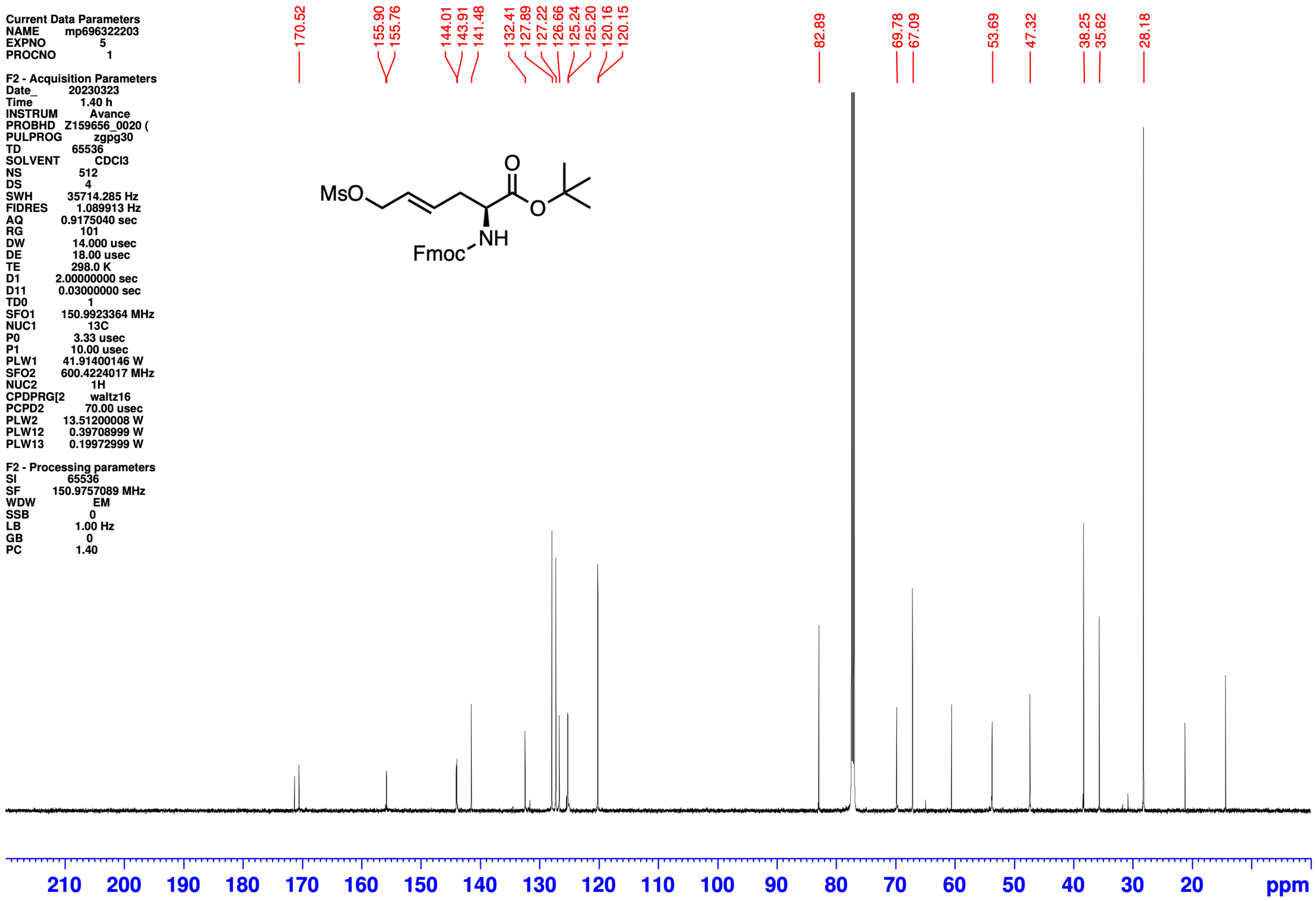

(41) *tert*-Butyl (*S*,*E*)-2-((((9*H*-fluoren-9-yl)methoxy)carbonyl)amino)-6-(isoindolin-2-yl)hex-4-enoate ^{1}H NMR

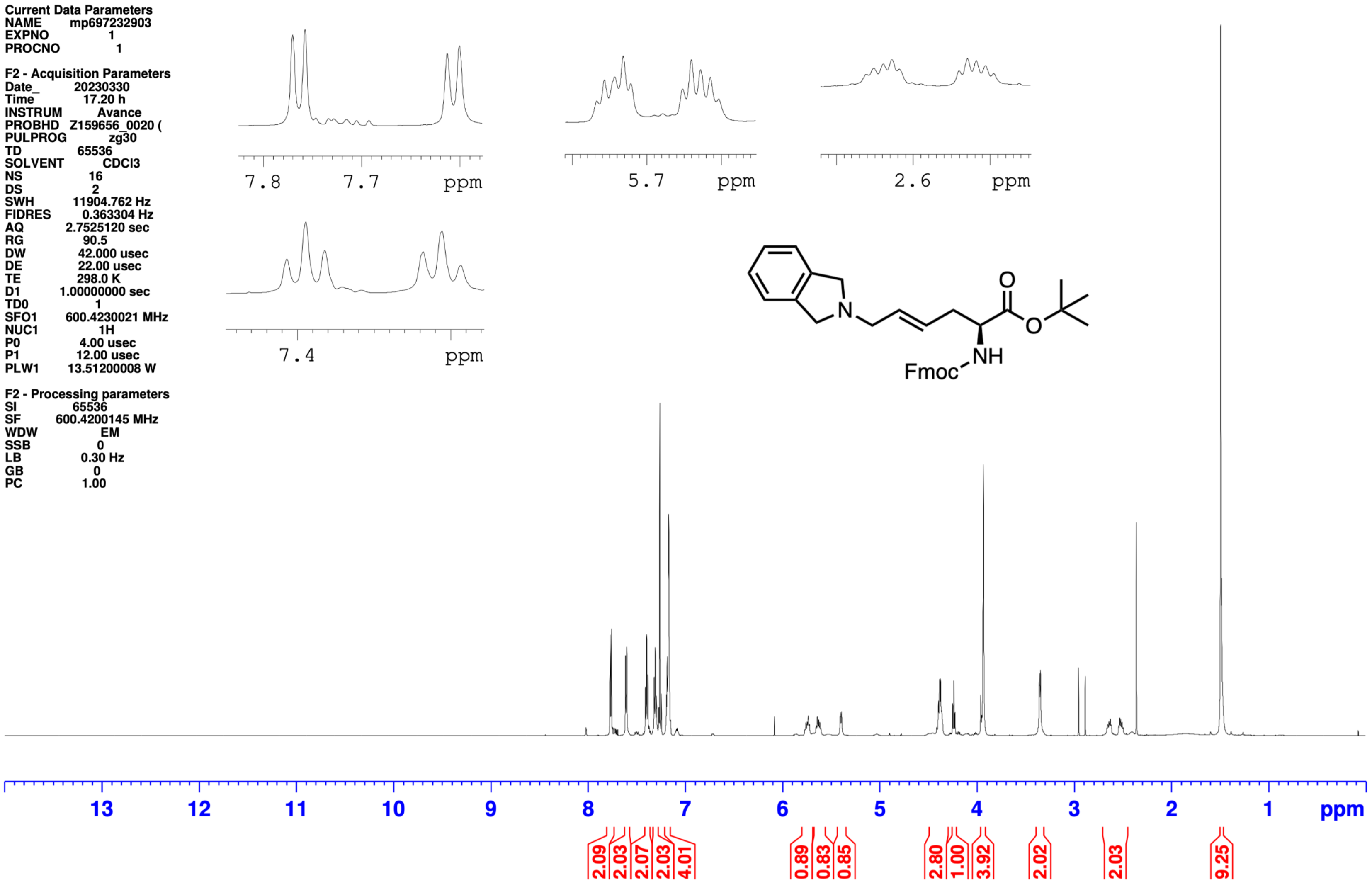

(41) *tert*-Butyl (*S*,*E*)-2-((((9*H*-fluoren-9-yl) methoxy)carbonyl)amino)-6-(isoindolin-2-yl)hex-4-enoate ^{13}C NMR

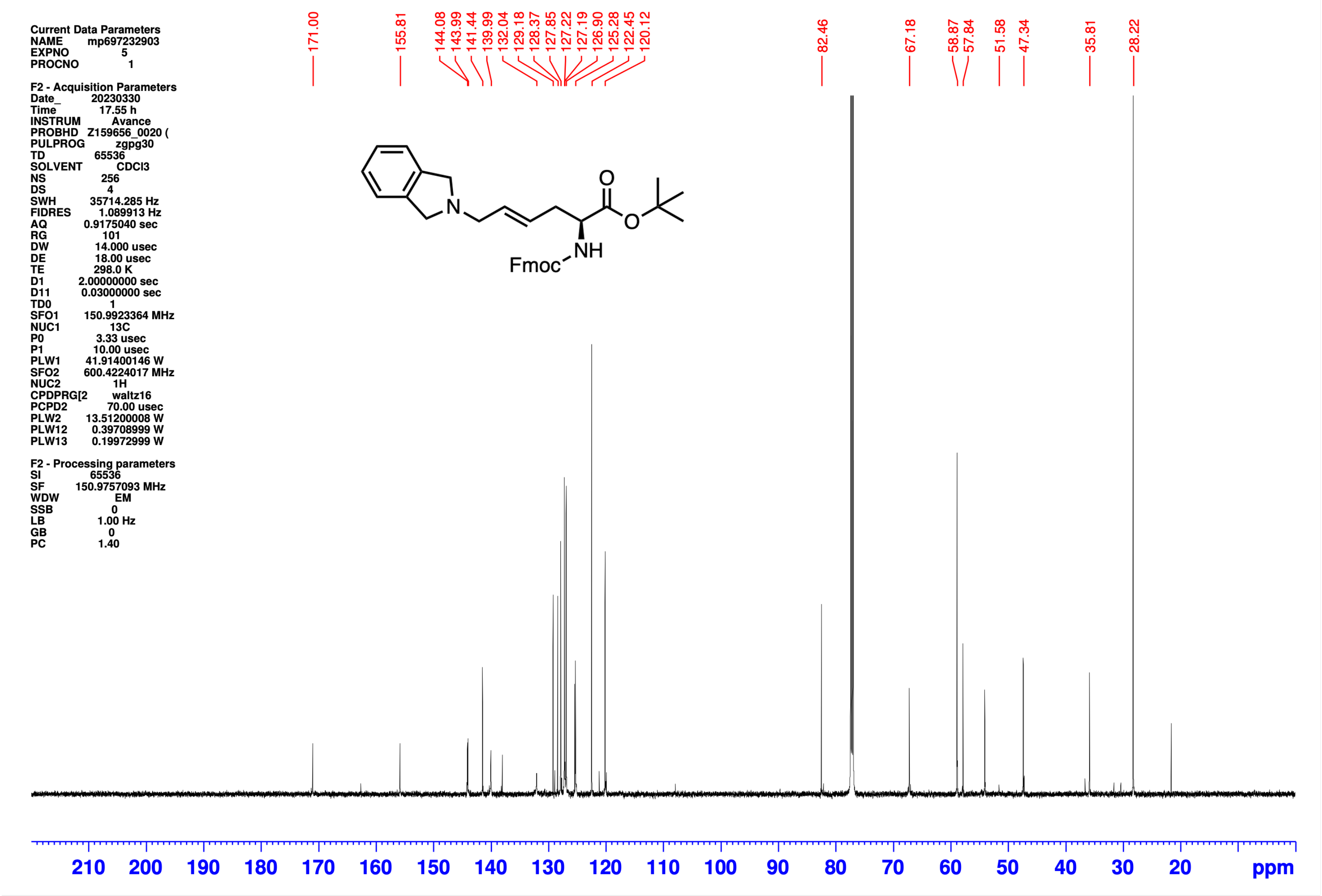

(42) *tert*-Butyl (*S*)-2-((((9*H*-fluoren-9-yl)methoxy)carbonyl)amino)-6-(isoindolin-2-yl)hexanoate ^{1}H NMR

Current Data Parameters
NAME mp697730404
EXPNO 1
PROCNO 1

F2 - Acquisition Parameters
Date_ 20230405
Time 7.28 h
INSTRUM Avance
PROBHD Z159656_0020 (
PULPROG zg30
TD 65536
SOLVENT CDCl3
NS 16
DS 2
SWH 11904.762 Hz
FIDRES 0.363304 Hz
AQ 2.7525120 sec
RG 101
DW 42.000 usec
DE 22.00 usec
TE 298.0 K
D1 1.00000000 sec
TD0 1
SFO1 600.4230021 MHz
NUC1 1H
P0 4.00 usec
P1 12.00 usec
PLW1 13.51200008 W

F2 - Processing parameters
SI 65536
SF 600.4200145 MHz
WDW EM
SSB 0
LB 0.30 Hz
GB 0
PC 1.00

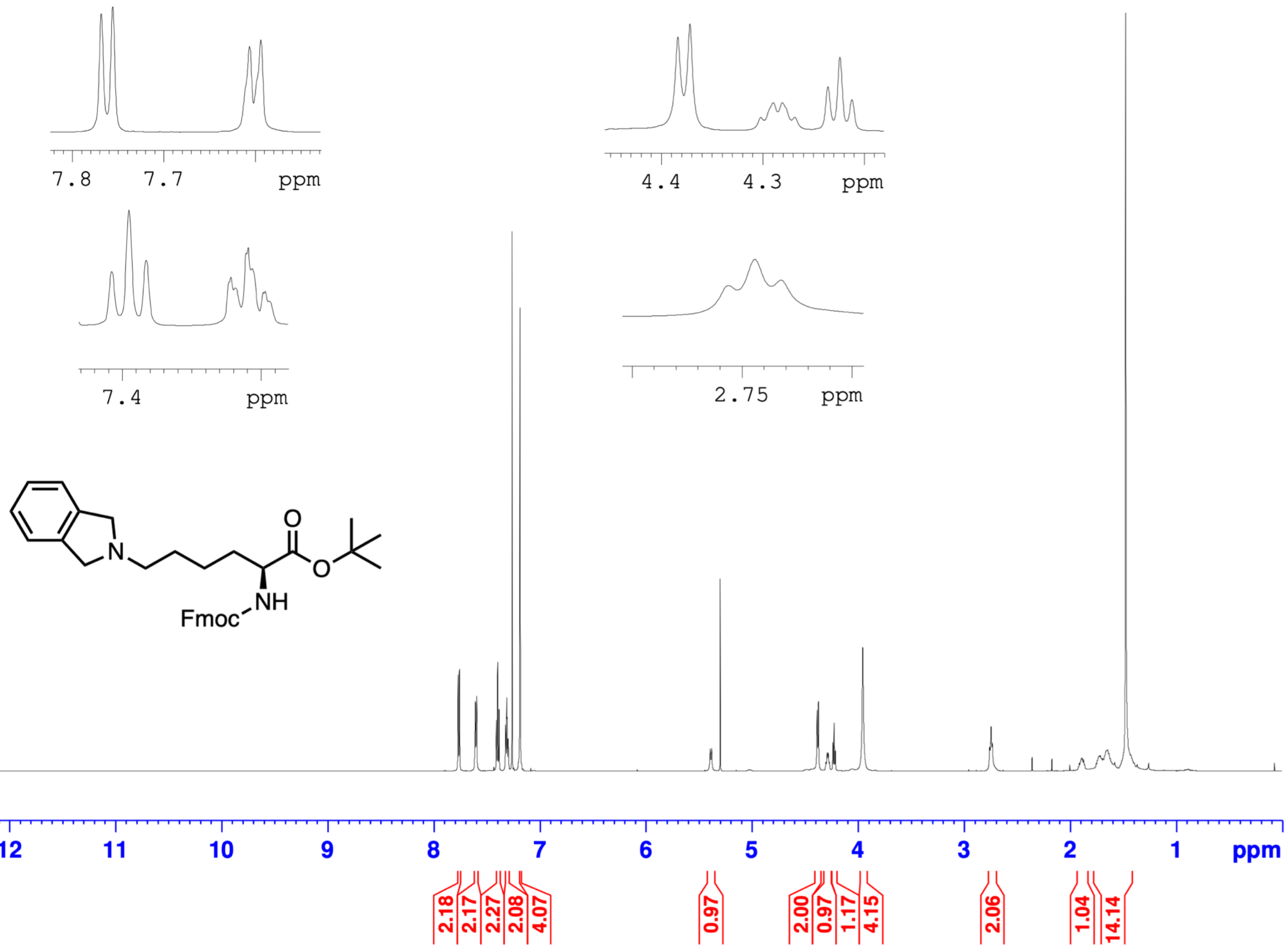

(42) ***tert*-Butyl (*S*)-2-((((9*H*-fluoren-9-yl)methoxy)carbonyl)amino)-6-(isoindolin-2-yl)hexanoate ^{13}C NMR**

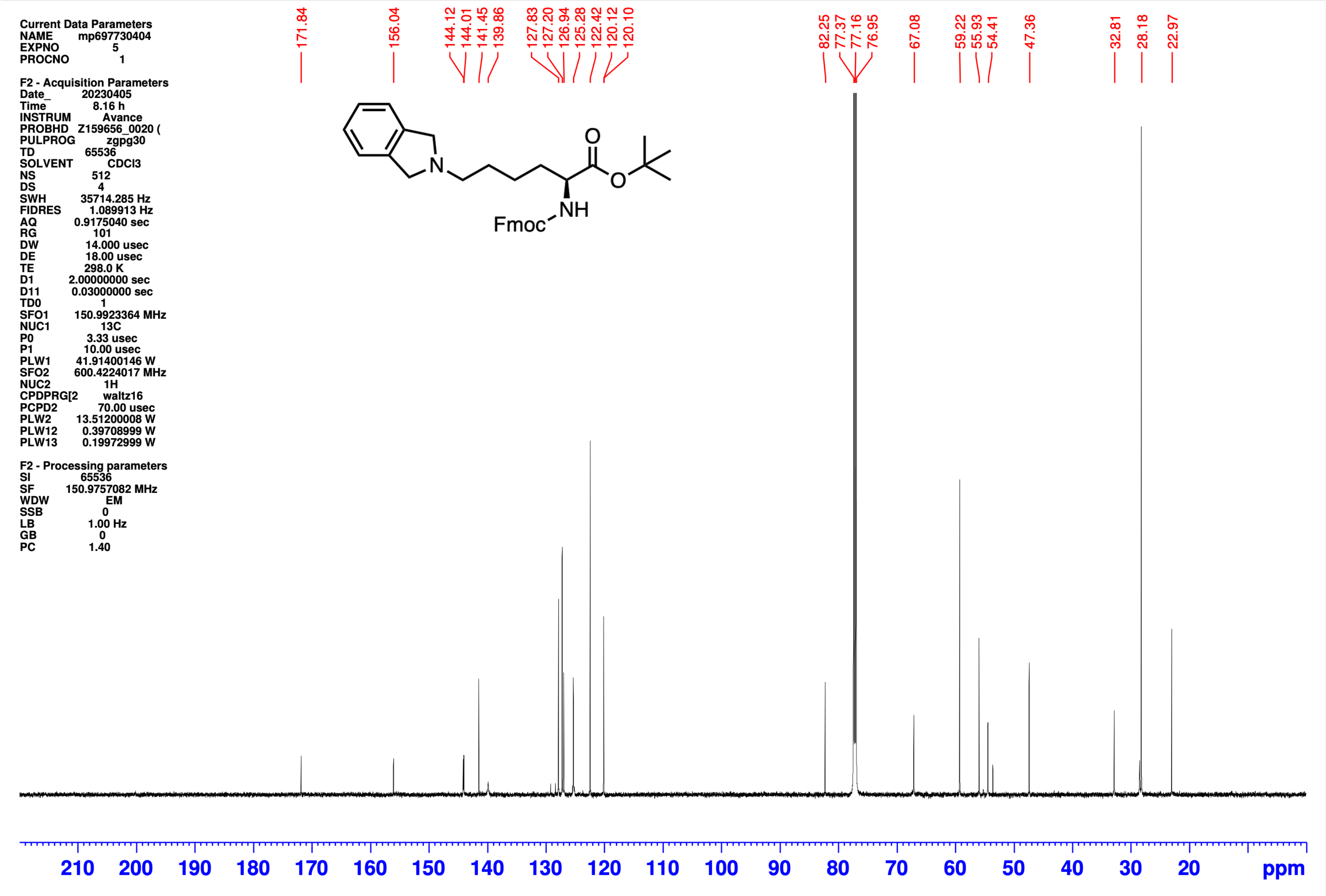

(43) (9*H*-Fluoren-9-yl)methyl (*S*)-(1-(3-(*N*-(1,3-dimethyl-2-oxo-6-(3-propoxyphenoxy)-2,3-dihydro-1*H*-benzo[*d*]imidazol-5-yl)sulfamoyl)phenyl)-17-(isoindolin-2-yl)-1,12-dioxo-5,8-dioxa-2,11-diazaheptadecan-13-yl)carbamate ^{1}H NMR

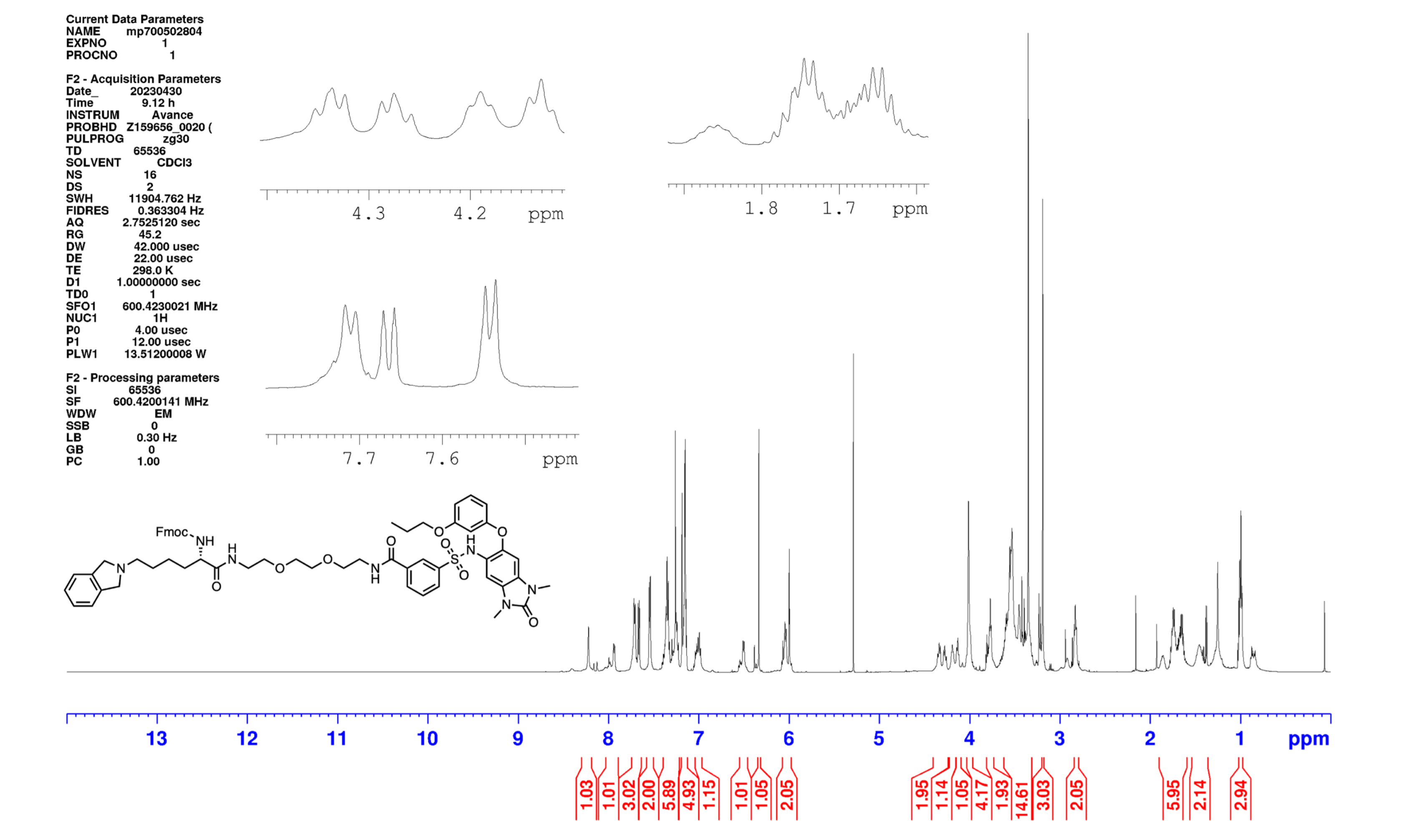

(43) (9*H*-Fluoren-9-yl)methyl (*S*)-(1-(3-(*N*-(1,3-dimethyl-2-oxo-6-(3-propoxyphenoxy)-2,3-dihydro-1*H*-benzo[*d*]imidazol-5-yl)sulfamoyl)phenyl)-17-(isoindolin-2-yl)-1,12-dioxo-5,8-dioxa-2,11-diazaheptadecan-13-yl)carbamate ^{13}C NMR

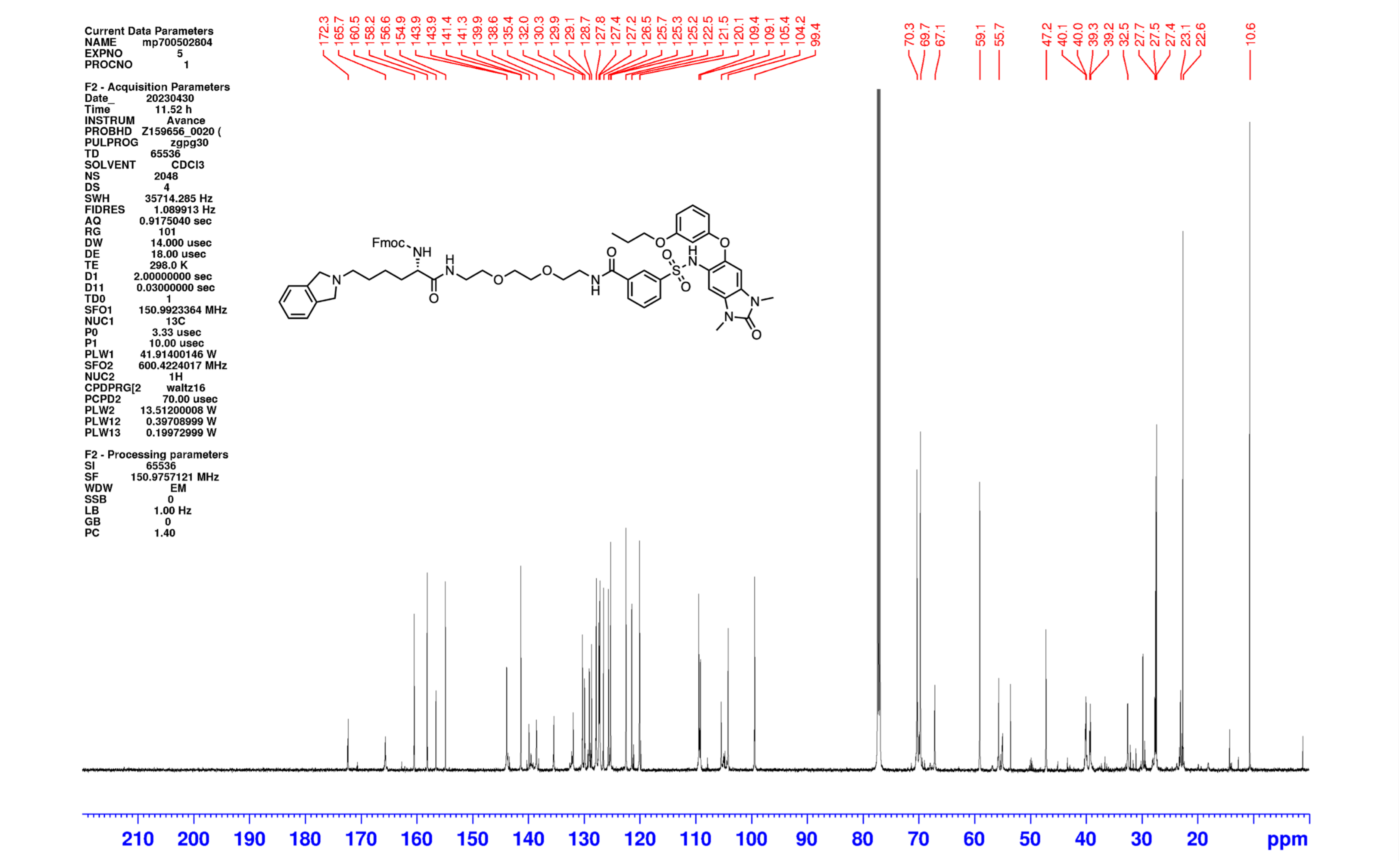

(44) (9*H*-Fluoren-9-yl)methyl (*S*)-(1-(3-(*N*-(1,3-dimethyl-2-oxo-6-(3-propoxyphenoxy)-2,3-dihydro-1*H*-benzo[*d*]imidazol-5-yl)sulfamoyl)phenyl)-20-(isoindolin-2-yl)-1,15-dioxo-5,8,11-trioxa-2,14-diazaicosan-16-yl)carbamate ^{1}H NMR

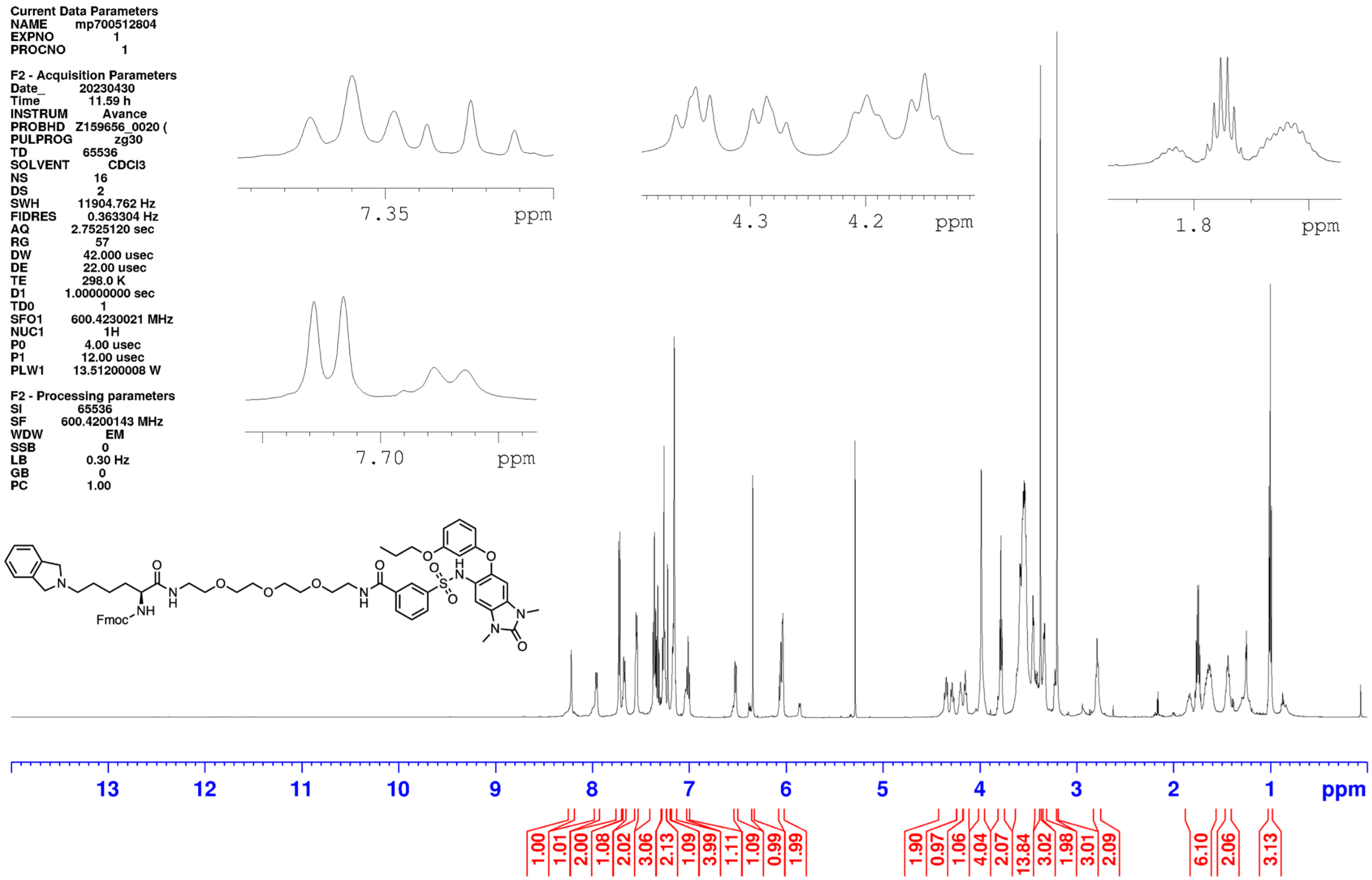

(44) (9*H*-Fluoren-9-yl)methyl (*S*)-(1-(3-(*N*-(1,3-dimethyl-2-oxo-6-(3-propoxyphenoxy)-2,3-dihydro-1*H*-benzo[*d*]imidazol-5-yl)sulfamoyl)phenyl)-20-(isoindolin-2-yl)-1,15-dioxo-5,8,11-trioxa-2,14-diazaicosan-16-yl)carbamate ^{13}C NMR

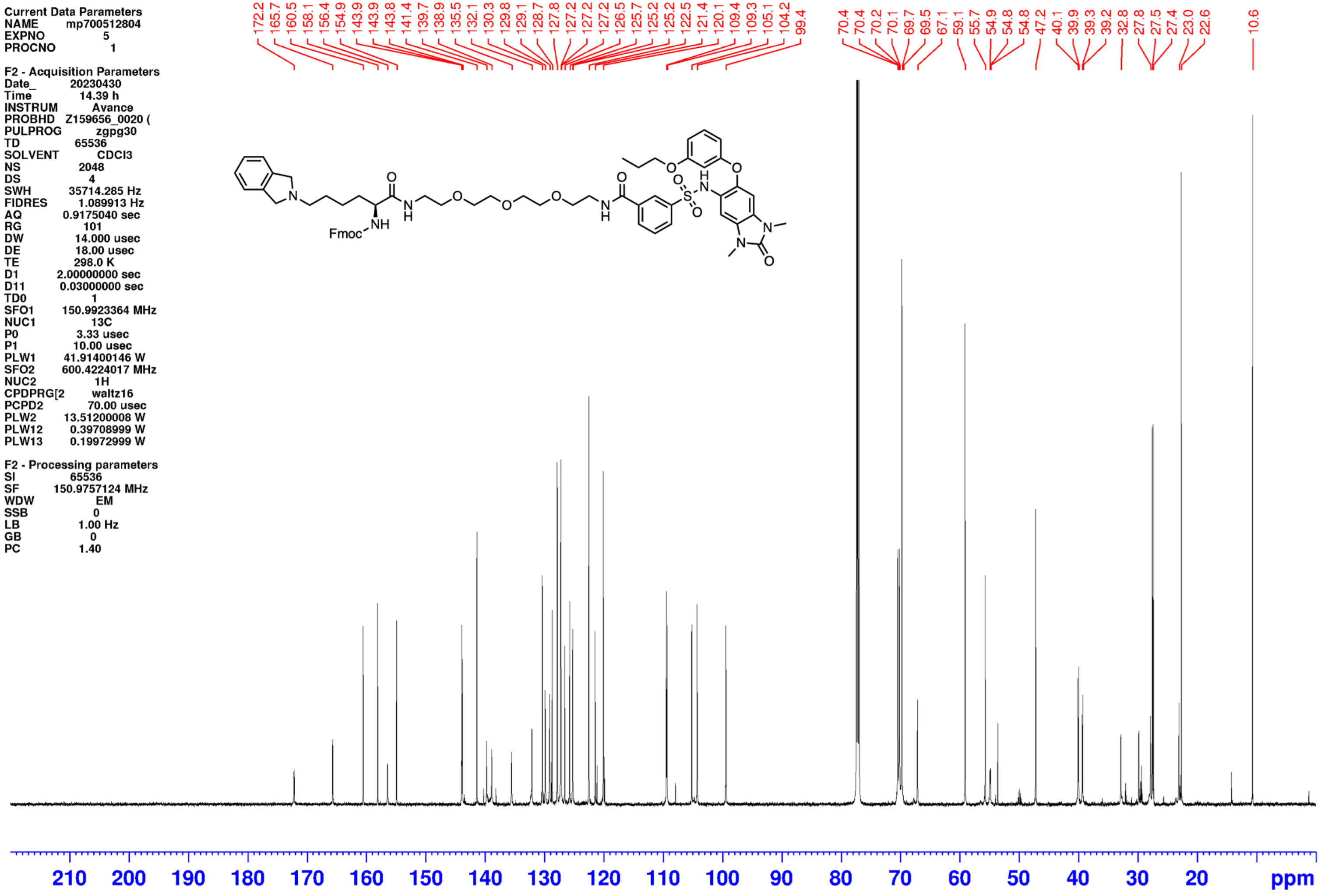

(45) (9*H*-Fluoren-9-yl)methyl (*S*)-(1-(3-(*N*-(1,3-dimethyl-2-oxo-6-(3-propoxyphenoxy)-2,3-dihydro-1*H*-benzo[*d*]imidazol-5-yl)sulfamoyl)phenyl)-23-(isoindolin-2-yl)-1,18-dioxo-5,8,11,14-tetraoxa-2,17-diazatricosan-19-yl)carbamate ^{1}H NMR

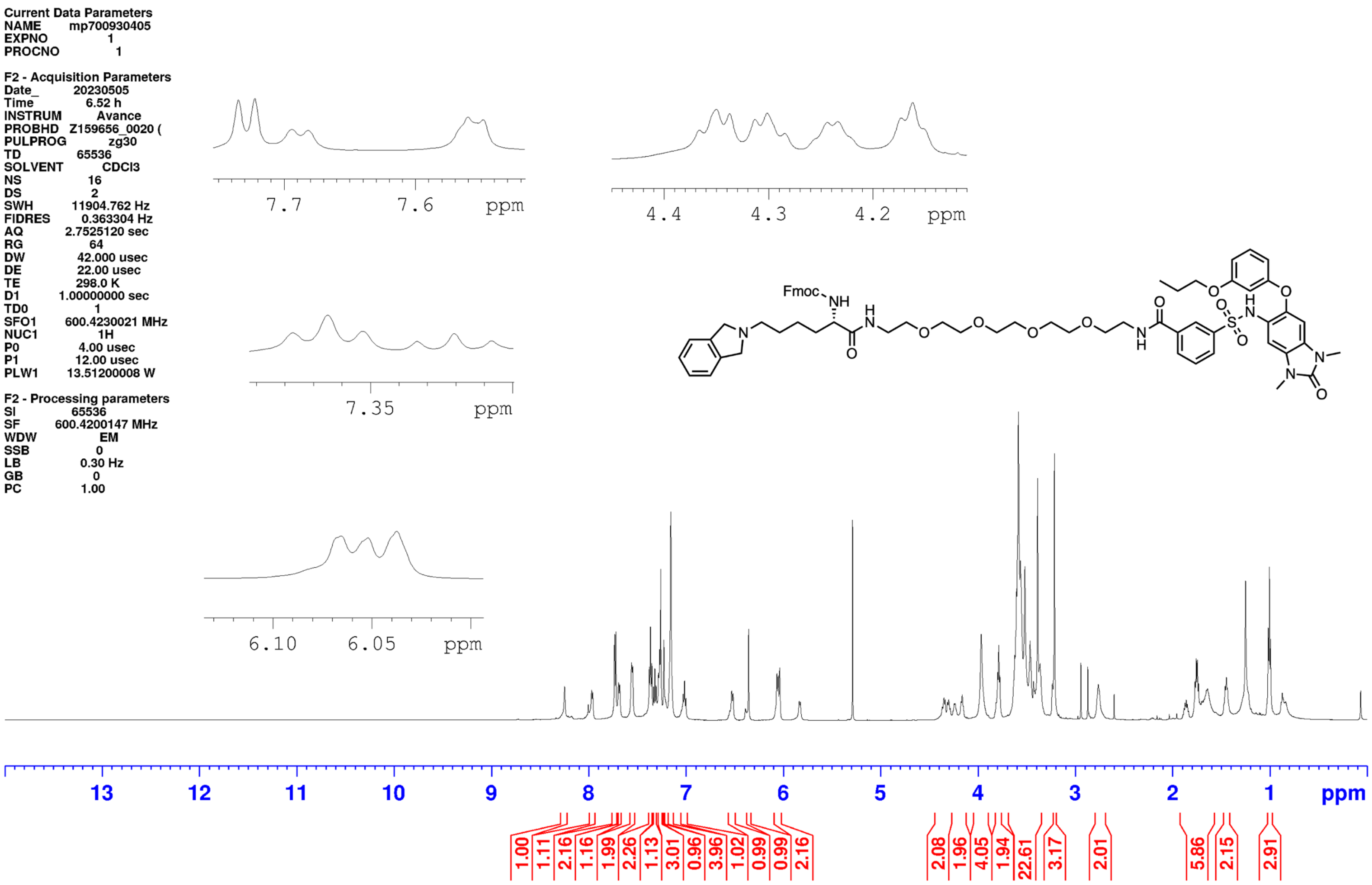

(45) (9*H*-Fluoren-9-yl)methyl (*S*)-(1-(3-(*N*-(1,3-dimethyl-2-oxo-6-(3-propoxyphenoxy)-2,3-dihydro-1*H*-benzo[*d*]imidazol-5-yl)sulfamoyl)phenyl)-23-(isoindolin-2-yl)-1,18-dioxo-5,8,11,14-tetraoxa-2,17-diazatricosan-19-yl)carbamate ^{13}C NMR

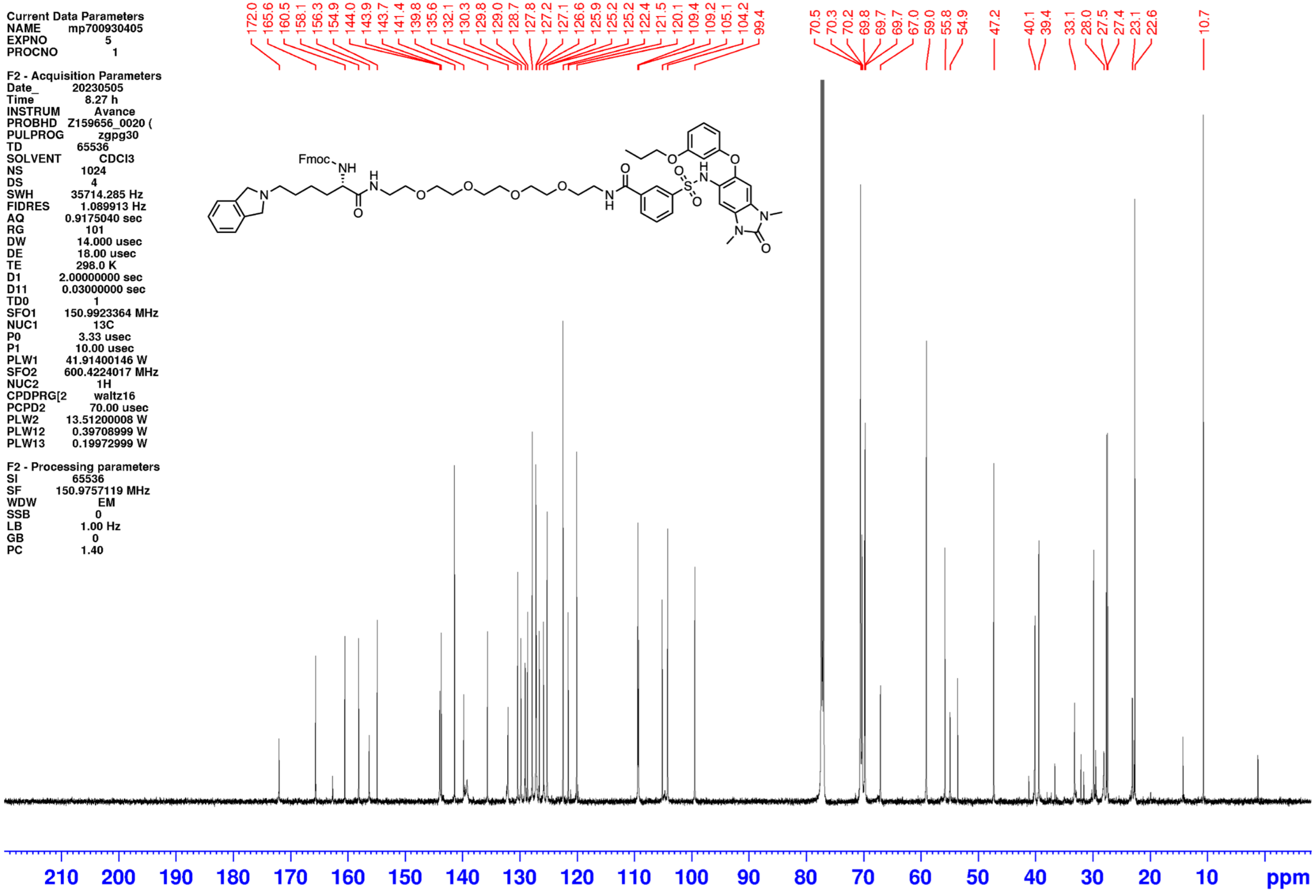

(53) Benzyl (*S*)-(1-(methylamino)-1-oxopent-4-en-2-yl)carbamate ^{1}H NMR

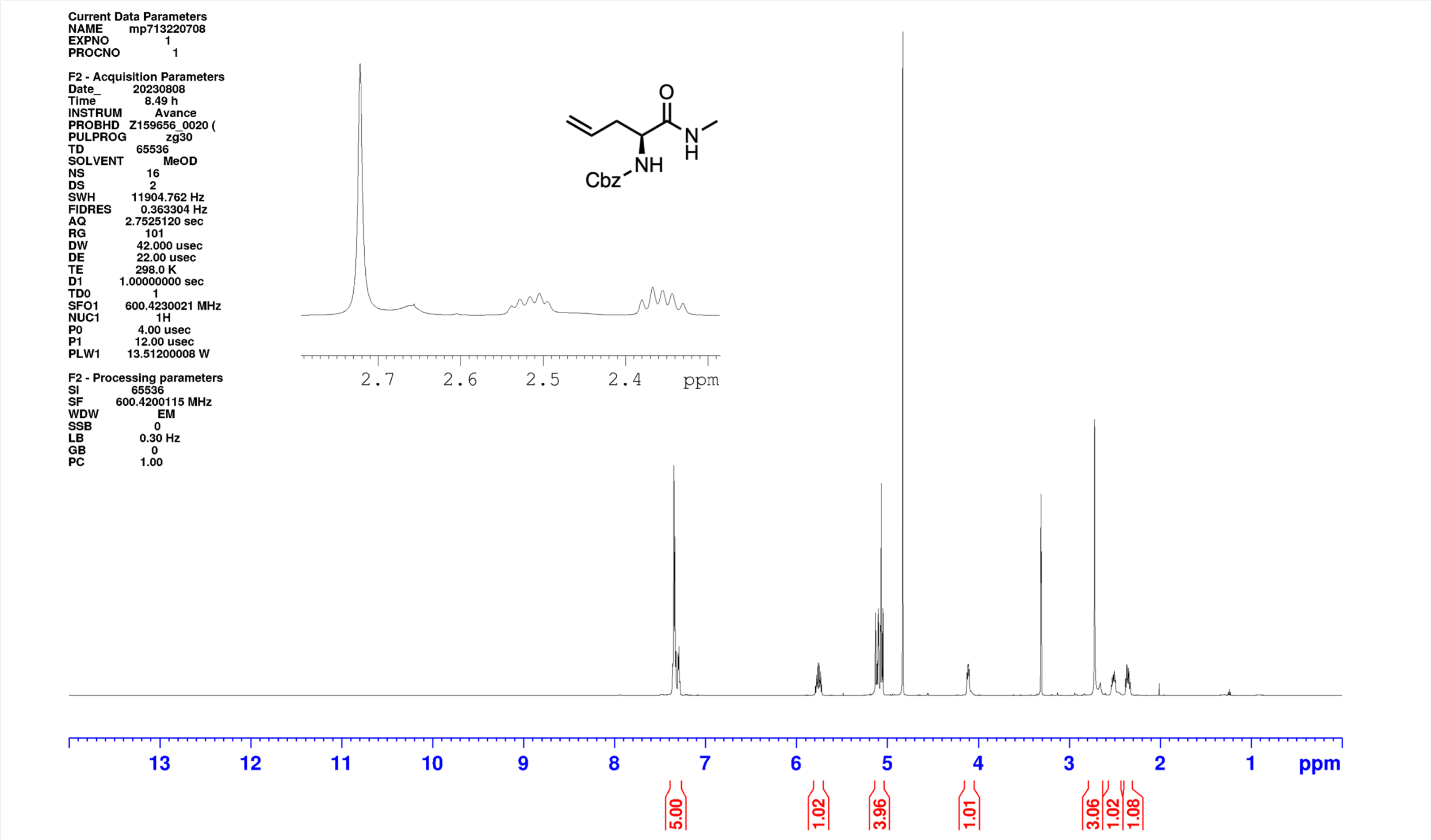

(53) Benzyl (*S*)-(1-(methylamino)-1-oxopent-4-en-2-yl)carbamate ^{13}C NMR

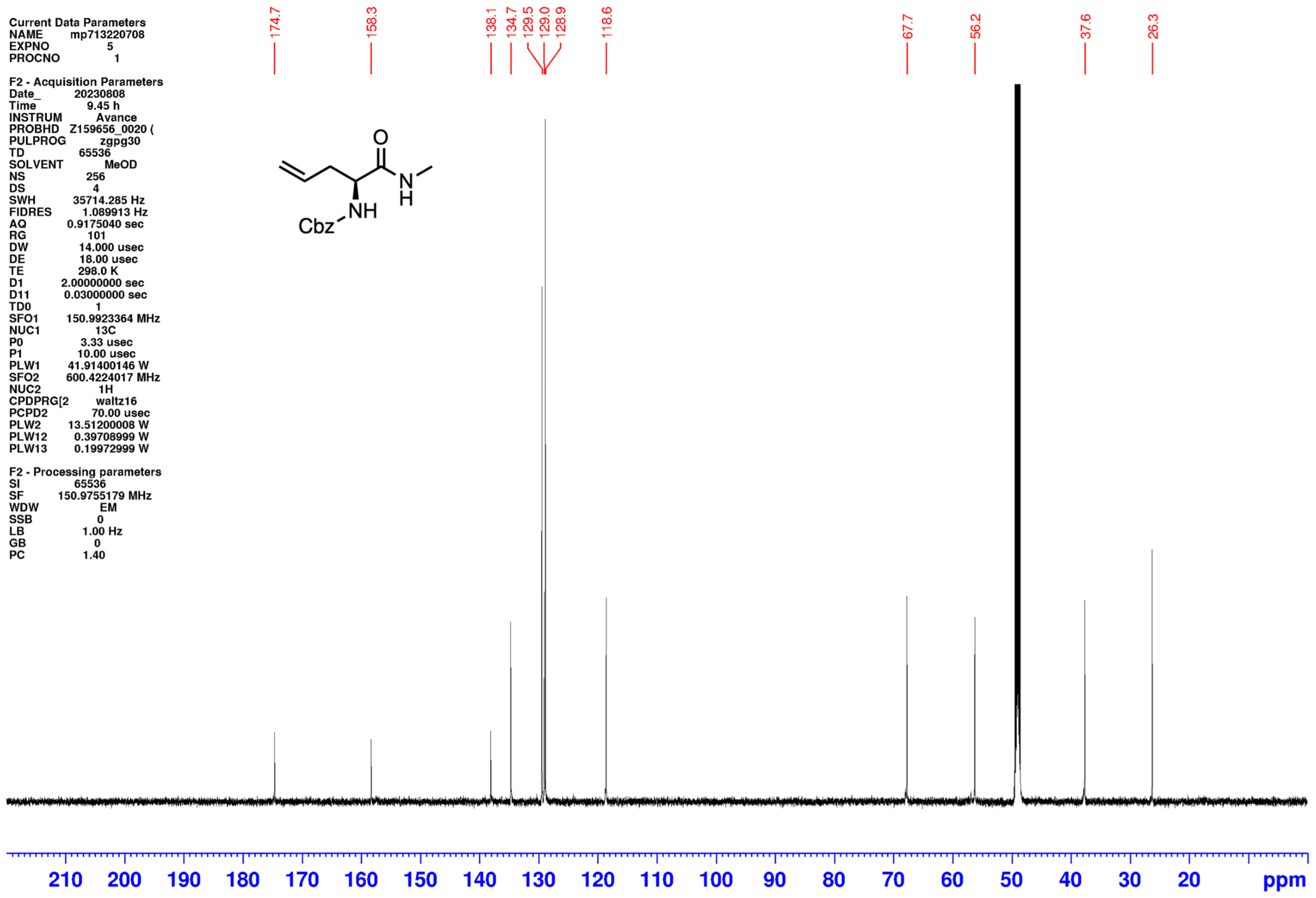

(54) Benzyl (*S*,*E*)-(6-(isoindolin-2'''-yl)-1-(methylamino)-1-oxohex-4-en-2-yl)carbamate hydrochloride ^{1}H NMR

Current Data Parameters
NAME mp714251708
EXPNO 1
PROCNO 1

F2 - Acquisition Parameters
Date_ 20230817
Time 18.31 h
INSTRUM av700
PROBHD Z44908_0059 (C
PULPROG zg60
TD 65536
SOLVENT MeOD
NS 16
DS 2
SWH 11160.714 Hz
FIDRES 0.340598 Hz
AQ 2.9360127 sec
RG 5.6
DW 44.800 usec
DE 10.00 usec
TE 298.0 K
D1 1.00000000 sec
TD0 1
SFO1 699.8534993 MHz
NUC1 1H
P1 11.00 usec
PLW1 5.01189995 W

F2 - Processing parameters
SI 65536
SF 699.8500134 MHz
WDW EM
SSB 0
LB 0.30 Hz
GB 0
PC 1.00

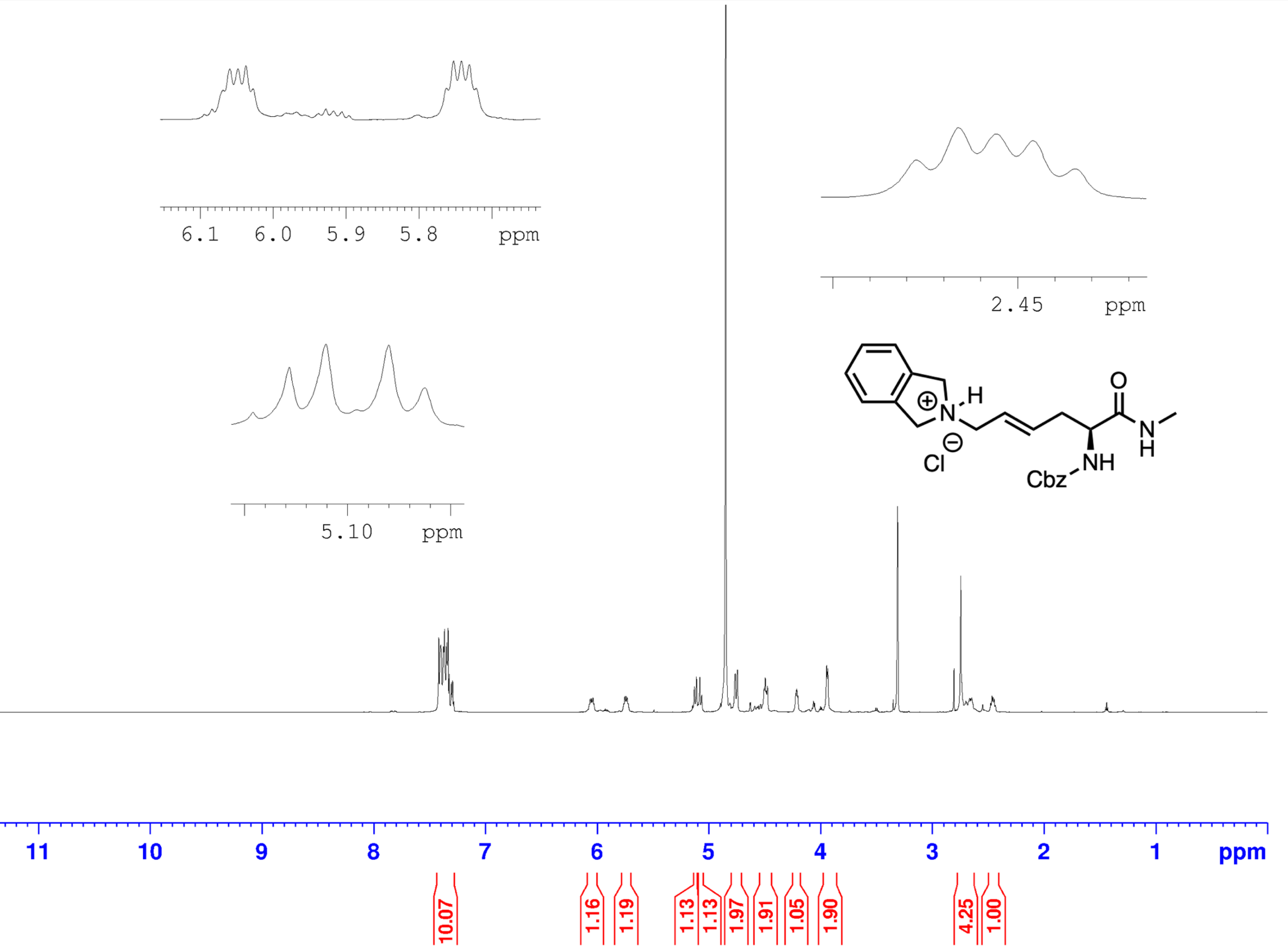

(54) Benzyl (*S*,*E*)-(6-(isoindolin-2'''-yl)-1-(methylamino)-1-oxohex-4-en-2-yl)carbamate hydrochloride ^{13}C NMR

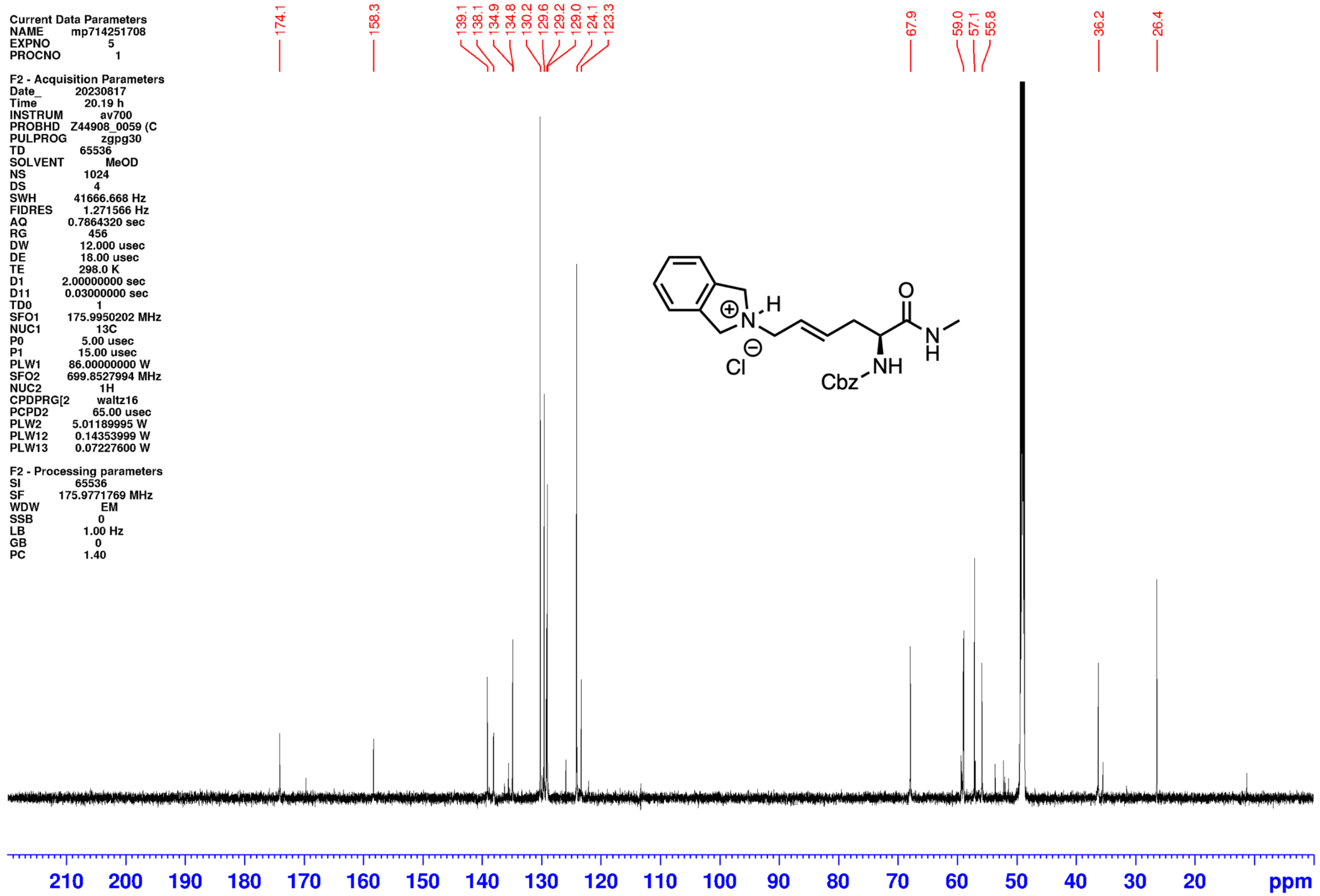

7 LC-MS Chromatograms for 50–52 and 56

(50) PDC4

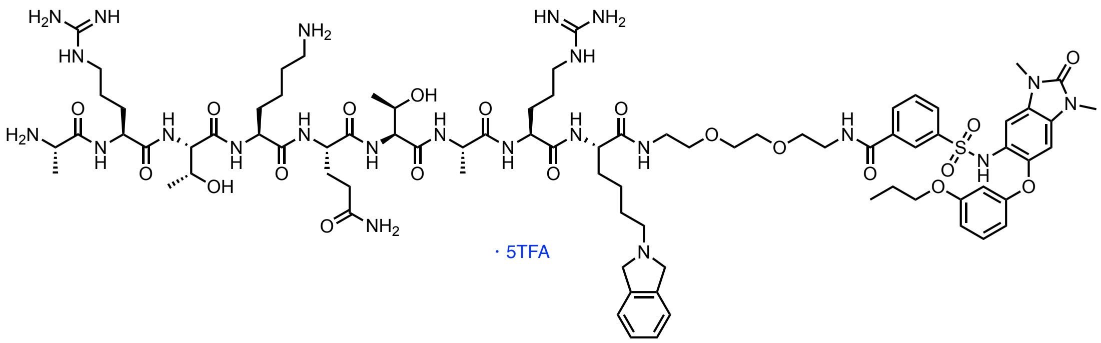

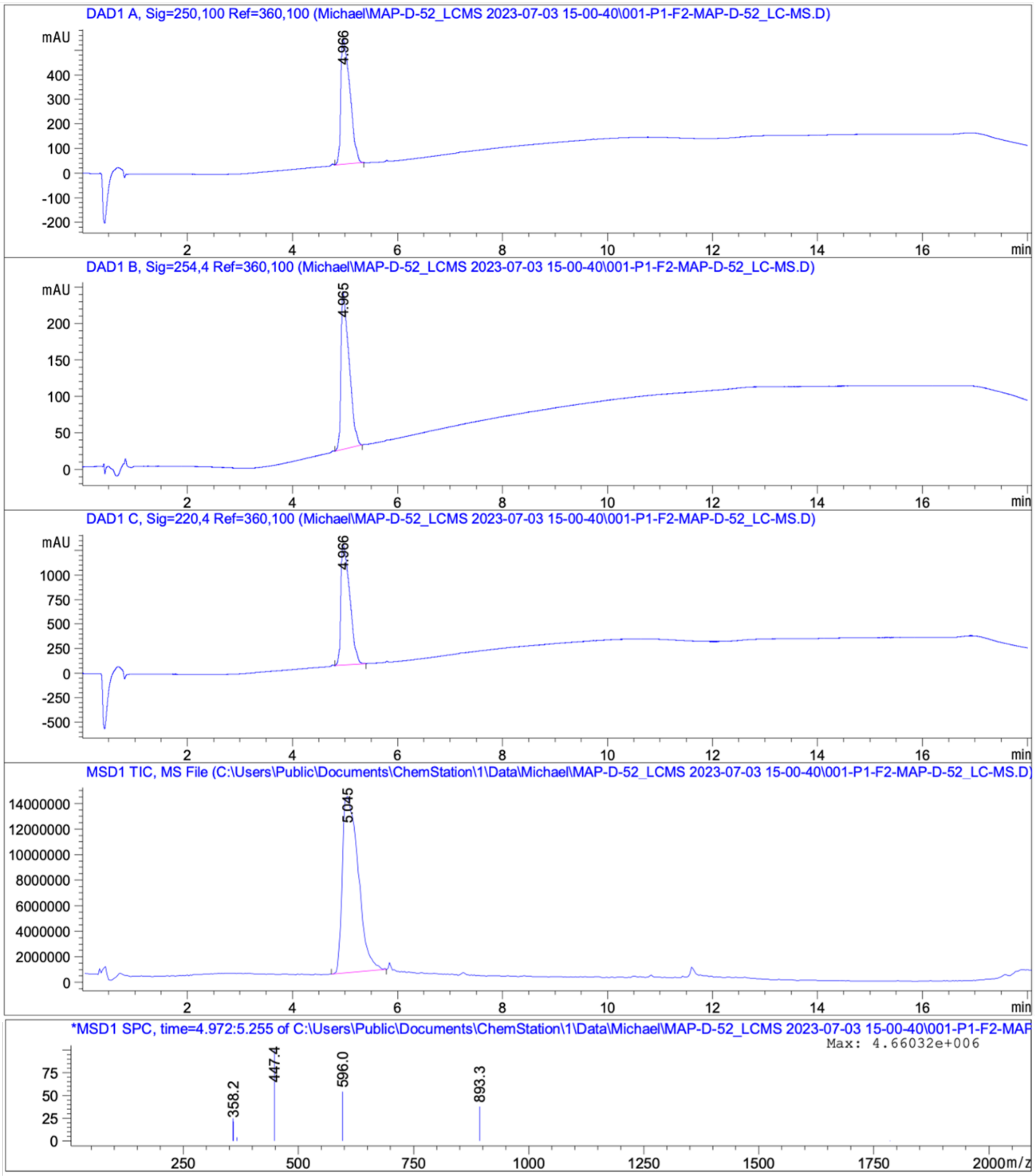

(51) PDC5

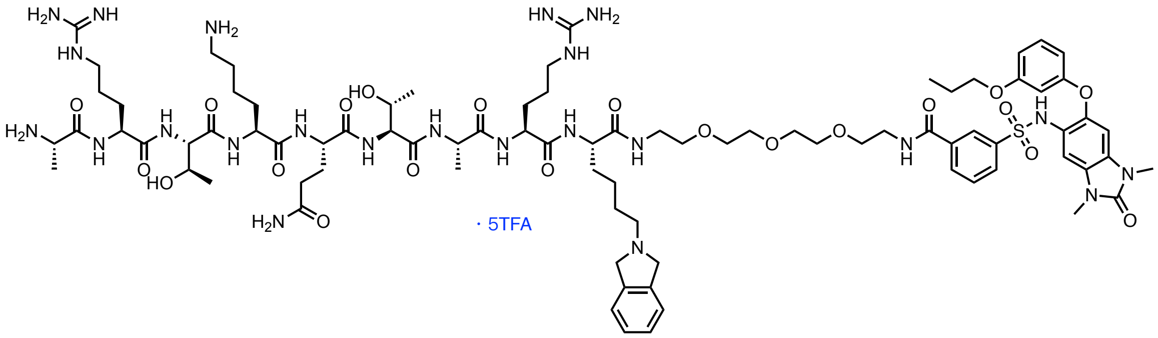
· 5TFA

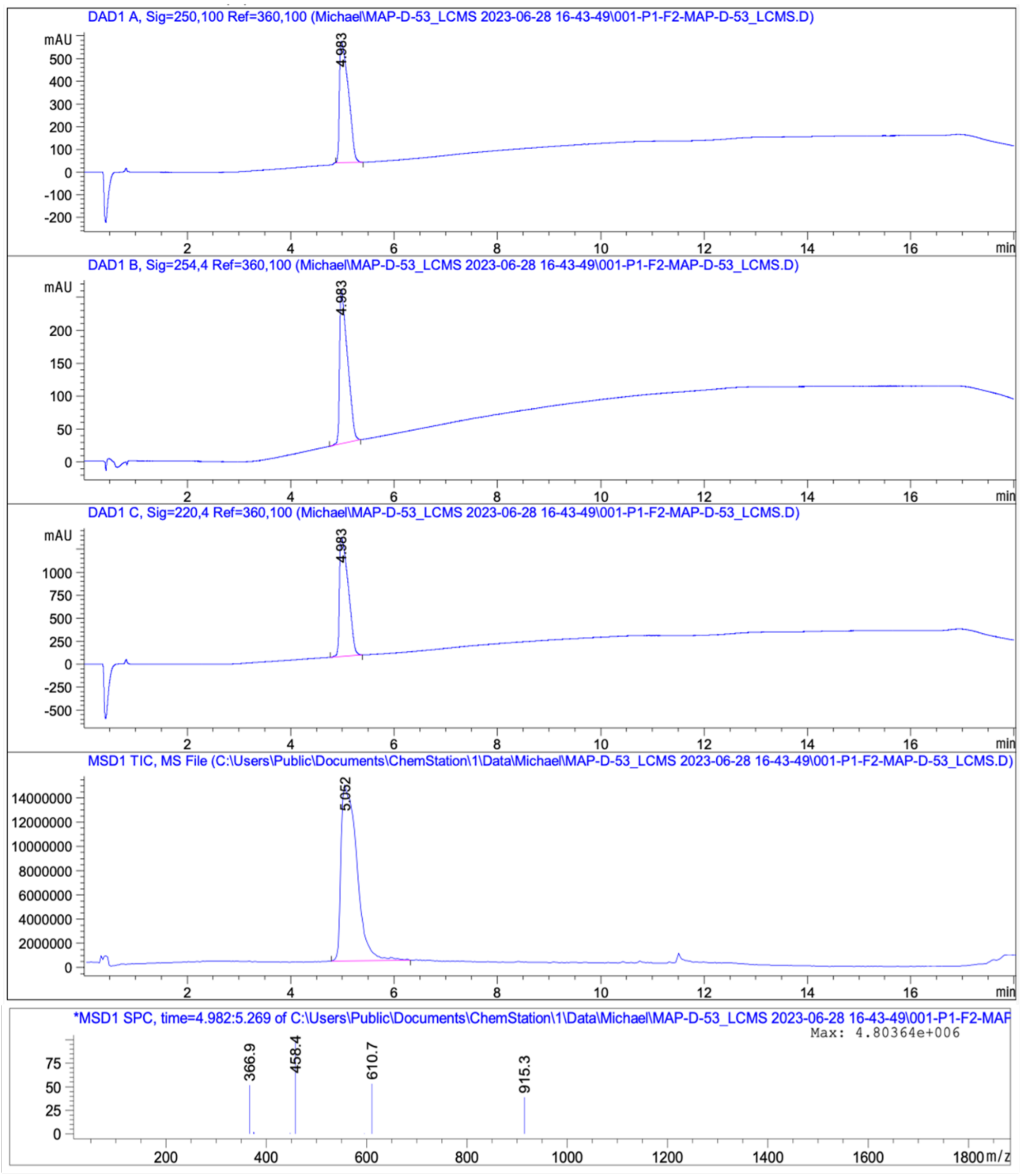
DAD1 A, Sig=250,100 Ref=360,100 (Michael\MAP-D-53_LCMS 2023-06-28 16-43-49\001-P1-F2-MAP-D-53_LCMS.D)
4.983
DAD1 B, Sig=254,4 Ref=360,100 (Michael\MAP-D-53_LCMS 2023-06-28 16-43-49\001-P1-F2-MAP-D-53_LCMS.D)
4.983
DAD1 C, Sig=220,4 Ref=360,100 (Michael\MAP-D-53_LCMS 2023-06-28 16-43-49\001-P1-F2-MAP-D-53_LCMS.D)
4.983
MSD1 TIC, MS File (C:\Users\Public\Documents\ChemStation\1\Data\Michael\MAP-D-53_LCMS 2023-06-28 16-43-49\001-P1-F2-MAP-D-53_LCMS.D)
5.052
*MSD1 SPC, time=4.982:5.269 of C:\Users\Public\Documents\ChemStation\1\Data\Michael\MAP-D-53_LCMS 2023-06-28 16-43-49\001-P1-F2-MAF
Max: 4.80364e+006
366.9
458.4
610.7
915.3
m/z
min

(52) PDC6

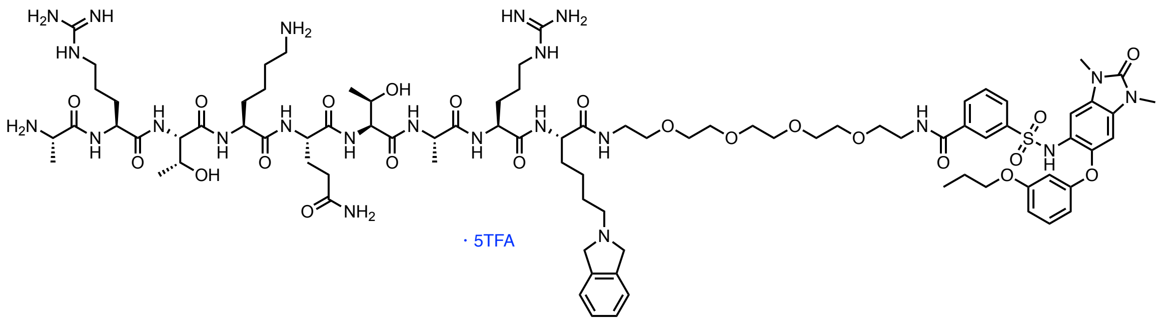
· 5TFA

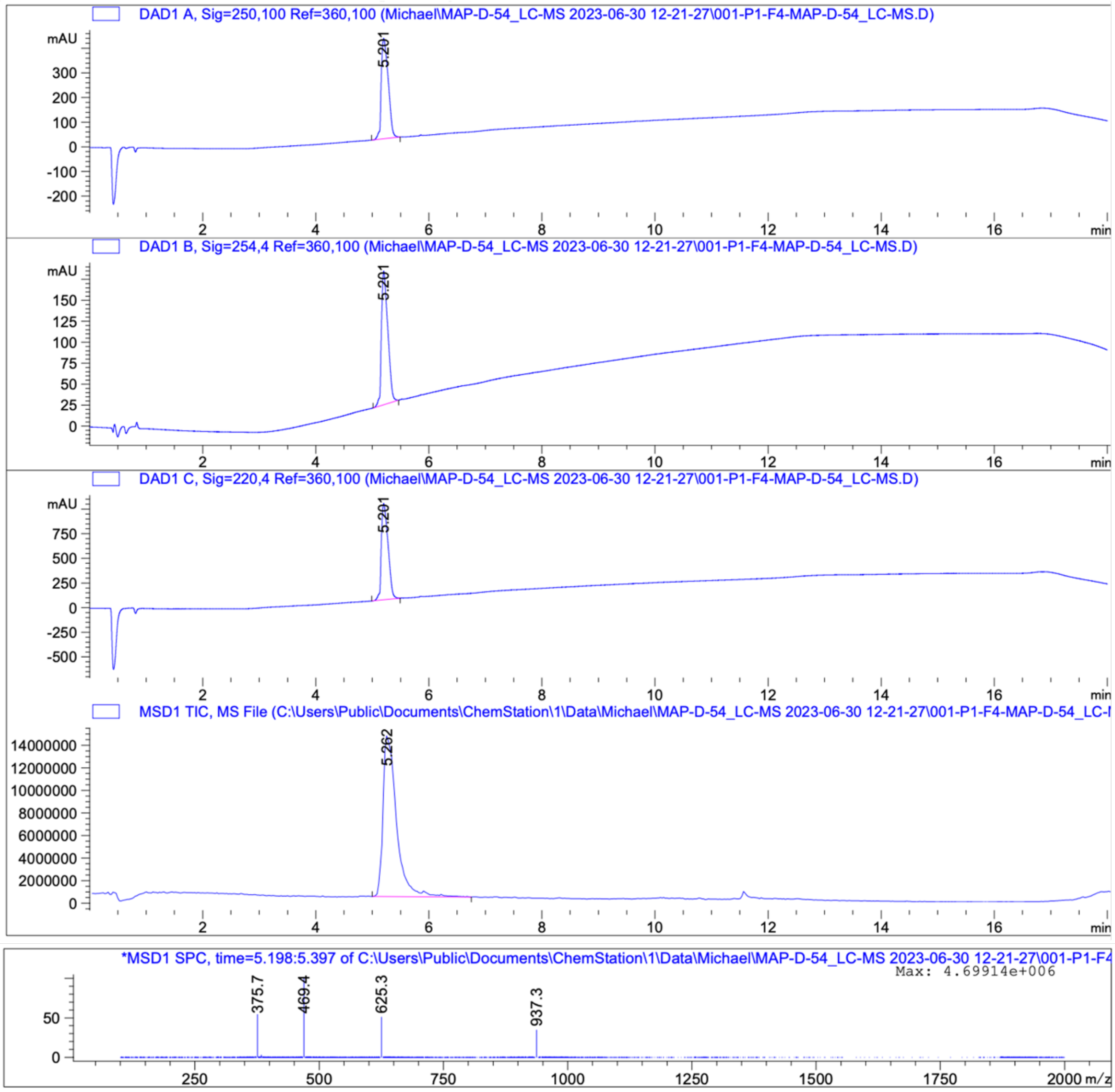
DAD1 A, Sig=250,100 Ref=360,100 (Michael\MAP-D-54_LC-MS 2023-06-30 12-21-27\001-P1-F4-MAP-D-54_LC-MS.D)
5.201
DAD1 B, Sig=254,4 Ref=360,100 (Michael\MAP-D-54_LC-MS 2023-06-30 12-21-27\001-P1-F4-MAP-D-54_LC-MS.D)
5.201
DAD1 C, Sig=220,4 Ref=360,100 (Michael\MAP-D-54_LC-MS 2023-06-30 12-21-27\001-P1-F4-MAP-D-54_LC-MS.D)
5.201
MSD1 TIC, MS File (C:\Users\Public\Documents\ChemStation\1\Data\Michael\MAP-D-54_LC-MS 2023-06-30 12-21-27\001-P1-F4-MAP-D-54_LC-
5.262
*MSD1 SPC, time=5.198:5.397 of C:\Users\Public\Documents\ChemStation\1\Data\Michael\MAP-D-54_LC-MS 2023-06-30 12-21-27\001-P1-F4
Max: 4.69914e+006
375.7
469.4
625.3
937.3

(56) PHD Peptide 3

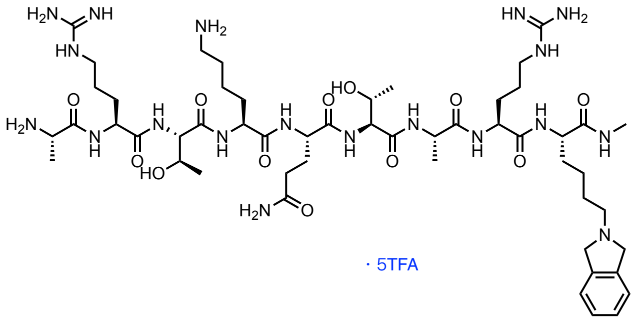

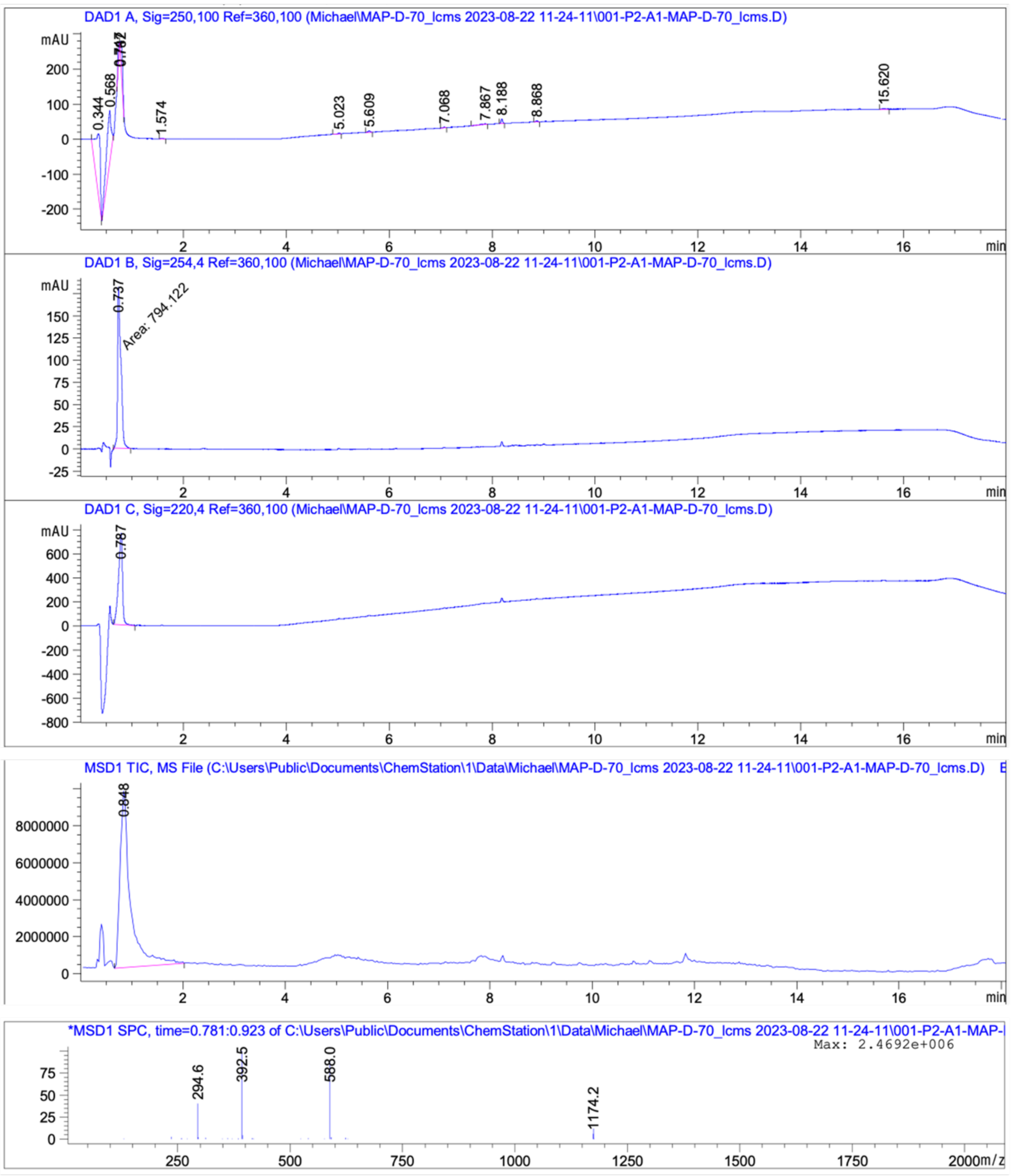

8 HPLC Chromatograms for P1–P16, 28, 32–34, 50–52, and 56

H3K9Me$_3$-mimicking Peptides P1–P16

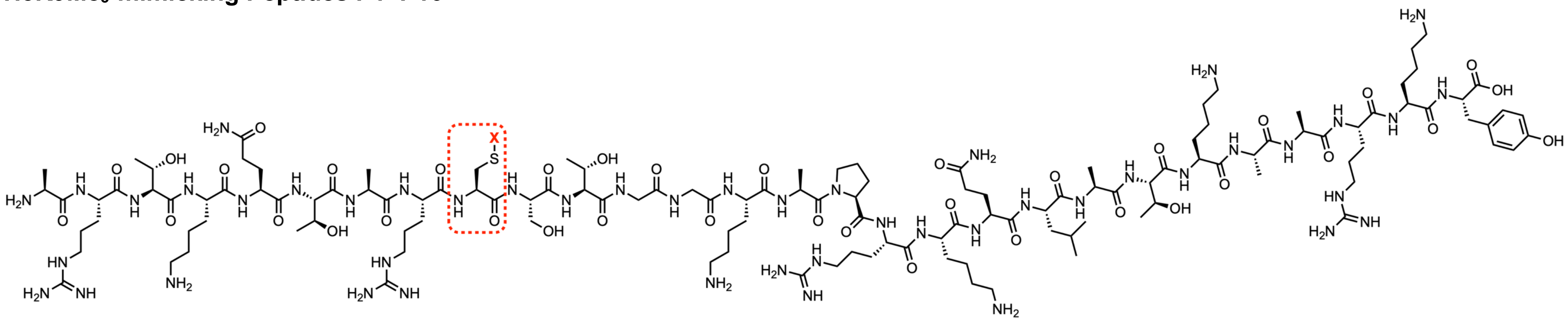

Amino Acid Sequence: ARTKQTARC(**X**)STGGKAPRKQLATKAARKY

Peptide	X
P1	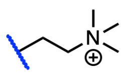
P2	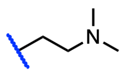
P3	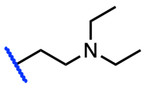
P4	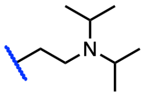

Peptide	X
P5	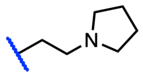
P6	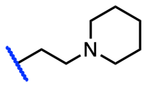
P7	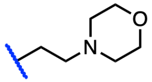
P8	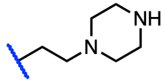

Peptide	X
P9	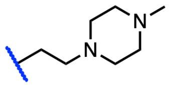
P10	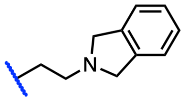
P11	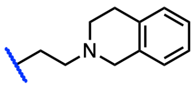
P12	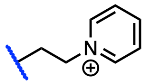

Peptide	X
P13	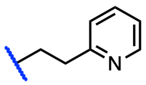
P14	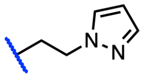
P15	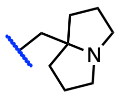
P16	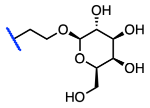

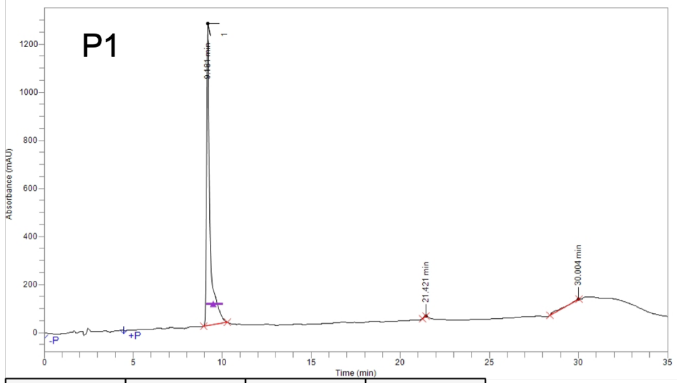

Time	Height	Area	Area %
9.181	1,256,947.2	17,650,054.2	98.29
21.421	980.9	21,301.1	0.12
30.004	819.4	286,544.4	1.60
Total		17,957,899.7	100.00

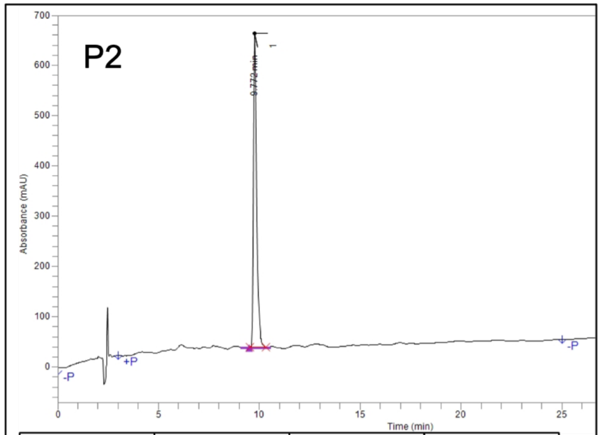

Time	Height	Area	Area %
9.772	624,807.8	6,420,549.1	100.00
Total		6,420,549.1	100.00

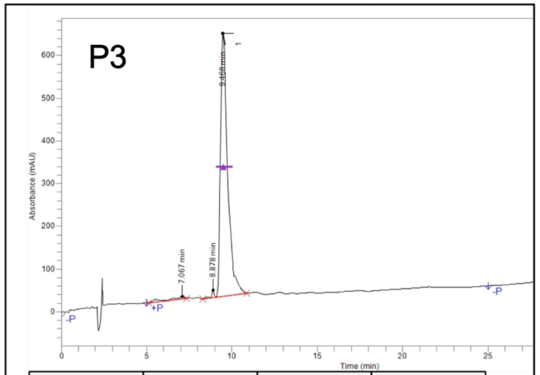

Time	Height	Area	Area %
5.541	4,921.1	158,407.6	0.86
7.067	4,626.2	154,943.2	0.85
8.878	17,456.6	167,914.8	0.92
9.468	615,970.3	17,841,867.1	97.37
Total		18,323,132.7	100.00

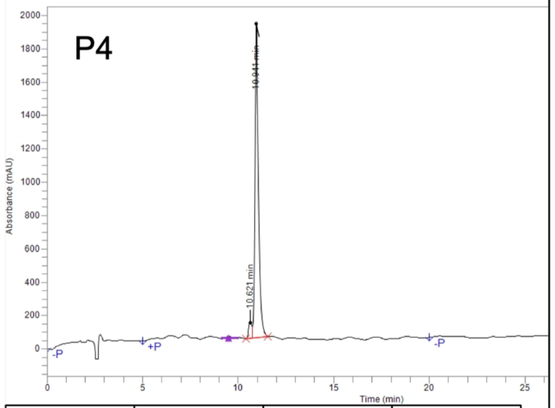

Time	Height	Area	Area %
10.621	95,430.1	1,114,232.6	4.36
10.941	1,883,944.9	24,458,460.5	95.64
Total		25,572,693.2	100.00

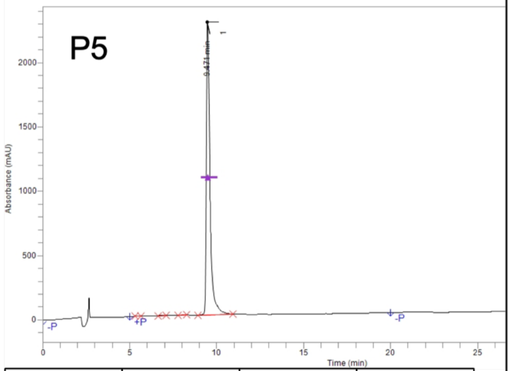

Time	Height	Area	Area %
5.590	547.7	12,495.1	0.04
7.035	649.2	24,076.0	0.07
8.224	597.9	24,534.9	0.08
9.471	2,280,215.8	32,391,649.9	99.81
Total		32,452,755.9	100.00

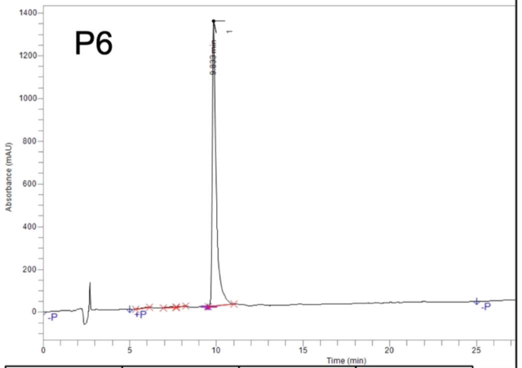

Time	Height	Area	Area %
6.106	693.5	47,083.5	0.24
7.434	3,265.7	82,077.9	0.41
8.184	496.2	23,849.3	0.12
9.833	1,336,644.0	19,837,195.9	99.23
Total		19,990,206.6	100.00

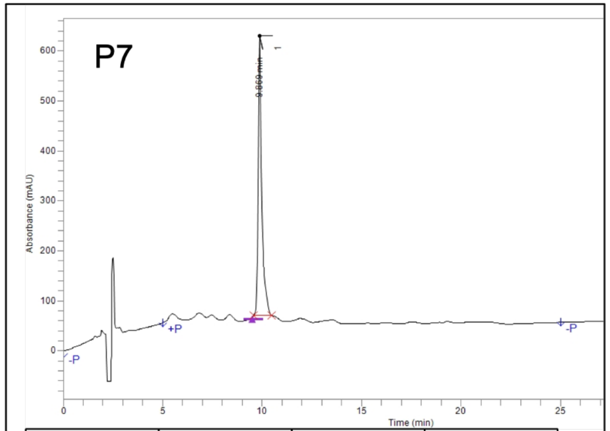

Time	Height	Area	Area %
9.869	560,452.2	6,591,956.1	100.00
**Total**		6,591,956.1	100.00

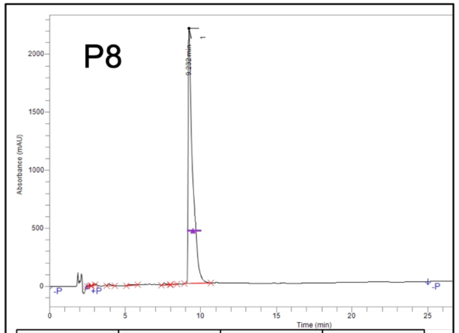

Time	Height	Area	Area %
2.672	9,039.5	52,107.3	0.11
2.940	11,324.4	60,222.9	0.13
3.874	13,471.5	198,045.0	0.43
5.228	7,737.0	182,846.8	0.40
7.576	16,818.4	220,087.2	0.48
8.282	5,316.0	49,451.8	0.11
9.232	2,201,790.5	45,419,643.5	98.35
**Total**		46,182,404.5	100.00

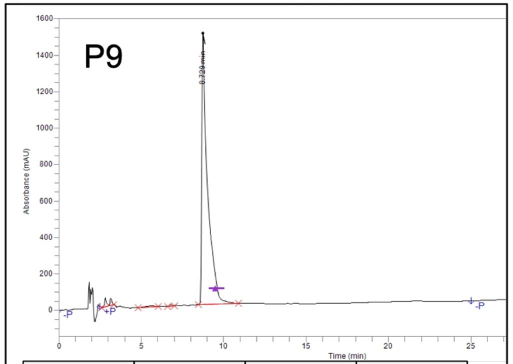

Time	Height	Area	Area %
2.816	47,132.1	358,806.4	0.93
3.156	35,299.4	287,543.7	0.75
5.650	6,711.2	258,025.8	0.67
6.803	10,701.6	91,811.1	0.24
8.729	1,490,732.7	37,490,307.3	97.41
**Total**		38,486,494.2	100.00

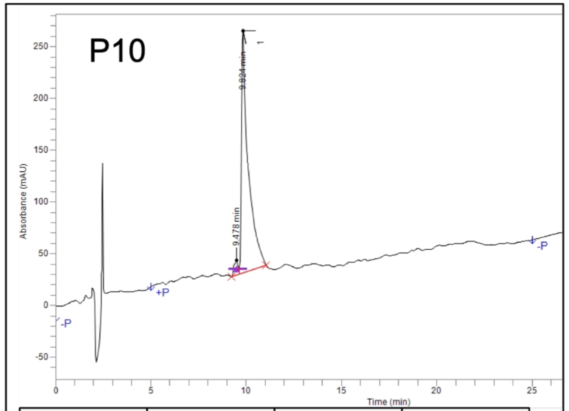

Time	Height	Area	Area %
9.478	14,476.5	202,959.9	3.50
9.824	234,152.7	5,594,026.3	96.50
**Total**		5,796,986.2	100.00

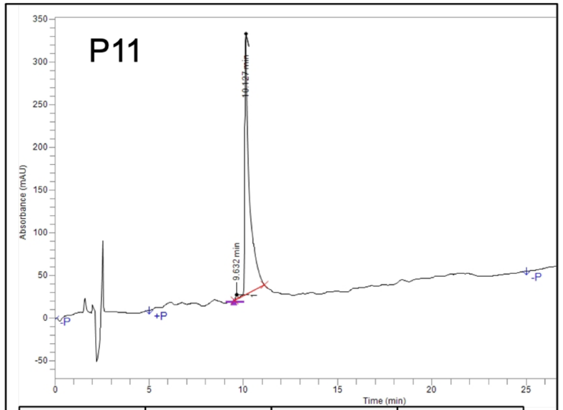

Time	Height	Area	Area %
9.632	6,125.8	43,353.7	0.83
10.127	305,888.9	5,157,467.5	99.17
**Total**		5,200,821.1	100.00

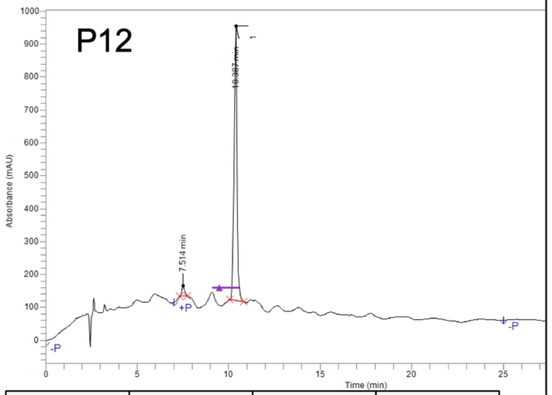

Time	Height	Area	Area %
7.514	31,089.7	361,834.1	4.05
10.387	835,331.8	8,565,068.8	95.95
**Total**		8,926,902.9	100.00

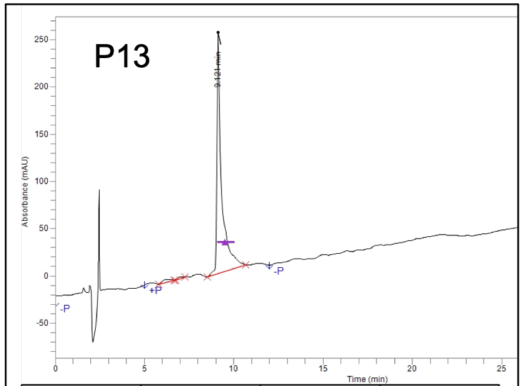

Time	Height	Area	Area %
6.456	1,963.3	88,594.8	1.75
7.032	1,621.6	27,938.1	0.55
9.121	255,656.3	4,939,819.9	97.70
**Total**		5,056,352.9	100.00

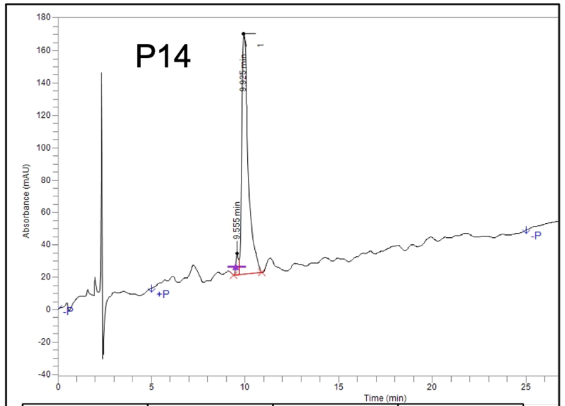

Time	Height	Area	Area %
9.555	13,332.1	135,959.7	3.67
9.925	148,701.8	3,568,829.3	96.33
**Total**		3,704,788.9	100.00

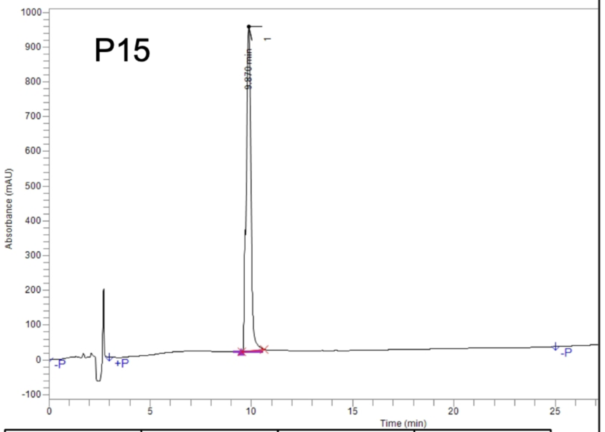

Time	Height	Area	Area %
9.870	934,087.5	14,600,122.2	100.00
**Total**		14,600,122.2	100.00

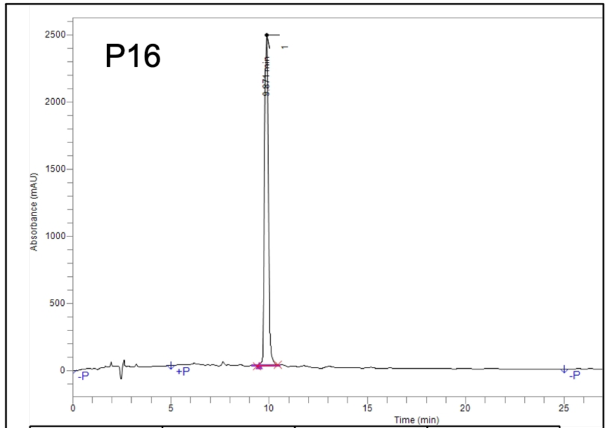

Time	Height	Area	Area %
9.871	2,462,217.8	31,848,986.0	100.00
**Total**		31,848,986.0	100.00

(28) 3-(*N*-(1,3-Dimethyl-2-oxo-6-(3-propoxyphenoxy)-2,3-dihydro-1*H*-benzo[*d*]imidazol-5-yl)sulfamoyl)benzoic acid

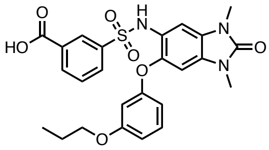

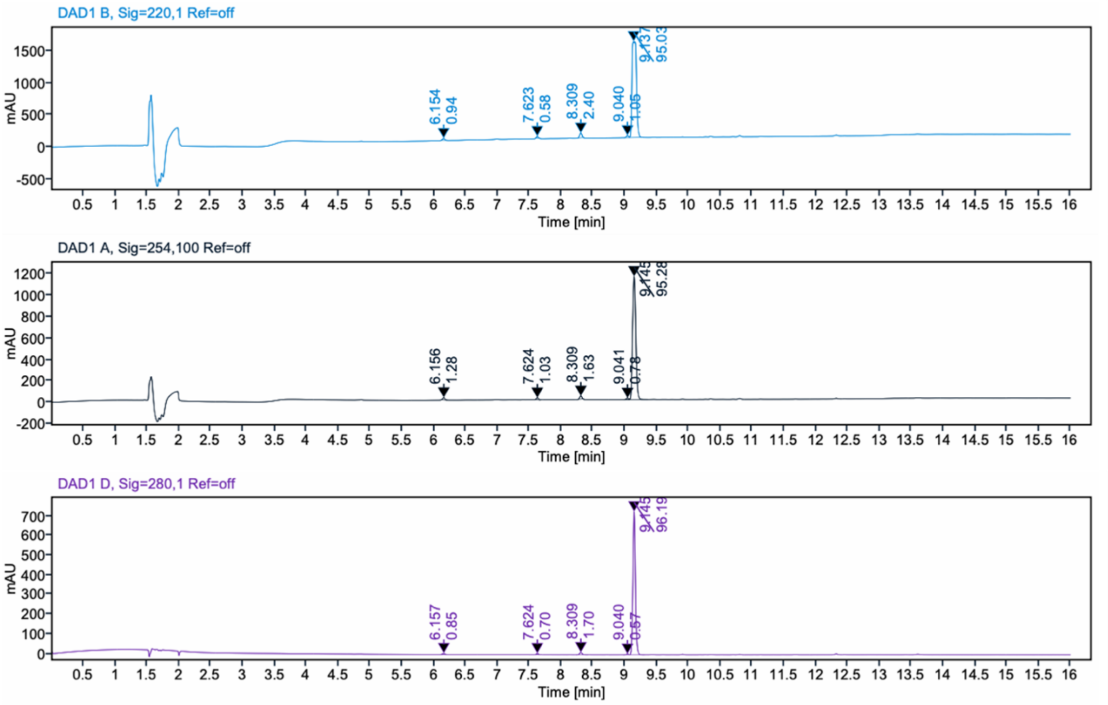

Signal: DAD1 B, Sig=220,1 Ref=off

RT [min]	Type	Width [min]	Area	Height	Area%
6.154	MM	0.0406	70.7560	29.0731	0.9388
7.623	MM	0.0285	44.0723	25.7582	0.5847
8.309	MM	0.0412	180.7276	73.1441	2.3979
9.040	MF	0.0412	78.9560	31.9489	1.0476
9.137	FM	0.0804	7162.4551	1484.1761	95.0310
		**Sum**	7536.9670		

Signal: DAD1 A, Sig=254,100 Ref=off

RT [min]	Type	Width [min]	Area	Height	Area%
6.156	MM	0.0437	53.6648	20.4808	1.2820
7.624	MM	0.0423	42.9388	16.9129	1.0257
8.309	MM	0.0395	68.3300	28.8032	1.6323
9.041	BV E	0.0384	32.6434	13.1872	0.7798
9.145	VB R	0.0577	3988.5232	1142.4351	95.2802
		**Sum**	4186.1003		

Signal: DAD1 D, Sig=280,1 Ref=off

RT [min]	Type	Width [min]	Area	Height	Area%
6.157	MM	0.0486	17.3865	5.9681	0.8485
7.624	MM	0.0377	14.3446	6.3383	0.7001
8.309	BB	0.0445	34.8025	11.9448	1.6985
9.040	MF	0.0425	11.5964	4.5505	0.5660
9.145	FM	0.0452	1970.8463	727.3156	96.1869
		**Sum**	2048.9763		

(32) 3-(*N*-(1,3-Dimethyl-2-oxo-6-(3-propoxyphenoxy)-2,3-dihydro-1*H*-benzo[*d*]imidazol-5-yl)sulfamoyl)-*N*-(2-oxo-6,9-dioxa-3-azaundecan-11-yl)benzamide

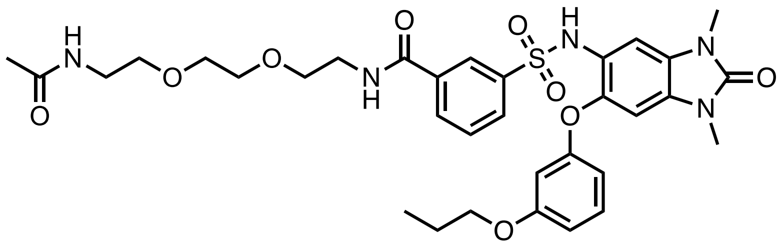

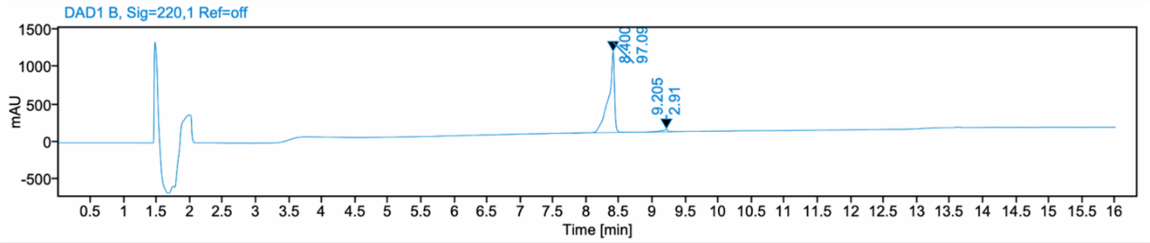

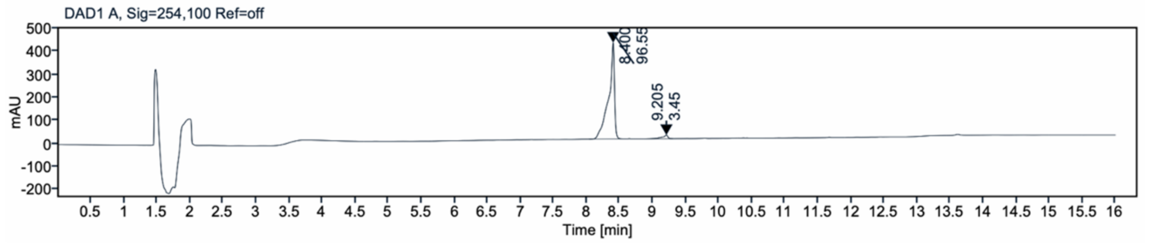

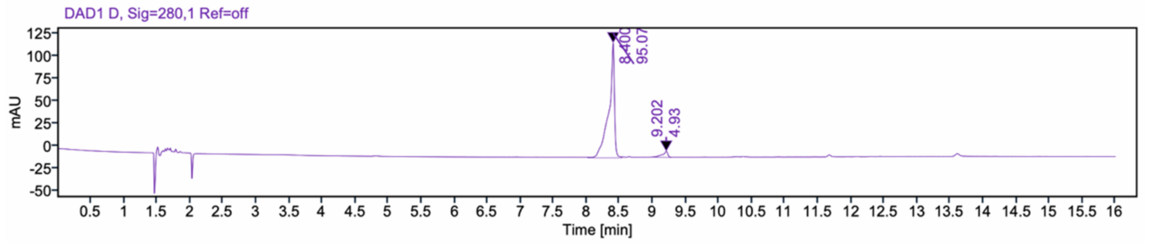

Signal: DAD1 B, Sig=220,1 Ref=off

RT [min]	Type	Width [min]	Area	Height	Area%
8.400	VB R	0.0845	6792.0425	1070.2261	97.0871
9.205	BB	0.0803	203.7801	32.6387	2.9129
		**Sum**	6995.8226		

Signal: DAD1 A, Sig=254,100 Ref=off

RT [min]	Type	Width [min]	Area	Height	Area%
8.400	BB	0.0827	2586.3210	417.8310	96.5452
9.205	BB	0.0807	92.5486	15.4814	3.4548
		**Sum**	2678.8696		

Signal: DAD1 D, Sig=280,1 Ref=off

RT [min]	Type	Width [min]	Area	Height	Area%
8.400	MM	0.0995	765.6229	128.2305	95.0712
9.202	MM	0.0974	39.6920	6.7944	4.9288
		**Sum**	805.3148		

(33) 3-(*N*-(1,3-Dimethyl-2-oxo-6-(3-propoxyphenoxy)-2,3-dihydro-1*H*-benzo[*d*]imidazol-5-yl)sulfamoyl)-*N*-(2-oxo-6,9,12-trioxa-3-azatetradecan-14-yl)benzamide

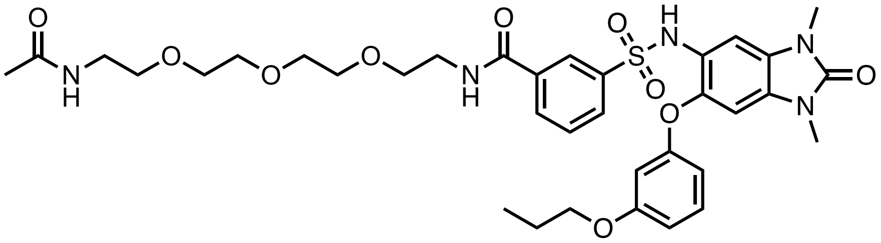

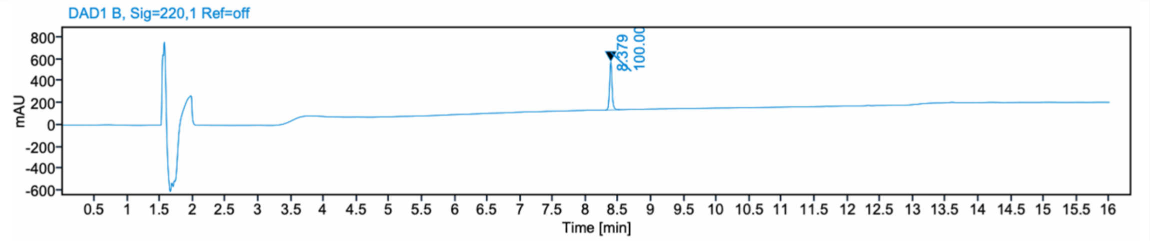

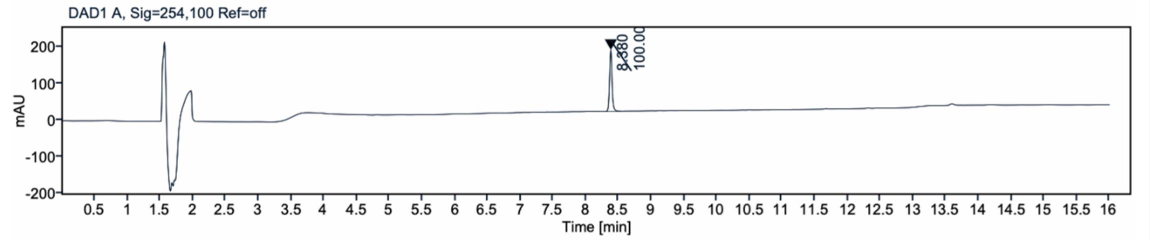

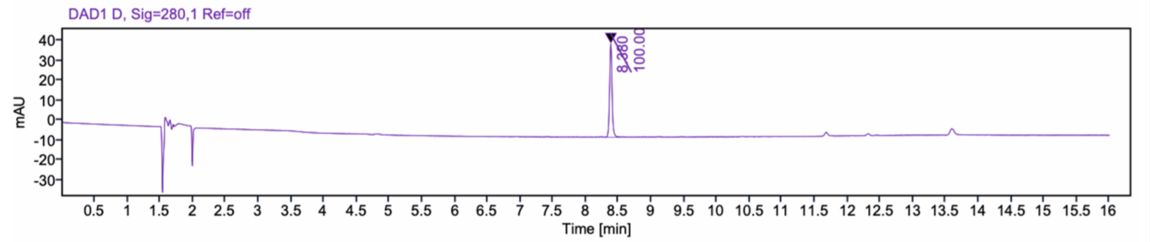

Signal: DAD1 B, Sig=220,1 Ref=off

RT [min]	Type	Width [min]	Area	Height	Area%
8.379	BB	0.0403	1159.1935	438.8969	100.0000
		**Sum**	1159.1935		

Signal: DAD1 A, Sig=254,100 Ref=off

RT [min]	Type	Width [min]	Area	Height	Area%
8.380	BB	0.0403	441.4357	167.2781	100.0000
		**Sum**	441.4357		

Signal: DAD1 D, Sig=280,1 Ref=off

RT [min]	Type	Width [min]	Area	Height	Area%
8.380	BB	0.0406	123.2187	46.9504	100.0000
		**Sum**	123.2187		

(34) 3-(*N*-(1,3-Dimethyl-2-oxo-6-(3-propoxyphenoxy)-2,3-dihydro-1*H*-benzo[*d*]imidazol-5-yl)sulfamoyl)-*N*-(2-oxo-6,9,12,15-tetraoxa-3-azaheptadecan-17-yl)benzamide

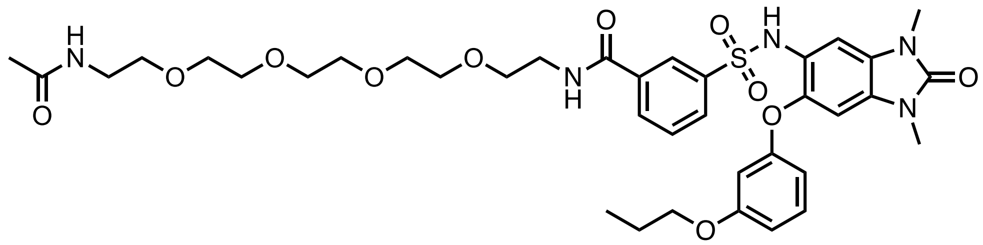

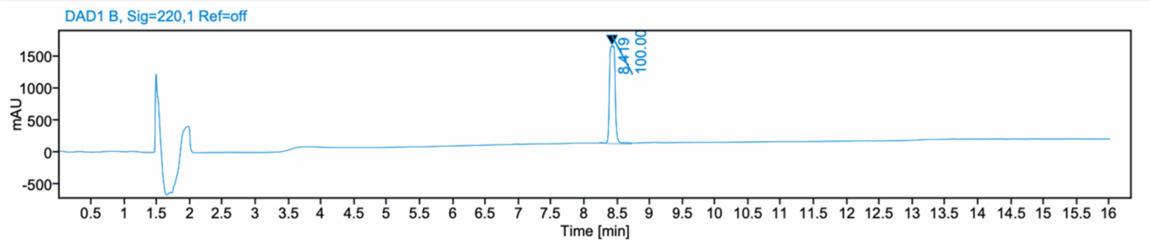

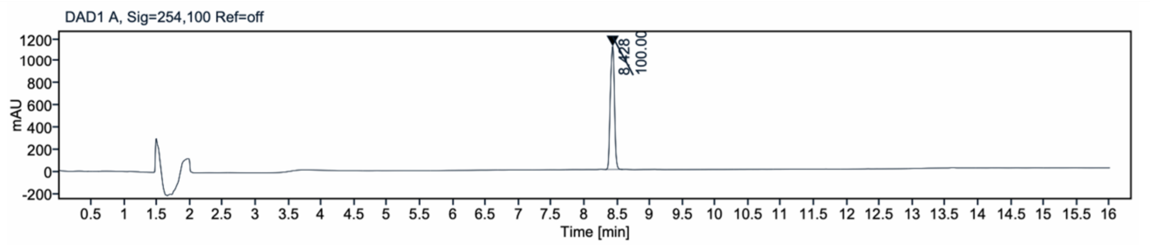

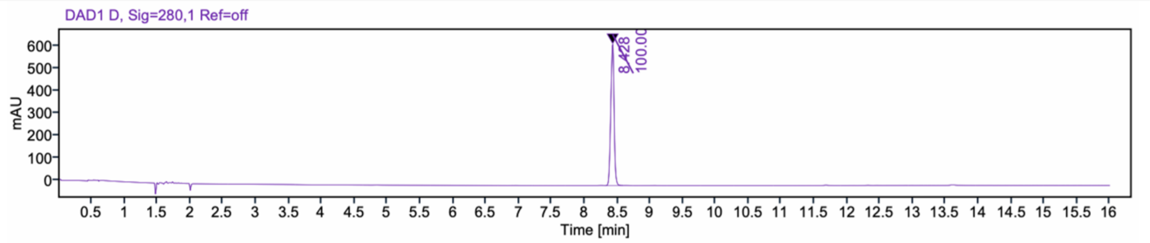

Signal: DAD1 B, Sig=220,1 Ref=off

RT [min]	Type	Width [min]	Area	Height	Area%
8.419	MM	0.0999	9196.1250	1534.9658	100.0000
		**Sum**	9196.1250		

Signal: DAD1 A, Sig=254,100 Ref=off

RT [min]	Type	Width [min]	Area	Height	Area%
8.428	BB	0.0740	4885.5239	1116.9390	100.0000
		**Sum**	4885.5239		

Signal: DAD1 D, Sig=280,1 Ref=off

RT [min]	Type	Width [min]	Area	Height	Area%
8.428	BB	0.0560	2204.2925	626.8787	100.0000
		**Sum**	2204.2925		

(50) PDC4

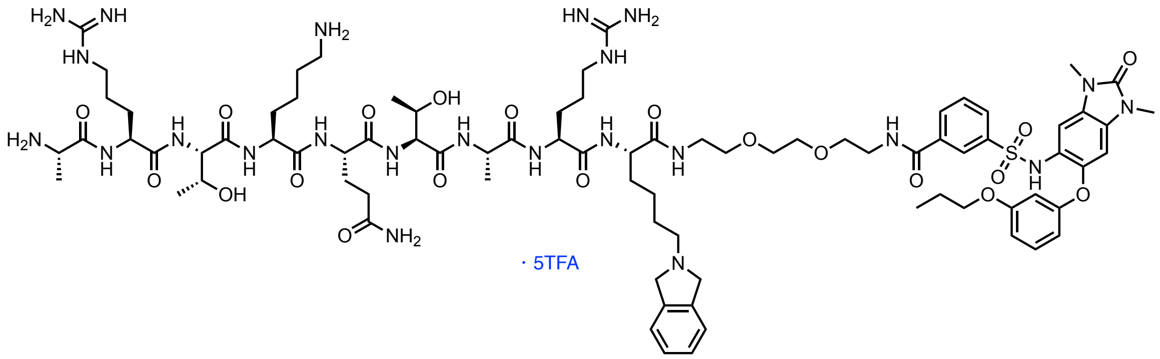

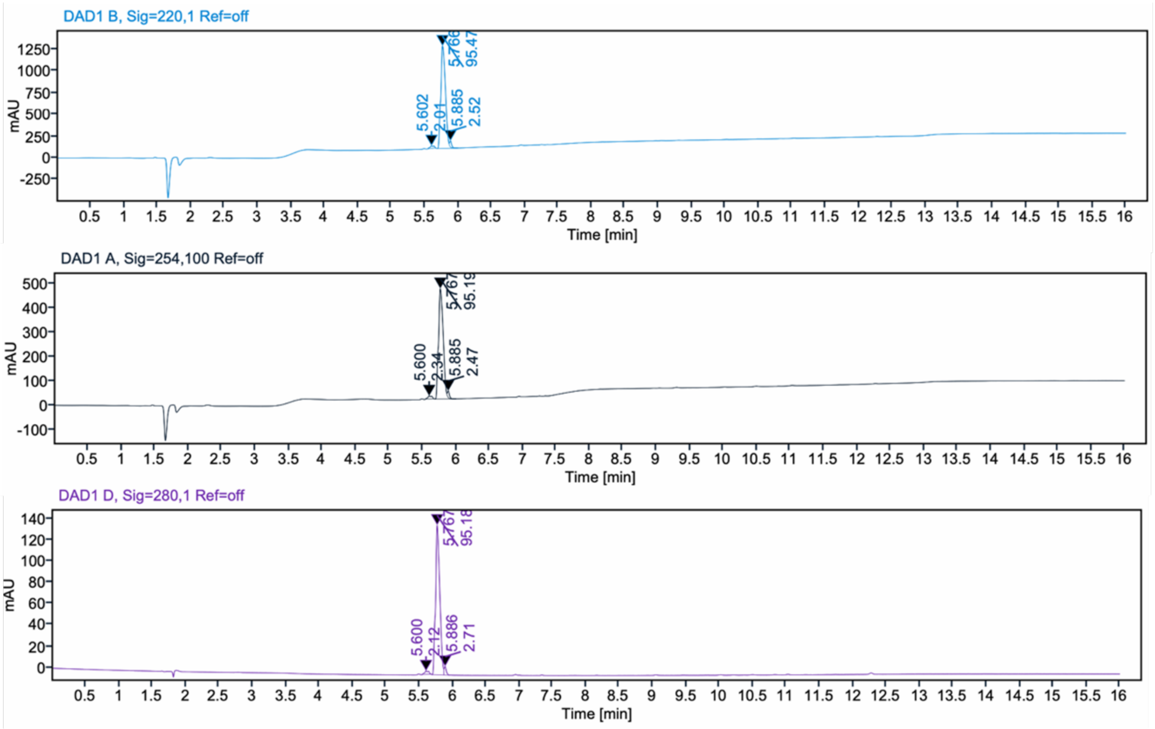

Signal: DAD1 B, Sig=220,1 Ref=off

RT [min]	Type	Width [min]	Area	Height	Area%
5.602	MM	0.0657	120.3803	30.5181	2.0114
5.766	BV R	0.0643	5713.6802	1173.7549	95.4690
5.885	VV E	0.0312	150.7956	69.7947	2.5196
		**Sum**	5984.8561		

Signal: DAD1 A, Sig=254,100 Ref=off

RT [min]	Type	Width [min]	Area	Height	Area%
5.600	MM	0.0691	54.6659	13.1805	2.3436
5.767	BV R	0.0715	2220.3271	454.4994	95.1893
5.885	VB E	0.0310	57.5465	27.4085	2.4671
		**Sum**	2332.5395		

Signal: DAD1 D, Sig=280,1 Ref=off

RT [min]	Type	Width [min]	Area	Height	Area%
5.600	MM	0.0695	13.8088	3.3111	2.1174
5.767	MF	0.0737	620.7062	140.3202	95.1756
5.886	FM	0.0363	17.6545	8.1163	2.7070
		**Sum**	652.1695		

(51) PDC5

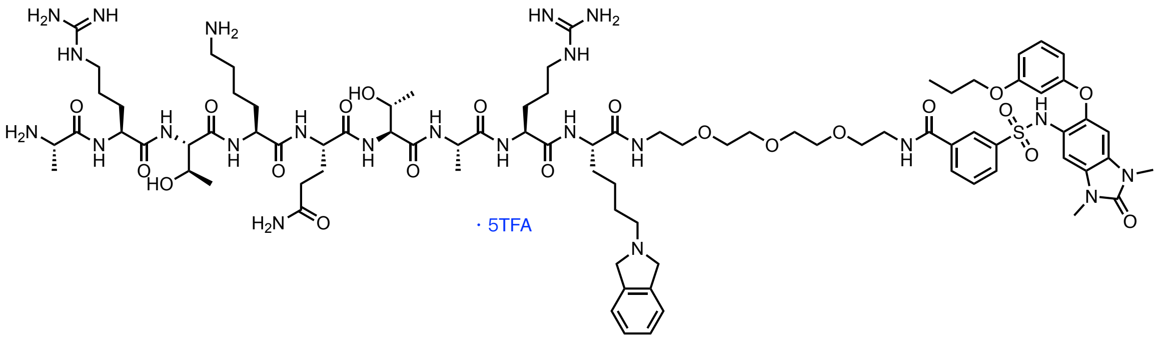

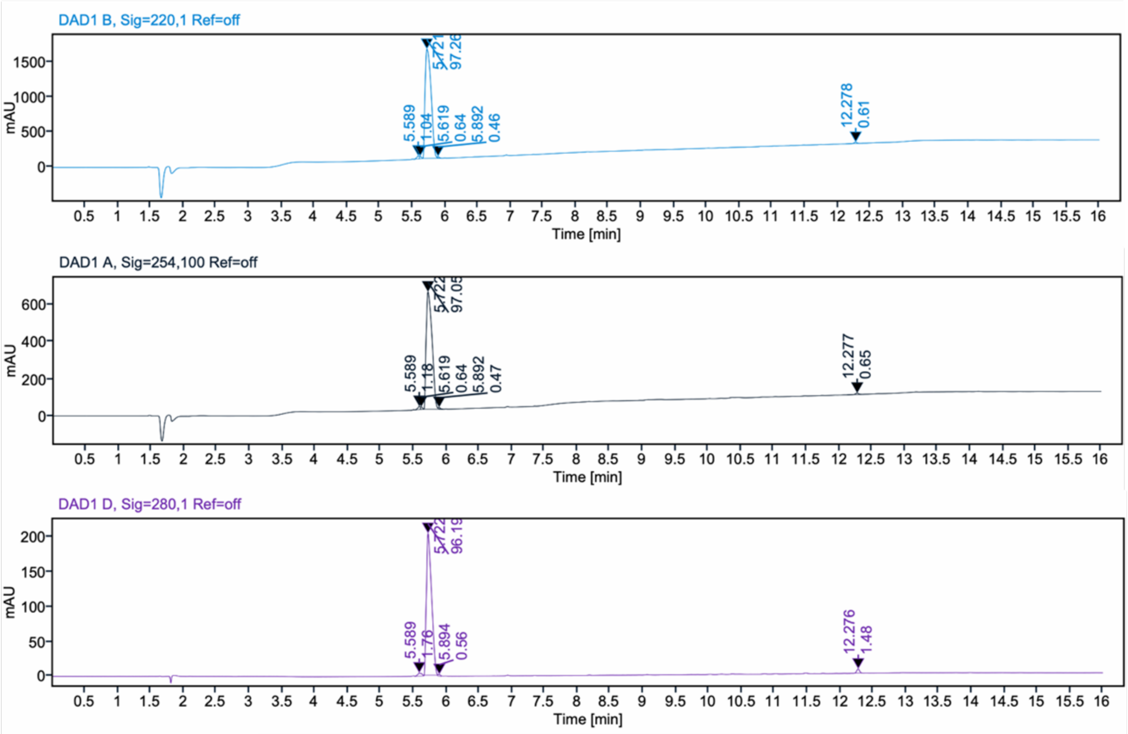

Signal:	DAD1 B, Sig=220,1 Ref=off				
RT [min]	Type	Width [min]	Area	Height	Area%
5.589	BV	0.0345	105.0308	43.7189	1.0352
5.619	VB	0.0280	64.4301	34.9734	0.6350
5.721	BV R	0.0865	9867.9688	1546.1902	97.2595
5.892	VB E	0.0307	47.1364	22.2657	0.4646
12.278	BB	0.0438	61.4499	18.2534	0.6057
		Sum	10146.0158		

Signal:	DAD1 A, Sig=254,100 Ref=off				
RT [min]	Type	Width [min]	Area	Height	Area%
5.589	BV	0.0370	47.9056	18.0283	1.1808
5.619	VB	0.0277	26.0846	14.3393	0.6430
5.722	BV R	0.0843	3937.4248	635.6541	97.0543
5.892	VB E	0.0305	19.1095	9.0831	0.4710
12.277	BB	0.0485	26.4047	7.9953	0.6509

Signal:	DAD1 D, Sig=280,1 Ref=off				
RT [min]	Type	Width [min]	Area	Height	Area%
5.589	MM	0.0721	20.2840	4.6920	1.7611
5.722	MM	0.0911	1107.9709	202.6207	96.1943
5.894	MM	0.0413	6.4897	2.6173	0.5634
12.276	MM	0.0482	17.0600	5.8986	1.4812
		Sum	1151.8047		

(52) PDC6

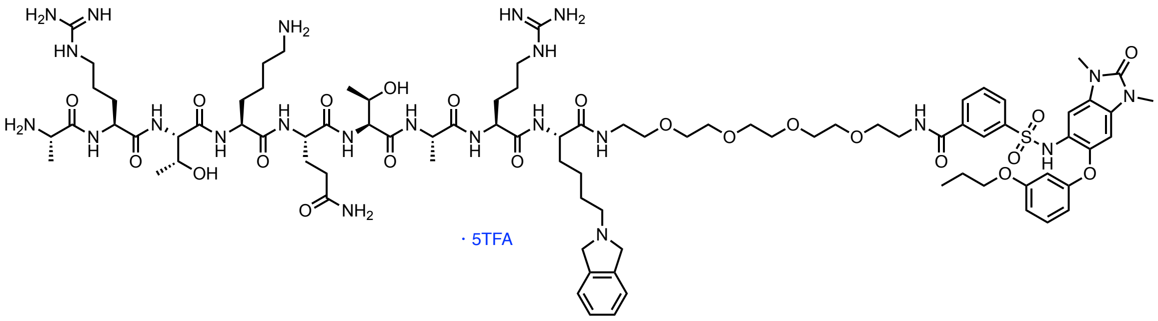

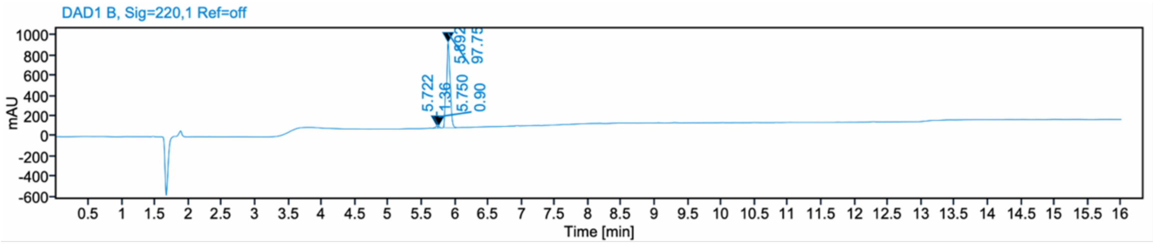

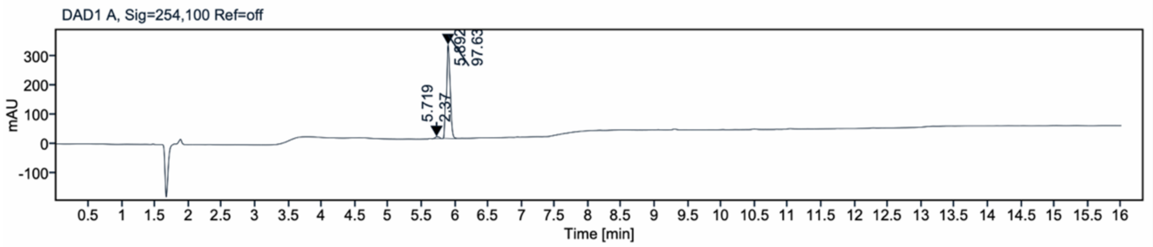

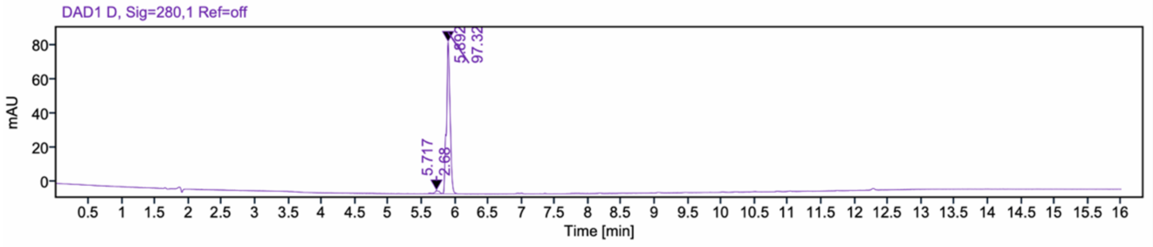

Signal: DAD1 B, Sig=220,1 Ref=off

RT [min]	Type	Width [min]	Area	Height	Area%
5.722	BV	0.0302	45.4820	18.4662	1.3582
5.750	VB	0.0244	30.0065	15.3082	0.8961
5.892	MM	0.0650	3273.1433	838.9249	97.7457
		**Sum**	3348.6317		

Signal: DAD1 A, Sig=254,100 Ref=off

RT [min]	Type	Width [min]	Area	Height	Area%
5.719	BV	0.0561	31.2783	7.5136	2.3734
5.892	MM	0.0661	1286.6101	324.3882	97.6266
		**Sum**	1317.8884		

Signal: DAD1 D, Sig=280,1 Ref=off

RT [min]	Type	Width [min]	Area	Height	Area%
5.717	MM	0.0789	9.2368	1.9501	2.6835
5.892	MM	0.0628	334.9674	88.9557	97.3165
		**Sum**	344.2042		

(56) PHD Peptide 3

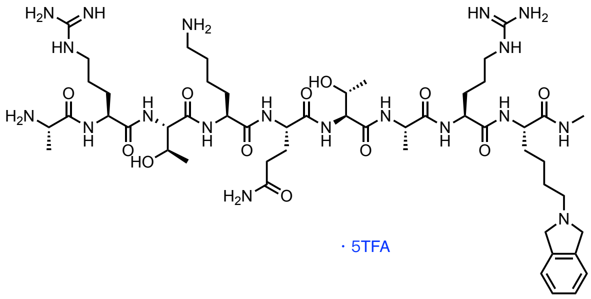

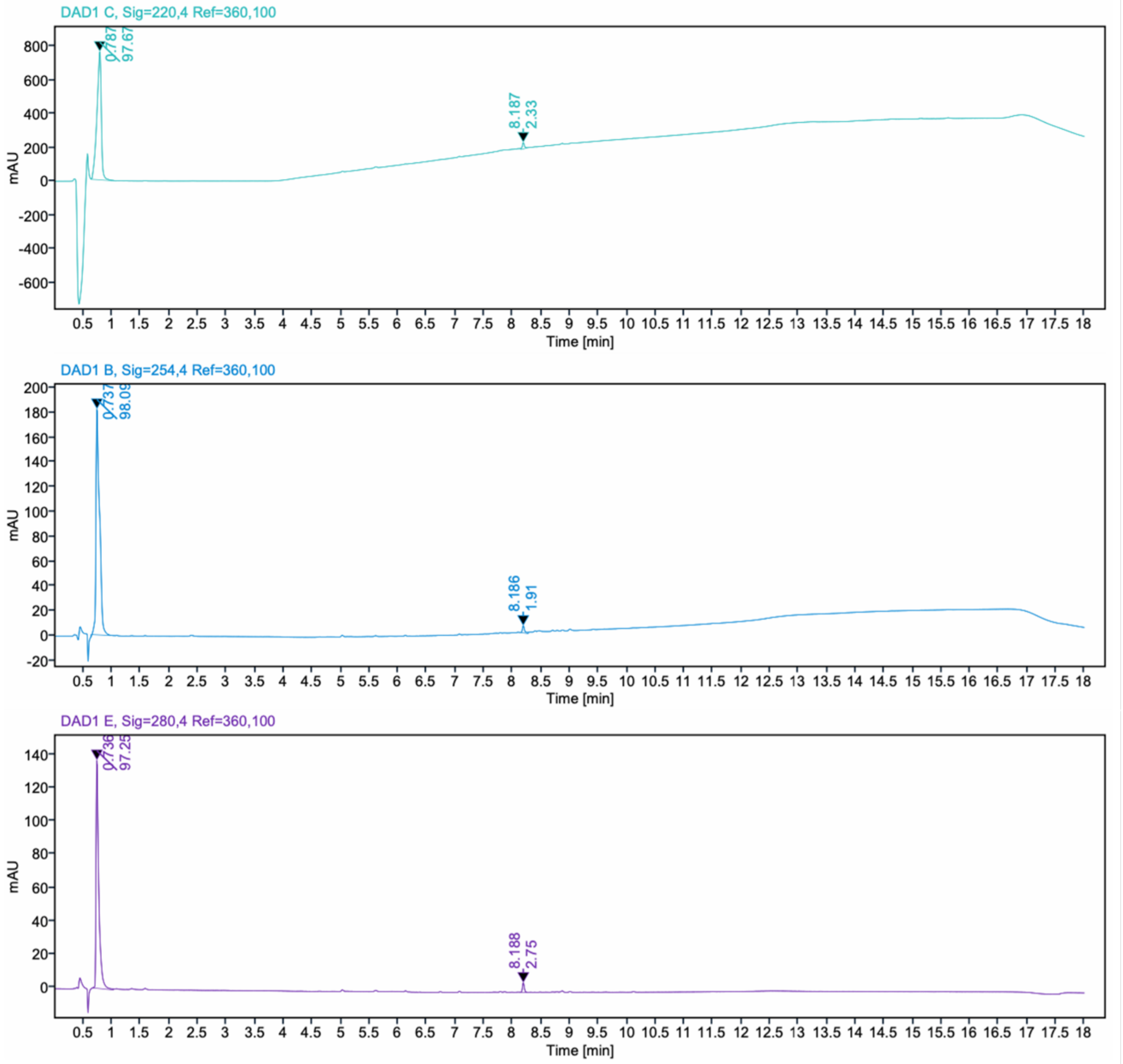

Signal: DAD1 C, Sig=220,4 Ref=360,100

RT [min]	Type	Width [min]	Area	Height	Area%
0.787	BB	0.0757	4056.1399	761.1058	97.6717
8.187	MM	0.0429	96.6887	37.5530	2.3283
		**Sum**	4152.8286		

Signal: DAD1 B, Sig=254,4 Ref=360,100

RT [min]	Type	Width [min]	Area	Height	Area%
0.737	MM	0.0727	794.1224	181.9515	98.0888
8.186	MM	0.0455	15.4734	5.6685	1.9112
		**Sum**	809.5958		

Signal: DAD1 E, Sig=280,4 Ref=360,100

RT [min]	Type	Width [min]	Area	Height	Area%
0.736	MM	0.0556	456.8456	136.9389	97.2533
8.188	MM	0.0371	12.9026	5.7904	2.7467
		**Sum**	469.7482		

SI References

[1] W. S. Palmer, G. Poncet-Montange, G. Liu, A. Petrocchi, N. Reyna, G. Subramanian, J. Theroff, A. Yau, M. Kost-Alimova, J. P. Bardenhagen, E. Leo, H. E. Shepard, T. N. Tieu, X. Shi, Y. Zhan, S. Zhao, M. C. Barton, G. Draetta, C. Toniatti, P. Jones, M. Geck-Do, and J. N. Andersen, "Structure-Guided Design of IACS-9571, a Selective High-Affinity Dual TRIM24-BRPF1 Bromodomain Inhibitor" *J. Med. Chem.* **2016**, *59*, 1440–1454.

[2] J. Bennett, O. Fedorov, C. Tallant, O. Monteiro, J. Meier, V. Gamble, P. Savitsky, G. A. Nunez-Alonso, B. Haendler, C. Rogers, P. E. Brennan, S. Müller, and S. Knapp, "Discovery of a Chemical Tool Inhibitor Targeting the Bromodomains of TRIM24 and BRPF" *J. Med. Chem.* **2016**, *59*, 1642–1647.

[3] Q. Hu, C. Wang, Q. Xiang, R. Wang, C. Zhang, M. Zhang, X. Xue, G. Luo, X. Liu, X. Wu, Y. Zhang, D. Wu, Y. Xu, "Discovery and Optimization of Novel N-benzyl-3,6-dimethylbenzo[d]isoxazol-5-amine Derivatives as Potent and Selective TRIM24 Bromodomain Inhibitors with Potential Anti-Cancer Activities" *Bioorg. Chem.* **2020**, *94*, 103424.

[4] Q. Xiang, G. Luo, C. Zhang, Q. Hu, C. Wang, T. Wu, H. Xu, J. Hu, X. Zhuang, M. Zhang, S. Wu, J. Xu, Y. Zhang, J. Liu, Y. Xu, "Discovery, Optimization and Evaluation of 1-(Indolin-1-yl)ethan-1-ones as Novel Selective TRIM24/BRPF1 Bromodomain Inhibitors" *Eur. J. Med. Chem.* **2022**, *236*, 114311.

[5] L. N. Gechijian, D. L. Buckley, M. A. Lawlor, J. M. Reyes, J. Paulk, C. J. Ott, G. E. Winter, M. A. Erb, T. G. Scott, M. Xu, H. S. Seo, S. Dhe-Paganon, N. P. Kwiatkowski, J. A. Perry, J. Qi, N. S. Gray, J. E. Bradner, "Functional TRIM24 Degrader via Conjugation of Ineffectual Bromodomain and VHL Ligands" *Nat. Chem. Biol.* **2018**, *14*, 405–412.

[6] A. Vezzoli, N. Bonadies, M. D. Allen, S. M. V. Freund, C. M. Santiveri, B. T. Kvinlaug, B. J. P. Huntly, B. Göttgens, M. Bycroft, "Molecular Basis of Histone H3K36me3 Recognition by the PWWP Domain of BRPF1" *Nat. Struct. Mol. Biol.* **2010**, *17*, 617–619.

[7] B. J. Klein, K. L. Cox, S. M. Jang, J. Côté, M. G. Poirier, T. G. Kutateladze, "Molecular Basis for the PZP Domain of BRPF1 Association with Chromatin" *Struct.* **2020**, *28*, 105–110.

[8] J. O. Obi, M. Y. Lubula, G. Cornilescu, A. Henrickson, K. McGuire, C. M. Evans, M. Phillips, S. P. Boyson, B. Demeler, J. L. Markley, K. C. Glass, “The BRPF1 Bromodomain is a Molecular Reader of Di-Acetyllysine” *Curr. Res. Struct. Biol.* **2020**, *2*, 104–115.

[9] A. R. Sekirnik, J. K. Reynolds, L. See, J. P. Bluck, A. R. Scorah, C. Tallant, B. Lee, K. B. Leszczynska, R. L. Grimley, R. I. Storer, M. Malattia, S. Crespillo, S. Caria, S. Duclos, E. M. Hammond, S. Knapp, G. M. Morris, F. Duarte, P. C. Biggin, S. J. Conway, “Identification of Histone Peptide Binding Specificity and Small-Molecule Ligands for the TRIM33α and TRIM33β Bromodomains” *ACS Chem. Biol.* **2022**, *17*, 2753–2768.

[10] X. Pan, H. Wang, C. Li, J. Z. H. Zhang, C. Ji, “MolGpka: A Web Server for Small Molecule pKa Prediction Using a Graph-Convolutional Neural Network” *J. Chem. Inf. Model.* **2021**, *61*, 3159–3165.

[11] W. S. Palmer, P. Jones, G. Liu, A. Petrocchi, N. Reyna, G. Subrumanian, J. Theroff, A. Yau (University of Texas), US2016060260A1, **2016**.

[12] J. K. Reynolds, Ph.D. Thesis, University of Oxford (U.K.), **2020**.

[13] B. J. G. E. Pieters, J. C. J. Hintzen, Y. Grobben, A. H. K. Al Temimi, J. J. A. G. Kamps, J. Mecinović, “Installation of Trimethyllysine Analogs on Intact Histones via Cysteine Alkylation” *Bioconjug. Chem.* **2019**, *30*, 952–958.

[14] M. D. Simon, K. M. Shokat, “A Method to Site-Specifically Incorporate Methyl-Lysine Analogues into Recombinant Proteins” *Methods Enzymol.* **2012**, *512*, 57–69.

[15] W. W. Tsai, Z. Wang, T. T. Yiu, K. C. Akdemir, W. Xia, S. Winter, C. Y. Tsai, X. Shi, D. Schwarzer, W. Plunkett, B. Aronow, O. Gozani, W. Fischle, M. C. Hung, D. J. Patel, M. C. Barton, “TRIM24 Links a Non-Canonical Histone Signature to Breast Cancer” *Nature* **2010**, *468*, 927–932.

[16] L. J. O’Connor, C. Cazares-Körner, J. Saha, C. N. G. Evans, M. R. L. Stratford, E. M. Hammond, S. J. Conway, “Design, Synthesis and Evaluation of Molecularly Targeted Hypoxia-Activated Prodrugs” *Nat. Protoc.* **2016**, *11*, 781–794.

[17] A. Skwarska, E. D. D. Calder, D. Sneddon, H. Bolland, M. L. Odyniec, I. N. Mistry, J. Martin, L. K. Folkes, S. J. Conway, E. M. Hammond, “Development and Pre-Clinical Testing of a Novel Hypoxia-Activated KDAC Inhibitor” *Cell Chem. Biol.* **2021**, *28*, 1258-1270.

[18] G. R. Fulmer, A. J. M. Miller, N. H. Sherden, H. E. Gottlieb, A. Nudelman, B. M. Stoltz, J. E. Bercaw, K. I. Goldberg, “NMR Chemical Shifts of Trace Impurities: Common Laboratory Solvents, Organics, and Gases in Deuterated Solvents Relevant to the Organometallic Chemist” *Organomet.* **2010**, *29*, 2176–2179.

[19] B. Hansen, R. Zahradník, K. Dušek, J. Klaban, “Kinetics of Formation and Reactions of Quaternary Ethylenimonium Compounds” *Acta. Chem. Scand.* **1962**, *16*, 1945–1955.

[20] R. Lakhan, B. J. Rai, “Synthesis and Antibacterial Activity of 2-[ω-(Dialkylamino)alkyl]thio]-3-aryl(or alkyl)-6,8-disubstituted-4(3H)-quinazolinones” *J. Chem. Eng. Data* **1987**, *32*, 384–386.

[21] R. Lakhan, O. P. Singh, “Syntheses of Some New 4(3H)-Quinazolinones as Potential CNS Active Agents” *Arch. Pharm.* **1985**, *318*, 228–238.

[22] G. Faust, W. Fiedler, “Über Reaktionen von Äthylenhalogeniden mit Primären, Sekundären und Tertiären β-Hydroxyäthylaminen” *J. Prakt. Chem.* **1963**, *21*, 113–130.

[23] S. Bhagwat, R. V. Tiu, W. Wu (Eli Lilly & Co.), WO2018005234A1, **2018**.

[24] E. G. Tse, S. D. Houston, C. M. Williams, G. P. Savage, L. M. Rendina, I. Hallyburton, M. Anderson, R. Sharma, G. S. Walker, R. S. Obach, M. H. Todd, “Nonclassical Phenyl Bioisosteres as Effective Replacements in a Series of Novel Open-Source Antimalarials” *J. Med. Chem.* **2020**, *63*, 11585–11601.

[25] C. B. Xue, J. Roderick, S. Jackson, M. Rafalski, A. Rockwell, S. Mousa, R. E. Olson, W. F. DeGrado, “Design, Synthesis, and in vitro Activities of Benzamide-Core Glycoprotein IIb/IIIa Antagonists: 2,3-Diaminopropionic Acid Derivatives as Surrogates of Aspartic Acid” *Bioorg. Med. Chem.* **1997**, *5*, 693–705.

[26] P. G. Baraldi, R. Romagnoli, M. D. C. Nuñez, M. Perretti, M. J. Paul-Clark, M. Ferrario, M. Govoni, F. Benedini, E. Ongini, “Synthesis of Nitro Esters of Prednisolone, New Compounds Combining Pharmacological Properties of Both Glucocorticoids and Nitric Oxide” *J. Med. Chem.* **2003**, *47*, 711–719.

[27] D. R. Hou, Y. D. Hsieh, Y. W. Hsieh, “New Formation of 4,5,6,7-Tetrahydroisoindoles” *Tet. Lett.* **2005**, *46*, 5927–5929.

[28] W. Xie, B. Gong, S. Ning, N. Liu, Z. Zhang, X. Che, L. Zheng, J. Xiang, “Diethyl Phosphite Promoted Electrochemical Oxidation of Tetrahydroisoquinolines to 3,4-Dihydroisoquinolin-1(2H)-ones” *Synlett* **2019**, *30*, 2077–2080.

[29] A. R. Katritzky, M. J. Mokrosz, “The Preparation of Some 1-Vinylpyridinium Salts” *Heterocycles* **1984**, *22*, 505.

[30] D. Mambwe, M. Kumar, R. Ferger, D. Taylor, M. Njoroge, D. Coertzen, J. Reader, M. Van Der Watt, L. M. Birkholtz, K. Chibale, “Structure-Activity Relationship Studies Reveal New Astemizole Analogues Active against Plasmodium falciparum In Vitro” *ACS Med. Chem. Lett.* **2021**, *12*, 1333–1341.

[31] P. López, C. G. Seipelt, P. Merkling, L. Sturz, J. Álvarez, A. Dölle, M. D. Zeidler, S. Cerdán, P. Ballesteros, “N-2-(Azol-1(2)-yl)ethyliminodiacetic Acids: a Novel Series of Gd(III) Chelators as T2 Relaxation Agents for Magnetic Resonance Imaging” *Bioorg. Med. Chem.* **1999**, *7*, 517–527.

[32] C. Blaszykowski, E. Aktoudianakis, C. Bressy, D. Alberico, M. Lautens, “Preparation of Annulated Nitrogen-Containing Heterocycles via a One-Pot Palladium-Catalyzed Alkylation/Direct Arylation Sequence” *Org. Lett.* **2006**, *8*, 2043–2045.

[33] M. A. Marx, J. G. Christensen, C. R. Smith, J. P. Fischer, A. C. Burns (Mirati Therapeutics Inc.; Array Biopharma Inc.), WO2020146613A1, **2020**.

[34] J. Geng, G. Mantovani, L. Tao, J. Nicolas, G. Chen, R. Wallis, D. A. Mitchell, B. R. G. Johnson, S. D. Evans, D. M. Haddleton, “Site-Directed Conjugation of “Clicked” Glycopolymers To Form Glycoprotein Mimics: Binding to Mammalian Lectin and Induction of Immunological Function” *J. Am. Chem. Soc.* **2007**, *129*, 15156–15163.

[35] T. A. Gillam, C. Caporale, R. D. Brooks, C. A. Bader, A. Sorvina, M. V. Werrett, P. J. Wright, J. L. Morrison, M. Massi, D. A. Brooks, S. Zacchini, S. M. Hickey, S. Stagni, and S. E. Plush, “Neutral Re(I) Complex Platform for Live Intracellular Imaging” *Inorg. Chem.* **2021** *60*, 10173–10185.

[36] J. R. Hwu, C. I. Hsu, M. H. Hsu, Y. C. Liang, R. C. C. Huang, Y. C. Lee, “Glycosylated Nordihydroguaiaretic Acids as Anti-Cancer Agents” *Bioorg. Med. Chem. Lett.* **2011**, *21*, 380–382.

[37] K. E. Ryu, B. R. Kim, G. H. Sung, H. J. Yoon, Y. J. Yoon, “Facile Synthesis of Benzo[d]azol-2(3 H)-ones Using 2-Phenoxycarbonyl-4,5-dichloropyridazin-3(2 H)-one as Green CO Source” *Synlett* **2015**, *26*, 1985–1990.

[38] N. N. Smolyar, O. Y. Pankina, A. I. Bondarenko, Y. S. Borodkin, A. I. Khizhan, “Synthesis of 1,3-Dialkyl-5-(hetaryl-1-yl)-1,3-dihydrobenzimidazol-2-ones” *Russ. J. Org. Chem.* **2011**, *47*, 1190–1193.

[39] M. T. Conconi, G. Marzaro, L. Urbani, I. Zanusso, R. Di Liddo, I. Castagliuolo, P. Brun, F. Tonus, A. Ferrarese, A. Guiotto, A. Chilin, “Quinazoline-Based Multi-Tyrosine Kinase Inhibitors: Synthesis, Modeling, Antitumor and Antiangiogenic Properties” *Eur. J. Med. Chem.* **2013**, *67*, 373–383.

[40] S. N. Kolodyazhnaya, A. M. Simonov, “Investigations in the Field of Benzimidazole Derivatives” *Chem. Heterocycl. Compd.* **1969**, *5*, 529–532.

[41] O. J. Stratton, Master’s Thesis, University of Oxford (U.K.), **2019**.

[42] E. Plettner, Y. Gong, R. Gries (Simon Fraser University), US2010190865A1, **2010**.

[43] Ž. Jakopin, M. Gobec, J. Kodela, T. Hazdovac, I. Mlinarič-Raščan, M. Sollner Dolenc, “Synthesis of Conformationally Constrained γ-d-Glutamyl-meso-diaminopimelic Acid Derivatives as Ligands of Nucleotide-Binding Oligomerization Domain Protein 1 (Nod1)” *Eur J. Med. Chem.* **2013**, *69*, 232–243.
